# CRISPR-Cas12a-Based Detection for the Major SARS-CoV-2 Variants of Concern

**DOI:** 10.1128/Spectrum.01017-21

**Published:** 2021-11-17

**Authors:** Yuanhao Liang, Hongqing Lin, Lirong Zou, Jianhui Zhao, Baisheng Li, Haiying Wang, Jing Lu, Jiufeng Sun, Xingfen Yang, Xiaoling Deng, Shixing Tang

**Affiliations:** a Department of Epidemiology, School of Public Health, Southern Medical University, Guangzhou, China; b Guangdong Provincial Institute of Public Health, Guangdong Center for Diseases Control and Prevention, Guangzhou, China; c Wenzhou Institute, University of Chinese Academy of Sciences, Wenzhou, China; d Department of Infectious Diseases, Nanfang Hospital, Southern Medical University, Guangzhou, China; University of Georgia

**Keywords:** SARS-CoV-2, variant screening, emerging SARS-CoV-2 variant, CRISPR-Cas system

## Abstract

A big challenge for the control of COVID-19 pandemic is the emergence of variants of concern (VOCs) or variants of interest (VOIs) of severe acute respiratory syndrome coronavirus 2 (SARS-CoV-2), which may be more transmissible and/or more virulent and could escape immunity obtained through infection or vaccination. A simple and rapid test for SARS-CoV-2 variants is an unmet need and is of great public health importance. In this study, we designed and analytically validated a CRISPR-Cas12a system for direct detection of SARS-CoV-2 VOCs. We further evaluated the combination of ordinary reverse transcription-PCR (RT-PCR) and CRISPR-Cas12a to improve the detection sensitivity and developed a universal system by introducing a protospacer adjacent motif (PAM) near the target mutation sites through PCR primer design to detect mutations without PAM. Our results indicated that the CRISPR-Cas12a assay could readily detect the signature spike protein mutations (K417N/T, L452R/Q, T478K, E484K/Q, N501Y, and D614G) to distinguish alpha, beta, gamma, delta, kappa, lambda, and epsilon variants of SARS-CoV-2. In addition, the open reading frame 8 (ORF8) mutations (T/C substitution at nt28144 and the corresponding change of amino acid L/S) could differentiate L and S lineages of SARS-CoV-2. The low limit of detection could reach 10 copies/reaction. Our assay successfully distinguished 4 SARS-CoV-2 strains of wild type and alpha (B.1.1.7), beta (B.1.351), and delta (B.1.617.2) variants. By testing 32 SARS-CoV-2-positive clinical samples infected with the wild type (*n* = 5) and alpha (*n* = 11), beta (*n* = 8), and delta variants (*n* = 8), the concordance between our assay and sequencing was 100%. The CRISPR-based approach is rapid and robust and can be adapted for screening the emerging mutations and immediately implemented in laboratories already performing nucleic acid amplification tests or in resource-limited settings.

**IMPORTANCE** We described CRISPR-Cas12-based multiplex allele-specific assay for rapid SARS-CoV-2 variant genotyping. The new system has the potential to be quickly developed, continuously updated, and easily implemented for screening of SARS-CoV-2 variants in resource-limited settings. This approach can be adapted for emerging mutations and implemented in laboratories already conducting SARS-CoV-2 nucleic acid amplification tests using existing resources and extracted nucleic acid.

## INTRODUCTION

Like most RNA viruses, the novel severe acute respiratory syndrome coronavirus type 2 (SARS-CoV-2) has evolved, resulting in variants with different phenotypes and public health concern (1). Most importantly, mutations on the spike (S) gene of SARS-CoV-2 may affect virus entry, infectivity, transmission, and protective immune response, which, in turn, may challenge public health systems and affect measures against COVID-19 pandemic. It has been reported that D614G substitution in the S protein can affect viral infectivity and antigenicity (2, 3). Deletion of amino acids Δ69 to 70, a signature mutation in the S gene of SARS-CoV-2 B.1.1.7 variant, caused a “spike gene target failure” (SGTF), a false-negative detection of SARS-CoV-2 S gene by PCR assay (4). Further analysis indicated that the SARS-CoV-2 variant with SGTF is associated with high viral loads (5) and exhibits a substantial quick transmission capability (6). SGTF has thus been used as a proxy of B.1.1.7 variant to monitor its spread. Furthermore, N501Y mutation on the receptor binding domain (RBD) of SARS-CoV-2 S protein is associated with increased binding affinity to angiotensin-converting enzyme 2 (ACE2) receptor (7). It has been reported that variants with the deletion of amino acids Δ69 and 70 and K417N and E484K mutations in the SARS-CoV-2 S protein are linked to immune response evasion (4, 8, 9). Interestingly, a SARS-CoV-2 variant that was originally identified among farmed minks in Denmark was found to be transmitted to human beings in six countries (10).

The rapid emergence and transmission of SARS-CoV-2 variants worldwide highlight the importance of continuous surveillance of SARS-CoV-2 mutant viruses. The World Health Organization (WHO), in collaboration with other institutions, has proposed and established nomenclature systems for naming and tracking SARS-CoV-2 genetic lineages as specific variants of interest (VOIs) and variants of concern (VOCs) (https://www.who.int/en/activities/tracking-SARS-CoV-2-variants/). The most common VOCs include alpha (B.1.1.7), beta (B.1.351, B.1.351.2, and B.1.351.3), gamma (P.1, P.1.1, P.1.2, P.1.3, P.1.4, P.1.5, P.1.6, and P.1.7), and delta (B.1.617.2, AY.1, AY.2, AY.3, and AY.3.1), while the major VOIs are epsilon (B.1.427/B.1.429), zeta (P.2), eta (B.1.525), theta (P.3), iota (B.1.526), kappa (B.1.617.1), lambda (C.37), and mu (B.162.1). It has been reported that B.1.1.7 variant spread more quickly and efficiently, while B.1.351 and P.1/P.2 variants may affect the efficiency of the SARS-CoV-2-neutralizing antibodies (11–14). Muik et al. reported that the immune sera from COVID-19 vaccinee remain effective against B.1.1.7 (15), but their effects appeared attenuated in B.1.351 and P.1 (16). Cele et al. reported that SARS-CoV-2 variant B.1.351 can escape from neutralization by convalescent plasma collected from patients infected with variant B.1.1.7 (17).

However, the current sequencing approach to trace transmission of SARS-CoV-2 variants is difficult to sustain at the population level over the long term. A simple and rapid test with genotyping capability is an unmet need to facilitate high-throughput variant surveillance in particular in resource-limiting settings. Wang et al. have recently reported a multiplex SARS-CoV-2 genotyping assay by using reverse transcription-PCR (RT-PCR) to screen three mutations (L452R, E484K, and N501Y) in the S protein (18). Similar PCR-based assays have been implemented for large-scale screening to provide important data for precise intervention and prevention of SARS-CoV-2 infection (19–22).

In the past decades, as a novel gene-editing tool, CRISPR and CRISPR-associated (Cas) proteins have led to unprecedented advances in molecular biology (23). In the past several years, CRISPR-Cas technology is also revolutionizing molecular diagnostics for both infectious diseases and noninfectious diseases and can be used to detect gene mutations in pathogens or circulating cell-free DNAs (24–27). CRISPR diagnostics has thus been called next-generation diagnostics and promises to be rapid, accurate, and portable diagnostic tools (28). In fact, several inventions based on CRISPR-Cas technology have been reported and adapted for detecting SARS-CoV-2 infection (29–36). The principle of CRISPR-Cas-based diagnosis is that CRISPR RNA (crRNA) or guided RNA (gRNA) can specifically bind the target sequences and activate Cas enzymes for both sequence-specific cutting (in *cis*) and nonspecific sequence cleavage (in *trans*). Mismatches between the crRNA and the target sequences will affect the *trans*-cleavage rate (25). Furthermore, for the CRISPR-Cas12 system, a protospacer adjacent motif (PAM) is required for target recognition and cleavage (37). We believe that CRISPR-Cas12a-mediated assay can augment gold-standard PCR-based genotyping to become a new tool to detect the most common VOCs or VOIs of SARS-CoV-2.

In this study, we designed and analytically validated a CRISPR-Cas12a system for direct detection of SARS-CoV-2 VOCs. We further evaluated the combination of ordinary RT-PCR and CRISPR-Cas12a to improve the detection sensitivity. In addition, as a proof of concept, we developed a universal system by introducing a PAM sequence near the target mutation sites through PCR primer design for detecting any mutations without adjacent PAM.

## RESULTS

### Optimization of Cas12a-mediated detection.

**FIG 1:**
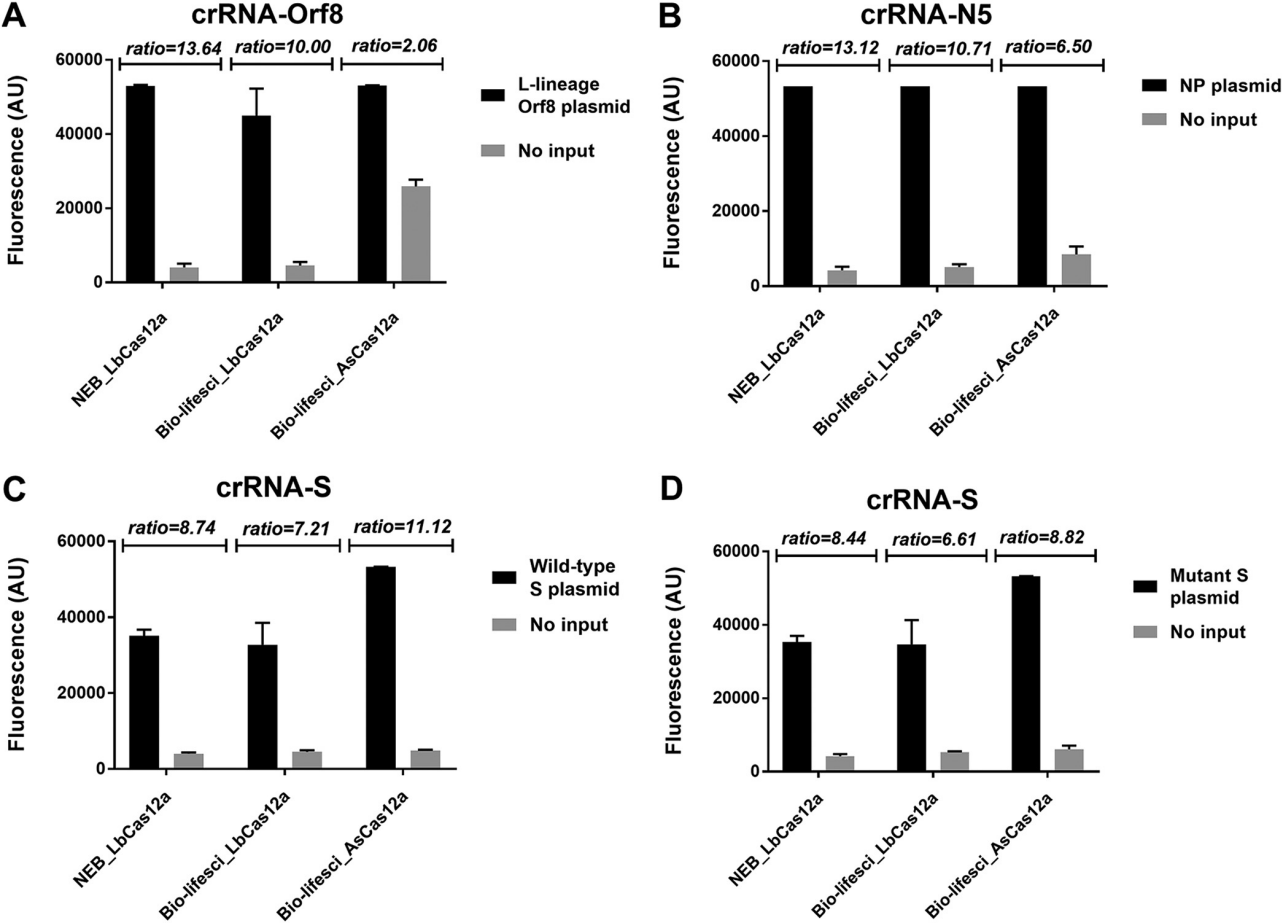
Comparison of *trans*-cleavage activity of Cas12a from different sources. The *trans*-cleavage activity of different Cas12a enzymes was analyzed in the CRISPR-Cas12 direct detection system using the mixture of crRNA and plasmid DNA for crRNA-Orf8/L lineage of Orf8 gene (A), crRNA-N5/N gene (B), crRNA-S/wild-type S gene (C), and crRNA-S/mutant S gene (D), respectively. NEB_LbCas12a and Bio-lifesci_LbCas12a were Cas12a enzymes of *Lachnospiraceae* bacterium and purchased from New England Biolabs (NEB, MA, USA) and Bio-lifesci (Guangzhou, China), respectively, while Bio-lifesci_AsCas12a represents Cas12a protein of *Acidaminococcus* sp. BV3L6 and purchased from Bio-lifesci. Fluorescence values represent mean ± standard deviation (SD) from 3 replicates of experiment. The ratio of fluorescence signal for SARS-CoV-2 plasmid DNA over no-input control is presented at the top of each panel.

We first compared the activity of different Cas12a enzymes (Fig. 1) by using the L lineage of open reading frame 8 (ORF8), nucleoprotein (NP), wild-type (WT) S, and its mutant S gene as the templates. In general, two LbCas12a enzymes from Bio-lifesci and NEB, respectively, and one AsCas12a enzyme from Bio-lifesci exhibited similar performance, although AsCas12a showed a slightly higher background when testing the L lineage of ORF8 (Fig. 1A). The fluorescence ratio of sample to control was nonsignificantly different with respect for the target sequences for the three Cas12a enzymes tested (Fig. 1). These results indicated similar *trans*-cleavage activity of Cas12a enzymes from different vendors. Therefore, NEB LBCas12a was used for further evaluation.

**FIG 2:**
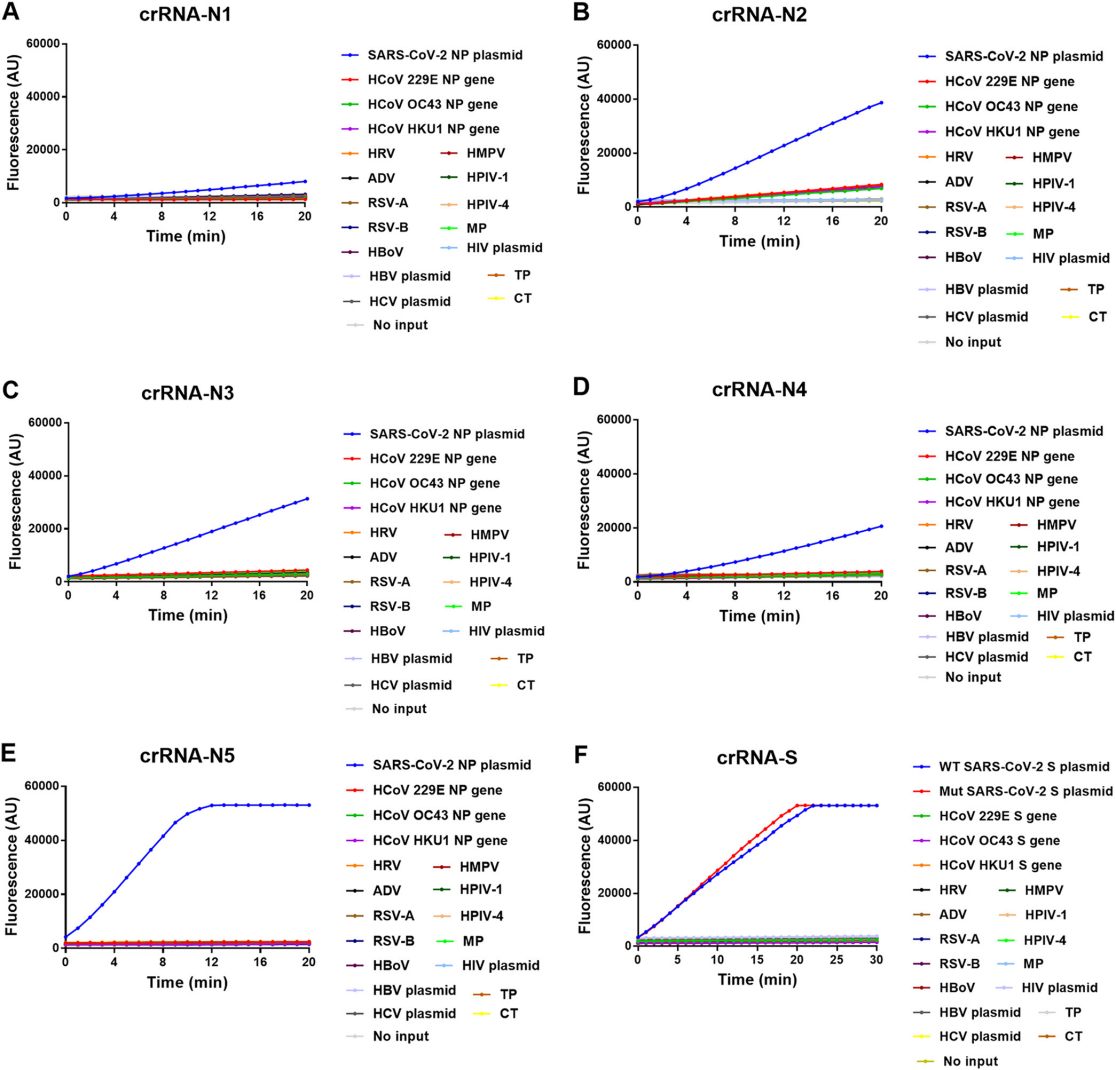
Time course reaction of CRISPR-Cas12a-mediated detection of SARS-CoV-2. The fluorescence was measured at different time points using 10^10^ copies/μl SARS-CoV-2 nucleoprotein (NP) plasmid DNA as the template and different NP-specific guide RNAs crRNA-N1 (A), crRNA-N2 (B), crRNA-N3 (C), crRNA-N4 (D), and crRNA-N5 (E), respectively, or using 10^10^ copies/μl wild-type and mutant spike (S) gene as the template and guide RNA crRNA-S (F). In each panel, assay specificity was validated with nucleic acid samples of common human coronavirus (HCoV) 229E, HCoV OC43, and HCoV HKU1, rhinovirus (HRV), adenovirus (ADV), respiratory syncytial virus (RSV) A and B, human bocavirus (HBoV), human metapneumovirus (HMPV), human parainfluenza virus (HPIV-1 and HPIV-4), and Mycoplasma pneumoniae, as well as HIV-1, HBV, HCV, Chlamydia trachomatis, and Treponema pallidum. The mean value of fluorescence of three independent experiments is presented. No input indicates negative control of no-plasmid DNA.

To determine the capability of various crRNAs in the DNA direct detection system, we designed 5 crRNAs (crRNA-N1, crRNA-N2, crRNA-N3, crRNA-N4, and crRNA-N5) for SARS-CoV-2 N gene and 1 crRNA (crRNA-S) specific for both WT and mutant S gene (Table S4 in the supplemental material). The results showed that all the 6 crRNAs can specifically bind the target sequences but exhibited different levels of fluorescence signals (Fig. 2). crRNA N5 showed the quickest reaction among the 5 NP crRNAs analyzed and reached the plateau of fluorescence signal around 10 min (Fig. 2E). Furthermore, the crRNA-S could detect both WT and mutant S gene with relatively high signal intensity and reached the peak of signal around 20 min (Fig. 2F). These results indicate the different performances of crRNAs in CRISPR Cas12a-mediated direct detection.

**FIG 3:**
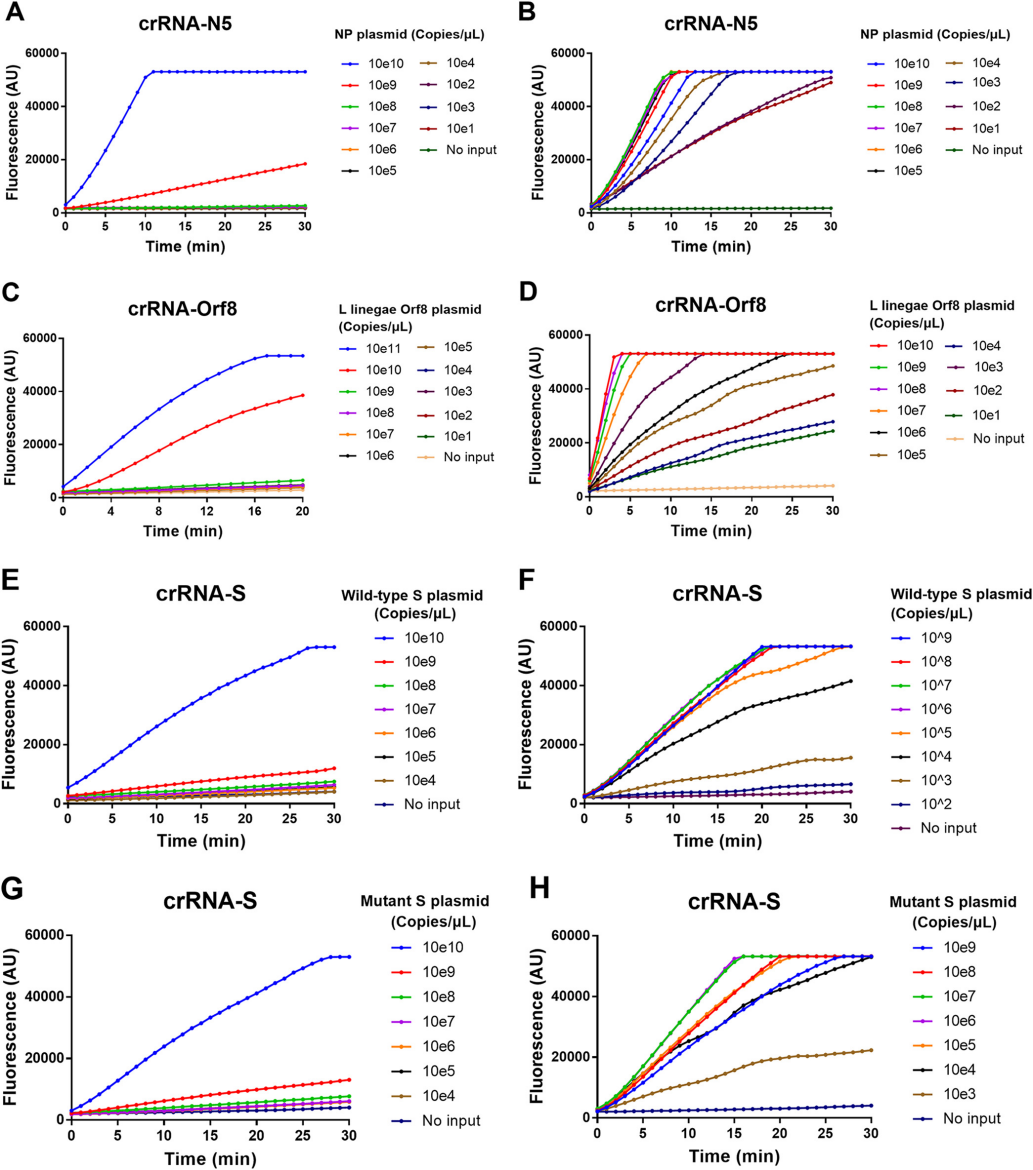
Low limit of detection (LOD) for CRISPR-Cas12a direct detection (left panels) and the integrated PCR/CRISPR-Cas12a detection (right panels). (A to H) Fluorescence was measured at different time points using different amount of SARS-CoV-2 plasmid DNA of nucleoprotein (A and B), ORF8 (C and D), and wild type (E and F) or mutant (G and H) spike gene as the template. No input indicates negative control of no-plasmid DNA. The mean value of fluorescence of three independent experiments is presented.

Then, we adopted crRNA-N5, ORF8, and S to determine the low limit of detection (LOD) of Cas12a-mediated DNA detection assay using 10-fold serial dilutions of the target DNA template, which ranged from 10^11^ to 10 copies/μl. We found that there was a very good linear relationship between the reaction time and fluorescence intensity, although the fluorescence signal reached a plateau around 20 to 25 min in the presence of a high concentration of target templates. The LODs for crRNA-N5, ORF8, and S-mediated assay were 10^9^, 10^10^, and 10^10^ copies/μl, respectively (Fig. 3A, C, E, and G). Obviously, the LOD for the Cas12a-mediated direct detection without a preamplification step of the target template could not satisfy the practical application since the mean viral loads in nasal swabs of COVID-19 patients are 1.4 × 10^3^ copies/μl (38). We thus further evaluated whether multiple crRNAs targeting different regions of the SARS-CoV-2 genome could improve the detection sensitivity as Fozouni et al. reported in the CRISPR-Cas13 system (32). We found that the mixture of two crRNAs of N5 and S slightly increased the fluorescence value but did not significantly improve the LOD (Fig. S2). We then coupled PCR and Cas12a-mediated detection to determine the LOD (Fig. 3B, D, F, and H). As expected, in the presence of target preamplification, as few as 10 copies/μl of NP gene (Fig. 3B) or ORF8 gene (Fig. 3D) of SARS-CoV-2 could be readily detected, while 1,000 copies/μl of the WT (Fig. 3F) and mutant S gene (Fig. 3H) were detected. Therefore, compared with the Cas12a-mediated direct detection, the combination of PCR and Cas12a-mediated detection dramatically improved the LOD by 10^7^ to 10^8^ fold. In addition, the detection time was shortened from over 30 min to 5 to 10 min.

### Cas12a-mediated detection of SARS-CoV-2 mutations.

**FIG 4:**
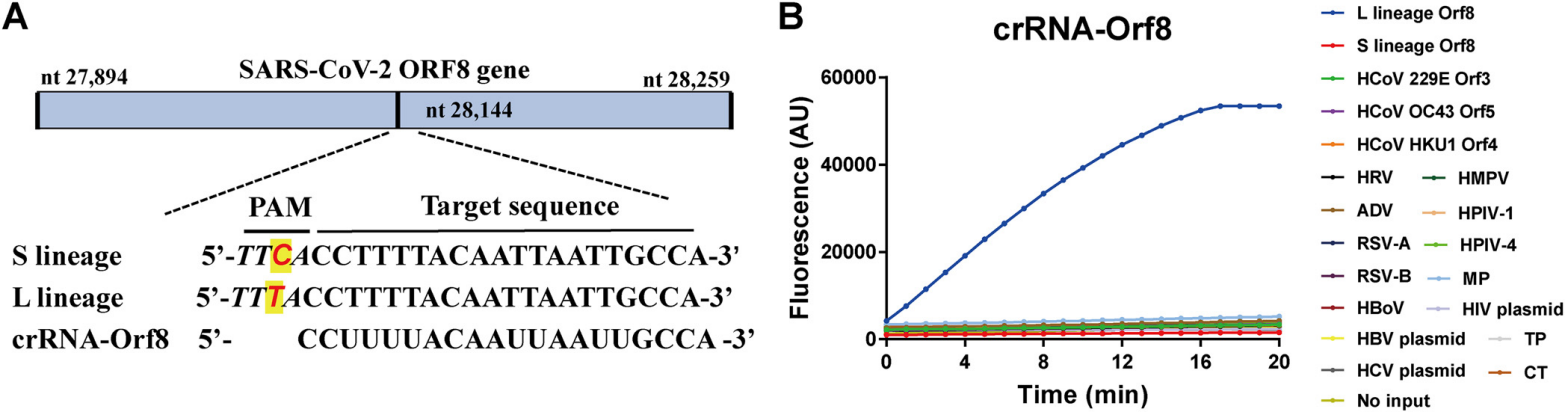
CRISPR-Cas12a-mediated direct detection of SARS-CoV-2 mutations. (A) Schematic of the SARS-CoV-2 Orf8 gene and the mutation for S and L lineage of SARS-CoV-2. The sequences and positions of the target gene ORF8, the guide RNA crRNA-Orf8, and the protospacer adjacent motif (PAM) are indicated. (B) Fluorescence was measured at different time points using 10^11^ copies/μl plasmid DNA of SARS-CoV-2 L and S lineage as the template. Assay specificity was validated with nucleic acid samples of nucleic acid samples of common human coronavirus (HCoV) 229E, HCoV OC43, and HCoV HKU1, rhinovirus (HRV), adenovirus (ADV), respiratory syncytial virus (RSV) A and B, human bocavirus (HBoV), human metapneumovirus (HMPV), human parainfluenza virus (HPIV-1 and HPIV-4) and Mycoplasma pneumoniae, as well as HIV-1, HBV, HCV, Chlamydia trachomatis, and Treponema pallidum. The mean value of fluorescence of three independent experiments is presented. No input indicates negative control of no plasmid DNA.

The CRISPR-Cas12a-based detection relies on the *trans*-cleavage activity of Cas12a protein activated upon complementary binding of the crRNA to the specific target. Mismatches between them could inhibit the activation of Cas12a and its *trans*-cleavage activity and could be used to detect mutations of SARS-CoV-2. To test this principle, we select the L and S lineages of SARS-CoV-2 as an example. The two viral strains of L and S lineage are characterized with a single nucleotide mutation of C/T at nt28144 in the SARS-CoV-2 ORF8 (39). Interestingly, the C/T mutation results in the loss of PAM sequence in the ORF8 S lineage (TTC), but not in the L lineage (TTT) (Fig. 4A). Binding of crRNA-ORF8 with ORF8 sequence activated Cas12a and cleaved the fluorescence probe, so we could see the increasing fluorescence intensity over time for the L lineage template but no fluorescence signal for S lineage template (Fig. 4B), indicating the capacity of Cas12a-mediated assay to specifically distinguish L and S lineage of SARS-CoV-2.

**FIG 5:**
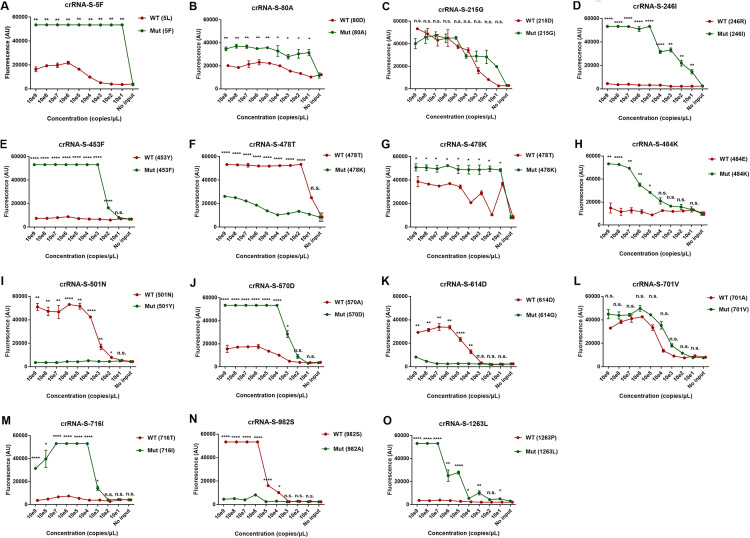
Detection of the single nucleotide mutations across SARS-CoV-2 spike protein by PCR-CRISPR/Cas12a-mediated assay. Different concentrations of SARS-CoV-2 DNA were used as the template and amplified by PCR followed by detection of CRISPR-Cas12a-mediated assay. Wild type (WT) and mutant (Mut) template are labeled in red and green, respectively. The position and name of the amino acid for the templates are indicated in the brackets. The name of the crRNA and the corresponding mutant amino acid were put at the top of each panel. The fluorescence values represent the mean ± standard deviation (SD) of 3 replicates. Two-tailed student's *t* test was used to analyze the difference between wild-type and mutant template. n.s., not significant; *, *P* < 0.05; **, *P* < 0.01; ****, *P* < 0.0001; WT, wild type; Mut, mutant; no input, negative control with no plasmid DNA.

We designed 20 specific crRNAs, including crRNA-S-5F, S-80A, S-215G, S-246I, S-417N, S-452R, S-452Q, S-453F, S-478K, S-484K, S-484Q, S-570D, S-701V, S-716I, and S-1263L specific for the mutant S gene and crRNA-S-417K, S-478T, S-501N, S-614D, and S-982S for the wild-type S gene (Table S4) to differentially detect mutant strains of SARS-CoV-2. We combined PCR preamplification of the template and the Cas12a-mediated detection and found that, except for crRNA-S-215G (Fig. 5C) and crRNA-S-701V (Fig. 5L), the rest of the corresponding crRNAs are able to specifically differentiate the wild-type S gene from the mutant S gene. These results indicated that our system could specifically distinguish the templates with signature mutations at the S gene of SARS-CoV-2 (Fig. 5).

### A universal PCR/Cas12a-mediated detection of SARS-CoV-2 mutations.

**FIG 6:**
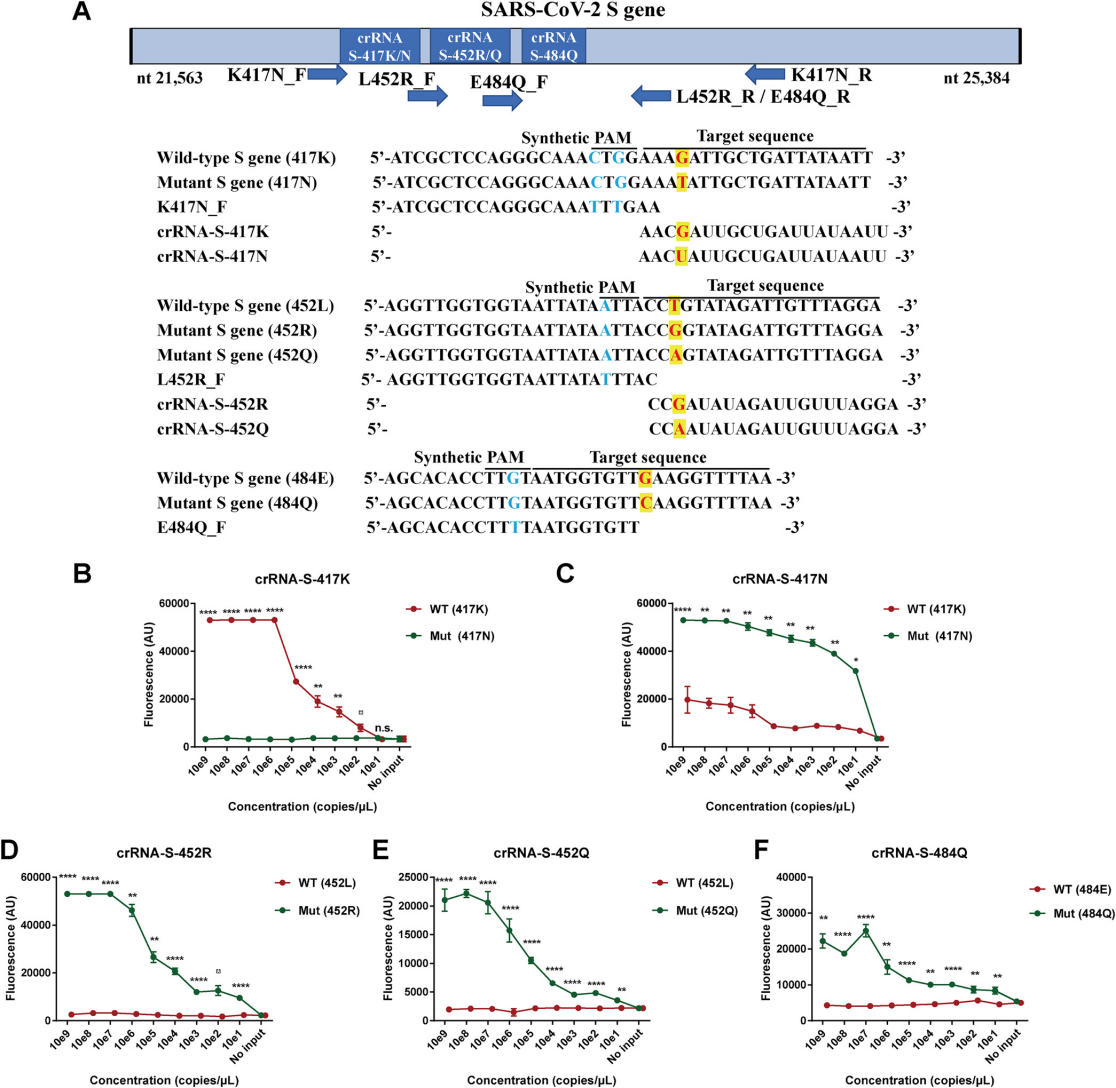
Strategy of adding protospacer adjacent motif (PAM) near mutation sites by using PCR primers to promote CRISPR/Cas12a-mediated detection of SARS-CoV-2 mutants. (A) Schematic of the SARS-CoV-2 spike (S) genome, forward primer with PAM sequence, and the sequences and locations of the corresponding guide RNAs crRNA-S-417K, crRNA-S-417N, crRNA-S-452R, crRNA-S-452Q, and crRNA-S-484Q for SARS-CoV-2 mutants 417N, 452R/Q, and 484Q, respectively. Different concentrations of SARS-CoV-2 DNA were used as the template and amplified by PCR with the aforementioned forward primers followed by detection of CRISPR-Cas12a-mediated assay to detect the single nucleotide mutation of K417N (B and C), L452R (D), L452Q (E), and E484Q (F), respectively. The fluorescence values represent the mean ± standard deviation (SD) of 3 replicates. Two-tailed Student's *t* test was used to analyze the difference between wild type and mutant template. n.s., not significant; *, *P* < 0.05; **, *P* < 0.01; ****, *P* < 0.0001; WT, wild type; Mut, mutant; no input, negative control with no plasmid DNA.

We know that the activation and catalytic activity of Cas12a depend on the sequences of both PAM motif and crRNA. In practice, it is impossible to always find the PAM motif near the mutation sites analyzed or mutations at the potential PAM motif to inhibit the efficiency of Cas12a-mediated detection as we showed for the S lineage of SARS-CoV-2 ORF8 (Fig. 4). Therefore, we proposed to introduce a PAM sequence upstream of the crRNA-cDNA sequence by designing a specific PCR primer containing the PAM motif. The PCR products amplified by the aforementioned primer will always insert a PAM motif right before the crRNA sequence and can be detected by Cas12a-mediated cleavage. In this study, we used the proposed strategy to add PAM motif upstream of the three mutations K417N, L452R/Q, and E484Q that are characteristic mutations present in the S gene of beta, delta, and lambda variants of SARS-CoV-2 (Fig. 6A; Fig. S1) with the specific PCR primers (Table S5). As shown in Fig. 6, 100 copies of the wild-type (417K) strain could be distinguished from 10^9^ copies of the K417N mutation (Fig. 6B), while 10 copies of the mutant strains (K417N, L452R/Q, or E484Q) could be readily distinguished from 10^9^ copies of the wild-type (417K, 452L, or 484E) strain (Fig. 6C to F). These results indicated that trace amounts of the target template could be specifically detected and distinguished from a large amount of the sequences with mutations.

### Detection of SARS-CoV-2 mutations using RT-PCR/Cas12a-mediated assay.

**FIG 7:**
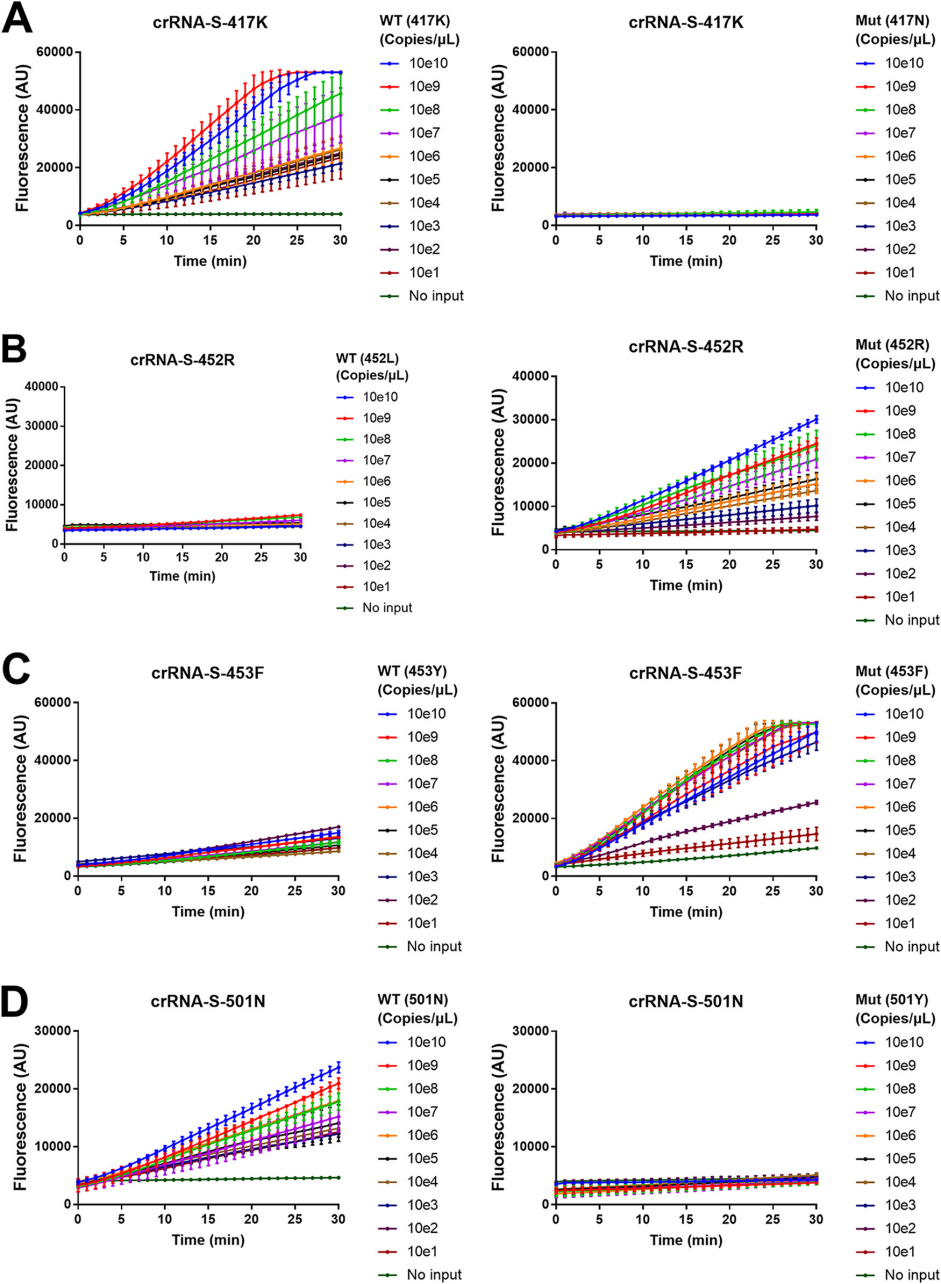
Combination of RT-PCR and CRISPR-Cas12a-mediated detection to detect SARS-CoV-2 mutations. A series of 10-fold diluted *in vitro*-transcribed SARS-CoV-2 RNAs was used as the templates for RT-PCR followed by detection of CRISPR-Cas12a-mediated assay using guide RNAs for crRNA-S-417K (A), crRNA-S-452R (B), crRNA-S-453F (C), and crRNA-S-501N (D), respectively. The position and name of the amino acid for the templates are indicated in brackets. The fluorescence was measured at different time points and presented as the mean ± standard deviation (SD) from 3 replicates. WT, wild type; Mut, mutant; no input, negative control with no plasmid DNA.

Until now, all the experiments were done using SARS-CoV-2 plasmid DNA as the template but not SARS-CoV-2 RNA. To validate the system for RNA detection, we used serially diluted *in vitro*-transcribed WT and mutant SARS-CoV-2 S gene RNA (10^10^ to 10 copies/μl) to simulate virus RNA to validate a RT-PCR/Cas12a-mediated assay. We designed 19 crRNAs to detect the corresponding 19 mutations in the SARS-CoV-2 S gene and chose 4 crRNAs to evaluate our assay because (i) all of these 4 mutations are located in the receptor binding domain (RBD) and may affect the fusion and internalization of the virus with host cells (40) and detection of these mutations has clear clinical implication (2); (ii) the crRNAs cover both wild-type S gene (crRNA-S-417K and crRNA-S-501N) and mutant S gene (crRNA-S-452R and crRNA-S-453F) (Table S4); and (iii) the PAM sequence is naturally available upstream of two mutations (Y453F, N501Y) and has to be artificially introduced in another two mutations (K417N, L452R) by using PCR primers. Our data revealed that all 4 mutations could be readily distinguished in our RT-PCR/Cas12a-mediated assay (Fig. 7). Both crRNA-S-417K (Fig. 7A) and crRNA-S-501N (Fig. 7D) could distinguish 10 copies of the wild-type S gene from 10^10^ copies of the mutant S gene, while 10^3^ copies and 10^2^ copies of the mutant S gene could be distinguished from 10^10^ copies of the wild-type S gene by using crRNA-S-452R (Fig. 7B) and crRNA-S-453F (Fig. 7C), respectively. These results demonstrated that the combined RT-PCR and CRISP-Cas12a system could detect the SARS-CoV-2 RNA template.

### Screening of SARS-CoV-2 mutant viruses using RT-PCR/Cas12a-mediated assay.

**FIG 8:**
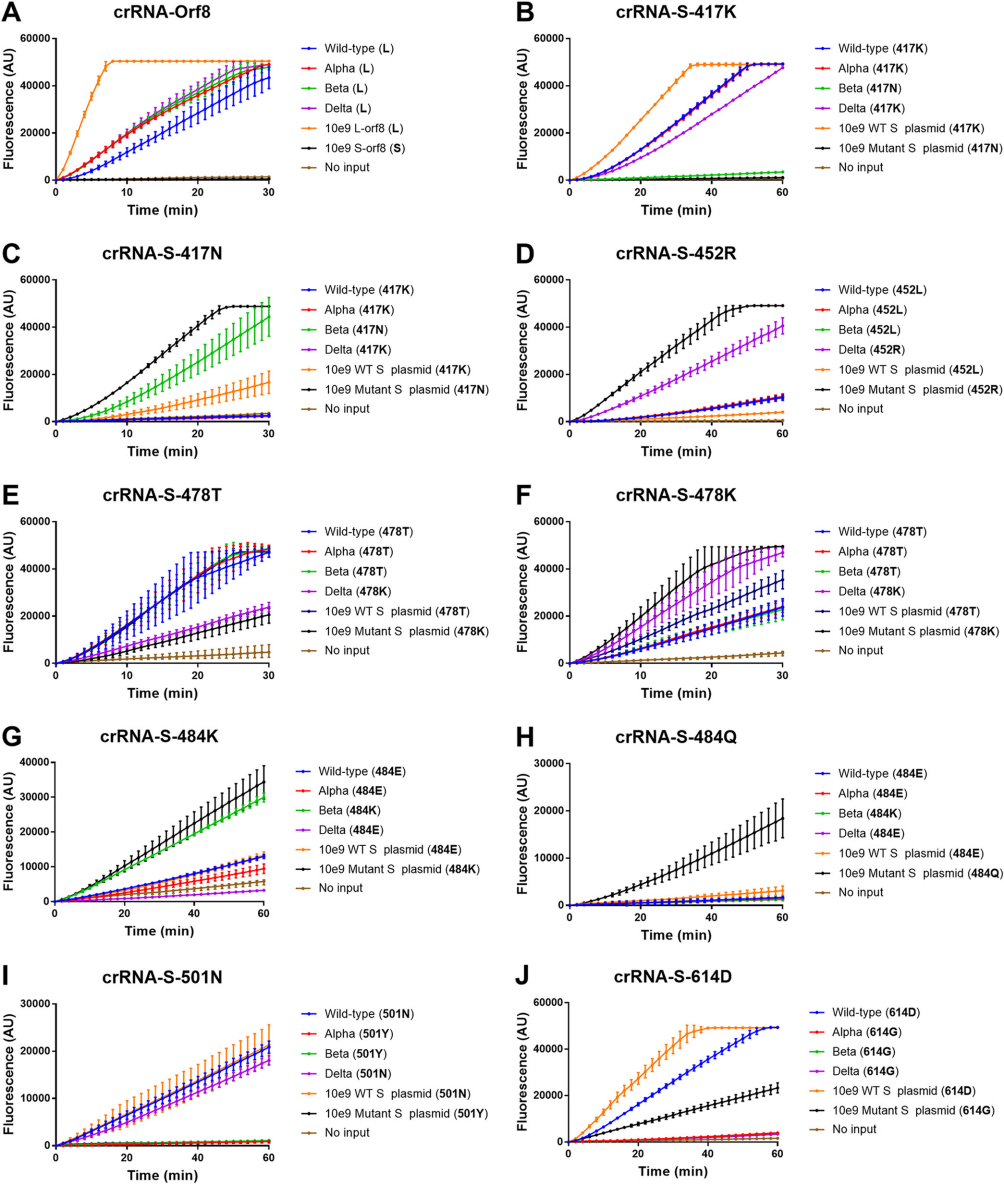
CRISPR-Cas12a-based detection of SARS-CoV-2 variants. Viral RNA templates were extracted from SARS-CoV-2 wild-type strain as well as alpha, beta, and delta SARS-CoV-2 variants of concern and amplified by RT-PCR followed by CRISPR-Cas12a-mediated detection by using crRNA-Orf8 (A), crRNA-S-417K (B), crRNA-S-417N (C), crRNA-S-452R (D), crRNA-S-487T (E), crRNA-S-487K (F), crRNA-S-484K (G), crRNA-S-484Q (H), crRNA-S-501N (I) and crRNA-S-614D (J) for the corresponding mutations. The position and name of the amino acid for the templates are indicated in the brackets. The plasmid DNA for S-lineage Orf8 (S-Orf8; 10^9^ copies/μl) and L lineage Orf8 (L-Orf8, 10^9^ copies/μl) gene is used as control in panel A. The plasmid DNA for wild-type (WT, 10^9^ copies/μl) and mutant spike (S, 10^9^ copies/μl) genes was used as control in the remaining panel B to J. The fluorescence was measured at different time points and presented as the mean ± standard deviation (SD) from 3 replicates. No input, negative control with no plasmid DNA.

**FIG 9:**
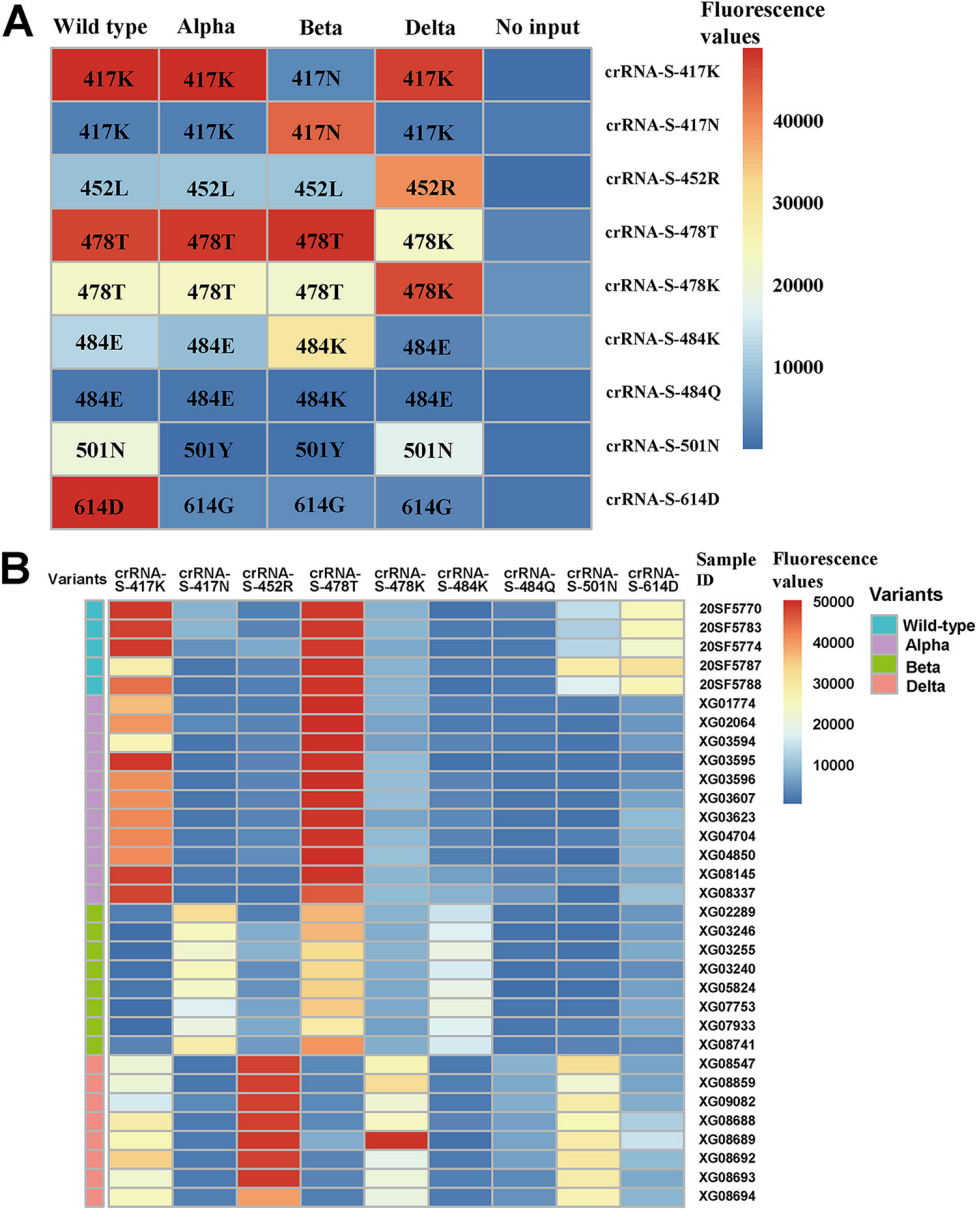
Heatmap of detection results for SARS-CoV-2. (A) Four virus isolates, including SARS-CoV-2 wild-type strain and alpha, beta, and delta variants were evaluated in CRISPR-Cas12a system using mutation specific crRNAs, i.e., cr-RNA-S-417K, crRNA-S-417N, crRNA-S-452R, crRNA-S-478T, crRNA-S-478K, and crRNA-S-484K, crRNA-S-484Q, crRNA-S-501N, and crRNA-S-614D. The corresponding signature residues detected by the specific crRNAs are indicated in the boxes of the heatmap. (B) Thirty-two clinical samples in a panel of 5 wild-type strains, 11 alpha variants, 8 beta variants, and 8 delta variants were evaluated in CRISPR-Cas12a system using mutation-specific crRNAs. The scale bar shows the range of fluorescence values represented by color change from blue to red.

We further validated the performance of our system in discriminating 4 SARS-CoV-2 strains isolated from clinical samples, including a wild-type strain from Wuhan, China, and alpha, beta, and delta variants isolated from imported COVID-19 patients. S lineage SARS-CoV-2 that emerged in the early stages of the COVID-19 outbreak in China has been replaced by the L lineage of the SARS-CoV-2 ORF8 gene around the world (39). Our results indicated that all the 4 SARS-CoV-2 viruses analyzed in this study can be classified into L lineage (Fig. 8A). Since the K417N mutation of the S protein only appears in the beta variant, the 417K-specific crRNA (crRNA-S-417K) could not recognize the beta variant and the negative control of the mutant S gene with the K417N mutation. No fluorescence signal was observed for the beta variant and mutant S (K417N) control. In contrast, strong fluorescence was recorded in the wild-type strain and alpha and delta variants as well as WT S plasmid DNA control (Fig. 8B). Furthermore, by using crRNA-S-417N specific for 417N, different results were observed between the beta variant or mutant S (K417N) control and the wild-type strain and alpha and delta variants, although moderate fluorescence signal was detected in the WT S plasmid DNA control (Fig. 8C). Similar results were observed for the L452R mutation for the delta variant only (Fig. 8D) and the N501Y mutation for both the wild type and delta variant (Fig. 8I). In addition, we designed crRNAs crRNA-S-478T and crRNA-S-478K specific for 478T and 478K, respectively, and observed different levels of fluorescence signals when detecting SARS-CoV-2 wild-type virus and variants, but the difference was not so robust (Fig. 8E and F). Furthermore, crRNA-S-484K could readily detect the beta variant with the E484K mutation (Fig. 8G). Since the kappa variant (B.1.617.1) carries E484Q mutations, we designed crRNA-S-484Q and found that it could clearly detect the mutant S plasmid DNA with the E484Q mutation (Fig. 8H). Finally, crRNA-S-614D was used to distinguish the D614G mutation. We found that it could only detect the wild-type virus and the wild-type S plasmid DNA with the 614D amino acid, although a moderate level of fluorescence was observed in the mutant S plasmid DNA with 614G (Fig. 8F). However, no fluorescence signal was observed in three SARS-CoV-2 variants (alpha, beta, and delta) with 614G and the no-DNA negative control (Fig. 8J). The detection results were visualized in a heatmap (Fig. 9A) and summarized in Table 1 and showed that the combined data could readily distinguish SARS-CoV-2 wild-type virus and the variants analyzed. We have further validated our assay by detecting 32 clinical samples in a panel of 5 wild-type strains, 11 alpha variants, 8 beta variants, and 8 delta variants; the results are presented in Fig. 9B and show 100% concordance with the sequencing approach.

**TABLE 1:** Detection of SARS-CoV-2 variants by CRISPR-Cas12a-based assay^*a*^

SARS-CoV-2 variant	Location	Signature mutations in spike protein	CRISPR-Cas12a-based assay using crRNAs:									
			S-417K	S-417N	S-452R	S-452Q	S-478T	S-478K	S-484K	S-484Q	S-501N	S-614D
Wild type	China	NA	+	−	−	−	+	−	−	−	+	+
Alpha	United Kingdom	N501Y, A570D, D614G	+	−	−	−	+	−	−	−	−	−
Epsilon	United States	L452R, D614G	+	−	+	−	+	−	−	−	−	−
Beta	South Africa	K417N, E484K, N501Y, D614G	−	+	−	−	+	−	+	−	−	−
Gamma	Brazil and Japan	K417T, E484K, N501Y, D614G	−	−	−	−	+	−	+	−	−	−
Kappa	India	L452R, E484Q, D614G	+	−	+	−	+	−	−	+	+	−
Delta	India	L452R, T478K, D614G	+	−	+	−	−	+	−	−	+	−
Lambda	Peru	L452Q, F490S, D614G	+	−	−	+	+	−	−	−	+	−

a NA, not applicable; +, robust fluorescence intensity; −, mild or no fluorescence intensity.

## DISCUSSION

CRISPR technology is being evaluated as a testing platform for SARS-CoV-2 infection (29, 32, 41–43) mainly because of the *cis*- and *trans*-cleavage activities of Cas12 and Cas13 effectors (44–47), which exhibit high specificity and sensitivity due to multiple-turnover signal amplification in the absence of preamplification of target sequences (48, 49). In our study, we developed a CRISPR-Cas12a-based genotyping assay and have approved its feasibility to detect single nucleotide mutations and to distinguish variants of SARS-CoV-2 based on the combination of a series of crRNAs that are specific for the most important and signature mutations, including K417N, L452R/Q, T478K, E484K/Q, and N501Y within the spike protein of SARS-CoV-2. As a proof of concept, three current WHO-classified variants of concern, i.e., alpha (B.1.1.7), beta (B.1.351), and delta (B.1.617.2), were readily detected and differentiated (Table 1) since they all harbor some of the signature mutations. In theory, the list of crRNAs for any emerging SARS-CoV-2 mutations could be easily updated to fulfill the need for a test that provides rapid results and can be administered immediately.

There are several major advantages of the CRISPR-Cas-based genotyping system. The whole testing could be finished at room temperature by replacing PCR with isothermal amplification such as recombinase polymerase amplification (24–27). In most CRISPR-based assays, the readout is fluorescence signal, but the results could also be visualized by naked eyes, detected by mobile phone (32), or presented in a later flow strip (27, 29, 50). Therefore, it could be a rapid point-of-care assay for both diagnosis and epidemiologic surveillance of SARS-CoV-2 variants. The later application is of great public health interest. In addition, CRISPR-based genotyping can be easily integrated into the current nucleic acid amplification systems without a significant increase in the cost and detection time since the equipment, reagents, and facilities can be shared, and the same extracted nucleic acid can be used. In fact, our assay is not only complementary to the diagnosis of SARS-CoV-2 infection to provide extra information about SARS-CoV-2 variants but also possible to fulfill the purpose of diagnosis by including a crRNA for detecting the highly conserved SARS-CoV-2 N gene in our system. In the future, the assay could be refined for dual diagnosis and genotyping of SARS-CoV-2 infection by using microfluidic chips, digital PCR, or other technologies (Fig. S3 in the supplemental material). Furthermore, we provide a new idea to use PCR primer to introduce PAM near the mutation sites where no PAM sequence is available, making the system more feasible for any emerging mutations and variants.

Except for our CRISPR-Cas12a method, RT-PCR and genome sequencing are usually used for characterizing mutations and genotyping, with their advantages and limitations (Table S6). PCR-based methods for detecting specific mutations of SARS-CoV-2 have been reported (19–22). Vogels et al. developed multiplex qPCR by using deletion of amino acids Δ3675 to 3677 in the ORF1a and deletion of amino acids Δ69 to 70 in the S gene to differentiate B.1.1.7, B.1.351, and P.1 variants (21). Rosa et al. also reported the identification of SARS-CoV-2 VOCs that were originally found in the United Kingdom, Brazil, and Spain in sewage samples, highlighting the importance of wastewater surveillance to explore SARS-CoV-2 diversity (20). Moria et al. reported a spike protein-based immunoassay to differentiate alpha (B.1.1.7) and beta (B.1.351) variants by using 4 monoclonal antibodies targeting various epitopes of SARS-CoV-2 spike protein (51). However, few methods or algorithms have been described for the genotyping of SARS-CoV-2 VOCs and VOIs. Our study proved the CRISPR-based genotyping method for the most common VOCs or VOIs of SARS-CoV-2.

Of note, Fozouni et al. recently reported an amplification-free CRISPR-Cas13a assay for direct detection of SARS-CoV-2 with a low limit of detection of 100 copies/ml by using multiple crRNAs to increase detection sensitivity (32). Direct detection without preamplification of target sequences is indeed a promising option for rapid point-of-care testing, but its sensitivity remains to be confirmed. Our preliminary results indicated that CRISPR-Cas12a direct detection could detect ∼10^9^ copies of plasmid DNA per reaction in under 30 min of measurement time. Previous studies also indicated a low detection limit of 10 pM target DNAs in a direct Cas12a assay (52, 53) or fM-level RNA in Cas13a assay (32). To improve the detection limit, Yue et al. described a droplet Cas amplification-free assay in picoliter-sized droplets to increase local molecule concentration and enhance reaction efficiency (54). They demonstrated a low limit of 17.5 copies/μl virus DNA in their system (55). Shi et al. described CRISPR-Cas12a only amplification network (CONAN) as a novel target amplification free detection with attomolar sensitivity of genomic DNA (42). We believe that further refinement of the CRISPR-Cas12 system by using a nucleic acid amplification-free method and nanodroplet reaction system could improve the detection sensitivity and simplify the detection.

One major limitation of our study is the lack of validation of a large size of clinical samples. Major validation data were obtained by using synthetic DNA templates with specific mutations of SARS-CoV-2. Only 4 virus isolates for SARS-CoV-2 variants and 32 clinical samples of SARS-CoV-2 wild type and 3 VOCs were tested (Fig. 9). In addition, the algorithm for genotyping SARS-CoV-2 variants remains to be determined by analyzing a large number of clinical samples infected with SARS-CoV-2 variants. The construction of a large sample panel with well-characterized SARS-CoV-2 variants is needed for the development and validation of SARS-CoV-2 variant genotyping. As a proof of concept, we proved the feasibility of CRISPR-Cas12 system for virus genotyping. However, our assay is limited to effectively distinguish currently known variants of SARS-CoV-2. Given that SARS-CoV-2 is evolving and novel mutations are emerging, the assays and crRNAs for specific mutations and variants of SARS-CoV-2 therefore remain to be refined and updated. We believe that the time to update the assay probes depends on the knowledge and importance of the potential novel variants. A simple way is to follow up the list of variants of concern or interests of SARS-CoV-2 published by WHO. For example, the lambda (C.37) variant with a unique L452Q substitution has been put into the list of VOIs by WHO in June 2021 and is predominantly circulating in South America (56). In addition, L452R mutation in spike protein has already been reported in other variants, including delta (B.1.617.2), epsilon (B.1.429), and kappa (B.1.617.1) variants. However, the L452Q substitution is almost exclusive to the lambda (C.37) variant. We have thus refined our CRISPR-Cas12a system to detect the lambda strain of SARS-CoV-2 by adding a crRNA S-452Q specific for 452Q (Fig. 6E). The testing of crRNA for the Mu variant is ongoing.

Based on sequence data available on GISAID, SARS-CoV-2 RBD and S1 domain as a whole can tolerate additional mutations in the regions that already harbor mutations, which, in turn, may create new combinations and multiple mutants, i.e., triple or even quadruple mutants. The potential multiple mutants occur sporadically even among the VOCs of SARS-CoV-2 and may result in mixed readouts based on the current approach of our assay. One approach to resolve this issue is to design a microfluidic chip or use a digital PCR platform to detect multiple mutations simultaneously and to develop an algorithm to classify the variants with various mutations. In the case of novel mutants, unclassified variants should only be characterized by genome sequencing.

In our study, we noticed the inefficiency of some crRNAs in differentiating SARS-CoV-2 variants, although they may work well when testing synthetic plasmid DNA. These results indicate that it is necessary to further evaluate the crRNAs by using clinical samples. It is also important to optimize the criteria for selecting crRNAs. At least, the crRNAs for diagnosis must be highly conserved among the virus isolates and may not be the same as those for genotyping.

In conclusion, we described a CRISPR-Cas12-based multiplex allele-specific assay for rapid SARS-CoV-2 variant genotyping. The new system has the potential to be quickly developed, continuously updated, and easily implemented for screening of SARS-CoV-2 variants in resource-limited settings. This approach can be adapted for emerging mutations and implemented in laboratories already conducting SARS-CoV-2 nucleic acid amplification technique (NAT) using existing resources and extracted nucleic acid.

## MATERIALS AND METHODS

### Plasmid DNA and virus RNA of SARS-CoV-2.

SARS-CoV-2 genome sequences of different variants from different regions were downloaded from NCBI GenBank database and aligned to determine the target sequences for developing CRISPR Cas12a-based assay to detect SARS-CoV-2 mutants (Table S1 and Fig. S1 in the supplemental material). The residues are highly conserved among the same variants from different geographic locations (Fig. S1), suggesting that it is possible to find highly conserved sequences to develop a molecular test and to ensure that the molecular test could be used in clinical labs worldwide. The target sequences of SARS-CoV-2 are synthesized and subcloned into the vector pUC57 as recombinant plasmids at Sangon Biotech (Shanghai, China). The SARS-CoV-2 target sequences include (i) the wild-type (WT) gene fragment of S protein (S; nucleotides [nt] 21,563 to 25,384; GenBank accession number MN908947); (ii) the mutant gene fragments of S protein, including mutations L5F, D80A, D215G, R246I, K417N, L452R/Q, Y453F, T478K, E484Q/K, N501Y, A570D, D614G, P681H, A701V, T716I, S982A, D1118H, and P1263L; (iii) the S lineage gene fragment of open reading frame 8 (ORF8; nt 27,894 to 28,259; GenBank accession no. MN908947); (iv) the L lineage fragment of ORF8 with mutation of S84L; and (v) the nucleoprotein (NP) gene fragment (nt 28,274 to 29,533; GenBank accession no. MN908947). All the target sequences used for plasmid construction are available in Table S2. Recombinant plasmid DNAs were quantified using a NanoDrop 2000 spectrophotometer (Thermo Fisher Scientific, MA, USA). The plasmid copy number was calculated using the following formula: plasmid copy number (copies/μl) = [6.02 × 10^23^ × plasmid concentration (ng/μl) × 10^−9^]/(plasmid length × 660).

Both WT and the mutant S gene were amplified using T7 promoter-tagged primer and reverse transcribed using the HiScribe T7 high-yield RNA synthesis kit (New England Biolabs, MA, USA) and then purified using the miRNeasy serum/plasma kit (Qiagen, Hilden, Germany). *In vitro*-transcribed WT and mutant SARS-CoV-2 S gene RNA were quantified using a NanoDrop 2000 spectrophotometer (Thermo Fisher Scientific, MA, USA) with concentrations of 7.2 × 10^11^ copies/μl and 9.7 × 10^11^ copies/μl, respectively. The RNAs were serially 10-fold diluted to prepare a series template with 10^10^ to 10 copies/μl, aliquoted, and stored at −80°C until use.

The SARS-CoV-2 wild-type strain (19A) isolated from a COVID-19 patient in Wuhan, China, and variants alpha (B1.1.7), beta (B.1.351), and delta (B1.617.2) isolated from imported COVID-19 patients were grown in Vero cells. Viral RNA was extracted by TRIzol reagent (Invitrogen, Carlsbad, CA), aliquoted, and stored at −80°C until use. Furthermore, 30-two SARS-CoV-2-positive clinical samples, including 5 wild-type strains, 11 alpha variants, 8 beta variants, and 8 delta variants were collected to evaluate the assay performance. The majority of these clinical samples were from imported COVID-19 patients from different countries and regions (Table S3). SARS-CoV-2 strains have been characterized by sequencing.

### Design and synthesis of crRNA.

The crRNAs were designed to specifically target the mutation residues based on the alignment analysis of multiple SARS-CoV-2 genome sequences of different VOCs or VOIs from different regions (Fig. S1). We evaluated two Cas12a enzymes, i.e., LbCas12a and AsCas12a, which are expressed in *Lachnospiraceae* bacterium (Lb) and *Acidaminococcus* sp. BV3L6 (As), respectively. The mixture of Cas12a and guide RNA (crRNA) with PAM (5′-TTTN-3′, where N refers to A/G/C) specifically binds the target dsDNA to activate Cas12a to cleave the target DNA sequence. For preparation of each crRNA, DNA oligonucleotides (T7-gRNA-oligonucleotide) containing T7 promoter, conserved stem-loop sequences, and guide sequences were synthesized from Ruiboxingke Biotechnology (Beijing, China) and transcribed *in vitro* using the HiScribe T7 high-yield RNA synthesis kit (New England Biolabs, MA, USA) according to the manufacturer’s instructions. To purify crRNAs, the transcription reactions were treated with 4 units of DNase I (New England Biolabs, MA, USA) at 37°C for 40 min and then purified using the miRNeasy serum/plasma kit (Qiagen, Hilden, Germany). These crRNAs were measured by a NanoDrop 2000 spectrophotometer, aliquoted, and stored at −80°C until use. All the crRNA sequences used in this study are available in Table S4.

### Cas12a-mediated fluoresce assay.

The CRISPR-Cas12a-mediated fluoresce assay for direct detection of the target has been previously described (29). In brief, 400 nM LbCas12a (New England Biolabs, MA, USA) or AsCas12a (Bio-lifesci, Guangzhou, China) was preincubated with 1,000 nM crRNA in 1× NEB buffer 2.1 at 37°C for 10 min to form crRNA-Cas12a complex followed by addition of various amounts of target DNA and 400 nM probe reporter (5′-6-FAM-TTATT-BHQ-1-3′), synthesized at Sangon Biotech (Shanghai, China), and incubated at 37°C for 1 h. Then, the fluorescence signal was monitored every 20 s on a fluorescent detector (Qitian, Jiangsu, China).

To validate the assay specificity, we tested common human coronavirus (HCoV) 229E, HCoV OC43, and HCoV HKU1 as well as various other respiratory pathogens, including rhinovirus (HRV), adenovirus (ADV), respiratory syncytial virus (RSV) A and B, human bocavirus (HBoV), human metapneumovirus (HMPV), human parainfluenza virus (HPIV-1 and HPIV-4), and Mycoplasma pneumoniae. These samples were kindly provided by Jincun Zhao of State Key Laboratory of Respiratory Disease, Guangzhou Medical University (Guangzhou, Guangdong, China). Assay specificity was also validated with HIV-1 (plasmid pNL4-3; 73 ng/μl), hepatitis B virus (HBV), hepatitis C virus (HCV) (plasmid JFH; 114 ng/μl), Chlamydia trachomatis, and Treponema pallidum. The HBV DNA was extracted from the serum samples included in the national reference materials for HBV detection kit (National Institutes for Food and Drug Control, China). The C. trachomatis genome DNA was extracted from the cervical secretion of C. trachomatis-infected patients, and the T. pallidum genome DNA was extracted from the T. pallidum strain isolated from T. pallidum-infected rabbit.

### Combination of PCR and Cas12a-mediated fluoresce assay.

Target nucleic acid sequences were first amplified by conventional PCR for DNA templates or by reverse transcription-PCR (RT-PCR) for RNA templates. The RNA template was reverse transcribed into cDNA using oligo(dT) and random primer according to the manufacturer’s instructions (Roche Diagnostics, Indianapolis, USA). To ensure the primers to account for the accumulation of diversity in the virus population, we have performed alignment analysis of multiple SARS-CoV-2 genome sequences using MAFFT version 7 (57). SARS-CoV-2 genome sequences of different variants from different regions were downloaded from the NCBI GenBank database, and the detailed information is provided in Table S1. Moreover, primers used in our assay were evaluated and validated as SARS-CoV-2 specific (target specific) by NCBI Primer-BLAST (58). The primer sequences are available in Table S5. Briefly, 12.5 μl ApexHF HS DNA polymerase FS mix (Accurate Biotechnology, Hunan, China), 1 μl of forward and reverse primers (10 μM), 8.5 μl of nuclease-free H_2_O, and 1 μl of target template were mixed. The reaction was run at 98°C for 30 s followed by 40 cycles of 98°C for 10 s, 55°C for 15 s, and 72°C for 30 s. Finally, 1 μl of PCR product was mixed with crRNA-LbCas12a complex (1,000 nM crRNA and 400 nM LbCas12a, respectively) and 400 nM probe reporter (5′-6-FAM-TTATT-BHQ-1-3′) and incubated at 37°C for 1 h. The fluorescence was measured every 20 s on a fluorescent detector.

### Statistical analysis.

Data were analyzed using R software version 3.5.2 (R Foundation for Statistical Computing). A two-tailed Student's *t* test was used to analyze the fluorescence difference between on-target and off-target templates detected by CRISPR-Cas12a-based assay. A *P* value of <0.05 was considered statistically significant.
