# Supplementary material for: CRISPR-Cas12a-Based Detection for the Major SARS-CoV-2 Variants of Concern
Source: Microbiol Spectr. 2021 Nov 17;9(3):e01017-21. doi: 10.1128/Spectrum.01017-21 (PMC8597640; doi:10.1128/Spectrum.01017-21)
Supplement: SUPPLEMENTAL FILE 1 — Supplemental material. Download SPECTRUM01017-21_Supp_1_seq14.pdf, PDF file, 0.4 MB [file spectrum01017-21_supp_1_seq14.pdf]

Supplementary Table S1. Detailed information of SARS-CoV-2 genomes used for multiple alignment

Accession	Pangolin lineage	WHO classification	Category	Length (bp)	Location
MN908947	B			29903	China
MW913362	B.1.1.7	Alpha	variant of concern	29763	Austria: Upper Austria, Voecklabruck
OU514166	B.1.1.7	Alpha	variant of concern	29810	Germany:Europe/Germany/Baden-Wurttemberg
OU514176	B.1.1.7	Alpha	variant of concern	29813	Germany:Europe/Germany/Baden-Wurttemberg
MZ350105	B.1.1.7	Alpha	variant of concern	29756	Ghana
MZ350109	B.1.1.7	Alpha	variant of concern	29773	Ghana
MZ266365	B.1.1.7	Alpha	variant of concern	29763	Hong Kong
BS001137	B.1.1.7	Alpha	variant of concern	29863	Japan: Tochigi
BS001138	B.1.1.7	Alpha	variant of concern	29862	Japan: Tochigi
BS001139	B.1.1.7	Alpha	variant of concern	29877	Japan: Tochigi
BS001140	B.1.1.7	Alpha	variant of concern	29862	Japan: Tochigi
OU538849	B.1.1.7	Alpha	variant of concern	29884	Switzerland:SO
OU538851	B.1.1.7	Alpha	variant of concern	29884	Switzerland:BE
MZ888516	B.1.1.7	Alpha	variant of concern	29882	Thailand: Kamphaeng Phet
OU562607	B.1.1.7	Alpha	variant of concern	29884	United Kingdom:Scotland
OU562608	B.1.1.7	Alpha	variant of concern	29884	United Kingdom:Scotland
MZ895877	B.1.1.7	Alpha	variant of concern	29521	USA: Washington
MZ298839	B.1.351	Beta	variant of concern	29764	Hong Kong
MZ298840	B.1.351	Beta	variant of concern	29764	Hong Kong
MW981442	B.1.351	Beta	variant of concern	29848	South Africa
MZ202314	B.1.351	Beta	variant of concern	29783	South Africa
MZ376663	B.1.351	Beta	variant of concern	29848	South Africa
OU539352	B.1.351	Beta	variant of concern	29885	Switzerland:AG

OU525106	B.1.351	Beta	variant of concern	29885	United Kingdom:England
MZ879365	B.1.351	Beta	variant of concern	29782	USA: Colorado
MZ913100	B.1.351	Beta	variant of concern	29712	USA: New Mexico
LC643036	B.1.617.2	Delta	variant of concern	29875	Japan: Tokyo
LC643044	B.1.617.2	Delta	variant of concern	29878	Japan: Tokyo
MZ854386	B.1.617.2	Delta	variant of concern	29830	Sierra Leone
MZ854387	B.1.617.2	Delta	variant of concern	29831	Sierra Leone
MZ854389	B.1.617.2	Delta	variant of concern	29830	Sierra Leone
MZ853946	B.1.617.2	Delta	variant of concern	29890	South Korea
OU539261	B.1.617.2	Delta	variant of concern	29893	Switzerland:TG
OU539690	B.1.617.2	Delta	variant of concern	29890	Switzerland:ZH
MZ888532	B.1.617.2	Delta	variant of concern	29835	Thailand: Kamphaeng Phet
MZ888533	B.1.617.2	Delta	variant of concern	29809	Thailand: Kamphaeng Phet
OU562629	B.1.617.2	Delta	variant of concern	29890	United Kingdom:England
MZ896136	B.1.617.2	Delta	variant of concern	29516	USA: Oregon
MZ477746	P.1	Gamma	variant of concern	29741	Brazil: Parana
MZ477748	P.1	Gamma	variant of concern	29738	Brazil: Parana
MW938104	P.1	Gamma	variant of concern	29874	Peru
OU539784	P.1	Gamma	variant of concern	29894	Switzerland:AG
OU539830	P.1	Gamma	variant of concern	29894	Switzerland:GR
OU539894	P.1	Gamma	variant of concern	29894	Switzerland:ZH
OU492255	P.1	Gamma	variant of concern	29898	United Kingdom:England
OU565182	P.1	Gamma	variant of concern	29898	United Kingdom:Scotland
MZ896179	P.1	Gamma	variant of concern	29531	USA: Oregon
MZ896359	P.1	Gamma	variant of concern	29827	USA: Minnesota

MZ896669	P.1	Gamma	variant of concern	29808	USA: California
MZ911221	B.1.427	Epsilon	variant of interest	29864	USA: Arizona, Pima
MZ375854	B.1.427	Epsilon	variant of interest	29870	Mexico: B.C.
MZ375858	B.1.427	Epsilon	variant of interest	29833	Mexico: B.C.
MZ943834	B.1.429	Epsilon	variant of interest	29780	USA: California
MZ687447	B.1.429	Epsilon	variant of interest	29838	USA: Tennessee, Davidson county
OU282717	B.1.429	Epsilon	variant of interest	29903	United Kingdom:Wales
OU235436	B.1.429	Epsilon	variant of interest	29903	United Kingdom:England
MZ277390	B.1.429	Epsilon	variant of interest	29891	Taiwan
MZ746235	B.1.617.1	Kappa	variant of interest	29791	USA: Utah
MZ724425	B.1.617.1	Kappa	variant of interest	29788	India
MZ571142	B.1.617.1	Kappa	variant of interest	29818	Morocco
MZ562746	B.1.617.1	Kappa	variant of interest	29837	India: Madhya Pradesh
MZ538882	B.1.617.1	Kappa	variant of interest	29823	USA: FL
OU322235	B.1.617.1	Kappa	variant of interest	29559	Germany:Europe/Germany/Berlin
OU290035	B.1.617.1	Kappa	variant of interest	29893	United Kingdom:England
MZ487800	B.1.617.1	Kappa	variant of interest	29809	USA: DC
OU265178	B.1.617.1	Kappa	variant of interest	29902	United Kingdom:Wales
OU247018	B.1.617.1	Kappa	variant of interest	29902	United Kingdom:England
OU254949	B.1.617.1	Kappa	variant of interest	29902	United Kingdom:Wales
MZ397173	B.1.617.1	Kappa	variant of interest	29830	Myanmar
MZ397175	B.1.617.1	Kappa	variant of interest	29812	Myanmar
MZ397176	B.1.617.1	Kappa	variant of interest	29822	Myanmar
MZ397177	B.1.617.1	Kappa	variant of interest	29823	Myanmar
OU168413	B.1.617.1	Kappa	variant of interest	29795	Germany:Europe/Germany/Saxony

OU172184	B.1.617.1	Kappa	variant of interest	29835	Switzerland:SO
OU182966	B.1.617.1	Kappa	variant of interest	29850	Switzerland:BL
MZ363839	B.1.617.1	Kappa	variant of interest	29801	India
MZ332524	B.1.617.1	Kappa	variant of interest	29801	India
MZ332527	B.1.617.1	Kappa	variant of interest	29801	India
OU061269	B.1.617.1	Kappa	variant of interest	29902	United Kingdom:Scotland
LC633761	B.1.617.1	Kappa	variant of interest	29890	Japan
MZ026886	B.1.617.1	Kappa	variant of interest	29525	Bahrain
MZ953185	C.37	Lambda	variant of interest	29805	USA: California
MZ953277	C.37	Lambda	variant of interest	29801	USA: Massachusetts
MZ937666	C.37	Lambda	variant of interest	29379	USA: Illinois
MZ942542	C.37	Lambda	variant of interest	29653	USA: Kentucky
MZ908711	C.37	Lambda	variant of interest	29785	USA: Virginia
MZ857990	C.37	Lambda	variant of interest	29750	USA: Tennessee
OU517038	C.37	Lambda	variant of interest	29795	Germany:Europe/Germany/Hesse
OU356036	C.37	Lambda	variant of interest	29873	United Kingdom:England
OU351967	C.37	Lambda	variant of interest	29873	Switzerland:GE
MZ496613	C.37	Lambda	variant of interest	29794	Peru
OU268528	C.37	Lambda	variant of interest	29873	Switzerland:GE
OU100999	C.37	Lambda	variant of interest	29752	Germany:Europe/Germany/Berlin
OU053018	C.37	Lambda	variant of interest	29873	United Kingdom:England
MZ275295	C.37	Lambda	variant of interest	29781	Peru
MZ275301	C.37	Lambda	variant of interest	29798	Peru

Supplementary Table S2. Summary of DNA target sequences used for plasmid construction

Gene fragments	Length (bp)	Sequence (5'→3')
Wild-type S gene	3822	ATGTTTGTTTTTTTTGTTTTATTGCCACTAGTCTCTAGTCAGTGTGTTAATCTTACAACCAGAACTCAATTACCCCCTGCATACAC<br>TAATTCTTTCACACGTGGTGTTTATTACCCTGACAAAGTTTTCAGATCCTCAGTTTTACATTCAACTCAGGACTTGTTCTTACCTT<br>TCTTTTCCAATGTTACTTGGTTCCATGCTATACATGTCTCTGGGACCAATGGTACTAAGAGGTTTGCTAACCCTGTCCTACCATTT<br>AATGATGGTGTTTATTTTGCTTCCACTGAGAAGTCTAACATAATAAGAGGCTGGATTTTTGGTACTACTTTAGATTCGAAGACCC<br>AGTCCCTACTTATTGTTAATAACGCTACTAATGTTGTTATTAAAGTCTGTGAATTTCAATTTTGTAATGATCCATTTTTGGGTGTT<br>TATTACCACAAAAACAACAAAAGTTGGATGGAAAGTGAGTTCAGAGTTTATTCTAGTGCGAATAATTGCACTTTTGAATATGTC<br>TCTCAGCCTTTTCTTATGGACCTTGAAGGAAAACAGGGTAATTTCAAAAATCTTAGGGAATTTGTGTTTAAGAATATTGATGGTT<br>ATTTTAAAATATATTCTAAGCACACGCCTATTAATTTAGTGCGTGGTCTCCCTCAGGGTTTTTCGGCTTTAGAACCATTGGTAGA<br>TTTGCCAATAGGTATTAACATCACTAGGTTTCAAACTTTACTTGCTTTACATATAAGTTATTTGACTCCTGGTGATTCTTCTTCAG<br>GTTGGACAGCTGGTGCTGCAGCTTATTATGTGGGTTATCTTCAACCTAGGACTTTTCTATTAAAATATAATGAAAATGGAACCAT<br>TACAGATGCTGTAGACTGTGCACTTGACCCTCTCTCAGAAACAAAGTGTACGTTGAAATCCTTCACTGTAGAAAAAGGAATCTA<br>TCAAACTTCTAACTTTAGAGTCCAACCAACAGAATCTATTGTTAGATTTCCTAATATTACAAACTTGTGCCCTTTTGGTGAAGTT<br>TTTAACGCCACCAGATTTGCATCTGTTTATGCTTGGAACAGGAAGAGAATCAGCAACTGTGTTGCTGATTATTCTGTCCTATATA<br>ATTCCGCATCATTTTCCACTTTTAAGTGTTATGGAGTGTCTCCTACTAAATTAAATGATCTCTGCTTTACTAATGTCTATGCAGAT<br>TCATTTGTAATTAGAGGTGATGAAGTCAGACAAATCGCTCCAGGGCAAACTGGAAACATTGCTGATTATAATTATAAATTACCA<br>GATGATTTTACAGGCTGCGTTATAGCTTGGAATTCTAACAATCTTGATTCTAAGGTTGGTGGTAATTATAATTACCTGTTTAGAT<br>TGTTTAGGAAGTCTAATCTCAAACCTTTTGAGAGAGATATTTCAACTGAAATCTATCAGGCCGGTAGCACACCTTGTAATGGTG<br>TTAAAGGTTTTAATTGTTACTTTCCTTTACAATCATATGGTTTCCAACCCACTTATGGTGTTGGTTACCAACCATACAGAGTAGT<br>AGTACTTTCTTTTGAACTTCTACATGCACCAGCAACTGTTTGTGGACCTAAAAAGTCTACTAATTTGGTTAAAAACAAATGTGTC<br>AATTTCAACTTCAATGGTTTAACAGGCACAGGTGTTCTTACTGAGTCTAACAAAAAGTTTCTGCCTTTCCAACAATTTGGCAGAG

		ACATTGATGACACTACTGATGCTGTCCGTGATCCACAGACACTTGAGATTCTTGACATTACACCATGTTCTTTTGGTGGTGTCAG<br>TGTTATAACACCAGGAACAAATACTTCTAACCAGGTTGCTGTTCTTTATCAGGGTGTTAACTGCACAGAAGTCCCTGTTGCTATT<br>CATGCAGATCAACTTACTCCTACTTGGCGTGTTTATTCTACAGGTTCTAATGTTTTTCAAACACGTGCAGGCTGTTTAATAGGGG<br>CTGAACATGTCAACAACTCATATGAGTGTGACATACCCATTGGTGCAGGTATATGCGCTAGTTATCAGACTCAGACTAATTCTC<br>ATCGGCGGGCACGTAGTGTAGCTAGTCAATCCATCATTGCCTACACTATGTCACTTGGTGTAGAAAATTCAGTTGCTTACTCTAA<br>TAACTCTATTGCCATACCCATAAATTTTACTATTAGTGTTACCACAGAAATTCTACCAGTGTCTATGACCAAGACATCAGTAGAT<br>TGTACAATGTACATTTGTGGTGATTCAACTGAATGCAGCAATCTTTTGTTGCAATATGGCAGTTTTTGTACACAATTAAACCGTG<br>CTTTAACTGGAATAGCTGTTGAACAAGACAAAAACACCCAAGAAGTTTTTGCACAAGTCAAACAAATTTACAAAACACCACCA<br>ATTAAAGATTTTGGTGGTTTTAATTTTTCACAAATATTACCAGATCCATCAAAACCAAGCAAGAGGTCATTTATTGAAGATCTAC<br>TTTTCAACAAAGTGACACTTGCAGATGCTGGCTTCATCAAACAATATGGTGATTGCCTTGGTGATATTGCTGCTAGAGACCTCAT<br>TTGTGCACAAAAGTTTAACGGCCTTACTGTTTTGCCACCTTTGCTCACAGATGAAATGATTGCTCAATACACTTCTGCACTGTTA<br>GCGGGTACAATCACTTCTGGTTGGACCTTTGGTGCAGGTGCTGCATTACAAATACCATTTGCTATGCAAATGGCTTATAGGTTTA<br>ATGGTATTGGAGTTACACAGAATGTTCTCTATGAGAACCAAAAATTGATTGCCAACCAATTTAATAGTGCTATTGGCAAAATTC<br>AAGACTCACTTTCTTCCACAGCAAGTGCACTTGGAAAACTTCAAGATGTGGTCAACCAAAATGCACAAGCTTTAAACACGCTTG<br>TTAAACAACTTAGCTCCAATTTTGGTGCAATTTCAAGTGTTTTAAATGATATCCTTGCACGTCTTGACAAAGTTGAGGCTGAAGT<br>GCAAATTGATAGGTTGATCACAGGCAGACTTCAAAGTTTGCAGACATATGTGACTCAACAATTAATTAGAGCTGCAGAAATCA<br>GAGCTTCTGCTAATCTTGCTGCTACTAAAATGTCAGAGTGTGTACTTGGACAATCAAAAAGAGTTGATTTTTGTGGAAAGGGCT<br>ATCATCTTATGTCCTTCCCTCAGTCAGCACCTCATGGTGTAGTCTTCTTGCATGTGACTTATGTCCCTGCACAAGAAAAGAACTT<br>CACAACTGCTCCTGCCATTTGTCATGATGGAAAAGCACACTTTCCTCGTGAAGGTGTCTTTGTTTCAAATGGCACACACTGGTTT<br>GTAACACAAAGGAATTTTTATGAACCACAAATCATTACTACACACAACACATTTGTGTCTGGTAACTGTGATGTTGTAATAGGA<br>ATTGTCAACAACACAGTTTATGATCCTTTGCAACCTGAATTAGACTCATTCAAGGAGGAGTTAGATAAATATTTTAAGAATCAT<br>ACATCACCAGATGTTGATTTAGGTGACATCTCTGGCATTAATGCTTCAGTTGTAAACATTCAAAAAGAAATTGACCGCCTCAAT<br>GAGGTTGCCAAGAATTTAAATGAATCTCTCATCGATCTCCAAGAACTTGGAAAGTATGAGCAGTATATAAAATGGCCATGGTAC<br>ATTTGGCTAGGTTTTATAGCTGGCTTGATTGCCATAGTAATGGTGACAATTATGCTTTGCTGTATGACCAGTTGCTGTAGTTGTC<br>TCAAGGGCTGTTGTTCTTGTGGATCCTGCTGCAAATTTGATGAAGACGACTCTGAGCTAGTGCTCAAAGGAGTCAAATTACATT<br>ACACATAA

**Mutant S gene**	**3822**	ATGTTTGTTTTTTTTGTTTTATTGCCACTAGTCTCTAGTCAGTGTGTTAATCTTACAACCAGAACTCAATTACCCCCTGCATACAC<br>TAATTCTTTCACACGTGGTGTTTATTACCCTGACAAAGTTTTCAGATCCTCAGTTTTACATTCAACTCAGGACTTGTTCTTACCTT<br>TCTTTTCCAATGTTACTTGGTTCCATGCTATACATGTCTCTGGGACCAATGGTACTAAGAGGTTTGCTAACCCTGTCCTACCATTT<br>AATGATGGTGTTTATTTTGCTTCCACTGAGAAGTCTAACATAATAAGAGGCTGGATTTTTGGTACTACTTTAGATTCGAAGACCC<br>AGTCCCTACTTATTGTTAATAACGCTACTAATGTTGTTATTAAAGTCTGTGAATTTCAATTTTGTAATGATCCATTTTTGGGTGTT<br>TATTACCACAAAAACAACAAAAGTTGGATGGAAAGTGAGTTCAGAGTTTATTCTAGTGCGAATAATTGCACTTTTGAATATGTC<br>TCTCAGCCTTTTCTTATGGACCTTGAAGGAAAACAGGGTAATTTCAAAAATCTTAGGGAATTTGTGTTTAAGAATATTGATGGTT<br>ATTTTAAAATATATTCTAAGCACACGCCTATTAATTTAGTGCGTGGTCTCCCTCAGGGTTTTTCGGCTTTAGAACCATTGGTAGA<br>TTTGCCAATAGGTATTAACATCACTAGGTTTCAAACTTTACTTGCTTTACATATAAGTTATTTGACTCCTGGTGATTCTTCTTCAG<br>GTTGGACAGCTGGTGCTGCAGCTTATTATGTGGGTTATCTTCAACCTAGGACTTTTCTATTAAAATATAATGAAAATGGAACCAT<br>TACAGATGCTGTAGACTGTGCACTTGACCCTCTCTCAGAAACAAAGTGTACGTTGAAATCCTTCACTGTAGAAAAAGGAATCTA<br>TCAAACTTCTAACTTTAGAGTCCAACCAACAGAATCTATTGTTAGATTTCCTAATATTACAAACTTGTGCCCTTTTGGTGAAGTT<br>TTTAACGCCACCAGATTTGCATCTGTTTATGCTTGGAACAGGAAGAGAATCAGCAACTGTGTTGCTGATTATTCTGTCCTATATA<br>ATTCCGCATCATTTTCCACTTTTAAGTGTTATGGAGTGTCTCCTACTAAATTAAATGATCTCTGCTTTACTAATGTCTATGCAGAT<br>TCATTTGTAATTAGAGGTGATGAAGTCAGACAAATCGCTCCAGGGCAAACTGGAAATATTGCTGATTATAATTATAAATTACCA<br>GATGATTTTACAGGCTGCGTTATAGCTTGGAATTCTAACAATCTTGATTCTAAGGTTGGTGGTAATTATAATTACCGGTTTAGAT<br>TGTTTAGGAAGTCTAATCTCAAACCTTTTGAGAGAGATATTTCAACTGAAATCTATCAGGCCGGTAGCAAACCTTGTAATGGTG<br>TTCAAGGTTTTAATTGTTACTTTCCTTTACAATCATATGGTTTCCAACCCACTTATGGTGTTGGTTACCAACCATACAGAGTAGT<br>AGTACTTTCTTTTGAACTTCTACATGCACCAGCAACTGTTTGTGGACCTAAAAAGTCTACTAATTTGGTTAAAAACAAATGTGTC<br>AATTTCAACTTCAATGGTTTAACAGGCACAGGTGTTCTTACTGAGTCTAACAAAAAGTTTCTGCCTTTCCAACAATTTGGCAGAG<br>ACATTGATGACACTACTGATGCTGTCCGTGATCCACAGACACTTGAGATTCTTGACATTACACCATGTTCTTTTGGTGGTGTCAG<br>TGTTATAACACCAGGAACAAATACTTCTAACCAGGTTGCTGTTCTTTATCAGGGTGTTAACTGCACAGAAGTCCCTGTTGCTATT<br>CATGCAGATCAACTTACTCCTACTTGGCGTGTTTATTCTACAGGTTCTAATGTTTTTCAAACACGTGCAGGCTGTTTAATAGGGG<br>CTGAACATGTCAACAACTCATATGAGTGTGACATACCCATTGGTGCAGGTATATGCGCTAGTTATCAGACTCAGACTAATTCTC<br>ATCGGCGGGCACGTAGTGTAGCTAGTCAATCCATCATTGCCTACACTATGTCACTTGGTGTAGAAAATTCAGTTGCTTACTCTAA<br>TAACTCTATTGCCATACCCATAAATTTTACTATTAGTGTTACCACAGAAATTCTACCAGTGTCTATGACCAAGACATCAGTAGAT

		TGTACAATGTACATTTGTGGTGATTCAACTGAATGCAGCAATCTTTTGTTGCAATATGGCAGTTTTTGTACACAATTAAACCGTGCTTTAACTGGAATAGCTGTTGAACAAGACAAAAACACCCAAGAAGTTTTTGCACAAGTCAAACAAATTTACAAAACACCACCAATTAAAGATTTTGGTGGTTTTAATTTTTCACAAATATTACCAGATCCATCAAAACCAAGCAAGAGGTCATTTATTGAAGATCTACTTTTCAACAAAGTGACACTTGCAGATGCTGGCTTCATCAAACAATATGGTGATTGCCTTGGTGATATTGCTGCTAGAGACCTCATTTGTGCACAAAAGTTTAACGGCCTTACTGTTTTGCCACCTTTGCTCACAGATGAAATGATTGCTCAATACACTTCTGCACTGTTAGCGGGTACAATCACTTCTGGTTGGACCTTTGGTGCAGGTGCTGCATTACAAATACCATTTGCTATGCAAATGGCTTATAGGTTTAATGGTATTGGAGTTACACAGAATGTTCTCTATGAGAACCAAAAATTGATTGCCAACCAATTTAATAGTGCTATTGGCAAAATTCAAGACTCACTTTCTTCCACAGCAAGTGCACTTGGAAAACTTCAAGATGTGGTCAACCAAAATGCACAAGCTTTAAACACGCTTGTTAAACAACTTAGCTCCAATTTTGGTGCAATTTCAAGTGTTTTAAATGATATCCTTGCACGTCTTGACAAAGTTGAGGCTGAAGTGCAAATTGATAGGTTGATCACAGGCAGACTTCAAAGTTTGCAGACATATGTGACTCAACAATTAATTAGAGCTGCAGAAATCAGAGCTTCTGCTAATCTTGCTGCTACTAAAATGTCAGAGTGTGTACTTGGACAATCAAAAAGAGTTGATTTTTGTGGAAAGGGCTATCATCTTATGTCCTTCCCTCAGTCAGCACCTCATGGTGTAGTCTTCTTGCATGTGACTTATGTCCCTGCACAAGAAAAGAACTTCACAACTGCTCCTGCCATTTGTCATGATGGAAAAGCACACTTTCCTCGTGAAGGTGTCTTTGTTTCAAATGGCACACACTGGTTTGTAACACAAAGGAATTTTTATGAACCACAAATCATTACTACACACAACACATTTGTGTCTGGTAACTGTGATGTTGTAATAGGAATTGTCAACAACACAGTTTATGATCCTTTGCAACCTGAATTAGACTCATTCAAGGAGGAGTTAGATAAATATTTTAAGAATCATACATCACCAGATGTTGATTTAGGTGACATCTCTGGCATTAATGCTTCAGTTGTAAACATTCAAAAAGAAATTGACCGCCTCAATGAGGTTGCCAAGAATTTAAATGAATCTCTCATCGATCTCCAAGAACTTGGAAAGTATGAGCAGTATATAAAATGGCCATGGTACATTTGGCTAGGTTTTATAGCTGGCTTGATTGCCATAGTAATGGTGACAATTATGCTTTGCTGTATGACCAGTTGCTGTAGTTGTCTCAAGGGCTGTTGTTCTTGTGGATCCTGCTGCAAATTTGATGAAGACGACTCTGAGCTAGTGCTCAAAGGAGTCAAATTACATTACACATAA
**S-Orf8 gene**	**366**	ATGAAATTTCTTGTTTTCTTAGGAATCATCACAACTGTAGCTGCATTTCACCAAGAATGTAGTTTACAGTCATGTACTCAACATCAACCATATGTAGTTGATGACCCGTGTCCTATTCACTTCTATTCTAAATGGTATATTAGAGTAGGAGCTAGAAAATCAGCACCTTTAATTGAATTGTGCGTGGATGAGGCTGGTTCTAAATCACCCATTCAGTACATCGATATCGGTAATTATACAGTTTCCTGTTCACCTTTTACAATTAATTGCCAGGAACCTAAATTGGGTAGTCTTGTAGTGCGTTGTTCGTTCTATGAAGACTTTTTAGAGTATCATGACGTTCGTGTTGTTTTAGATTTCATCTAA

**L-Orf8 gene**	**366**	ATGAAATTTCTTGTTTTCTTAGGAATCATCACAACTGTAGCTGCATTTCACCAAGAATGTAGTTTACAGTCATGTACTCAACATC<br>AACCATATGTAGTTGATGACCCGTGTCCTATTCACTTCTATTCTAAATGGTATATTAGAGTAGGAGCTAGAAAATCAGCACCTTT<br>AATTGAATTGTGCGTGGATGAGGCTGGTTCTAAATCACCCATTCAGTACATCGATATCGGTAATTATACAGTTTCCTGTTTACCT<br>TTTACAATTAATTGCCAGGAACCTAAATTGGGTAGTCTTGTAGTGCGTTGTTCGTTCTATGAAGACTTTTTAGAGTATCATGACG<br>TTCGTGTTGTTTTAGATTTCATCTAA
**NP gene**	**1260**	ATGTCTGATAATGGACCCCAAAATCAGCGAAATGCACCCCGCATTACGTTTGGTGGACCCTCAGATTCAACTGGCAGTAACCAG<br>AATGGAGAACGCAGTGGGGCGCGATCAAAACAACGTCGGCCCCAAGGTTTACCCAATAATACTGCGTCTTGGTTCACCGCTCTC<br>ACTCAACATGGCAAGGAAGACCTTAAATTCCCTCGAGGACAAGGCGTTCCAATTAACACCAATAGCAGTCCAGATGACCAAAT<br>TGGCTACTACCGAAGAGCTACCAGACGAATTCGTGGTGGTGACGGTAAAATGAAAGATCTCAGTCCAAGATGGTATTTCTACTA<br>CCTAGGAACTGGGCCAGAAGCTGGACTTCCCTATGGTGCTAACAAAGACGGCATCATATGGGTTGCAACTGAGGGAGCCTTGA<br>ATACACCAAAAGATCACATTGGCACCCGCAATCCTGCTAACAATGCTGCAATCGTGCTACAACTTCCTCAAGGAACAACATTGC<br>CAAAAGGCTTCTACGCAGAAGGGAGCAGAGGCGGCAGTCAAGCCTCTTCTCGTTCCTCATCACGTAGTCGCAACAGTTCAAGA<br>AATTCAACTCCAGGCAGCAGTAGGGGAACTTCTCCTGCTAGAATGGCTGGCAATGGCGGTGATGCTGCTCTTGCTTTGCTGCTG<br>CTTGACAGATTGAACCAGCTTGAGAGCAAAATGTCTGGTAAAGGCCAACAACAACAAGGCCAAACTGTCACTAAGAAATCTGC<br>TGCTGAGGCTTCTAAGAAGCCTCGGCAAAAACGTACTGCCACTAAAGCATACAATGTAACACAAGCTTTCGGCAGACGTGGTC<br>CAGAACAAACCCAAGGAAATTTTGGGGACCAGGAACTAATCAGACAAGGAACTGATTACAAACATTGGCCGCAAATTGCACA<br>ATTTGCCCCCAGCGCTTCAGCGTTCTTCGGAATGTCGCGCATTGGCATGGAAGTCACACCTTCGGGAACGTGGTTGACCTACAC<br>AGGTGCCATCAAATTGGATGACAAAGATCCAAATTTCAAAGATCAAGTCATTTTGCTGAATAAGCATATTGACGCATACAAAA<br>CATTCCCACCAACAGAGCCTAAAAAGGACAAAAAGAAGAAGGCTGATGAAACTCAAGCCTTACCGCAGAGACAGAAGAAACA<br>GCAAACTGTGACTCTTCTTCCTGCTGCAGATTTGGATGATTTCTCCAAACAATTGCAACAATCCATGAGCAGTGCTGACTCAACT<br>CAGGCCTAA

Note: NP gene, the gene encoding the nucleoprotein of SARS-CoV-2 (nt 28,274-29,533, NCBI accession number MN908947); Wild-type S gene, gene fragment of SARS-CoV-2 spike protein (nt 21,563-25,384, NCBI accession number MN908947); Mutant S gene, gene fragments of SARS-CoV-2 spike protein including mutations: L5F, D80A, D215G, R246I, K417N, L452R/Q, Y453F, E484Q, N501Y, A570D, D614G, P681H, A701V, T716I, S982A, D1118H, P1263L; S-Orf8 gene, S lineage gene fragment of open reading frame 8 (ORF8, nt 27,894-28,259, NCBI accession number MN908947); L-Orf8 gene, L lineage fragment of SARS-CoV-2 ORF8 with mutation of S84L.

Supplementary Table S3. Information of clinical samples

SARS-CoV-2 variants	Sample ID	Cycle threshold value (ORF1a/b)	Cycle threshold value (N)
Wild-type	20SF5770	37	36
	20SF5783	28	28
	20SF5774	21	21
	20SF5787	21	22
	20SF5788	28	28
Aplha	XG01774	21	20
	XG02064	24	23
	XG03594	17	16
	XG03595	26	26
	XG03596	15	15
	XG03607	18	16
	XG03623	16	15
	XG04704	18	17
	XG04850	21	22
	XG08145	24	22
	XG08337	17	16
Beta	XG02289	23	23
	XG03246	18	18
	XG03255	19	19
	XG03240	14	13
	XG05824	18	17
	XG07753	27	26
	XG07933	22	22

	XG08741	31	31
**Delta**	XG08547	20	19
	XG08859	18	19
	XG09082	16	17
	XG08688	21	21
	XG08689	15	15
	XG08692	16	16
	XG08693	16	16
	XG08694	18	16

Supplementary Table S4. Summary of guide RNA (crRNA) sequences

crRNA	Mutations detected	On-target	Off-target	Sequence (5'→3')
crRNA-N1	NA	NP gene	NA	UAAUUUCUACUAAGUGUAGACCCCCAGCGCUUCAGCGUUC
crRNA-N2	NA	NP gene	NA	UAAUUUCUACUAAGUGUAGAGUGGACCCUCAGAUUCAACU
crRNA-N3	NA	NP gene	NA	UAAUUUCUACUAAGUGUAGACCCAAUAAUACUGCGUCUUG
crRNA-N4	NA	NP gene	NA	UAAUUUCUACUAAGUGUAGAUACUACCUAGGAACUGGGCC
crRNA-N5	NA	NP gene	NA	UAAUUUCUACUAAGUGUAGACUGCUGCUUGACAGAUUGAA
crRNA-S	NA	Both wild-type and mutant S gene	NA	UAAUUUCUACUAAGUGUAGAAUAGGGGCUGAACAUGUCAA
crRNA-S-5F	L5F (Spike protein)	Mutant S gene (5F)	Wild-type S gene (5l)	UAAUUUCUACUAAGUGUAGAUUUUAUUGCCACUAGUCUCU
crRNA-S-80A	D80A (Spike protein)	Mutant S gene (80A)	Wild-type S gene (80D)	UAAUUUCUACUAAGUGUAGACUAACCCUGUCCUACCAUUU
crRNA-S-215G	D215G (Spike protein)	Mutant S gene (215G)	Wild-type S gene (215D)	UAAUUUCUACUAAGUGUAGAGUGCGUGGUCUCCCUCAGGG
crRNA-S-246I	R246I (Spike protein)	Mutant S gene (246I)	Wild-type S gene (246R)	UAAUUUCUACUAAGUGUAGACAUAUAAGUUAUUUGACUCC
crRNA-S-417K	K417N (Spike protein)	Wild-type S gene (417K)	Mutant S gene (417N)	UAAUUUCUACUAAGUGUAGAAACGAUUGCUGAUUAUAAUU
crRNA-S-417N	K417N (Spike protein)	Mutant S gene (417N)	Wild-type S gene (417K)	UAAUUUCUACUAAGUGUAGAAACUAUUGCUGAUUAUAAUU
crRNA-S-452R	L452R (Spike protein)	Mutant S gene (452R)	Wild-type S gene (452L)	UAAUUUCUACUAAGUGUAGACCGAUAUAGAUUGUUUAGGA
crRNA-S-452Q	L452Q (Spike protein)	Mutant S gene (452Q)	Wild-type S gene (452L)	UAAUUUCUACUAAGUGUAGACCAAUAUAGAUUGUUUAGGA

crRNA-S-453F	Y453F (Spike protein)	Mutant S gene (453F)	Wild-type S gene (453Y)	UAAUUUCUACUAAGUGUAGAGAUUGUUUAGGAAGUCUAAU
crRNA-S-478T	T478K (Spike protein)	Wild-type S gene (478T)	Mutant S gene (478K)	UAAUUUCUACUAAGUGUAGAAAGGGGUGCUACCGGCCUGA
crRNA-S-478K	T478K (Spike protein)	Mutant S gene (478K)	Wild-type S gene (478T)	UAAUUUCUACUAAGUGUAGAAAGGGUUGCUACCGGCCUGA
crRNA-S-484K	E484K (Spike protein)	Mutant S gene (484K)	Wild-type S gene (484E)	UAAUUUCUACUAAGUGUAGAACACCAUUACAAGGUGUGCU
crRNA-S-484Q	E484Q (Spike protein)	Mutant S gene (484Q)	Wild-type S gene (484E)	UAAUUUCUACUAAGUGUAGAAAUGGUGUACAAGGUUUUAA
crRNA-S-501N	N501Y (Spike protein)	Wild-type S gene (501N)	Mutant S gene (501Y)	UAAUUUCUACUAAGUGUAGACAACCCACUAAUGGUGUUGG
crRNA-S-570D	A570D (Spike protein)	Mutant S gene (570D)	Wild-type S gene (570A)	UAAUUUCUACUAAGUGUAGAGCAGAGACAUUGAUGACACU
crRNA-S-614D	D614G (Spike protein)	Wild-type S gene (614D)	Mutant S gene (614G)	UAAUUUCUACUAAGUGUAGAUCAGGACGUUAACUGCACAG
crRNA-S-701V	A701V (Spike protein)	Mutant S gene (701V)	Wild-type S gene (701A)	UAAUUUCUACUAAGUGUAGAUACACCAAGUGACAUAGUGU
crRNA-S-716I	T716I (Spike protein)	Mutant S gene (716I)	Wild-type S gene (716T)	UAAUUUCUACUAAGUGUAGAAUGGGUAUGGCAAUAGAGUU
crRNA-S-982S	S982A (Spike protein)	Wild-type S gene (982S)	Mutant S gene (982A)	UAAUUUCUACUAAGUGUAGAACGUCUUGACAAAGUUGAGG
crRNA-S-1263L	P1263L (Spike protein)	Mutant S gene (1263L)	Wild-type S gene (1263P)	UAAUUUCUACUAAGUGUAGAAGCACUAGCUCAGAGUCGUC
crRNA-Orf8	S84L (ORF8 protein)	L-Orf8 gene (84L)	S-Orf8 gene (84S)	UAAUUUCUACUAAGUGUAGACCUUUUACAAUUAAUUGCCA

Note: NA, not available; NP gene, the gene encoding the nucleoprotein of SARS-CoV-2 (nt 28,274-29,533, NCBI accession number MN908947); Wild-type S gene, gene fragment of SARS-CoV-2 spike protein (nt 21,563-25,384, NCBI accession number MN908947); Mutant S gene, gene fragments of SARS-CoV-2 spike protein including mutations: L5F, D80A, D215G, R246I, K417N, L452R/Q, Y453F, T478K, E484K/Q, N501Y, A570D, D614G, P681H, A701V, T716I, S982A, D1118H, P1263L; S-Orf8 gene, S lineage gene fragment of open reading frame 8 (ORF8, nt 27,894-28,259, NCBI accession number MN908947); L-Orf8 gene, L lineage fragment of SARS-CoV-2 ORF8 with mutation of S84L.

Supplementary Table S5. Summary of primer sequences

**Primer**	**Sequence (5'→3')**
N-gene_F	ATGTCTGATAATGGACC
N-gene_R	GGTGTGACTTCCATGCC
S-gene_F1	CTAGTGATGTTCTTGTT
S-gene_R1	TGCACAGTCTACAGCATC
S-gene_F2	ACTTGTGCCCTTTTGGTGAAG
S-gene_R2	GCTATTCCAGTTAAAGCACGGT
S-gene_F3	AGACTCACTTTCTTCCACAGCAA
S-gene_R3	GTATCGTTGCAGTAGCGCGA
Orf8-gene_F	AGGAATCATCACAACTGTAGC
Orf8-gene_R	AACGAACAACGCACTACAAGAC
K417N_F	ATCGCTCCAGGGCAAATTTGAAA
K417N_R	GCTATTCCAGTTAAAGCACGGT
L452R_F	AGGTTGGTGGTAATTATATTTAC
L452R_R	AGTACTACTACTCTGTATGGTTGGT
T478K-R	AACACCATTTCAAGGT
E484Q_F	AGCACACCTTTTAATGGTGTT
E484Q_R	AGTACTACTACTCTGTATGGTTGGT
HCoV 229E NP gene_F	CTACAGTCAAATGGGCTGATGCATCT
HCoV 229E NP gene_R	TGACTTCATCAATTATGTCAG
HCoV OC43 NP gene_F	TCCAGTAGTAGAGCGTCCTCTGGA
HCoV OC43 NP gene_R	GCTGATGTCCTCTGCAGTCAACTCTA
HCoV HKU1 NP gene_F	ATACTCCCGGTCATTATGCTGGAA
HCoV HKU1 NP gene_R	ACATAAGGATCATCAAGAGTAGC

HCoV 229E S gene outer_F	TTGTTGCATATGCCTTGTTGCATATTGC
HCoV 229E S gene outer__R	ATCTTTTCAACGTCGTAATAAGGAAGTTTAG
HCoV 229E S gene inner_F	ACTCTGTTTGCAACGGCTGTGTTGGT
HCoV 229E S gene inner__R	ACTCACCACAAAGATGAGCACGACTG
HCoV OC43 S gene outer_F	TATAAGTATTGATACTGTTGATGTTACTA
HCoV OC43 S gene outer__R	GTCCTTGAGATTGATGTAGCTATGA
HCoV OC43 S gene inner_F	CAGGTTCCACATATCGTAATAT
HCoV OC43 S gene inner__R	AGCTATACCTCTATTACCAGCAATGCA
HCoV HKU1 S gene outer_F	GCCTACAACATTAGCTGTTATA
HCoV HKU1 S gene outer__R	ACACACTCATTAACCTTCTC
HCoV HKU1 S gene inner_F	GTTAAGAATACTAAGTTGTATGT
HCoV HKU1 S gene inner__R	AGCCTATCAATCTGAACCTGA
HCoV 229E Orf3 gene_F	CTCTAGGTTTGTTCACATTGCA
HCoV 229E Orf3 gene_R	TCTGTGTTGACTATTCCAACAGC
HCoV OC43 Orf5 gene_F	ACTTCACATGACGACTAAGTT
HCoV OC43 Orf5 gene_R	ACAATCCACCATCACATGA
HCoV HKU1 Orf4 gene_F	ATGGACGTTTGGAGACCTAGC
HCoV HKU1 Orf4 gene_R	TCAACCATACTTATTAAGTGC

Supplementary Table S6. Comparison of CRISPR-Cas12a, RT-PCR and whole genome sequencing in detecting SARS-CoV-2 variants

Characteristics	CRISPR-Cas12a	RT-PCR(1, 2)	Whole genome sequencing§
**Procedure**	1. Amplification plus Cas12 cleavage (1.0-2h)<br>2. Amplification-free Cas12 system (20-30 min)	1. Amplification of target gene (1.5-2h)<br>2. Detection (0-30min) depending on different assays	1. Library preparations (5-7 h)<br>2. Sequencing (17-32 h)<br>3. Bioinformatic analysis (1-2 h)
**Assay results**	Qualitative/Semi-quantitative / diagnosis / genotyping	Quantitative / diagnosis / genotyping	Qualitative / genotyping / characterizing mutations
**Limits of detection**	1-10 copies per µL or higher	~1 copies per µL	Not applicable
**Estimated cost**	$4 per sample	$3 per sample	$87 per sample
**Benefits**	1. Isothermal amplification and reaction at room temperature.<br>2. Easily to find conserved primer sequences to ensure sensitivity.<br>3. Specificity relies on crRNA or gRNA.	1. Matured technology and products.<br>2. PCR equipment and facilities available.	1. Identify novel mutations and variants.<br>2. Provide complete genome sequence information.
**Challenges**	1. Limited to known mutations or variants with signature mutations.<br>2. Multiple readouts and mixed mutations need an integrated testing system and algorithm.	1. PCR equipment and facility needed;<br>2. Limited to known mutations or variants with signature mutations.<br>3. Difficult to find appropriate primers for specific mutations.<br>4. Not suitable for point-of-care testing.	1. Time-consuming for sequencing.<br>2. Complex bioinformatics pipeline.<br>3. High-end facilities and trained specialists.

1. **Wang H, Miller JA, Verghese M, Sibai M, Solis D, Mfuh KO, Jiang B, Iwai N, Mar M, Huang C, Yamamoto F, Sahoo MK, Zehnder J, Pinsky BA.** 2021. Multiplex SARS-CoV-2 Genotyping RT-PCR for Population-Level Variant Screening and Epidemiologic Surveillance. Journal of clinical microbiology**:**Jcm0085921.
2. **Zelyas N, Pabbaraju K, Croxen MA, Lynch T, Buss E, Murphy SA, Shokoples S, Wong A, Kanji JN, Tipples G.** 2021. Precision Response to the Rise of the SARS-CoV-2 B.1.1.7 Variant of Concern by Combining Novel PCR Assays and Genome Sequencing for Rapid Variant Detection and Surveillance. Microbiology spectrum **9:**e0031521.

§Based on Illumina MiSeq sequencing platforms.

```
                                1        10        20        30        40        50        60
MN908947/Wuhan-Hu-1             MFVFLVLLPLVSSQCVNLTTRTQLPPAYTNSFTRGVYYPDKVFRSSVLHSTQDLFLPFFS
BS001137/B.1.1.7/Alpha          MFVFLVLLPLVSSQCVNLTTRTQLPPAYTNSFTRGVYYPDKVFRSSVLHSTQDLFLPFFS
BS001138/B.1.1.7/Alpha          MFVFLVLLPLVSSQCVNLTTRTQLPPAYTNSFTRGVYYPDKVFRSSVLHSTQDLFLPFFS
BS001139/B.1.1.7/Alpha          MFVFLVLLPLVSSQCVNLTTRTQLPPAYTNSFTRGVYYPDKVFRSSVLHSTQDLFLPFFS
BS001140/B.1.1.7/Alpha          MFVFLVLLPLVSSQCVNLTTRTQLPPAYTNSFTRGVYYPDKVFRSSVLHSTQDLFLPFFS
MW913362/B.1.1.7/Alpha          MFVFLVLLPLVSSQCVNLTTRTQLPPAYTNSFTRGVYYPDKVFRSSVLHSTQDLFLPFFS
MZ266365/B.1.1.7/Alpha          MFVFLVLLPLVSSQCVNLTTRTQLPPAYTNSFTRGVYYPDKVFRSSVLHSTQDLFLPFFS
MZ350105/B.1.1.7/Alpha          MFVFLVLLPLVSSQCVNLTTRTQLPPAYTNSFTRGV.YPDKVFRSSVLHSTQDLFLPFFS
MZ350109/B.1.1.7/Alpha          MFVFLVLLPLVSSQCVNLTTRTQLPPAYTNSFTRGVYYPDKVFRSSVLHSTQDLFLPFFS
MZ888516/B.1.1.7/Alpha          MFVFLVLLPLVSSQCVNLTTRTQLPPAYTNSFTRGVYYPDKVFRSSVLHSTQDLFLPFFS
OU562607/B.1.1.7/Alpha          MFVFLVLLPLVSSQCVNLTTRTQLPPAYTNSFTRGVYYPDKVFRSSVLHSTQDLFLPFFS
OU514166/B.1.1.7/Alpha          MFVFLVLLPLVSSQCVNLTTRTQLPPAYTNSFTRGVYYPDKVFRSSVLHSTQDLFLPFFS
OU538851/B.1.1.7/Alpha          MFVFLVLLPLVSSQCVNLTTRTQLPPAYTNSFTRGVYYPDKVFRSSVLHSTQDLFLPFFS
OU538849/B.1.1.7/Alpha          MFVFLVLLPLVSSQCVNLTTRTQLPPAYTNSFTRGVYYPDKVFRSSVLHSTQDLFLPFFS
MZ895877/B.1.1.7/Alpha          MFVFLVLLPLVSSQCVNLTTRTQLPPAYTNSFTRGVYYPDKVFRSSVLHSTQDLFLPFFS
OU514176/B.1.1.7/Alpha          MFVFLVLLPLVSSQCVNLTTRTQLPPAYTNSFTRGVYYPDKVFRSSVLHSTQDLFLPFFS
OU562608/B.1.1.7/Alpha          MFVFLVLLPLVSSQCVNLTTRTQLPPAYTNSFTRGVYYPDKVFRSSVLHSTQDLFLPFFS
MW981442/B.1.351/Beta           MFVFLVLLPLVSSQCVNFTTRTQLPPAYTNSFTRGVYYPDKVFRSSVLHSTQDLFLPFFS
MZ298839/B.1.351/Beta           MFVFLVLLPLVSSQCVNLTTRTQLPPSYTNSFTRGVYYPDKVFRSSVLHSTQDLFLPFFS
MZ913100/B.1.351/Beta           MFVFLVLLPLVSSQCVNFTTRTQLPPAYTNSFTRGVYYPDKVFRSSVLHSTQDLFLPFFS
MZ879365/B.1.351/Beta           MFVFLVLLPLVSSQCVNFTTRTQLLPAYTNSFTRGVYYPDKVFRSSVLHSTQDLFLPFFS
OU525106/B.1.351/Beta           MFVFLVLLPLVSSQCVNLTTRTQLPPSYTNSFTRGVYYPDKVFRSSVLHSTQDLFLPFFS
OU539352/B.1.351/Beta           MFVFLVLLPLVSSQCVNLTTRTQLPPAYTNSFTRGVYYPDKVFRSSVLHSTQDLFLPFFS
MZ202314/B.1.351/Beta           MFVFLVLLPLVSSQCVNFTTRTQLPPAYTNSFTRGVYYPDKVFRSSVLHSTQDLFLPFFS
MZ298840/B.1.351/Beta           MFVFLVLLPLVSSQCVNLTTRTQLPPSYTNSFTRGVYYPDKVFRSSVLHSTQDLFLPFFS
MZ376663/B.1.351/Beta           MFVFLVLLPLVSSQCVNFTTRTQLPPAYTNSFTRGVYYPDKVFRSSVLHSTQDLFLPFFS
LC643036/B.1.617.2/Delta        MFVFLVLLPLVSSQCVNLRTRTQLPPAYTNSFTRGVYYPDKVFRSSVLHSTQDLFLPFFS
LC643044/B.1.617.2/Delta        MFVFLVLLPLVSSQCVNLRTRTQLPPAYTNSFTRGVYYPDKVFRSSVLHSTQDLFLPFFS
MZ853946/B.1.617.2/Delta        MFVFLVLLPLVSSQCVNLRTRTQLPPAYTNSFTRGVYYPDKVFRSSVLHSTQDLFLPFFS
MZ854386/B.1.617.2/Delta        MFVFLVLLPLVSSQCVNLRTRTQLPPAYTNSFTRGVYYPDKVFRSSVLHSTQDLFLPFFS
MZ888532/B.1.617.2/Delta        MFVFLVLLPLVSSQCVNLRTRTQLPPAYTNSFTRGVYYPDKVFRSSVLHSTQDLFLPFFS
MZ888533/B.1.617.2/Delta        MFVFLVLLPLVSSQCVNLRTRTQLPPAYTNSFTRGVYYPDKVFRSSVLHSTQDLFLPFFS
MZ896136/B.1.617.2/Delta        MFVFLVLLPLVSSQCVNLRTRTQLPPAYTNSFTRGVYYPDKVFRSSVLHSTQDLFLPFFS
OU539261/B.1.617.2/Delta        MFVFLVLLPLVSSQCVNLRTRTQLPPAYTNSFTRGVYYPDKVFRSSVLHSTQDLFLPFFS
MZ854389/B.1.617.2/Delta        MFVFLVLLPLVSSQCVNLRTRTQLPPAYTNSFTRGVYYPDKVFRSSVLHSTQDLFLPFFS
OU562629/B.1.617.2/Delta        MFVFLVLLPLVSSQCVNLRTRTQLPPAYTNSFTRGVYYPDKVFRSSVLHSTQDLFLPFFS
OU539690/B.1.617.2/Delta        MFVFLVLLPLVSSQCVNLRTRTQLPPAYTNSFTRGVYYPDKVFRSSVLHSTQDLFLPFFS
MZ854387/B.1.617.2/Delta        MFVFLVLLPLVSSQCVNLRTRTQLPPAYTNSFTRGVYYPDKVFRSSVLHSTQDLFLPFFS
OU539784/P.1/Gamma              MFVFLVLLPLVSSQCVNFTNRTQLPSAYTNSFTRGVYYPDKVFRSSVLHSTQDLFLPFFS
OU539830/P.1/Gamma              MFVFLVLLPLVSSQCVNFTNRTQLPSAYTNSFTRGVYYPDKVFRSSVLHSTQDLFLPFFS
OU539894/P.1/Gamma              MFVFLVLLPLVSSQCVNFTNRTQLPSAYTNSFTRGVYYPDKVFRSSVLHSTQDLFLPFFS
MZ477748/P.1/Gamma              MFVFLVLLPLVSSQCVNFTNRTQLPSAYTNSFTRGVYYPDKVFRSSVLHSTQDLFLPFFS
MZ477746/P.1/Gamma              MFVFLVLLPLVSSQCVNFTNRTQLPSAYTNSFTRGVYYPDKVFRSSVLHSTQDLFLPFFS
MW938104/P.1/Gamma              MFVFLVLLPLVSSQCVNFTNRTQLPSAYTNSFTRGVYYPDKVFRSSVLHSTQDLFLPFFS
MZ896179/P.1/Gamma              MFVFLVLLPLVSSQCVNFTNRTQLPSAYTNSFTRGVYYPDKVFRSSVLHSTQDLFLPFFS
MZ896359/P.1/Gamma              MFVFLVLLPLVSSQCVNFTNRTQLPSAYTNSFTRGVYYPDKVFRSSVLHSTQDLFLPFFS
MZ896669/P.1/Gamma              MFVFLVLLPLVSSQCVNFTNRTQLPSAYTNSFTRGVYYPDKVFRSSVLHSTQDLFLPFFS
OU565182/P.1/Gamma              MFVFLVLLPLVSSQCVNFTNRTQLPSAYTNSFTRGVYYPDKVFRSSVLHSTQDLFLPFFS
OU492255/P.1/Gamma              MFVFLVLLPLVSSQCVNFTNRTQLPSAYTNSFTRGVYYPDKVFRSSVLHSTQDLFLPFFS
MZ911221/B.1.427/Epsilon        MFVFLVLLPLVSIQCVNLTTRTQLPPAYTNSFTRGVYYPDKVFRSSVLHSTQDLFLPFFS
MZ375854/B.1.427/Epsilon        MFVFLVLLPLVSIQCVNLTTRTQLPPAYTNSFTRGVYYPDKVFRSSVLHSTQDLFLPFFS
MZ375858/B.1.427/Epsilon        MFVFLVLLPLVSIQCVNLTTRTQLPPAYTNSFTRGVYYPDKVFRSSVLHSTQDLFLPFFS
MZ943834/B.1.429/Epsilon        MFVFLVLLPLVSIQCVNLTTRTQLPPAYTNSFTRGVYYPDKVFRSSVLHSTQDLFLPFFS
MZ277390/B.1.429/Epsilon        MFVFLVLLPLVSIQCVNLTTRTQLPSAYTNSFTRGVYYPDKVFRSSVLHSTQDLFLPFFS
OU282717/B.1.429/Epsilon        MFVFLVLLPLVSIQCVNLTTRTQLPPAYTNSFTRGVYYPDKVFRSSVLHSTQDLFLPFFS
OU235436/B.1.429/Epsilon        MFVFLVLLPLVSIQCVNLTTRTQLPPAYTNSFTRGVYYPDKVFRSSVLHSTQDLFLPFFS
MZ687447/B.1.429/Epsilon        MFVFLVLLPLVSIQCVNLTTRTQLPPAYTNSFTRGVYYPDKVFRSSVLHSTQDLFLPFFS
MZ538882/B.1.617.1/Kappa        MFVFLVLLPLVSSQCVNLTTRTQLPPAYTNSFTRGVYYPDKVFRSSVLHSTQDLFLPFFS
MZ724425/B.1.617.1/Kappa        MFVFLVLLPLVSSQCVNLTTRTQLPPAYTNSFTRGVYYPDKVFRSSVLHSTQDLFLPFFS
MZ363839/B.1.617.1/Kappa        MFVFLVLLPLVSSQCVNLTTRTQLPPAYTNSFTRGVYYPDKVFRSSVLHSTQDLFLPFFS
MZ332524/B.1.617.1/Kappa        MFVFLVLLPLVSSQCVNLTTRTQLPPAYTNSFTRGVYYPDKVFRSSVLHSTQDLFLPFFS
MZ332527/B.1.617.1/Kappa        MFVFLVLLPLVSSQCVNLTTRTQLPPAYTNSFTRGVYYPDKVFRSSVLHSTQDLFLPFFS
LC633761/B.1.617.1/Kappa        MFVFLVLLPLVSSQCVNLTTRTQLPPAYTNSFTRGVYYPDKVFRSSVLHSTQDLFLPFFS
MZ562746/B.1.617.1/Kappa        MFVFLVLLPLVSSQCVNLTTRTQLPPAYTNSFTRGVYYPDKVFRSSVLHSTQDLFLPFFS
MZ571142/B.1.617.1/Kappa        MFVFLVLLPLVSSQCVNLTTRTQLPPAYTNSFTRGVYYPDKVFRSSVLHSTQDLFLPFFS
OU168413/B.1.617.1/Kappa        MFVFLVLLPLVSSQCVNLTTRTQLPPAYTNSFTRGVYYPDKVFRSSVLHSTQDLFLPFFS
MZ026886/B.1.617.1/Kappa        MFVFLVLLPLVSSQCVNLTTRTQLPPAYTNSFTRGVYYPDKVFRSSVLHSTQDLFLPFFS
OU172184/B.1.617.1/Kappa        MFVFLVLLPLVSSQCVNLTTRTQLPPAYTNSFTRGVYYPDKVFRSSVLHSTQDLFLPFFS
MZ397173/B.1.617.1/Kappa        MFVFLVLLPLVSSQCVNLTTRTQLPPAYTNSFTRGVYYPDKVFRSSVLHSTQDLFLPFFS
MZ397176/B.1.617.1/Kappa        MFVFLVLLPLVSSQCVNLTTRTQLPPAYTNSFTRGVYYPDKVFRSSVLHSTQDLFLPFFS
MZ397175/B.1.617.1/Kappa        MFVFLVLLPLVSSQCVNLTTRTQLPPAYTNSFTRGVYYPDKVFRSSVLHSTQDLFLPFFS
MZ397177/B.1.617.1/Kappa        MFVFLVLLPLVSSQCVNLTTRTQLPPAYTNSFTRGVYYPDKVFRSSVLHSTQDLFLPFFS
OU290035/B.1.617.1/Kappa        MFVFLVLLPLVSSQCVNLTTRTQLPPAYTNSFTRGVYYPDKVFRSSVLHSTQDLFLPFFS
OU265178/B.1.617.1/Kappa        MFVFLVLLPLVSSQCVNLTTRTQLPPAYTNSFTRGVYYPDKVFRSSVLHSTQDLFLPFFS
OU254949/B.1.617.1/Kappa        MFVFLVLLPLVSSQCVNLTTRTQLPPAYTNSFTRGVYYPDKVFRSSVLHSTQDLFLPFFS
OU247018/B.1.617.1/Kappa        MFVFLVLLPLVSSQCVNLTTRTQLPPAYTNSFTRGVYYPDKVFRSSVLHSTQDLFLPFFS
OU061269/B.1.617.1/Kappa        MFVFLVLLPLVSSQCVNLTTRTQLPPAYTNSFTRGVYYPDKVFRSSVLHSTQDLFLPFFS
OU182966/B.1.617.1/Kappa        MFVFLVLLPLVSSQCVNLTTRTQLPPAYTNSFTRGVYYPDKVFRSSVLHSTQDLFLPFFS
MZ746235/B.1.617.1/Kappa        MFVFLVLLPLVSSQCVNLTTRTQLPPAYTNSFTRGVYYPDKVFRSSVLHSTQDLFLPFFS
MZ487800/B.1.617.1/Kappa        MFVFLVLLPLVSSQCVNLTTRTQLPPAYTNSFTRGVYYPDKVFRSSVLHSTQDLFLPFFS
OU322235/B.1.617.1/Kappa        MFVFLVLLPLVSSQCVNLTTRTQLPPAYTNSFTRGVYYPDKVFRSSVLHSTQDLFLPFFS
MZ953185/C.37/Lambda            MFVFLVLLPLVSSQCVNLTTRTQLPPAYTNSFTRGVYYPDKVFRSSVLHSTQDLFLPFFS
MZ937666/C.37/Lambda            MFVFLVLLPLVSSQCVNLTTRTQLPPAYTNSFTRGVYYPDKVFRSSVLHSTQDLFLPFFS
OU356036/C.37/Lambda            MFVFLVLLPLVSSQCVNLTTRTQLPPAYTNSFTRGVYYPDKVFRSSVLHSTQDLFLPFFS
OU351967/C.37/Lambda            MFVFLVLLPLVSSQCVNLTTRIQLPPAYTNSFTRGVYYPDKVFRSSVLHSTQDLFLPFFS
OU053018/C.37/Lambda            MFVFLVLLPLVSSQCVNLTTRTQLPPAYTNSFTRGVYYPDKVFRSSVLHSTQDLFLPFFS
OU268528/C.37/Lambda            MFVFLVLLPLVSSQCVNLTTRTQLPPAYTNSFTRGVYYPDKVFRSSVLHSTQDLFLPFFS
OU100999/C.37/Lambda            MFVFLVLLPLVSSQCVNLTTRTQLPPAYTNSFTRGVYYPDKVFRSSVLHSTQDLFLPFFS
MZ275295/C.37/Lambda            MFVFLVLLPLVSSQCVNLTTRTQLPPAYTNSFTRGVYYPDKVFRSSVLHSTQDLFLPFFS
MZ275301/C.37/Lambda            MFVFLVLLPLVSSQCVNLTTRTQLPPAYTNSFTRGVYYPDKVFRSSVLHSTQDLFLPFFS
MZ908711/C.37/Lambda            MFVFLVLLPLVSSQCVNLTTRTQLPPAYTNSFTRGVYYPDKVFRSSVLHSTQDLFLPFFS
MZ496613/C.37/Lambda            MFVFLVLLPLVSSQCVNLTTRTQLPPAYTNSFTRGVYYPDKVFRSSVLHSTQDLFLPFFS
OU517038/C.37/Lambda            MFVFLVLLPLVSSQCVNLTTRTQLPPAYTNSFTRGVYYPDKVFRSSVLHSTQDLFLPFFS
MZ953277/C.37/Lambda            MFVFLVLLPLVSSQCVNLTTRTQLPPAYTNSFTRGVYYPDKVFRSSVLHSTQDLFLPFFS
MZ942542/C.37/Lambda            MFVFLVLLPLVSSQCVNLTTRTQLPPAYTNSFTRGVYYPDKVFRSSVLHSTQDLFLPFFS
MZ857990/C.37/Lambda            MFVFLVLLPLVSSQCVNLTTRTQLPPAYTNSFTRGVYYPDKVFRSSVLHSTQDLFLPFFS
```

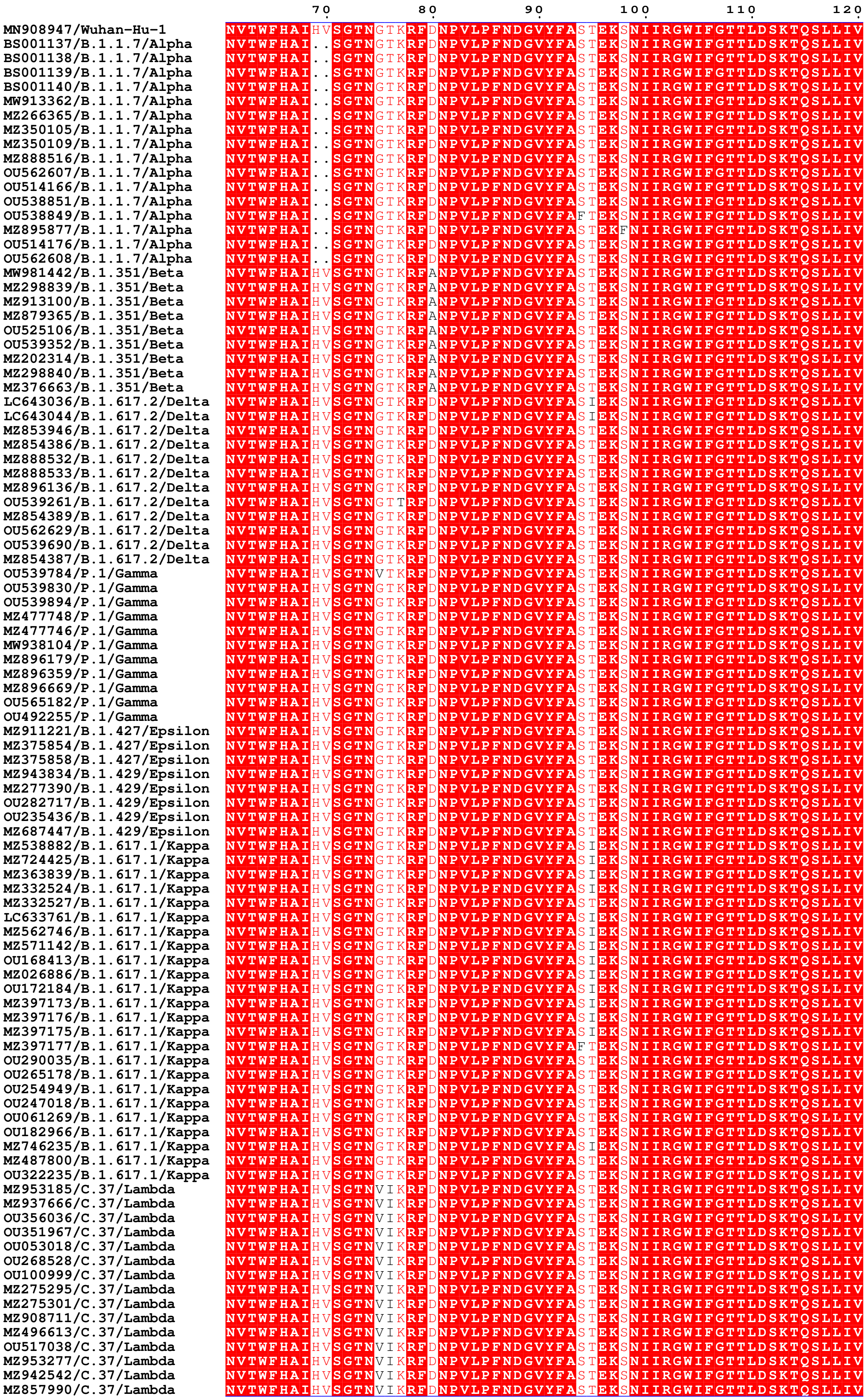

70 80 90 100 110 120
MN908947/Wuhan-Hu-1 NVTWFHAIHVSGTNGTKRFDNPVLPFNDGVYFASTEKSNIIRGWIFGTTLDSKTQSLLIV
BS001137/B.1.1.7/Alpha NVTWFHAI..SGTNGTKRFDNPVLPFNDGVYFASTEKSNIIRGWIFGTTLDSKTQSLLIV
BS001138/B.1.1.7/Alpha NVTWFHAI..SGTNGTKRFDNPVLPFNDGVYFASTEKSNIIRGWIFGTTLDSKTQSLLIV
BS001139/B.1.1.7/Alpha NVTWFHAI..SGTNGTKRFDNPVLPFNDGVYFASTEKSNIIRGWIFGTTLDSKTQSLLIV
BS001140/B.1.1.7/Alpha NVTWFHAI..SGTNGTKRFDNPVLPFNDGVYFASTEKSNIIRGWIFGTTLDSKTQSLLIV
MW913362/B.1.1.7/Alpha NVTWFHAI..SGTNGTKRFDNPVLPFNDGVYFASTEKSNIIRGWIFGTTLDSKTQSLLIV
MZ266365/B.1.1.7/Alpha NVTWFHAI..SGTNGTKRFDNPVLPFNDGVYFASTEKSNIIRGWIFGTTLDSKTQSLLIV
MZ350105/B.1.1.7/Alpha NVTWFHAI..SGTNGTKRFDNPVLPFNDGVYFASTEKSNIIRGWIFGTTLDSKTQSLLIV
MZ350109/B.1.1.7/Alpha NVTWFHAI..SGTNGTKRFDNPVLPFNDGVYFASTEKSNIIRGWIFGTTLDSKTQSLLIV
MZ888516/B.1.1.7/Alpha NVTWFHAI..SGTNGTKRFDNPVLPFNDGVYFASTEKSNIIRGWIFGTTLDSKTQSLLIV
OU562607/B.1.1.7/Alpha NVTWFHAI..SGTNGTKRFDNPVLPFNDGVYFASTEKSNIIRGWIFGTTLDSKTQSLLIV
OU514166/B.1.1.7/Alpha NVTWFHAI..SGTNGTKRFDNPVLPFNDGVYFASTEKSNIIRGWIFGTTLDSKTQSLLIV
OU538851/B.1.1.7/Alpha NVTWFHAI..SGTNGTKRFDNPVLPFNDGVYFASTEKSNIIRGWIFGTTLDSKTQSLLIV
OU538849/B.1.1.7/Alpha NVTWFHAI..SGTNGTKRFDNPVLPFNDGVYFAFTEKSNIIRGWIFGTTLDSKTQSLLIV
MZ895877/B.1.1.7/Alpha NVTWFHAI..SGTNGTKRFDNPVLPFNDGVYFASTEKFNIIRGWIFGTTLDSKTQSLLIV
OU514176/B.1.1.7/Alpha NVTWFHAI..SGTNGTKRFDNPVLPFNDGVYFASTEKSNIIRGWIFGTTLDSKTQSLLIV
OU562608/B.1.1.7/Alpha NVTWFHAI..SGTNGTKRFDNPVLPFNDGVYFASTEKSNIIRGWIFGTTLDSKTQSLLIV
MW981442/B.1.351/Beta NVTWFHAIHVSGTNGTKRFANPVLPFNDGVYFASTEKSNIIRGWIFGTTLDSKTQSLLIV
MZ298839/B.1.351/Beta NVTWFHAIHVSGTNGTKRFANPVLPFNDGVYFASTEKSNIIRGWIFGTTLDSKTQSLLIV
MZ913100/B.1.351/Beta NVTWFHAIHVSGTNGTKRFANPVLPFNDGVYFASTEKSNIIRGWIFGTTLDSKTQSLLIV
MZ879365/B.1.351/Beta NVTWFHAIHVSGTNGTKRFANPVLPFNDGVYFASTEKSNIIRGWIFGTTLDSKTQSLLIV
OU525106/B.1.351/Beta NVTWFHAIHVSGTNGTKRFANPVLPFNDGVYFASTEKSNIIRGWIFGTTLDSKTQSLLIV
OU539352/B.1.351/Beta NVTWFHAIHVSGTNGTKRFANPVLPFNDGVYFASTEKSNIIRGWIFGTTLDSKTQSLLIV
MZ202314/B.1.351/Beta NVTWFHAIHVSGTNGTKRFANPVLPFNDGVYFASTEKSNIIRGWIFGTTLDSKTQSLLIV
MZ298840/B.1.351/Beta NVTWFHAIHVSGTNGTKRFANPVLPFNDGVYFASTEKSNIIRGWIFGTTLDSKTQSLLIV
MZ376663/B.1.351/Beta NVTWFHAIHVSGTNGTKRFANPVLPFNDGVYFASTEKSNIIRGWIFGTTLDSKTQSLLIV
LC643036/B.1.617.2/Delta NVTWFHAIHVSGTNGTKRFDNPVLPFNDGVYFASIEKSNIIRGWIFGTTLDSKTQSLLIV
LC643044/B.1.617.2/Delta NVTWFHAIHVSGTNGTKRFDNPVLPFNDGVYFASIEKSNIIRGWIFGTTLDSKTQSLLIV
MZ853946/B.1.617.2/Delta NVTWFHAIHVSGTNGTKRFDNPVLPFNDGVYFASTEKSNIIRGWIFGTTLDSKTQSLLIV
MZ854386/B.1.617.2/Delta NVTWFHAIHVSGTNGTKRFDNPVLPFNDGVYFASTEKSNIIRGWIFGTTLDSKTQSLLIV
MZ888532/B.1.617.2/Delta NVTWFHAIHVSGTNGTKRFDNPVLPFNDGVYFASTEKSNIIRGWIFGTTLDSKTQSLLIV
MZ888533/B.1.617.2/Delta NVTWFHAIHVSGTNGTKRFDNPVLPFNDGVYFASTEKSNIIRGWIFGTTLDSKTQSLLIV
MZ896136/B.1.617.2/Delta NVTWFHAIHVSGTNGTKRFDNPVLPFNDGVYFASTEKSNIIRGWIFGTTLDSKTQSLLIV
OU539261/B.1.617.2/Delta NVTWFHAIHVSGTNGTTRFDNPVLPFNDGVYFASTEKSNIIRGWIFGTTLDSKTQSLLIV
MZ854389/B.1.617.2/Delta NVTWFHAIHVSGTNGTKRFDNPVLPFNDGVYFASTEKSNIIRGWIFGTTLDSKTQSLLIV
OU562629/B.1.617.2/Delta NVTWFHAIHVSGTNGTKRFDNPVLPFNDGVYFASTEKSNIIRGWIFGTTLDSKTQSLLIV
OU539690/B.1.617.2/Delta NVTWFHAIHVSGTNGTKRFDNPVLPFNDGVYFASTEKSNIIRGWIFGTTLDSKTQSLLIV
MZ854387/B.1.617.2/Delta NVTWFHAIHVSGTNGTKRFDNPVLPFNDGVYFASTEKSNIIRGWIFGTTLDSKTQSLLIV
OU539784/P.1/Gamma NVTWFHAIHVSGTNVTKRFDNPVLPFNDGVYFASTEKSNIIRGWIFGTTLDSKTQSLLIV
OU539830/P.1/Gamma NVTWFHAIHVSGTNGTKRFDNPVLPFNDGVYFASTEKSNIIRGWIFGTTLDSKTQSLLIV
OU539894/P.1/Gamma NVTWFHAIHVSGTNGTKRFDNPVLPFNDGVYFASTEKSNIIRGWIFGTTLDSKTQSLLIV
MZ477748/P.1/Gamma NVTWFHAIHVSGTNGTKRFDNPVLPFNDGVYFASTEKSNIIRGWIFGTTLDSKTQSLLIV
MZ477746/P.1/Gamma NVTWFHAIHVSGTNGTKRFDNPVLPFNDGVYFASTEKSNIIRGWIFGTTLDSKTQSLLIV
MW938104/P.1/Gamma NVTWFHAIHVSGTNGTKRFDNPVLPFNDGVYFASTEKSNIIRGWIFGTTLDSKTQSLLIV
MZ896179/P.1/Gamma NVTWFHAIHVSGTNGTKRFDNPVLPFNDGVYFASTEKSNIIRGWIFGTTLDSKTQSLLIV
MZ896359/P.1/Gamma NVTWFHAIHVSGTNGTKRFDNPVLPFNDGVYFASTEKSNIIRGWIFGTTLDSKTQSLLIV
MZ896669/P.1/Gamma NVTWFHAIHVSGTNGTKRFDNPVLPFNDGVYFASTEKSNIIRGWIFGTTLDSKTQSLLIV
OU565182/P.1/Gamma NVTWFHAIHVSGTNGTKRFDNPVLPFNDGVYFASTEKSNIIRGWIFGTTLDSKTQSLLIV
OU492255/P.1/Gamma NVTWFHAIHVSGTNGTKRFDNPVLPFNDGVYFASTEKSNIIRGWIFGTTLDSKTQSLLIV
MZ911221/B.1.427/Epsilon NVTWFHAIHVSGTNGTKRFDNPVLPFNDGVYFASTEKSNIIRGWIFGTTLDSKTQSLLIV
MZ375854/B.1.427/Epsilon NVTWFHAIHVSGTNGTKRFDNPVLPFNDGVYFASTEKSNIIRGWIFGTTLDSKTQSLLIV
MZ375858/B.1.427/Epsilon NVTWFHAIHVSGTNGTKRFDNPVLPFNDGVYFASTEKSNIIRGWIFGTTLDSKTQSLLIV
MZ943834/B.1.429/Epsilon NVTWFHAIHVSGTNGTKRFDNPVLPFNDGVYFASTEKSNIIRGWIFGTTLDSKTQSLLIV
MZ277390/B.1.429/Epsilon NVTWFHAIHVSGTNGTKRFDNPVLPFNDGVYFASTEKSNIIRGWIFGTTLDSKTQSLLIV
OU282717/B.1.429/Epsilon NVTWFHAIHVSGTNGTKRFDNPVLPFNDGVYFASTEKSNIIRGWIFGTTLDSKTQSLLIV
OU235436/B.1.429/Epsilon NVTWFHAIHVSGTNGTKRFDNPVLPFNDGVYFASTEKSNIIRGWIFGTTLDSKTQSLLIV
MZ687447/B.1.429/Epsilon NVTWFHAIHVSGTNGTKRFDNPVLPFNDGVYFASTEKSNIIRGWIFGTTLDSKTQSLLIV
MZ538882/B.1.617.1/Kappa NVTWFHAIHVSGTNGTKRFDNPVLPFNDGVYFASIEKSNIIRGWIFGTTLDSKTQSLLIV
MZ724425/B.1.617.1/Kappa NVTWFHAIHVSGTNGTKRFDNPVLPFNDGVYFASIEKSNIIRGWIFGTTLDSKTQSLLIV
MZ363839/B.1.617.1/Kappa NVTWFHAIHVSGTNGTKRFDNPVLPFNDGVYFASIEKSNIIRGWIFGTTLDSKTQSLLIV
MZ332524/B.1.617.1/Kappa NVTWFHAIHVSGTNGTKRFDNPVLPFNDGVYFASIEKSNIIRGWIFGTTLDSKTQSLLIV
MZ332527/B.1.617.1/Kappa NVTWFHAIHVSGTNGTKRFDNPVLPFNDGVYFASTEKSNIIRGWIFGTTLDSKTQSLLIV
LC633761/B.1.617.1/Kappa NVTWFHAIHVSGTNGTKRFDNPVLPFNDGVYFASIEKSNIIRGWIFGTTLDSKTQSLLIV
MZ562746/B.1.617.1/Kappa NVTWFHAIHVSGTNGTKRFDNPVLPFNDGVYFASIEKSNIIRGWIFGTTLDSKTQSLLIV
MZ571142/B.1.617.1/Kappa NVTWFHAIHVSGTNGTKRFDNPVLPFNDGVYFASIEKSNIIRGWIFGTTLDSKTQSLLIV
OU168413/B.1.617.1/Kappa NVTWFHAIHVSGTNGTKRFDNPVLPFNDGVYFASIEKSNIIRGWIFGTTLDSKTQSLLIV
MZ026886/B.1.617.1/Kappa NVTWFHAIHVSGTNGTKRFDNPVLPFNDGVYFASIEKSNIIRGWIFGTTLDSKTQSLLIV
OU172184/B.1.617.1/Kappa NVTWFHAIHVSGTNGTKRFDNPVLPFNDGVYFASIEKSNIIRGWIFGTTLDSKTQSLLIV
MZ397173/B.1.617.1/Kappa NVTWFHAIHVSGTNGTKRFDNPVLPFNDGVYFASIEKSNIIRGWIFGTTLDSKTQSLLIV
MZ397176/B.1.617.1/Kappa NVTWFHAIHVSGTNGTKRFDNPVLPFNDGVYFASIEKSNIIRGWIFGTTLDSKTQSLLIV
MZ397175/B.1.617.1/Kappa NVTWFHAIHVSGTNGTKRFDNPVLPFNDGVYFASIEKSNIIRGWIFGTTLDSKTQSLLIV
MZ397177/B.1.617.1/Kappa NVTWFHAIHVSGTNGTKRFDNPVLPFNDGVYFAFTEKSNIIRGWIFGTTLDSKTQSLLIV
OU290035/B.1.617.1/Kappa NVTWFHAIHVSGTNGTKRFDNPVLPFNDGVYFASTEKSNIIRGWIFGTTLDSKTQSLLIV
OU265178/B.1.617.1/Kappa NVTWFHAIHVSGTNGTKRFDNPVLPFNDGVYFASTEKSNIIRGWIFGTTLDSKTQSLLIV
OU254949/B.1.617.1/Kappa NVTWFHAIHVSGTNGTKRFDNPVLPFNDGVYFASTEKSNIIRGWIFGTTLDSKTQSLLIV
OU247018/B.1.617.1/Kappa NVTWFHAIHVSGTNGTKRFDNPVLPFNDGVYFASTEKSNIIRGWIFGTTLDSKTQSLLIV
OU061269/B.1.617.1/Kappa NVTWFHAIHVSGTNGTKRFDNPVLPFNDGVYFASTEKSNIIRGWIFGTTLDSKTQSLLIV
OU182966/B.1.617.1/Kappa NVTWFHAIHVSGTNGTKRFDNPVLPFNDGVYFASTEKSNIIRGWIFGTTLDSKTQSLLIV
MZ746235/B.1.617.1/Kappa NVTWFHAIHVSGTNGTKRFDNPVLPFNDGVYFASIEKSNIIRGWIFGTTLDSKTQSLLIV
MZ487800/B.1.617.1/Kappa NVTWFHAIHVSGTNGTKRFDNPVLPFNDGVYFASTEKSNIIRGWIFGTTLDSKTQSLLIV
OU322235/B.1.617.1/Kappa NVTWFHAIHVSGTNGTKRFDNPVLPFNDGVYFASTEKSNIIRGWIFGTTLDSKTQSLLIV
MZ953185/C.37/Lambda NVTWFHAIHVSGTNVIKRFDNPVLPFNDGVYFASTEKSNIIRGWIFGTTLDSKTQSLLIV
MZ937666/C.37/Lambda NVTWFHAIHVSGTNVIKRFDNPVLPFNDGVYFASTEKSNIIRGWIFGTTLDSKTQSLLIV
OU356036/C.37/Lambda NVTWFHAIHVSGTNVIKRFDNPVLPFNDGVYFASTEKSNIIRGWIFGTTLDSKTQSLLIV
OU351967/C.37/Lambda NVTWFHAIHVSGTNVIKRFDNPVLPFNDGVYFASTEKSNIIRGWIFGTTLDSKTQSLLIV
OU053018/C.37/Lambda NVTWFHAIHVSGTNVIKRFDNPVLPFNDGVYFASTEKSNIIRGWIFGTTLDSKTQSLLIV
OU268528/C.37/Lambda NVTWFHAIHVSGTNVIKRFDNPVLPFNDGVYFASTEKSNIIRGWIFGTTLDSKTQSLLIV
OU100999/C.37/Lambda NVTWFHAIHVSGTNVIKRFDNPVLPFNDGVYFASTEKSNIIRGWIFGTTLDSKTQSLLIV
MZ275295/C.37/Lambda NVTWFHAIHVSGTNVIKRFDNPVLPFNDGVYFASTEKSNIIRGWIFGTTLDSKTQSLLIV
MZ275301/C.37/Lambda NVTWFHAIHVSGTNVIKRFDNPVLPFNDGVYFASTEKSNIIRGWIFGTTLDSKTQSLLIV
MZ908711/C.37/Lambda NVTWFHAIHVSGTNVIKRFDNPVLPFNDGVYFASTEKSNIIRGWIFGTTLDSKTQSLLIV
MZ496613/C.37/Lambda NVTWFHAIHVSGTNVIKRFDNPVLPFNDGVYFASTEKSNIIRGWIFGTTLDSKTQSLLIV
OU517038/C.37/Lambda NVTWFHAIHVSGTNVIKRFDNPVLPFNDGVYFASTEKSNIIRGWIFGTTLDSKTQSLLIV
MZ953277/C.37/Lambda NVTWFHAIHVSGTNVIKRFDNPVLPFNDGVYFASTEKSNIIRGWIFGTTLDSKTQSLLIV
MZ942542/C.37/Lambda NVTWFHAIHVSGTNVIKRFDNPVLPFNDGVYFASTEKSNIIRGWIFGTTLDSKTQSLLIV
MZ857990/C.37/Lambda NVTWFHAIHVSGTNVIKRFDNPVLPFNDGVYFASTEKSNIIRGWIFGTTLDSKTQSLLIV

```
                                     130       140       150       160       170       180
                                       .         .         .         .         .         .
MN908947/Wuhan-Hu-1            NNATNVVIKVCEFQFCNDPFLGVYYHKNNKSWMESEFRVYSSANNCTFEYVSQPFLMDLE
BS001137/B.1.1.7/Alpha         NNATNVVIKVCEFQFCNDPFLG..YHKNNKSWMESEFRVYSSANNCTFEYVSQPFLMDLE
BS001138/B.1.1.7/Alpha         NNATNVVIKVCEFQFCNDPFLG..YHKNNKSWMESEFRVYSSANNCTFEYVSQPFLMDLE
BS001139/B.1.1.7/Alpha         NNATNVVIKVCEFQFCNDPFLG..YHKNNKSWMESEFRVYSSANNCTFEYVSQPFLMDLE
BS001140/B.1.1.7/Alpha         NNATNVVIKVCEFQFCNDPFLG..YYKNNKSWMESEFRVYSSANNCTFEYVSQPFLMDLE
MW913362/B.1.1.7/Alpha         NNATNVVIKVCEFQFCNDPFLG..YHKNNKSWMESEFRVYSSANNCTFEYVSQPFLMDLE
MZ266365/B.1.1.7/Alpha         NNATNVVIKVCEFQFCNDPFLG..YHKNNKSWMESEFRVYSSANNCTFEYVSQPFLMDLE
MZ350105/B.1.1.7/Alpha         NNATNVVIKVCEFQFCNDPFLG..YHKNNKSWMESEFRVYSSANNCTFEYVSQPFLMDLE
MZ350109/B.1.1.7/Alpha         NNATNVVIKVCEFQFCNDPFLG..YHKNNKSWMESEFRVYSSANNCTFEYVSQPFLMDLE
MZ888516/B.1.1.7/Alpha         NNATNVVIKVCEFQFCNDPFLG..YHKNNKSWMESEFRVYSSANNCTFEYVSQPFLMDLE
OU562607/B.1.1.7/Alpha         NNATNVVIKVCEFQFCNDPFLG..YHKNNKSWMESEFRVYSSANNCTFEYVSQPFFMDLE
OU514166/B.1.1.7/Alpha         NNATNVVIKVCEFQFCNDPFLG..YHKNNKSWMESEFRVYSSANNCTFEYVSQPFLMDLE
OU538851/B.1.1.7/Alpha         NNATNVVIKVCEFQFCNDPFLG..YHKNNKSWMESEFRVYSSANNCTFEYVSQPFLMDLE
OU538849/B.1.1.7/Alpha         NNATNVVIKVCEFQFCNDPFLG..YHKNNKSWMESEFRVYSSANNCTFEYVSQPFLMDLE
MZ895877/B.1.1.7/Alpha         NNATNVVIKVCEFQFCNHPFLG..YHKNNKSWMESEFRVYSSANNCTFEYVSQPFLMDLE
OU514176/B.1.1.7/Alpha         NNATNVVIKVCEFQFCNDPFLG..YHKNNKSWMESEFRVYSSANNCTFEYVSQPFLMDLE
OU562608/B.1.1.7/Alpha         NNATNVVIKVCEFQFCNDPFLG..YHKNNKSWMESEFRVYSSANNCTFEYVSQPFLMDLE
MW981442/B.1.351/Beta          NNATNVVIKVCEFQFCNDPFLGVYYHKNNKSWMESEFRVYSSANNCTFEYVSQPFLMDLE
MZ298839/B.1.351/Beta          NNATNVVIKVCEFQFCNDPFLGVYYHKNNKSWMESEFRVYSSANNCTFEYVSQPFLMDLE
MZ913100/B.1.351/Beta          NNATNVVIKVCEFQFCNYPFLGVYYHKNNKSWMESEFRVYSSANNCTFEYVSQPFLMDLE
MZ879365/B.1.351/Beta          NNATNVVIKVCEFQFCNDPFLGVYYHKNNKSWMESEFRVYSSANNCTFEYVSQPFLMDLE
OU525106/B.1.351/Beta          NNATNVVIKVCEFQFCNDPFLGVYYHKNNKSWMESEFRVYSSANNCTFEYVSQPFLMDLE
OU539352/B.1.351/Beta          NNATNVVIKVCEFQFCNDPFLGVYYHKNNKSWMESEFRVYSSANNCTFEYVSQPFLMDLE
MZ202314/B.1.351/Beta          NNATNVVIKVCEFQFCNDPFLGVYYHKNNKSWMESEFRVYSSANNCTFEYVSQPFLMDLE
MZ298840/B.1.351/Beta          NNATNVVIKVCEFQFCNDPFLGVYYHKNNKSWMESEFRVYSSANNCTFEYVSQPFLMDLE
MZ376663/B.1.351/Beta          NNATNVVIKVCEFQFCNDPFLGVYYHKNNKSWMESEFRVYSSANNCTFEYVSQPFLMDLE
LC643036/B.1.617.2/Delta       NNATNVVIKVCEFQFCNDPFLDVYYHKNNKSWMES...VYSSANNCTFEYVSQPFLMDLE
LC643044/B.1.617.2/Delta       NNATNVVIKVCEFQFCNDPFLDVYYHKNNKSWMES...VYSSANNCTFEYVSQPFLMDLE
MZ853946/B.1.617.2/Delta       NNATNVVIKVCEFQFCNDPFLDVYYHKNNKSWMES...VYSSANNCTFEYVSQPFLMDLE
MZ854386/B.1.617.2/Delta       NNATNVVIKVCEFQFCNDPFLDVYYHKNNKSWMESE.GVYSSANNCTFEYVSQPFLMDLE
MZ888532/B.1.617.2/Delta       NNATNVVIKVCEFQFCNDPFLGVYYHKNNKSWMES...VYSSANNCTFEYVSQPFLMDLE
MZ888533/B.1.617.2/Delta       NNATNVVIKVCEFQFCNDPFLDVYYHKNNKSWMES...VYSSANNCTFEYVSQPFLMDLE
MZ896136/B.1.617.2/Delta       NNATNVVIKVCEFQFCNDPFLDVYYHKNNKSWMES...VYSSANNCTFEYVSQPFLMDLE
OU539261/B.1.617.2/Delta       NNATNVVIKVCEFQFCNDPFLGVYYHKNNKSWMES...VYSSANNCTFEYVSQPFLMDLE
MZ854389/B.1.617.2/Delta       NNATNVVIKVCEFQFCNDPFLDVYYHKNNKSWMES...VYSSANNCTFEYVSQPFLMDLE
OU562629/B.1.617.2/Delta       NNATNVVIKVCEFQFCNDPFLDVYYHKNNKSWMES...VYSSANNCTFEYVSQPFLMDLE
OU539690/B.1.617.2/Delta       NNATNVVIKVCEFQFCNDPFLGVYYHKNNKSWMES...VYSSANNCTFEYVSQPFLMDLE
MZ854387/B.1.617.2/Delta       NNATNVVIKVCEFQFCNDPFLDVYYHKNNKSWMESE.RVYSSANNCTFEYVSQPFLMDLE
OU539784/P.1/Gamma             NNATNVVIKVCEFQFCNYPFLGVYYHKNNKSWMESEFRVYSSANNCTFEYVSQPFLMDLE
OU539830/P.1/Gamma             NNATNVVIKVCEFQFCNYPFLGVYYHKNNKSWMESEFRVYSSANNCTFEYVSQPFLMDLE
OU539894/P.1/Gamma             NNATNVVIKVCEFQFCNYPFLGVYYHKNNKSWMESEFRVYSSANNCTFEYVSQPFLMDLE
MZ477748/P.1/Gamma             NNATNVVIKVCEFQFCNYPFLGVYYHKNNKSWMESEFRVYSSANNCTFEYVSQPFLMDLE
MZ477746/P.1/Gamma             NNATNVVIKVCEFQFCNYPFLGVYYHKNNKSWMESEFRVYSSANNCTFEYVSQPFLMDLE
MW938104/P.1/Gamma             NNATNVVIKVCEFQFCNYPFLGVYYHKNNKSWMESEFRVYSSANNCTFEYVSQPFLMDLE
MZ896179/P.1/Gamma             NNATNVVIKVCEFQFCNYPFLGVYYHKNNKSWMESEFRVYSSANNCTFEYVSQPFLMDLE
MZ896359/P.1/Gamma             NNATNVVIKVCEFQFCNYPFLGVYYHKNNKSWMESEFRVYSSANNCTFEYVSQPFLMDLE
MZ896669/P.1/Gamma             NNATNVVIKVCEFQFCNYPFLGVYYHKNNKSWMESEFRVYSSANNCTFEYVSQPFLMDLE
OU565182/P.1/Gamma             NNATNVVIKVCEFQFCNYPFLGVYYHKNNKSWMESEFRVYSSANNCTFEYVSQPFLMDLE
OU492255/P.1/Gamma             NNATNVVIKVCEFQFCNYPFLGVYYHKNNKSWMESEFRVYSSANNCTFEYVSQPFLMDLE
MZ911221/B.1.427/Epsilon       NNATNVVIKVCEFQFCNDPFLGVYYHKNNKSCMESEFRVYSSANNCTFEYVSQPFLMDLE
MZ375854/B.1.427/Epsilon       NNATNVVIKVCEFQFCNDPFLGVYYHKNNKSCMESEFRVYSSANNCTFEYVSQPFLMDLE
MZ375858/B.1.427/Epsilon       NNATNVVIKVCEFQFCNDPFLGVYYHKNNKSCMESEFRVYSSANNCTFEYVSQPFLMDLE
MZ943834/B.1.429/Epsilon       NNATNVVIKVCEFQFCNDPFLGVYYHKNNKSCMESEFRVYSSANNCTFEYVSQPFLMDLE
MZ277390/B.1.429/Epsilon       NNATNVVIKVCEFQFCNDPFLGVYYHKNNKSCMESEFRVYSSANNCTFEYVSQPFLMDLE
OU282717/B.1.429/Epsilon       NNATNVVIKVCEFQFCNDPFLGVYYHKNNKSCMESEFRVYSSANNCTFEYVSQPFLMDLE
OU235436/B.1.429/Epsilon       NNATNVVIKVCEFQFCNDPFLGVYYHKNNKSCMESEFRVYSSANNCTFEYVSQPFLMDLE
MZ687447/B.1.429/Epsilon       NNATNVVIKVCEFQFCNDPFLGVYYHKNNKSCMESEFRVYSSANNCTFEYVSQPFLMDLE
MZ538882/B.1.617.1/Kappa       NNATNVVIKVCEFQFCNDPFLDVYYHKNNKSWMKSEFRVYSSANNCTFEYVSQPFLMDLE
MZ724425/B.1.617.1/Kappa       NNATNVVIKVCEFQFCNDPFLDVYYHKNNKSWMKSEFRVYSSANNCTFEYVSQPFLMDLE
MZ363839/B.1.617.1/Kappa       NNATNVVIKVCEFQFCNDPFLDVYYHKNNKSWMESEFRVYSSANNCTFEYVSQPFLMDLE
MZ332524/B.1.617.1/Kappa       NNATNVVIKVCEFQFCNDPFLDVYYHKNNKSWMESEFRVYSSANNCTFEYVSQPFLMDLE
MZ332527/B.1.617.1/Kappa       NNATNVVIKVCEFQFCNDPFLGVYYHKNNKSWMESEFRVYSSANNCTFEYVSQPFLMDLE
LC633761/B.1.617.1/Kappa       NNATNVVIKVCEFQFCNDPFLDVYYHKNNKSWMKSEFRVYSSANNCTFEYVSQPFLMDLE
MZ562746/B.1.617.1/Kappa       NNATNVVIKVCEFQFCNDPFLDVYYHKNNKSWMKSEFRVYSSANNCTFEYVSQPFLMDLE
MZ571142/B.1.617.1/Kappa       NNATNVVIKVCEFQFCNDPFLDVYYHKNNKSWMKSEFRVYSSANNCTFEYVSQPFLMDLE
OU168413/B.1.617.1/Kappa       NNATNVVIKVCEFQFCNDPFLDVYYHKNNKSWMKSEFRVYSSANNCTFEYVSQPFLMDLE
MZ026886/B.1.617.1/Kappa       NNATNVVIKVCEFQFCNDPFLGVYYHKNNKSWMKSEFRVYSSANNCTFEYVSQPFLMDLE
OU172184/B.1.617.1/Kappa       NNATNVVIKVCEFQFCNDPFLGVYYHKNNKSWMESEFRVYSSANNCTFEYVSQPFLMDLE
MZ397173/B.1.617.1/Kappa       NNATNVVIKVCEFQFCNDPFLDVYYHKNNKSWMKSEFRVYSSANNCTFEYVSQPFLMDLE
MZ397176/B.1.617.1/Kappa       NNATNVVIKVCEFQFCNDPFLDVYYHKNNKSWMKSEFRVYSSANNCTFEYVSQPFLMDLE
MZ397175/B.1.617.1/Kappa       NNATNVVIKVCEFQFCNDPFLDVYYHKNNKSWMKSEFRVYSSANNCTFEYVSQPFLMDLE
MZ397177/B.1.617.1/Kappa       NNATNVVIKVCEFQFCNDPFLDVYYHKNNKSWMKSEFRVYSSANNCTFEYVSQPFLMDLE
OU290035/B.1.617.1/Kappa       NNATNVVIKVCEFQFCNDPFLDVYYHKNNKSWMKSEFRVYSSANNCTFEYVSQPFLMDLE
OU265178/B.1.617.1/Kappa       NNATNVVIKVCEFQFCNDPFLDVYYHKNNKSWMKSEFRVYSSANNCTFEYVSQPFLMDLE
OU254949/B.1.617.1/Kappa       NNATNVVIKVCEFQFCNDPFLDVYYHKNNKSWMKSEFRVYSSANNCTFEYVSQPFLMDLE
OU247018/B.1.617.1/Kappa       NNATNVVIKVCEFQFCNDPFLDVYYHKNNKSWMKSEFRVYSSANNCTFEYVSQPFLMDLE
OU061269/B.1.617.1/Kappa       NNATNVVIKVCEFQFCNDPFLDVYYHKNNKSWMKSEFRVYSSANNCTFEYVSQPFLMDLE
OU182966/B.1.617.1/Kappa       NNATNVVIKVCEFQFCNDPFLDVYYHKNNKSWMKSEFRVYSSANNCTFEYVSQPFLMDLE
MZ746235/B.1.617.1/Kappa       NNATNVVIKVCEFQFCNDPFLDVYYHKNNKSWMKSEFRVYSSANNCTFEYVSQPFLMDLE
MZ487800/B.1.617.1/Kappa       NNATNVVIKVCEFQFCNDPFLDVYYHKNNKSWMKSEFRVYSSANNCTFEYVSQPFLMDLE
OU322235/B.1.617.1/Kappa       NNATNVVIKVCEFQFCNDPFLDVYYHKNNKSWMKSEFRVYSSANNCTFEYVSQPFLMDLE
MZ953185/C.37/Lambda           NNATNVVIKVCEFQFCNDPFLGVYYHKNNKSWMESEFRVYSSANNCTFEYVSQPFLMDLE
MZ937666/C.37/Lambda           NNATNVVIKVCEFQFCNDPFLGVYYHKNNKSWMESEFRVYSSANNCTFEYVSQPFLMDLE
OU356036/C.37/Lambda           NNATNVVIKVCEFQFCNDPFLGVYYHKNNKSWMESEFRVYSSANNCTFEYVSQPFLMDLE
OU351967/C.37/Lambda           NNATNVVIKVCEFQFCNDPFLGVYYHKNNKSWMESEFRVYSSANNCTFEYVSQPFLMDLE
OU053018/C.37/Lambda           NNATNVVIKVCEFQFCNDPFLGVYYHKNNKSWMESEFRVYSSANNCTFEYVSQPFLMDLE
OU268528/C.37/Lambda           NNATNVVIKVCEFQFCNDPFLGVYYHKNNKSWMESEFRVYSSANNCTFEYVSQPFLMDLE
OU100999/C.37/Lambda           NNATNVVIKVCEFQFCNDPFLGVYYHKNNKSWMESEFRVYSSANNCTFEYVSQPFLMDLE
MZ275295/C.37/Lambda           NNATNVVIKVCEFQFCNDPFLGVYYHKNNKSWMESEFRVYSSANNCTFEYVSQPFLMDLE
MZ275301/C.37/Lambda           NNATNVVIKVCEFQFCNDPFLGVYYHKNNKSWMESEFRVYSSANNCTFEYVSQPFLMDLE
MZ908711/C.37/Lambda           NNATNVVIKVCEFQFCNDPFLGVYYHKNNKSWMESEFRVYSSANNCTFEYVSQPFLMDLE
MZ496613/C.37/Lambda           NNATNVVIKVCEFQFCNDPFLGVYYHKNNKSWMESEFRVYSSANNCTFEYVSQPFLMDLE
OU517038/C.37/Lambda           NNATNVVIKVCEFQFCNDPFLGVYYHKNNKSWMESELRVYSSANNCTFEYVSQPFLMDLE
MZ953277/C.37/Lambda           NNATNVVIKVCEFQFCNDPFLGVYYHKNNKSWMESEFRVYSSANNCTFEYVSQPFLMDLE
MZ942542/C.37/Lambda           NNATNVVIKVCEFQFCNDPFLGVYYHKNNKSWMESEFRVYSSANNCTFEYVSQPFLMDLE
MZ857990/C.37/Lambda           NNATNVVIKVCEFQFCNDPFLGVYYHKNNKSWMESEFRVYSSANNCTFEYVSQPFLMDLE
```

```
                                   190       200       210       220       230       240
MN908947/Wuhan-Hu-1        GKQGNFKNLREFVFKNIDGYFKIYSKHTPINLVRDLPQGFSALEPLVDLPIGINITRFQT
BS001137/B.1.1.7/Alpha     GKQGNFKNLREFVFKNIDGYFKIYSKHTPINLVRDLPQGFSALEPLVDLPIGINITRFQT
BS001138/B.1.1.7/Alpha     GKQGNFKNLREFVFKNIDGYFKIYSKHTPINLVRDLPQGFSALEPLVDLPIGINITRFQT
BS001139/B.1.1.7/Alpha     GKQGNFKNLREFVFKNIDGYFKIYSKHTPINLVRDLPQGFSALEPLVDLPIGINITRFQT
BS001140/B.1.1.7/Alpha     GKQGNFKNLREFVFKNIDGYFKIYSKHTPINLVRDLPQGFSALEPLVDLPIGINITRFQT
MW913362/B.1.1.7/Alpha     GKQGNFKNLREFVFKNIDGYFKIYSKHTPINLVRDLPQGFSALEPLVDLPIGINITRFQT
MZ266365/B.1.1.7/Alpha     GKQGNFKNLREFVFKNIDGYFKIYSKHTPINLVRDLPQGFSALEPLVDLPIGINITRFQT
MZ350105/B.1.1.7/Alpha     GKQGNFKNLREFVFKNIDGYFKIYSKHTPINLVRDLPQGFSALEPLVDLPIGINITRFQT
MZ350109/B.1.1.7/Alpha     GKQGNFKNLREFVFKNIDGYFKIYSKHTPINLVRDLPQGFSALEPLVDLPIGINITRFQT
MZ888516/B.1.1.7/Alpha     GKQGNFKNLREFVFKNIDGYFKIYSKHTPINLVRDLPQGFSALEPLVDLPIGINITRFQT
OU562607/B.1.1.7/Alpha     GKQGNFKNLREFVFKNIDGYFKIYSKHTPINLVRDLPQGFSALEPLVDLPIGINITRFQT
OU514166/B.1.1.7/Alpha     GKQGNFKNLREFVFKNIDGYFKIYSKHTPINLVRDLPQGFSALEPLVDLPIGINITRFQT
OU538851/B.1.1.7/Alpha     GKQGNFKNLREFVFKNIDGYFKIYSKHTPINLVRDLPQGFSALEPLVDLPIGINITRFQT
OU538849/B.1.1.7/Alpha     GKQGNFKNLREFVFKNIDGYFKIYSKHTPINLVRDLPQGFSALEPLVDLPIGINITRFQT
MZ895877/B.1.1.7/Alpha     GKQGNFKNLREFVFKNIDGYFKIYSKHTPINLVRDLPQGFSALEPLVDLPIGINITRFQT
OU514176/B.1.1.7/Alpha     GKQGNFKNLREFVFKNIDGYFKIYSKHTPINLVRDLPQGFSALEPLVDLPIGINITRFQT
OU562608/B.1.1.7/Alpha     GKQGNFKNLREFVFKNIDGYFKIYSKHTPINLVRDLPQGFSALEPLVDLPIGINITRFQT
MW981442/B.1.351/Beta      GKQGNFKNLREFVFKNIDGYFKIYSKHTPINLVRGLPQGFSALEPLVDLPIGINITRFQT
MZ298839/B.1.351/Beta      GKQGNFKNLREFVFKNIDGYFKIYSKHTPINLVRGLPQGFSALEPLVDLPIGINITRFQT
MZ913100/B.1.351/Beta      GKQGNFKNLREFVFKNIDGYFKIYSKHTPINLVRGLPQGFSALEPLVDLPIGINITRFQT
MZ879365/B.1.351/Beta      GKQGNFKNLREFVFKNIDGYFKIYSKHTPINLVRGLPQGFSALEPLVDLPIGINITRFQT
OU525106/B.1.351/Beta      GKQGNFKNLREFVFKNIDGYFKIYSKHTPINLVRGLPQGFSALEPLVDLPIGINITRFQT
OU539352/B.1.351/Beta      GKQGNFKNLREFVFKNIDGYFKIYSKHTPINLVRGLPQGFSALEPLVDLPIGINITRFQT
MZ202314/B.1.351/Beta      GKQGNFKNLREFVFKNIDGYFKIYSKHTPINLVRGLPQGFSALEPLVDLPIGINITRFQT
MZ298840/B.1.351/Beta      GKQGNFKNLREFVFKNIDGYFKIYSKHTPINLVRGLPQGFSALEPLVDLPIGINITRFQT
MZ376663/B.1.351/Beta      GKQGNFKNLREFVFKNIDGYFKIYSKHTPINLVRGLPQGFSALEPLVDLPIGINITRFQT
LC643036/B.1.617.2/Delta   GKQGNFKNLREFVFKNIDGYFKIYSKHTPINLVRDLPHGFSALEPLVDLPIGINITRFQT
LC643044/B.1.617.2/Delta   GKQGNFKNLREFVFKNIDGYFKIYSKHTPINLVRDLPHGFSALEPLVDLPIGINITRFQT
MZ853946/B.1.617.2/Delta   GKQGNFKNLREFVFKNIDGYFKIYSKHTPINLVRDLPQGFSALEPLVDLPIGINITRFQT
MZ854386/B.1.617.2/Delta   GKQGNFKNLREFVFKNIDGYFKIYSKHTPINLVRDLPQGFSALEPLVDLPIGINITRFQT
MZ888532/B.1.617.2/Delta   GKQGNFKNLREFVFKNIDGYFKIYSKHTPINLVRDLPQGFSALEPLVDLPIGINITRFQT
MZ888533/B.1.617.2/Delta   GKQGNFKNLREFVFKNIDGYFKIYSKHTPINLVRDLPQGFSALEPLVDLPIGINITRFQT
MZ896136/B.1.617.2/Delta   GKQGNFKNLREFVFKNIDGYFKIYSKHTPINLVRDLPQGFSVLEPLVDLPIGINITRFQT
OU539261/B.1.617.2/Delta   GKQGNFKNLREFVFKNIDGYFKIYSKHTPINLVRDLPQGFSALEPLVDLPIGINITRFQT
MZ854389/B.1.617.2/Delta   GKQGNFKNLREFVFKNIDGYFKIYSKHTPINLVRDLPQGFSALEPLVDLPIGINITRFQT
OU562629/B.1.617.2/Delta   GKQGNFKNLREFVFKNIDGYFKIYSKHTPINLVRDLPQGFSALEPLVDLPIGINITRFQT
OU539690/B.1.617.2/Delta   GKQGNFKNLREFVFKNIDGYFKIYSKHTPINLVRDLPQGFSALEPLVDLPIGINITRFQT
MZ854387/B.1.617.2/Delta   GKQGNFKNLREFVFKNIDGYFKIYSKHTPINLVRDLPQGFSALEPLVDLPIGINITRFQT
OU539784/P.1/Gamma         GKQGNFKNLSEFVFKNIDGYFKIYSKHTPINLVRDLPQGFSALEPLVDLPIGINITRFQT
OU539830/P.1/Gamma         GKQGNFKNLSEFVFKNIDGYFKIYSKHTPINLVRDLPQGFSALEPLVDLPIGINITRFQT
OU539894/P.1/Gamma         GKQGNFKNLSEFVFKNIDGYFKIYSKHTPINLVRDLPQGFSALEPLVDLPIGINITRFQT
MZ477748/P.1/Gamma         GKQGNFKNLSEFVFKNIDGYFKIYSKHTPINLVRDLPQGFSALEPLVDLPIGINITRFQT
MZ477746/P.1/Gamma         GKQGNFKNLSEFVFKNIDGYFKIYSKHTPINLVRDLPQGFSALEPLVDLPIGINITRFQT
MW938104/P.1/Gamma         GKQGNFKNLSEFVFKNIDGYFKIYSKHTPINLVRDLPQGFSALEPLVDLPIGINITRFQT
MZ896179/P.1/Gamma         GKQGNFKNLSEFVFKNIDGYFKIYSKHTPINLVRDLPQGFSALEPLVDLPIGINITRFQT
MZ896359/P.1/Gamma         GKQGNFKNLSEFVFKNIDGYFKIYSKHTPINLVRDLPQGFSALEPLVDLPIGINITRFQT
MZ896669/P.1/Gamma         GKQGNFKNLSEFVFKNIDGYFKIYSKHTPINLVRDLPQGFSALEPLVDLPIGINITRFQT
OU565182/P.1/Gamma         GKQGNFKNLSEFVFKNIDGYFKIYSKHTPINLVRDLPQGFSALEPLVDLPIGINITRFQT
OU492255/P.1/Gamma         GKQGNFKNLSEFVFKNIDGYFKIYSKHTPINLVRDLPQGFSALEPLVDLPIGINITRFQT
MZ911221/B.1.427/Epsilon   GKQGNFKNLREFVFKNIDGYFKIYSKHTPINLVRDLPQGFSALEPLVDLPIGINITRFQT
MZ375854/B.1.427/Epsilon   GKQGNFKNLREFVFKNIDGYFKIYSKHTPINLVRDLPQGFSALEPLVDLPIGINITRFQT
MZ375858/B.1.427/Epsilon   GKQGNFKNLREFVFKNIDGYFKIYSKHTPINLVRDLPQGFSALEPLVDLPIGINITRFQT
MZ943834/B.1.429/Epsilon   GKQGNFKNLREFVFKNIDGYFKIYSKHTPINLVRDLPQGFSALEPLVDLPIGINITRFQT
MZ277390/B.1.429/Epsilon   GKQGNFKNLREFVFKNIDGYFKIYSKHTPINLVRDLPQGFSALEPLVDLPIGINITRFQT
OU282717/B.1.429/Epsilon   GKQGNFKNLREFVFKNIDGYFKIYSKHTPINLVRDLPQGFSALEPLVDLPIGINITRFQT
OU235436/B.1.429/Epsilon   GKQGNFKNLREFVFKNIDGYFKIYSKHTPINLVRDLPQGFSALEPLVDLPIGINITRFQT
MZ687447/B.1.429/Epsilon   GKQGNFKNLREFVFKNIDGYFKIYSKHTPINLVRDLPQGFSALEPLVDLPIGINITRFQT
MZ538882/B.1.617.1/Kappa   GKQGNFKNLREFVFKNIDGYFKIYSKHTPINLVRDLPQGFSALEPLVDLPIGINITRFQT
MZ724425/B.1.617.1/Kappa   GKQGNFKNLREFVFKNIDGYFKIYSKHTPINLVRDLPQGFSALEPLVDLPIGINITRFQT
MZ363839/B.1.617.1/Kappa   GKQGNFKNLREFVFKNIDGYFKIYSKHTPINLVRDLPQGFSALEPLVDLPIGINITRFQT
MZ332524/B.1.617.1/Kappa   GKQGNFKNLREFVFKNIDGYFKIYSKHTPINLVRDLPQGFSALEPLVDLPIGINITRFQT
MZ332527/B.1.617.1/Kappa   GKQGNFKNLREFVFKNIDGYFKIYSKHTPINLVRDLPQGFSALEPLVDLPIGINITRFQT
LC633761/B.1.617.1/Kappa   GKQGNFKNLREFVFKNIDGYFKIYSKHTPINLVRDLPQGFSALEPLVDLPIGINITRFQT
MZ562746/B.1.617.1/Kappa   GKQGNFKNLREFVFKNIDGYFKIYSKHTPINLVRDLPQGFSALEPLVDLPIGINITRFQT
MZ571142/B.1.617.1/Kappa   GKQGNFKNLREFVFKNIDGYFKIYSKHTPINLVRDLPQGFSALEPLVDLPIGINITRFQT
OU168413/B.1.617.1/Kappa   GKQGNFKNLREFVFKNIDGYFKIYSKHTPINLVRDLPQGFSALEPLVDLPIGINITRFQT
MZ026886/B.1.617.1/Kappa   GKQGNFKNLREFVFKNIDGYFKIYSKHTPINLVRDLPQGFSALEPLVDLPIGINITRFQT
OU172184/B.1.617.1/Kappa   GKQGNFKNLREFVFKNIDGYFKIYSKHTPINLVRDLPQGFSALEPLVDLPIGINITRFQT
MZ397173/B.1.617.1/Kappa   GKQGNFKNLREFVFKNIDGYFKIYSKHTPINLVRDLPQGFSALEPLVDLPICINITRFQT
MZ397176/B.1.617.1/Kappa   GKQGNFKNLREFVFKNIDGYFKIYSKHTPINLVRDLPQGFSALEPLVDLPICINITRFQT
MZ397175/B.1.617.1/Kappa   GKQGNFKNLREFVFKNIDGYFKIYSKHTPINLVRDLPQGFSALEPLVDLPICINITRFQT
MZ397177/B.1.617.1/Kappa   GKQGNFKNLREFVFKNIDGYFKIYSKHTPINLVRDLPQGFSALEPLVDLPICINITRFQT
OU290035/B.1.617.1/Kappa   GKQGNFKNLREFVFKNIDGYFKIYSKHTPINLVRDLPHGFSALEPLVDLPIGINITRFQT
OU265178/B.1.617.1/Kappa   GKQGNFKNLREFVFKNIDGYFKIYSKHTPINLVRDLPHGFSALEPLVDLPIGINITRFQT
OU254949/B.1.617.1/Kappa   GKQGNFKNLREFVFKNIDGYFKIYSKHTPINLVRDLPHGFSALEPLVDLPIGINITRFQT
OU247018/B.1.617.1/Kappa   GKQGNFKNLREFVFKNIDGYFKIYSKHTPINLVRDLPQGFSALEPLVDLPIGINITRFQT
OU061269/B.1.617.1/Kappa   GKQGNFKNLREFVFKNIDGYFKIYSKHTPINLVRDLPQGFSALEPLVDLPIGINITRFQT
OU182966/B.1.617.1/Kappa   GKQGNFKNLREFVFKNIDGYFKIYSKHTPINLVRDLPQGFSALEPLVDLPIGINITRFQT
MZ746235/B.1.617.1/Kappa   GKQGNFKNLREFVFKNIDGYFKIYSKHTPINLVRDLPQGFSALEPLVDLPIGINITRFQT
MZ487800/B.1.617.1/Kappa   GKQGNFKNLREFVFKNIDGYFKIYSKHTPINLVRDLPQGFSALEPLVDLPIGINITRFQT
OU322235/B.1.617.1/Kappa   GKQGNFKNLREFVFKNIDGYFKIYSKHTPINLVRDLPQGFSALEPLVDLPIGINITRFQT
MZ953185/C.37/Lambda       GKQGNFKNLREFVFKNIDGYFKIYSKHTPINLVRDLPQGFSALEPLVDLPIGINITRFQT
MZ937666/C.37/Lambda       GKQGNFKNLREFVFKNIDGYFKIYSKHTPINLVRDLPQGFSALEPLVDLPIGINITRFQT
OU356036/C.37/Lambda       GKQGNFKNLREFVFKNIDGYFKIYSKHTPINLVRDLPQGFSALEPLVDLPIGINITRFQT
OU351967/C.37/Lambda       GKQGNFKNLREFVFKNIDGYFKIYSKHTPINLVRDLPQGFSALEPLVDLPIGINITRFQT
OU053018/C.37/Lambda       GKQGNFKNLREFVFKNIDGYFKIYSKHTPINLVRDLPQGFSALEPLVDLPIGINITRFQT
OU268528/C.37/Lambda       GKQGNFKNLREFVFKNIDGYFKIYSKHTPINLVRDLPQGFSALEPLVDLPIGINITRFQT
OU100999/C.37/Lambda       GKQGNFKNLREFVFKNIDGYFKIYSKHTPINLVRDLPQGFSALEPLVDLPIGINITRFQT
MZ275295/C.37/Lambda       GKQGNFKNLREFVFKNIDGYFKIYSKHTPINLVRDLPQGFSALEPLVDLPIGINITRFQT
MZ275301/C.37/Lambda       GKQGNFKNLREFVFKNIDGYFKIYSKHTPINLVRDLPQGFSALEPLVDLPIGINITRFQT
MZ908711/C.37/Lambda       GKQGNFKNLREFVFKNIDGYFKIYSKHTPINLVRDLPQGFSALEPLVDLPIGINITRFQT
MZ496613/C.37/Lambda       GKQGNFKNLREFVFKNIDGYFKIYSKHTPINLVRDLPQGFSALEPLVDLPIGINITRFQT
OU517038/C.37/Lambda       GKQGNFKNLREFVFKNIDGYFKIYSKHTPINLVRDLPQGFSALEPLVDLPIGINITRFQT
MZ953277/C.37/Lambda       GKQGNFKNLREFVFKNIDGYFKIYSKHTPINLVRDLPQGFSALEPLVDLPIGINITRFQT
MZ942542/C.37/Lambda       GKQGNFKNLREFVFKNIDGYFKIYSKHTPINLVRDLPQGFSALEPLVDLPIGINITRFQT
MZ857990/C.37/Lambda       GKQGNFKNLREFVFKNIDGYFKIYSKHTPINLVRDLPQGFSALEPLVDLPIGINITRFQT
```

```
                                     250       260       270       280       290       300
MN908947/Wuhan-Hu-1           LLALHRSYLTPGDSSSGWTAGAAAYYVGYLQPRTFLLKYNENGTITDAVDCALDPLSETK
BS001137/B.1.1.7/Alpha        LLALHRSYLTPGDSSSGWTAGAAAYYVGYLQPRTFLLKYNENGTITDAVDCALDPLSETK
BS001138/B.1.1.7/Alpha        LLALHRSYLTPGDSSSGWTAGAAAYYVGYLQPRTFLLKYNENGTITDAVDCALDPLSETK
BS001139/B.1.1.7/Alpha        LLALHRSYLTPGDSSSGWTAGAAAYYVGYLQPRTFLLKYNENGTITDAVDCALDPLSETK
BS001140/B.1.1.7/Alpha        LLALHRSYLTPGDSSSGWTAGAAAYYVGYLQPRTFLLKYNENGTITDAVDCALDPLSETK
MW913362/B.1.1.7/Alpha        LLALHRSYLTPGDSSSGWTAGAAAYYVGYLQPRTFLLKYNENGTITDAVDCALDPLSETK
MZ266365/B.1.1.7/Alpha        LLALHRSYLTPGDSSSGWTAGAAAYYVGYLQPRTFLLKYNENGTITDAVDCALDPLSETK
MZ350105/B.1.1.7/Alpha        LLALHRSYLTPGDSSSGWTAGAAAYYVGYLQPRTFLLKYNENGTITDAVDCALDPLSETK
MZ350109/B.1.1.7/Alpha        LLALHRSYLTPGDSSSGWTAGAAAYYVGYLQPRTFLLKYNENGTITDAVDCALDPLSETK
MZ888516/B.1.1.7/Alpha        LLALHRSYLTPGDSSSGWTAGAAAYYVGYLQPRTFLLKYNENGTITDAVDCALDPLSETK
OU562607/B.1.1.7/Alpha        LLALHRSYLTPGDSSSGWTAGAAAYYVGYLQPRTFLLKYNENGTITDAVDCALDPLSETK
OU514166/B.1.1.7/Alpha        LLALHRSYLTPGDSSSGWTAGAAAYYVGYLQPRTFLLKYNENGTITDAVDCALDPLSETK
OU538851/B.1.1.7/Alpha        LLALHSSYLTPGDSSSGWTAGAAAYYVGYLQPRTFLLKYNENGTITDAVDCALDPLSETK
OU538849/B.1.1.7/Alpha        LLALHRSYLTPGDSSSGWTAGAAAYYVGYLQPRTFLLKYNENGTITDAVDCALDPLSETK
MZ895877/B.1.1.7/Alpha        LLALHRSYLTPGDSSSGWTAGAAAYYVGYLQPRTFLLKYNENGTITDAVDCALDPLSETK
OU514176/B.1.1.7/Alpha        LLALHRSYLTPGDSSSGWTAGAAAYYVGYLQPRTFLLKYNENGTITDAVDCALDPLSETK
OU562608/B.1.1.7/Alpha        LLALHRSYLTPGDSSSGWTAGAAAYYVGYLQPRTFLLKYNENGTITDAVDCALDPLSETK
MW981442/B.1.351/Beta         L...HRSYLTPGDSSSGWTAGAAAYYVGYLQPRTFLLKYNENGTITDAVDCALDPLSETK
MZ298839/B.1.351/Beta         L...HRSYLTPGDSSSGWTAGAAAYYVGYLQPRTFLLKYNENGTITDAVDCALDPLSETK
MZ913100/B.1.351/Beta         L...HRSYLTPGDSSSGWTAGAAAYYVGYLQPRTFLLKYNENGTITDAVDCALDPLSETK
MZ879365/B.1.351/Beta         L...HRSYLTPGDSSSGWTAGAAAYYVGYLQPRTFLLKYNENGTITDAVDCALDPLSETK
OU525106/B.1.351/Beta         L...HRSYLTPGDSSSGWTAGAAAYYVGYLQPRTFLLKYNENGTITDAVDCALDPLSETK
OU539352/B.1.351/Beta         L...HRSYLTPGDSSSGWTAGAAAYYVGYLQPRTFLLKYNENGTITDAVDCALDPLSETK
MZ202314/B.1.351/Beta         L...HRSYLTPGDSSSGWTAGAAAYYVGYLQPRTFLLKYNENGTITDAVDCALDPLSETK
MZ298840/B.1.351/Beta         L...HRSYLTPGDSSSGWTAGAAAYYVGYLQPRTFLLKYNENGTITDAVDCALDPLSETK
MZ376663/B.1.351/Beta         L...HRSYLTPGDSSSGWTAGAAAYYVGYLQPRTFLLKYNENGTITDAVDCALDPLSETK
LC643036/B.1.617.2/Delta      LLALHRSYLTPGDSSSGWTAGAAAYYVGYLQPRTFLLKYNENGTITDAVDCALDPLSETK
LC643044/B.1.617.2/Delta      LLALHRSYLTPGDSSSGWTAGAAAYYVGYLQPRTFLLKYNENGTITDAVDCALDPLSETK
MZ853946/B.1.617.2/Delta      LLALHRSYLTPGDSSSGWTAGAAAYYVGYLQPRTFLLKYNENGTITDAVDCALDPLSETK
MZ854386/B.1.617.2/Delta      LLALHRSYLTPGDSSSGWTAGAAAYYVGYLQPRTFLLKYNENGTITDAVDCALDPLSETK
MZ888532/B.1.617.2/Delta      LLALHRSYLTPGDSSSGWTAGAAAYYVGYLQPRTFLLKYNENGTITDAVDCALDPLSETK
MZ888533/B.1.617.2/Delta      LLALHRSYLTPGDSSSGWTAGAAAYYVGYLQPRTFLLKYNENGTITDAVDCALDPLSETK
MZ896136/B.1.617.2/Delta      LLALHRSYLTPGDSSSGWTAGAAAYYVGYLQPRTFLLKYNENGTITDAIDCALDPLSETK
OU539261/B.1.617.2/Delta      LLALHRSYLTPGDSSSGWTAGAAAYYVGYLQPRTFLLKYNENGTITDAVDCALDPLSETK
MZ854389/B.1.617.2/Delta      LLALHRSYLTPGDSSSGWTAGAAAYYVGYLQPRTFLLKYNENGTITDAVDCALDPLSETK
OU562629/B.1.617.2/Delta      LLALHRSYLTPGDSSSGWTAGAAAYYVGYLQPRTFLLKYNENGTITDAVDCALDPLSETK
OU539690/B.1.617.2/Delta      LLALHRSYLTPGDSSSGWTAGAAAYYVGYLQPRTFLLKYNENGTITDAVDCALDPLSETK
MZ854387/B.1.617.2/Delta      LLALHRSYLTPGDSSSGWTAGAAAYYVGYLQPRTFLLKYNENGTITDAVDCALDPLSETK
OU539784/P.1/Gamma            LLALHRSYLTPGDSSSGWTAGAAAYYVGYLQPRTFLLKYNENGTITDAVDCALDPLSETK
OU539830/P.1/Gamma            LLALHRSYLTPGDSSSGWTAGAAAYYVGYLQPRTFLLKYNENGTITDAVDCALDPLSETK
OU539894/P.1/Gamma            LLALHRSYLTPGDSSSGWTAGAAAYYVGYLQPRTFLLKYNENGTITDAVDCALDPLSETK
MZ477748/P.1/Gamma            LLALHRSYLTPGDSSSGWTASAAAYYVGYLQPRTFLLKYNENGTITDAVDCALDPLSETK
MZ477746/P.1/Gamma            LLALHRSYLTPGDSSSGWTAGAAAYYVGYLQPRTFLLKYNENGTITDAVDCALDPLSETK
MW938104/P.1/Gamma            LLALHRSYLTPGDSSSGWTAGAAAYYVGYLQPRTFLLKYNENGTITDAVDCALDPLSETK
MZ896179/P.1/Gamma            LLALHRSYLTPGDSSSGWTAGAAAYYVGYLQPRTFLLKYNENGTITDAVDCALDPLSETK
MZ896359/P.1/Gamma            LLALHRSYLTPGDSSSGWTAGAAAYYVGYLQPRTFLLKYNENGTITDAVDCALDPLSETK
MZ896669/P.1/Gamma            LLALHRSYLTPGDSSSGWTAGAAAYYVGYLQPRTFLLKYNENGTITDAVDCALDPLSETK
OU565182/P.1/Gamma            LLALHRSYLTPGDSSSGWTAGAAAYYVGYLQPRTFLLKYNENGTITDAVDCALDPLSETK
OU492255/P.1/Gamma            LLALHRSYLTPGDSSSGWTAGAAAYYVGYLQPRTFLLKYNENGTITDAVDCALDPLSETK
MZ911221/B.1.427/Epsilon      LLALHRSYLTPGDSSSGWTAGAAAYYVGYLQPRTFLLKYNENGTITDAVDCALDPLSETK
MZ375854/B.1.427/Epsilon      LLALHRSYLTPGDSSSGLTAGAAAYYVGYLQPRTFLLKYNENGTITDAVDCALDPLSETK
MZ375858/B.1.427/Epsilon      LLALHRSYLTPGDSSSGLTAGAAAYYVGYLQPRTFLLKYNENGTITDAVDCALDPLSETK
MZ943834/B.1.429/Epsilon      LLALHRSYLTPGDSSSGWTAGAAAYYVGYLQPRTFLLKYNENGTITDAVDCALDPLSETK
MZ277390/B.1.429/Epsilon      LLALHRSYLTPGDSSSGWTAGAAAYYVGYLQPRTFLLKYNENGTITDAVDCALDPLSETK
OU282717/B.1.429/Epsilon      LLALHRSYLTPGDSSSGWTAGAAAYYVGYLQPRTFLLKYNENGTITDAVDCALDPLSETK
OU235436/B.1.429/Epsilon      LLALHRSYLTPGDSSSGWTAGAAAYYVGYLQPRTFLLKYNENGTITDAVDCALDPLSETK
MZ687447/B.1.429/Epsilon      LLALHRSYLTPGDSSSGWTAGAAAYYVGYLQPRTFLLKYNENGTITDAVDCALDPLSETK
MZ538882/B.1.617.1/Kappa      LLALHRSYLTPGDSSSGWTAGAAAYYVGYLQPRTFLLKYNENGTITDAVDCALDPLSETK
MZ724425/B.1.617.1/Kappa      LLALHRSYLTPGDSSSGWTAGAAAYYVGYLQPRTFLLKYNENGTITDAVDCALDPLSETK
MZ363839/B.1.617.1/Kappa      LLALHRSYLTPGDSSSGWTAGAAAYYVGYLQPRTFLLKYNENGTITDAVDCALDPLSETK
MZ332524/B.1.617.1/Kappa      LLALHRSYLTPGDSSSGWTAGAAAYYVGYLQPRTFLLKYNENGTITDAVDCALDPLSETK
MZ332527/B.1.617.1/Kappa      LLALHRSYLTPGDSSSGWTAGAAAYYVGYLQPRTFLLKYNENGTITDAVDCALDPLSETK
LC633761/B.1.617.1/Kappa      LLALHRSYLTPGDSSSGWTAGAAAYYVGYLQPRTFLLKYNENGTITDAVDCALDPLSETK
MZ562746/B.1.617.1/Kappa      LLALHRSYLTPGDSSSGWTAGAAAYYVGYLQPRTFLLKYNENGTITDAVDCALDPLSETK
MZ571142/B.1.617.1/Kappa      LLALHRSYLTPGDSSSGWTAGAAAYYVGYLQPRTFLLKYNENGTITDAVDCALDPLSETK
OU168413/B.1.617.1/Kappa      LLALHRSYLTPGDSSSGWTAGAAAYYVGYLQPRTFLLKYNENGTITDAVDCALDPLSETK
MZ026886/B.1.617.1/Kappa      LLALHRSYLTPGDSSSGWTAGAAAYYVGYLQPRTFLLKYNENGTITDAVDCALDPLSETK
OU172184/B.1.617.1/Kappa      LLALHRSYLTPGDSSSGWTAGAAAYYVGYLQPRTFLLKYNENGTITDAVDCALDPLSETK
MZ397173/B.1.617.1/Kappa      LLALHRSYLTPGDSSSGWTAGAAAYYVGYLQPRTFLLKYNENGTITDAVDCALDPLSETK
MZ397176/B.1.617.1/Kappa      LLALHRSYLTPGDSSSGWTAGAAAYYVGYLQPRTFLLKYNENGTITDAVDCALDPLSETK
MZ397175/B.1.617.1/Kappa      LLALHRSYLTPGDSSSGWTAGAAAYYVGYLQPRTFLLKYNENGTITDAVDCALDPLSETK
MZ397177/B.1.617.1/Kappa      LLALHRSYLTPGDSSSGWTAGAAAYYVGYLQPRTFLLKYNENGTITDAVDCALDPLSETK
OU290035/B.1.617.1/Kappa      LLALHRSYLTPGDSSSGWTAGAAAYYVGYLQPRTFLLKYNENGTITDAVDCALDPLSETK
OU265178/B.1.617.1/Kappa      LLALHRSYLTPGDSSSGWTAGAAAYYVGYLQPRTFLLKYNENGTITDAVDCALDPLSETK
OU254949/B.1.617.1/Kappa      LLALHRSYLTPGDSSSGWTAGAAAYYVGYLQPRTFLLKYNENGTITDAVDCALDPLSETK
OU247018/B.1.617.1/Kappa      LLALHRSYLTPGDSSSGWTAGAAAYYVGYLQPRTFLLKYNENGTITDAVDCALDPLSETK
OU061269/B.1.617.1/Kappa      LLALHRSYLTPGDSSSGWTAGAAAYYVGYLQPRTFLLKYNENGTITDAVDCALDPLSETK
OU182966/B.1.617.1/Kappa      LLALHRSYLTPGDSSSGWTAGAAAYYVGYLQPRTFLLKYNENGTITDAVDCALDPLSETK
MZ746235/B.1.617.1/Kappa      LLALHRSYLTPGDSSSGWTAGAAAYYVGYLQPRTFLLKYNENGTITDAVDCALDPLSETK
MZ487800/B.1.617.1/Kappa      LLALHRSYLTPGDSSSGWTAGAAAYYVGYLQPRTFLLKYNENGTITDAVDCALDPLSETK
OU322235/B.1.617.1/Kappa      LLALHRSYLTPGDSSSGWTAGAAAYYVGYLQPRTFLLKYNENGTITDAVDCALDPLSETK
MZ953185/C.37/Lambda          LLALHN.......SSSGWTAGAAAYYVGYLQPRTFLLKYNENGTITDAVDCALDPLSETK
MZ937666/C.37/Lambda          LLALHN.......SSSGWTAGAAAYYVGYLQPRTFLLKYNENGTITDAVDCALDPLSETK
OU356036/C.37/Lambda          LLALHN.......SSSGWTAGAAAYYVGYLQPRTFLLKYNENGTITDAVDCALDPLSETK
OU351967/C.37/Lambda          LLALHN.......SSSGWTAGAAAYYVGYLQPRTFLLKYNENGTITDAVDCALDPLSETK
OU053018/C.37/Lambda          LLALHN.......SSSGWTAGAAAYYVGYLQPRTFLLKYNENGTITDAVDCALDPLSETK
OU268528/C.37/Lambda          LLALHN.......SSSGWTAGAAAYYVGYLQPRTFLLKYNENGTITDAVDCALDPLSETK
OU100999/C.37/Lambda          LLALHN.......SSSGWTAGAAAYYVGYLQPRTFLLKYNENGTITDAVDCALDPLSETK
MZ275295/C.37/Lambda          LLALHN.......SSSGWTAGAAAYYVGYLQPRTFLLKYNENGTITDAVDCALDPLSETK
MZ275301/C.37/Lambda          LLALHN.......SSSGWTAGAAAYYVGYLQPRTFLLKYNENGTITDAVDCALDPLSETK
MZ908711/C.37/Lambda          LLALHN.......SSSGWTAGAAAYYVGYLQPRTFLLKYNENGTITDAVDCALDPLSETK
MZ496613/C.37/Lambda          LLALHN.......SSSGWTAGAAAYYVGYLQPRTFLLKYNENGTITDAVDCALDPLSETK
OU517038/C.37/Lambda          LLALHN.......SSSGWTAGAAAYYVGYLQPRTFLLKYNENGTITDAVDCALDPLSETK
MZ953277/C.37/Lambda          LLALHN.......SSSGWTAGAAAYYVGYLQPRTFLLKYNENGTITDAVDCALDPLSETK
MZ942542/C.37/Lambda          LLALHN.......SSSGWTAGAAAYYVGYLQPRTFLLKYNENGTITDAVDCALDPLSETK
MZ857990/C.37/Lambda          LLALHN.......SSSGWTAGAAAYYVGYLQPRTFLLKYNENGTITDAVDCALDPLSETK
```

```
                                   310       320       330       340       350       360
MN908947/Wuhan-Hu-1          CTLKSFTVEKGIYQTSNFRVQPTESIVRFPNITNLCPFGEVFNATRFASVYAWNRKRISN
BS001137/B.1.1.7/Alpha       CTLKSFTVEKGIYQTSNFRVQPTESIVRFPNITNLCPFGEVFNATRFASVYAWNRKRISN
BS001138/B.1.1.7/Alpha       CTLKSFTVEKGIYQTSNFRVQPTESIVRFPNITNLCPFGEVFNATRFASVYAWNRKRISN
BS001139/B.1.1.7/Alpha       CTLKSFTVEKGIYQTSNFRVQPTESIVRFPNITNLCPFGEVFNATRFASVYAWNRKRISN
BS001140/B.1.1.7/Alpha       CTLKSFTVEKGIYQTSNFRVQPTESIVRFPNITNLCPFGEVFNATRFASVYAWNRKRISN
MW913362/B.1.1.7/Alpha       CTLKSFTVEKGIYQTSNFRVQPTESIVRFPNITNLCPFGEVFNATRFASVYAWNRKRISN
MZ266365/B.1.1.7/Alpha       CTLKSFTVEKGIYQTSNFRVQPTESIVRFPNITNLCPFGEVFNATRFASVYAWNRKRISN
MZ350105/B.1.1.7/Alpha       CTLKSFTVEKGIYQTSNFRVQPTESIVRFPNITNLCPFGEVFNATRFASVYAWNRKRISN
MZ350109/B.1.1.7/Alpha       CTLKSFTVEKGIYQTSNFRVQPTESIVRFPNITNLCPFGEVFNATRFASVYAWNRKRISN
MZ888516/B.1.1.7/Alpha       CTLKSFTVEKGIYQTSNFRVQPTESIVRFPNITNLCPFGEVFNATRFASVYAWNRKRISN
OU562607/B.1.1.7/Alpha       CTLKSFTVEKGIYQTSNFRVQPTESIVRFPNITNLCPFGEVFNATRFASVYAWNRKRISN
OU514166/B.1.1.7/Alpha       CTLKSFTVEKGIYQTSNFRVQPTESIVRFPNITNLCPFGEVFNATRFASVYAWNRKRISN
OU538851/B.1.1.7/Alpha       CTLKSFTVEKGIYQTSNFRVQPTESIVRFPNITNLCPFGEVFNATRFASVYAWNRKRISN
OU538849/B.1.1.7/Alpha       CTLKSFTVEKGIYQTSNFRVQPTESIVRFPNITNLCPFGEVFNATRFASVYAWNRKRISN
MZ895877/B.1.1.7/Alpha       CTLKSFTVEKGIYQTSNFRVQPTESIVRFPNITNLCPFGEVFNATRFASVYAWNRKRISN
OU514176/B.1.1.7/Alpha       CTLKSFTVEKGIYQTSNFRVQPTESIVRFPNITNLCPFGEVFNATRFASVYAWNRKRISN
OU562608/B.1.1.7/Alpha       CTLKSFTVEKGIYQTSNFRVQPTESIVRFPNITNLCPFGEVFNATRFASVYAWNRKRISN
MW981442/B.1.351/Beta        CTLKSFTVEKGIYQTSNFRVQPTESIVRFPNITNLCPFGEVFNATRFASVYAWNRKRISN
MZ298839/B.1.351/Beta        CTLKSFTVEKGIYQTSNFRVQPTESIVRFPNITNLCPFGEVFNATRFASVYAWNRKRISN
MZ913100/B.1.351/Beta        CTLKSFTVEKGIYQTSNFRVQPTESIVRFPNITNLCPFGEVFNATRFASVYAWNRKRISN
MZ879365/B.1.351/Beta        CTLKSFTVEKGIYQTSNFRVQPTESIVRFPNITNLCPFGEVFNATRFASVYAWNRKRISN
OU525106/B.1.351/Beta        CTLKSFTVEKGIYQTSNFRVQPTESIVRFPNITNLCPFGEVFNATRFASVYAWNRKRISN
OU539352/B.1.351/Beta        CTLKSFTVEKGIYQTSNFRVQPTESIVRFPNITNLCPFGEVFNATRFASVYAWNRKRISN
MZ202314/B.1.351/Beta        CTLKSFTVEKGIYQTSNFRVQPTESIVRFPNITNLCPFGEVFNATRFASVYAWNRKRISN
MZ298840/B.1.351/Beta        CTLKSFTVEKGIYQTSNFRVQPTESIVRFPNITNLCPFGEVFNATRFASVYAWNRKRISN
MZ376663/B.1.351/Beta        CTLKSFTVEKGIYQTSNFRVQPTESIVRFPNITNLCPFGEVFNATRFASVYAWNRKRISN
LC643036/B.1.617.2/Delta     CTLKSFTVEKGIYQTSNFRVQPTESIVRFPNITNLCPFGEVFNATRFASVYAWNRKRISN
LC643044/B.1.617.2/Delta     CTLKSFTVEKGIYQTSNFRVQPTESIVRFPNITNLCPFGEVFNATRFASVYAWNRKRISN
MZ853946/B.1.617.2/Delta     CTLKSFTVEKGIYQTSNFRVQPTESIVRFPNITNLCPFGEVFNATRFASVYAWNRKRISN
MZ854386/B.1.617.2/Delta     CTLKSFTVEKGIYQTSNFRVQPTESIVRFPNITNLCPFGEVFNATRFASVYAWNRKRISN
MZ888532/B.1.617.2/Delta     CTLKSFTVEKGIYQTSNFRVQPTESIVRFPNITNLCPFGEVFNATRFASVYAWNRKRISN
MZ888533/B.1.617.2/Delta     CTLKSFTVEKGIYQTSNFRVQPTESIVRFPNITNLCPFGEVFNATRFASVYAWNRKRISN
MZ896136/B.1.617.2/Delta     CTLKSFTVEKGIYQTSNFRVQPTESIVRFPNITNLCPFGEVFNATRFASVYAWNRKRISN
OU539261/B.1.617.2/Delta     CTLKSFTVEKGIYQTSNFRVQPTESIVRFPNITNLCPFGEVFNATRFASVYAWNRKRISN
MZ854389/B.1.617.2/Delta     CTLKSFTVEKGIYQTSNFRVQPTESIVRFPNITNLCPFGEVFNATRFASVYAWNRKRISN
OU562629/B.1.617.2/Delta     CTLKSFTVEKGIYQTSNFRVQPTESIVRFPNITNLCPFGEVFNATRFASVYAWNRKRISN
OU539690/B.1.617.2/Delta     CTLKSFTVEKGIYQTSNFRVQPTESIVRFPNITNLCPFGEVFNATRFASVYAWNRKRISN
MZ854387/B.1.617.2/Delta     CTLKSFTVEKGIYQTSNFRVQPTESIVRFPNITNLCPFGEVFNATRFASVYAWNRKRISN
OU539784/P.1/Gamma           CTLKSFTVEKGIYQTSNFRVQPTESIVRFPNITNLCPFGEVFNATRFASVYAWNRKRISN
OU539830/P.1/Gamma           CTLKSFTVEKGIYQTSNFRVQPTESIVRFPNITNLCPFGEVFNATRFASVYAWNRKRISN
OU539894/P.1/Gamma           CTLKSFTVEKGIYQTSNFRVQPTESIVRFPNITNLCPFGEVFNATRFASVYAWNRKRISN
MZ477748/P.1/Gamma           CTLKSFTVEKGIYQTSNFRVQPTESIVRFPNITNLCPFGEVFNATRFASVYAWNRKRISN
MZ477746/P.1/Gamma           CTLKSFTVEKGIYQTSNFRVQPTESIVRFPNITNLCPFGEVFNATRFASVYAWNRKRISN
MW938104/P.1/Gamma           CTLKSFTVEKGIYQTSNFRVQPTESIVRFPNITNLCPFGEVFNATRFASVYAWNRKRISN
MZ896179/P.1/Gamma           CTLKSFTVEKGIYQTSNFRVQPTESIVRFPNITNLCPFGEVFNATRFASVYAWNRKRISN
MZ896359/P.1/Gamma           CTLKSFTVEKGIYQTSNFRVQPTESIVRFPNITNLCPFGEVFNATRFASVYAWNRKRISN
MZ896669/P.1/Gamma           CTLKSFTVEKGIYQTSNFRVQPTESIVRFPNITNLCPFGEVFNATRFASVYAWNRKRISN
OU565182/P.1/Gamma           CTLKSFTVEKGIYQTSNFRVQPTESIVRFPNITNLCPFGEVFNATRFASVYAWNRKRISN
OU492255/P.1/Gamma           CTLKSFTVEKGIYQTSNFRVQPTESIVRFPNITNLCPFGEVFNATRFASVYAWNRKRISN
MZ911221/B.1.427/Epsilon     CTLKSFTVEKGIYQTSNFRVQPTESIVRFPNITNLCPFGEVFNATRFASVYAWNRKRISN
MZ375854/B.1.427/Epsilon     CTLKSFTVEKGIYQTSNFRVQPTESIVRFPNITNLCPFGEVFNATRFASVYAWNRKRISN
MZ375858/B.1.427/Epsilon     CTLKSFTVEKGIYQTSNFRVQPTESIVRFPNITNLCPFGEVFNATRFASVYAWNRKRISN
MZ943834/B.1.429/Epsilon     CTLKSFTVEKGIYQTSNFRVQPTESIVRFPNITNLCPFGEVFNATRFASVYAWNRKRISN
MZ277390/B.1.429/Epsilon     CTLKSFTVEKGIYQTSNFRVQPTESIVRFPNITNLCPFGEVFNATRFASVYAWNRKRISN
OU282717/B.1.429/Epsilon     CTLKSFTVEKGIYQTSNFRVQPTESIVRFPNITNLCPFGEVFNATRFASVYAWNRKRISN
OU235436/B.1.429/Epsilon     CTLKSFTVEKGIYQTSNFRVQPTESIVRFPNITNLCPFGEVFNATRFASVYAWNRKRISN
MZ687447/B.1.429/Epsilon     CTLKSFTVEKGIYQTSNFRVQPTESIVRFPNITNLCPFGEVFNATRFASVYAWNRKRISN
MZ538882/B.1.617.1/Kappa     CTLKSFTVEKGIYQTSNFRVQPTESIVRFPNITNLCPFGEVFNATRFASVYAWNRKRISN
MZ724425/B.1.617.1/Kappa     CTLKSFTVEKGIYQTSNFRVQPTESIVRFPNITNLCPFGEVFNATRFASVYAWNRKRISN
MZ363839/B.1.617.1/Kappa     CTLKSFTVEKGIYQTSNFRVQPTESIVRFPNITNLCPFGEVFNATRFASVYAWNRKRISN
MZ332524/B.1.617.1/Kappa     CTLKSFTVEKGIYQTSNFRVQPTESIVRFPNITNLCPFGEVFNATRFASVYAWNRKRISN
MZ332527/B.1.617.1/Kappa     CTLKSFTVEKGIYQTSNFRVQPTESIVRFPNITNLCPFGEVFNATRFASVYAWNRKRISN
LC633761/B.1.617.1/Kappa     CTLKSFTVEKGIYQTSNFRVQPTESIVRFPNITNLCPFGEVFNATRFASVYAWNRKRISN
MZ562746/B.1.617.1/Kappa     CTLKSFTVEKGIYQTSNFRVQPTESIVRFPNITNLCPFGEVFNATRFASVYAWNRKRISN
MZ571142/B.1.617.1/Kappa     CTLKSFTVEKGIYQTSNFRVQPTESIVRFPNITNLCPFGEVFNATRFASVYAWNRKRISN
OU168413/B.1.617.1/Kappa     CTLKSFTVEKGIYQTSNFRVQPTESIVRFPNITNLCPFGEVFNATRFASVYAWNRKRISN
MZ026886/B.1.617.1/Kappa     CTLKSFTVEKGIYQTSNFRVQPTESIVRFPNITNLCPFGEVFNATRFASVYAWNRKRISN
OU172184/B.1.617.1/Kappa     CTLKSFTVEKGIYQTSNFRVQPTESIVRFPNITNLCPFGEVFNATRFASVYAWNRKRISN
MZ397173/B.1.617.1/Kappa     CTLKSFTVEKGIYQTSNFRVQPTESIVRFPNITNLCPFGEVFNATRFASVYAWNRKRISN
MZ397176/B.1.617.1/Kappa     CTLKSFTVEKGIYQTSNFRVQPTESIVRFPNITNLCPFGEVFNATRFASVYAWNRKRISN
MZ397175/B.1.617.1/Kappa     CTLKSFTVEKGIYQTSNFRVQPTESIVRFPNITNLCPFGEVFNATRFASVYAWNRKRISN
MZ397177/B.1.617.1/Kappa     CTLKSFTVEKGIYQTSNFRVQPTESIVRFPNITNLCPFGEVFNATRFASVYAWNRKRISN
OU290035/B.1.617.1/Kappa     CTLKSFTVEKGIYQTSNFRVQPTESIVRFPNITNLCPFGEVFNATRFASVYAWNRKRISN
OU265178/B.1.617.1/Kappa     CTLKSFTVEKGIYQTSNFRVQPTESIVRFPNITNLCPFGEVFNATRFASVYAWNRKRISN
OU254949/B.1.617.1/Kappa     CTLKSFTVEKGIYQTSNFRVQPTESIVRFPNITNLCPFGEVFNATRFASVYAWNRKRISN
OU247018/B.1.617.1/Kappa     CTLKSFTVEKGIYQTSNFRVQPTESIVRFPNITNLCPFGEVFNATRFASVYAWNRKRISN
OU061269/B.1.617.1/Kappa     CTLKSFTVEKGIYQTSNFRVQPTESIVRFPNITNLCPFGEVFNATRFASVYAWNRKRISN
OU182966/B.1.617.1/Kappa     CTLKSFTVEKGIYQTSNFRVQPTESIVRFPNITNLCPFGEVFNATRFASVYAWNRKRISN
MZ746235/B.1.617.1/Kappa     CTLKSFTVEKGIYQTSNFRVQPTESIVRFPNITNLCPFGEVFNATRFASVYAWNRKRISN
MZ487800/B.1.617.1/Kappa     CTLKSFTVEKGIYQTSNFRVQPTESIVRFPNITNLCPFGEVFNATRFASVYAWNRKRISN
OU322235/B.1.617.1/Kappa     CTLKSFTVEKGIYQTSNFRVQPTESIVRFPNITNLCPFGEVFNATRFASVYAWNRKRISN
MZ953185/C.37/Lambda         CTLKSFTVEKGIYQTSNFRVQPTESIVRFPNITNLCPFGEVFNATRFASVYAWNRKRISN
MZ937666/C.37/Lambda         CTLKSFTVEKGIYQTSNFRVQPTESIVRFPNITNLCPFGEVFNATRFASVYAWNRKRISN
OU356036/C.37/Lambda         CTLKSFTVEKGIYQTSNFRVQPTESIVRFPNITNLCPFGEVFNATRFASVYAWNRKRISN
OU351967/C.37/Lambda         CTLKSFTVEKGIYQTSNFRVQPTESIVRFPNITNLCPFGEVFNATRFASVYAWNRKRISN
OU053018/C.37/Lambda         CTLKSFTVEKGIYQTSNFRVQPTESIVRFPNITNLCPFGEVFNATRFASVYAWNRKRISN
OU268528/C.37/Lambda         CTLKSFTVEKGIYQTSNFRVQPTESIVRFPNITNLCPFGEVFNATRFASVYAWNRKRISN
OU100999/C.37/Lambda         CTLKSFTVEKGIYQTSNFRVQPTESIVRFPNITNLCPFGEVFNATRFASVYAWNRKRISN
MZ275295/C.37/Lambda         CTLKSFTVEKGIYQTSNFRVQPTESIVRFPNITNLCPFGEVFNATRFASVYAWNRKRISN
MZ275301/C.37/Lambda         CTLKSFTVEKGIYQTSNFRVQPTESIVRFPNITNLCPFGEVFNATRFASVYAWNRKRISN
MZ908711/C.37/Lambda         CTLKSFTVEKGIYQTSNFRVQPTESIVRFPNITNLCPFGEVFNATRFASVYAWNRKRISN
MZ496613/C.37/Lambda         CTLKSFTVEKGIYQTSNFRVQPTESIVRFPNITNLCPFGEVFNATRFASVYAWNRKRISN
OU517038/C.37/Lambda         CTLKSFTVEKGIYQTSNFRVQPTESIVRFPNITNLCPFGEVFNATRFASVYAWNRKRISN
MZ953277/C.37/Lambda         CTLKSFTVEKGIYQTSNFRVQPTESIVRFPNITNLCPFGEVFNATRFASVYAWNRKRISN
MZ942542/C.37/Lambda         CTLKSFTVEKGIYQTSNFRVQPTESIVRFPNITNLCPFGEVFNATRFASVYAWNRKRISN
MZ857990/C.37/Lambda         CTLKSFTVEKGIYQTSNFRVQPTESIVRFPNITNLCPFGEVFNATRFASVYAWNRKRISN
```

```
                                  370       380       390       400       410       420
                                    .         .         .         .         .         .
MN908947/Wuhan-Hu-1          CVADYSVLYNSASFSTFKCYGVSPTKLNDLCFTNVYADSFVIRGDEVRQIAPGQTGKIAD
BS001137/B.1.1.7/Alpha       CVADYSVLYNSASFSTFKCYGVSPTKLNDLCFTNVYADSFVIRGDEVRQIAPGQTGKIAD
BS001138/B.1.1.7/Alpha       CVADYSVLYNSASFSTFKCYGVSPTKLNDLCFTNVYADSFVIRGDEVRQIAPGQTGKIAD
BS001139/B.1.1.7/Alpha       CVADYSVLYNSASFSTFKCYGVSPTKLNDLCFTNVYADSFVIRGDEVRQIAPGQTGKIAD
BS001140/B.1.1.7/Alpha       CVADYSVLYNSASFSTFKCYGVSPTKLNDLCFTNVYADSFVIRGDEVRQIAPGQTGKIAD
MW913362/B.1.1.7/Alpha       CVADYSVLYNSASFSTFKCYGVSPTKLNDLCFTNVYADSFVIRGDEVRQIAPGQTGKIAD
MZ266365/B.1.1.7/Alpha       CVADYSVLYNSASFSTFKCYGVSPTKLNDLCFTNVYADSFVIRGDEVRQIAPGQTGKIAD
MZ350105/B.1.1.7/Alpha       CVADYSVLYNSASFSTFKCYGVSPTKLNDLCFTNVYADSFVIRGDEVRQIAPGQTGKIAD
MZ350109/B.1.1.7/Alpha       CVADYSVLYNSASFSTFKCYGVSPTKLNDLCFTNVYADSFVIRGDEVRQIAPGQTGKIAD
MZ888516/B.1.1.7/Alpha       CVADYSVLYNSASFSTFKCYGVSPTKLNDLCFTNVYADSFVIRGDEVRQIAPGQTGKIAD
OU562607/B.1.1.7/Alpha       CVADYSVLYNSASFSTFKCYGVSPTKLNDLCFTNVYADSFVIRGDEVRQIAPGQTGKIAD
OU514166/B.1.1.7/Alpha       CVADYSVLYNSASFSTFKCYGVSPTKLNDLCFTNVYADSFVIRGDEVRQIAPGQTGKIAD
OU538851/B.1.1.7/Alpha       CVADYSVLYNSASFSTFKCYGVSPTKLNDLCFTNVYADSFVIRGDEVRQIAPGQTGKIAD
OU538849/B.1.1.7/Alpha       CVADYSVLYNSASFSTFKCYGVSPTKLNDLCFTNVYADSFVIRGDEVRQIAPGQTGKIAD
MZ895877/B.1.1.7/Alpha       CVADYSVLYNSASFSTFKCYGVSPTKLNDLCFTNVYADSFVIRGDEVRQIAPGQTGKIAD
OU514176/B.1.1.7/Alpha       CVADYSVLYNSVSFSTFKCYGVSPTKLNDLCFTNVYADSFVIRGDEVRQIAPGQTGKIAD
OU562608/B.1.1.7/Alpha       CVADYSVLYNSASFSTFKCYGVSPTKLNDLCFTNVYADSFVIRGDEVRQIAPGQTGKIAD
MW981442/B.1.351/Beta        CVADYSVLYNSASFSTFKCYGVSPTKLNDLCFTNVYADSFVIRGDEVRQIAPGQTGKIAD
MZ298839/B.1.351/Beta        CVADYSVLYNSASFSTFKCYGVSPTKLNDLCFTNVYADSFVIRGDEVRQIAPGQTGNIAD
MZ913100/B.1.351/Beta        CVADYSVLYNSASFSTFKCYGVSPTKLNDLCFTNVYADSFVIRGDEVRQIAPGQTGNIAD
MZ879365/B.1.351/Beta        CVADYSVLYNSASFSTFKCYGVSPTKLNDLCFTNVYADSFVIRGDEVRQIAPGQTGNIAD
OU525106/B.1.351/Beta        CVADYSVLYNSASFSTFKCYGVSPTKLNDLCFTNVYADSFVIRGDEVRQIAPGQTGNIAD
OU539352/B.1.351/Beta        CVADYSVLYNSASFSTFKCYGVSPTKLNDLCFTNVYADSFVIRGDEVRQIAPGQTGNIAD
MZ202314/B.1.351/Beta        CVADYSVLYNSASFSTFKCYGVSPTKLNDLCFTNVYADSFVIRGDEVRQIAPGQTGNIAD
MZ298840/B.1.351/Beta        CVADYSVLYNSASFSTFKCYGVSPTKLNDLCFTNVYADSFVIRGDEVRQIAPGQTGNIAD
MZ376663/B.1.351/Beta        CVADYSVLYNSASFSTFKCYGVSPTKLNDLCFTNVYADSFVIRGDEVRQIAPGQTGKIAD
LC643036/B.1.617.2/Delta     CVADYSVLYNSASFSTFKCYGVSPTKLNDLCFTNVYADSFVIRGDEVRQIAPGQTGKIAD
LC643044/B.1.617.2/Delta     CVADYSVLYNSASFSTFKCYGVSPTKLNDLCFTNVYADSFVIRGDEVRQIAPGQTGKIAD
MZ853946/B.1.617.2/Delta     CVADYSVLYNSASFSTFKCYGVSPTKLNDLCFTNVYADSFVIRGDEVRQIAPGQTGKIAD
MZ854386/B.1.617.2/Delta     CVADYSVLYNSASFSTFKCYGVSPTKLNDLCFTNVYADSFVIRGDEVRQIAPGQTGKIAD
MZ888532/B.1.617.2/Delta     CVADYSVLYNSASFSTFKCYGVSPTKLNDLCFTNVYADSFVIRGDEVRQIAPGQTGKIAD
MZ888533/B.1.617.2/Delta     CVADYSVLYNSASFSTFKCYGVSPTKLNDLCFTNVYADSFVIRGDEVRQIAPGQTGKIAD
MZ896136/B.1.617.2/Delta     CVADYSVLYNSASFSTFKCYGVSPTKLNDLCFTNVYADSFVIRGDEVRQIAPGQTGKIAD
OU539261/B.1.617.2/Delta     CVADYSVLYNSASFSTFKCYGVSPTKLNDLCFTNVYADSFVIRGDEVRQIAPGQTGKIAD
MZ854389/B.1.617.2/Delta     CVADYSVLYNSASFSTFKCYGVSPTKLNDLCFTNVYADSFVIRGDEVRQIAPGQTGKIAD
OU562629/B.1.617.2/Delta     CVADYSVLYNSASFSTFKCYGVSPTKLNDLCFTNVYADSFVIRGDEVRQIAPGQTGKIAD
OU539690/B.1.617.2/Delta     CVADYSVLYNSASFSTFKCYGVSPTKLNDLCFTNVYADSFVIRGDEVRQIAPGQTGKIAD
MZ854387/B.1.617.2/Delta     CVADYSVLYNSASFSTFKCYGVSPTKLNDLCFTNVYADSFVIRGDEVRQIAPGQTGKIAD
OU539784/P.1/Gamma           CVADYSVLYNSASFSTFKCYGVSPTKLNDLCFTNVYADSFVIRGDEVRQIAPGQTGTIAD
OU539830/P.1/Gamma           CVADYSVLYNSASFSTFKCYGVSPTKLNDLCFTNVYADSFVIRGDEVRQIAPGQTGTIAD
OU539894/P.1/Gamma           CVADYSVLYNSASFSTFKCYGVSPTKLNDLCFTNVYADSFVIRGDEVRQIAPGQTGTIAD
MZ477748/P.1/Gamma           CVADYSVLYNSASFSTFKCYGVSPTKLNDLCFTNVYADSFVIRGDEVRQIAPGQTGTIAD
MZ477746/P.1/Gamma           CVADYSVLYNSASFSTFKCYGVSPTKLNDLCFTNVYADSFVIRGDEVRQIAPGQTGTIAD
MW938104/P.1/Gamma           CVADYSVLYNSASFSTFKCYGVSPTKLNDLCFTNVYADSFVIRGDEVRQIAPGQTGTIAD
MZ896179/P.1/Gamma           CVADYSVLYNSASFSTFKCYGVSPTKLNDLCFTNVYADSFVIRGDEVRQIAPGQTGTIAD
MZ896359/P.1/Gamma           CVADYSVLYNSASFSTFKCYGVSPTKLNDLCFTNVYADSFVIRGDEVRQIAPGQTGTIAD
MZ896669/P.1/Gamma           CVADYSVLYNSASFSTFKCYGVSPTKLNDLCFTNVYADSFVIRGDEVRQIAPGQTGTIAD
OU565182/P.1/Gamma           CVADYSVLYNSASFSTFKCYGVSPTKLNDLCFTNVYADSFVIRGDEVRQIAPGQTGTIAD
OU492255/P.1/Gamma           CVADYSVLYNSASFSTFKCYGVSPTKLNDLCFTNVYADSFVIRGDEVRQIAPGQTGTIAD
MZ911221/B.1.427/Epsilon     CVADYSVLYNSASFSTFKCYGVSPTKLNDLCFTNVYADSFVIRGDEVRQIAPGQTGKIAD
MZ375854/B.1.427/Epsilon     CVADYSVLYNSASFSTFKCYGVSPTKLNDLCFTNVYADSFVIRGDEVRQIAPGQTGKIAD
MZ375858/B.1.427/Epsilon     CVADYSVLYNSASFSTFKCYGVSPTKLNDLCFTNVYADSFVIRGDEVRQIAPGQTGKIAD
MZ943834/B.1.429/Epsilon     CVADYSVLYNSASFSTFKCYGVSPTKLNDLCFTNVYADSFVIRGDEVRQIAPGQTGKIAD
MZ277390/B.1.429/Epsilon     CVADYSVLYNSASFSTFKCYGVSPTKLNDLCFTNVYADSFVIRGDEVRQIAPGQTGKIAD
OU282717/B.1.429/Epsilon     CVADYSVLYNSASFSTFKCYGVSPTKLNDLCFTNVYADSFVIRGDEVRQIAPGQTGKIAD
OU235436/B.1.429/Epsilon     CVADYSVLYNSASFSTFKCYGVSPTKLNDLCFTNVYADSFVIRGDEVRQIAPGQTGKIAD
MZ687447/B.1.429/Epsilon     CVADYSVLYNSASFSTFKCYGVSPTKLNDLCFTNVYADSFVIRGDEVRQIAPGQTGKIAD
MZ538882/B.1.617.1/Kappa     CVADYSVLYNSASFSTFKCYGVSPTKLNDLCFTNVYADSFVIRGDEVRQIAPGQTGKIAD
MZ724425/B.1.617.1/Kappa     CVADYSVLYNSASFSTFKCYGVSPTKLNDLCFTNVYADSFVIRGDEVRQIAPGQTGKIAD
MZ363839/B.1.617.1/Kappa     CVADYSVLYNSASFSTFKCYGVSPTKLNDLCFTNVYADSFVIRGDEVRQIAPGQTGKIAD
MZ332524/B.1.617.1/Kappa     CVADYSVLYNSASFSTFKCYGVSPTKLNDLCFTNVYADSFVIRGDEVRQIAPGQTGKIAD
MZ332527/B.1.617.1/Kappa     CVADYSVLYNSASFSTFKCYGVSPTKLNDLCFTNVYADSFVIRGDEVRQIAPGQTGKIAD
LC633761/B.1.617.1/Kappa     CVADYSVLYNSASFSTFKCYGVSPTKLNDLCFTNVYADSFVIRGDEVRQIAPGQTGKIAD
MZ562746/B.1.617.1/Kappa     CVADYSVLYNSASFSTFKCYGVSPTKLNDLCFTNVYADSFVIRGDEVRQIAPGQTGKIAD
MZ571142/B.1.617.1/Kappa     CVADYSVLYNSASFSTFKCYGVSPTKLNDLCFTNVYADSFVIRGDEVRQIAPGQTGKIAD
OU168413/B.1.617.1/Kappa     CVADYSVLYNSASFSTFKCYGVSPTKLNDLCFTNVYADSFVIRGDEVRQIAPGQTGKIAD
MZ026886/B.1.617.1/Kappa     CVADYSVLYNSASFSTFKCYGVSPTKLNDLCFTNVYADSFVIRGDEVRQIAPGQTGKIAD
OU172184/B.1.617.1/Kappa     CVADYSVLYNSASFSTFKCYGVSPTKLNDLCFTNVYADSFVIRGDEVRQIAPGQTGKIAD
MZ397173/B.1.617.1/Kappa     CVADYSVLYNSASFSTFKCYGVSPTKLNDLCFTNVYADSFVIRGDEVRQIAPGQTGKIAD
MZ397176/B.1.617.1/Kappa     CVADYSVLYNSASFSTFKCYGVSPTKLNDLCFTNVYADSFVIRGDEVRQIAPGQTGKIAD
MZ397175/B.1.617.1/Kappa     CVADYSVLYNSASFSTFKCYGVSPTKLNDLCFTNVYADSFVIRGDEVRQIAPGQTGKIAD
MZ397177/B.1.617.1/Kappa     CVADYSVLYNSASFSTFKCYGVSPTKLNDLCFTNVYADSFVIRGDEVRQIAPGQTGKIAD
OU290035/B.1.617.1/Kappa     CVADYSVLYNSASFSTFKCYGVSPTKLNDLCFTNVYADSFVIRGDEVRQIAPGQTGKIAD
OU265178/B.1.617.1/Kappa     CVADYSVLYNSASFSTFKCYGVSPTKLNDLCFTNVYADSFVIRGDEVRQIAPGQTGKIAD
OU254949/B.1.617.1/Kappa     CVADYSVLYNSASFSTFKCYGVSPTKLNDLCFTNVYADSFVIRGDEVRQIAPGQTGKIAD
OU247018/B.1.617.1/Kappa     CVADYSVLYNSASFSTFKCYGVSPTKLNDLCFTNVYADSFVIRGDEVRQIAPGQTGKIAD
OU061269/B.1.617.1/Kappa     CVADYSVLYNSASFSTFKCYGVSPTKLNDLCFTNVYADSFVIRGDEVRQIAPGQTGKIAD
OU182966/B.1.617.1/Kappa     CVADYSVLYNSASFSTFKCYGVSPTKLNDLCFTNVYADSFVIRGDEVRQIAPGQTGKIAD
MZ746235/B.1.617.1/Kappa     CVADYSVLYNSASFSTFKCYGVSPTKLNDLCFTNVYADSFVIRGDEVRQIAPGQTGKIAD
MZ487800/B.1.617.1/Kappa     CVADYSVLYNSASFSTFKCYGVSPTKLNDLCFTNVYADSFVIRGDEVRQIAPGQTGKIAD
OU322235/B.1.617.1/Kappa     CVADYSVLYNSASFSTFKCYGVSPTKLNDLCFTNVYADSFVIRGDEVRQIAPGQTGKIAD
MZ953185/C.37/Lambda         CVADYSVLYNSASFSTFKCYGVSPTKLNDLCFTNVYADSFVIRGDEVRQIAPGQTGKIAD
MZ937666/C.37/Lambda         CVADYSVLYNSASFSTFKCYGVSPTKLNDLCFTNVYADSFVIRGDEVRQIAPGQTGKIAD
OU356036/C.37/Lambda         CVADYSVLYNSASFSTFKCYGVSPTKLNDLCFTNVYADSFVIRGDEVRQIAPGQTGKIAD
OU351967/C.37/Lambda         CVADYSVLYNSASFSTFKCYGVSPTKLNDLCFTNVYADSFVIRGDEVRQIAPGQTGKIAD
OU053018/C.37/Lambda         CVADYSVLYNSASFSTFKCYGVSPTKLNDLCFTNVYADSFVIRGDEVRQIAPGQTGKIAD
OU268528/C.37/Lambda         CVADYSVLYNSASFSTFKCYGVSPTKLNDLCFTNVYADSFVIRGDEVRQIAPGQTGKIAD
OU100999/C.37/Lambda         CVADYSVLYNSASFSTFKCYGVSPTKLNDLCFTNVYADSFVIRGDEVRQIAPGQTGKIAD
MZ275295/C.37/Lambda         CVADYSVLYNSASFSTFKCYGVSPTKLNDLCFTNVYADSFVIRGDEVRQIAPGQTGKIAD
MZ275301/C.37/Lambda         CVADYSVLYNSASFSTFKCYGVSPTKLNDLCFTNVYADSFVIRGDEVRQIAPGQTGKIAD
MZ908711/C.37/Lambda         CVADYSVLYNSASFSTFKCYGVSPTKLNDLCFTNVYADSFVIRGDEVRQIAPGQTGKIAD
MZ496613/C.37/Lambda         CVADYSVLYNSASFSTFKCYGVSPTKLNDLCFTNVYADSFVIRGDEVRQIAPGQTGKIAD
OU517038/C.37/Lambda         CVADYSVLYNSASFSTFKCYGVSPTKLNDLCFTNVYADSFVIRGDEVRQIAPGQTGKIAD
MZ953277/C.37/Lambda         CVADYSVLYNSASFSTFKCYGVSPTKLNDLCFTNVYADSFVIRGDEVRQIAPGQTGKIAD
MZ942542/C.37/Lambda         CVADYSVLYNSASFSTFKCYGVSPTKLNDLCFTNVYADSFVIRGDEVRQIAPGQTGKIAD
MZ857990/C.37/Lambda         CVADYSVLYNSASFSTFKCYGVSPTKLNDLCFTNVYADSFVIRGDEVRQIAPGQTGKIAD
```

Sequence	430 440 450 460 470 480
MN908947/Wuhan-Hu-1	YNYKLPDDFTGCVIAWNSNNLDSKVGGNYNYLYRLFRKSNLKPFERDISTEIYQAGSTPC
BS001137/B.1.1.7/Alpha	YNYKLPDDFTGCVIAWNSNNLDSKVGGNYNYLYRLFRKSNLKPFERDISTEIYQAGSTPC
BS001138/B.1.1.7/Alpha	YNYKLPDDFTGCVIAWNSNNLDSKVGGNYNYLYRLFRKSNLKPFERDISTEIYQAGSTPC
BS001139/B.1.1.7/Alpha	YNYKLPDDFTGCVIAWNSNNLDSKVGGNYNYLYRLFRKSNLKPFERDISTEIYQAGSTPC
BS001140/B.1.1.7/Alpha	YNYKLPDDFTGCVIAWNSNNLDSKVGGNYNYLYRLFRKSNLKPFERDISTEIYQAGSTPC
MW913362/B.1.1.7/Alpha	YNYKLPDDFTGCVIAWNSNNLDSKVGGNYNYLYRLFRKSNLKPFERDISTEIYQAGSTPC
MZ266365/B.1.1.7/Alpha	YNYKLPDDFTGCVIAWNSNNLDSKVGGNYNYLYRLFRKSNLKPFERDISTEIYQAGSTPC
MZ350105/B.1.1.7/Alpha	YNYKLPDDFTGCVIAWNSNNLDSKVGGNYNYLYRLFRKSNLKPFERDISTEIYQAGSTPC
MZ350109/B.1.1.7/Alpha	YNYKLPDDFTGCVIAWNSNNLDSKVGGNYNYLYRLFRKSNLKPFERDISTEIYQAGSTPC
MZ888516/B.1.1.7/Alpha	YNYKLPDDFTGCVIAWNSNNLDSKVGGNYNYLYRLFRKSNLKPFERDISTEIYQAGSTPC
OU562607/B.1.1.7/Alpha	YNYKLPDDFTGCVIAWNSNNLDSKVGGNYNYLYRLFRKSNLKPFERDISTEIYQAGSTPC
OU514166/B.1.1.7/Alpha	YNYKLPDDFTGCVIAWNSNNLDSKVGGNYNYLYRLFRKSNLKPFERDISTEIYQAGSTPC
OU538851/B.1.1.7/Alpha	YNYKLPDDFTGCVIAWNSNNLDSKVGGNYNYLYRLFRKSNLKPFERDISTEIYQAGSTPC
OU538849/B.1.1.7/Alpha	YNYKLPDDFTGCVIAWNSNNLDSKVGGNYNYLYRLFRKSNLKPFERDISTEIYQAGSTPC
MZ895877/B.1.1.7/Alpha	YNYKLPDDFTGCVIAWNSNNLDSKVGGNYNYLYRLFRKSNLKPFERDISTEIYQAGSTPC
OU514176/B.1.1.7/Alpha	YNYKLPDDFTGCVIAWNSNNLDSKVGGNYNYLYRLFRKSNLKPFERDISTEIYQAGSTPC
OU562608/B.1.1.7/Alpha	YNYKLPDDFTGCVIAWNSNNLDSKVGGNYNYLYRLFRKSNLKPFERDISTEIYQAGSTPC
MW981442/B.1.351/Beta	YNYKLPDDFTGCVIAWNSNNLDSKVGGNYNYLYRLFRKSNLKPFERDISTEIYQAGSTPC
MZ298839/B.1.351/Beta	YNYKLPDDFTGCVIAWNSNNLDSKVGGNYNYLYRLFRKSNLKPFERDISTEIYQAGSTPC
MZ913100/B.1.351/Beta	YNYKLPDDFTGCVIAWNSNNLDSKVGGNYNYLYRLFRKSNLKPFERDISTEIYQAGSTPC
MZ879365/B.1.351/Beta	YNYKLPDDFTGCVIAWNSNNLDSKVGGNYNYLYRLFRKSNLKPFERDISTEIYQAGSTPC
OU525106/B.1.351/Beta	YNYKLPDDFTGCVIAWNSNNLDSKVGGNYNYLYRLFRKSNLKPFERDISTEIYQAGSTPC
OU539352/B.1.351/Beta	YNYKLPDDFTGCVIAWNSNNLDSKVGGNYNYLYRLFRKSNLKPFERDISTEIYQAGSTPC
MZ202314/B.1.351/Beta	YNYKLPDDFTGCVIAWNSNNLDSKVGGNYNYLYRLFRKSNLKPFERDISTEIYQAGSTPC
MZ298840/B.1.351/Beta	YNYKLPDDFTGCVIAWNSNNLDSKVGGNYNYLYRLFRKSNLKPFERDISTEIYQAGSTPC
MZ376663/B.1.351/Beta	YNYKLPDDFTGCVIAWNSNNLDSKVGGNYNYLYRLFRKSNLKPFERDISTEIYQAGSTPC
LC643036/B.1.617.2/Delta	YNYKLPDDFTGCVIAWNSNNLDSKVGGNYNYRYRLFRKSNLKPFERDISTEIYQAGSKPC
LC643044/B.1.617.2/Delta	YNYKLPDDFTGCVIAWNSNNLDSKVGGNYNYRYRLFRKSNLKPFERDISTEIYQAGSKPC
MZ853946/B.1.617.2/Delta	YNYKLPDDFTGCVIAWNSNNLDSKVGGNYNYRYRLFRKSNLKPFERDISTEIYQAGSKPC
MZ854386/B.1.617.2/Delta	YNYKLPDDFTGCVIAWNSNNLDSKVVGNYNYRYRLFRKSNLKPFERDISTEIYQAGSKPC
MZ888532/B.1.617.2/Delta	YNYKLPDDFTGCVIAWNSNNLDSKVGGNYNYRYRLFRKSNLKPFERDISTEIYQAGSKPC
MZ888533/B.1.617.2/Delta	YNYKLPDDFTGCVIAWNSNNLDSKVGGNYNYRYRLFRKSNLKPFERDISTEIYQAGSKPC
MZ896136/B.1.617.2/Delta	YNYKLPDDFTGCVIAWNSNNLDSKVGGNYNYRYRLFRKSNLKPFERDISTEIYQAGSKPC
OU539261/B.1.617.2/Delta	YNYKLPDDFTGCVIAWNSNNLDSKVGGNYNYRYRLFRKSNLKPFERDISTEIYQAGSKPC
MZ854389/B.1.617.2/Delta	YNYKLPDDFTGCVIAWNSNNLDSKVGGNYNYRYRLFRKSNLKPFERDISTEIYQAGSKPC
OU562629/B.1.617.2/Delta	YNYKLPDDFTGCVIAWNSNNLDSKVGGNYNYRYRLFRKSNLKPFERDISTEIYQAGSKPC
OU539690/B.1.617.2/Delta	YNYKLPDDFTGCVIAWNSNNLDSKVGGNYNYRYRLFRKSNLKPFERDISTEIYQAGSKPC
MZ854387/B.1.617.2/Delta	YNYKLPDDFTGCVIAWNSNNLDSKVGGNYNYRYRLFRKSNLKPFERDISTEIYQAGSKPC
OU539784/P.1/Gamma	YNYKLPDDFTGCVIAWNSNNLDSKVGGNYNYLYRLFRKSNLKPFERDISTEIYQAGSTPC
OU539830/P.1/Gamma	YNYKLPDDFTGCVIAWNSNNLDSKVGGNYNYLYRLFRKSNLKPFERDISTEIYQAGSTPC
OU539894/P.1/Gamma	YNYKLPDDFTGCVIAWNSNNLDSKVGGNYNYLYRLFRKSNLKPFERDISTEIYQAGSTPC
MZ477748/P.1/Gamma	YNYKLPDDFTGCVIAWNSNNLDSKVGGNYNYLYRLFRKSNLKPFERDISTEIYQAGSTPC
MZ477746/P.1/Gamma	YNYKLPDDFTGCVIAWNSNNLDSKVGGNYNYLYRLFRKSNLKPFERDISTEIYQAGSTPC
MW938104/P.1/Gamma	YNYKLPDDFTGCVIAWNSNNLDSKVGGNYNYLYRLFRKSNLKPFERDISTEIYQAGSTPC
MZ896179/P.1/Gamma	YNYKLPNDFTGCVIAWNSNNLDSKVGGNYNYLYRLFRKSNLKPFERDISTEIYQAGSTPC
MZ896359/P.1/Gamma	YNYKLPDDFTGCVIAWNSNNLDSKVGGNYNYLYRLFRKSNLKPFERDISTEIYQAGSTPC
MZ896669/P.1/Gamma	YNYKLPDDFTGCVIAWNSNNLDSKVGGNYNYLYRLFRKSNLKPFERDISTEIYQAGSTPC
OU565182/P.1/Gamma	YNYKLPDDFTGCVIAWNSNNLDSKVGGNYNYLYRLFRKSNLKPFERDISTEIYQAGSTPC
OU492255/P.1/Gamma	YNYKLPDDFTGCVIAWNSNNLDSKVGGNYNYLYRLFRKSNLKPFERDISTEIYQAGSTPC
MZ911221/B.1.427/Epsilon	YNYKLPDDFTGCVIAWNSNNLDSKVGGNYNYRYRLFRKSNLKPFERDISTEIYQAGSTPC
MZ375854/B.1.427/Epsilon	YNYKLPDDFTGCVIAWNSNNLDSKVGGNYNYRYRLFRKSNLKPFERDISTEIYQAGSTPC
MZ375858/B.1.427/Epsilon	YNYKLPDDFTGCVIAWNSNNLDSKVGGNYNYRYRLFRKSNLKPFERDISTEIYQAGSTPC
MZ943834/B.1.429/Epsilon	YNYKLPDDFTGCVIAWNSNNLDSKVGGNYNYRYRLFRKSNLKPFERDISTEIYQAGSTPC
MZ277390/B.1.429/Epsilon	YNYKLPDDFTGCVIAWNSNNLDSKVGGNYNYRYRLFRKSNLKPFERDISTEIYQAGSTPC
OU282717/B.1.429/Epsilon	YNYKLPDDFTGCVIAWNSNNLDSKVGGNYNYRYRLFRKSNLKPFERDISTEIYQAGSTPC
OU235436/B.1.429/Epsilon	YNYKLPDDFTGCVIAWNSNNLDSKVGGNYNYRYRLFRKSNLKPFERDISTEIYQAGSTPC
MZ687447/B.1.429/Epsilon	YNYKLPDDFTGCVIAWNSNNLDSKVGGNYNYRYRLFRKSNLKPFERDISTEIYQAGSTPC
MZ538882/B.1.617.1/Kappa	YNYKLPDDFTGCVIAWNSNNLDSKVGGNYNYRYRLFRKSNLKPFERDISTEIYQAGSTPC
MZ724425/B.1.617.1/Kappa	YNYKLPDDFTGCVIAWNSNNLDSKVGGNYNYRYRLFRKSNLKPFERDISTEIYQAGSTPC
MZ363839/B.1.617.1/Kappa	YNYKLPDDFTGCVIAWNSNNLDSKVGGNYNYRYRLFRKSNLKPFERDISTEIYQAGSTPC
MZ332524/B.1.617.1/Kappa	YNYKLPDDFTGCVIAWNSNNLDSKVGGNYNYRYRLFRKSNLKPFERDISTEIYQAGSTPC
MZ332527/B.1.617.1/Kappa	YNYKLPDDFTGCVIAWNSNNLDSKVGGNYNYRYRLFRKSNLKPFERDISTEIYQAGSTPC
LC633761/B.1.617.1/Kappa	YNYKLPDDFTGCVIAWNSNNLDSKVGGNYNYRYRLFRKSNLKPFERDISTEIYQAGSTPC
MZ562746/B.1.617.1/Kappa	YNYKLPDDFTGCVIAWNSNNLDSKVGGNYNYRYRLFRKSNLKPFERDISTEIYQAGSTPC
MZ571142/B.1.617.1/Kappa	YNYKLPDDFTGCVIAWNSNNLDSKVGGNYNYRYRLFRKSNLKPFERDISTEIYQAGSTPC
OU168413/B.1.617.1/Kappa	YNYKLPDDFTGCVIAWNSNNLDSKVGGNYNYRYRLFRKSNLKPFERDISTEIYQAGSTPC
MZ026886/B.1.617.1/Kappa	YNYKLPDDFTGCVIAWNSNNLDSKVGGNYNYRYRLFRKSNLKPFERDISTEIYQAGSTPC
OU172184/B.1.617.1/Kappa	YNYKLPDDFTGCVIAWNSNNLDSKVGGNYNYRYRLFRKSNLKPFERDISTEIYQAGSTPC
MZ397173/B.1.617.1/Kappa	YNYKLPDDFTGCVIAWNSNNLDSKVGGNYNYRYRLFRKSNLKPFERDISTEIYQAGSTPC
MZ397176/B.1.617.1/Kappa	YNYKLPDDFTGCVIAWNSNNLDSKVGGNYNYRYRLFRKSNLKPFERDISTEIYQAGSTPC
MZ397175/B.1.617.1/Kappa	YNYKLPDDFTGCVIAWNSNNLDSKVGGNYNYRYRLFRKSNLKPFERDISTEIYQAGSTPC
MZ397177/B.1.617.1/Kappa	YNYKLPDDFTGCVIAWNSNNLDSKVGGNYNYRYRLFRKSNLKPFERDISTEIYQAGSTPC
OU290035/B.1.617.1/Kappa	YNYKLPDDFTGCVIAWNSNNLDSKVGGNYNYRYRLFRKSNLKPFERDISTEIYQAGSTPC
OU265178/B.1.617.1/Kappa	YNYKLPDDFTGCVIAWNSNNLDSKVGGNYNYRYRLFRKSNLKPFERDISTEIYQAGSTPC
OU254949/B.1.617.1/Kappa	YNYKLPDDFTGCVIAWNSNNLDSKVGGNYNYRYRLFRKSNLKPFERDISTEIYQAGSTPC
OU247018/B.1.617.1/Kappa	YNYKLPDDFTGCVIAWNSNNLDSKVGGNYNYRYRLFRKSNLKPFERDISTEIYQAGSTPC
OU061269/B.1.617.1/Kappa	YNYKLPDDFTGCVIAWNSNNLDSKVGGNYNYRYRLFRKSNLKPFERDISTEIYQAGSTPC
OU182966/B.1.617.1/Kappa	YNYKLPDDFTGCVIAWNSNNLDSKVGGNYNYRYRLFRKSNLKPFERDISTEIYQAGSTPC
MZ746235/B.1.617.1/Kappa	YNYKLPDDFTGCVIAWNSNNLDSKVGGNYNYRYRLFRKSNLKPFERDISTEIYQAGSTPC
MZ487800/B.1.617.1/Kappa	YNYKLPDDFTGCVIAWNSNNLDSKVGGNYNYRYRLFRKSNLKPFERDISTEIYQAGSTPC
OU322235/B.1.617.1/Kappa	YNYKLPDDFTGCVIAWNSNNLDSKVGGNYNYRYRLFRKSNLKPFERDISTEIYQAGSTPC
MZ953185/C.37/Lambda	YNYKLPDDFTGCVIAWNSNNLDSKVGGNYNYQYRLFRKSNLKPFERDISTEIYQAGSTPC
MZ937666/C.37/Lambda	YNYKLPDDFTGCVIAWNSNNLDSKVGGNYNYQYRLFRKSNLKPFERDISTEIYQAGSTPC
OU356036/C.37/Lambda	YNYKLPDDFTGCVIAWNSNNLDSKVGGNYNYQYRLFRKSNLKPFERDISTEIYQAGSTPC
OU351967/C.37/Lambda	YNYKLPDDFTGCVIAWNSNNLDSKVGGNYNYQYRLFRKSNLKPFERDISTEIYQAGSTPC
OU053018/C.37/Lambda	YNYKLPDDFTGCVIAWNSNNLDSKVGGNYNYQYRLFRKSNLKPFERDISTEIYQAGSTPC
OU268528/C.37/Lambda	YNYKLPDDFTGCVIAWNSNNLDSKVGGNYNYQYRLFRKSNLKPFERDISTEIYQAGSTPC
OU100999/C.37/Lambda	YNYKLPDDFTGCVIAWNSNNLDSKVGGNYNYQYRLFRKSNLKPFERDISTEIYQAGSTPC
MZ275295/C.37/Lambda	YNYKLPDDFTGCVIAWNSNNLDSKVGGNYNYQYRLFRKSNLKPFERDISTEIYQAGSTPC
MZ275301/C.37/Lambda	YNYKLPDDFTGCVIAWNSNNLDSKVGGNYNYQYRLFRKSNLKPFERDISTEIYQAGSTPC
MZ908711/C.37/Lambda	YNYKLPDDFTGCVIAWNSNNLDSKVGGNYNYQYRLFRKSNLKPFERDISTEIYQAGSTPC
MZ496613/C.37/Lambda	YNYKLPDDFTGCVIAWNSNNLDSKVGGNYNYQYRLFRKSNLKPFERDISTEIYQAGSTPC
OU517038/C.37/Lambda	YNYKLPDDFTGCVIAWNSNNLDSKVGGNYNYQYRLFRKSNLKPFERDISTEIYQAGSTPC
MZ953277/C.37/Lambda	YNYKLPDDFTGCVIAWNSNNLDSKVGGNYNYQYRLFRKSNLKPFERDISTEIYQAGSTPC
MZ942542/C.37/Lambda	YNYKLPDDFTGCVIAWNSNNLDSKVGGNYNYQYRLFRKSNLKPFERDISTEIYQAGSTPC
MZ857990/C.37/Lambda	YNYKLPDDFTGCVIAWNSNNLDSKVGGNYNYQYRLFRKSNLKPFERDISTEIYQAGSTPC

	490 500 510 520 530 540
MN908947/Wuhan-Hu-1	NGVEGFNCYFPLQSYGFQPTNGVGYQPYRVVVLSFELLHAPATVCGPKKSTNLVKNKCVN
BS001137/B.1.1.7/Alpha	NGVEGFNCYFPLQSYGFQPTYGVGYQPYRVVVLSFELLHAPATVCGPKKSTNLVKNKCVN
BS001138/B.1.1.7/Alpha	NGVEGFNCYFPLQSYGFQPTYGVGYQPYRVVVLSFELLHAPATVCGPKKSTNLVKNKCVN
BS001139/B.1.1.7/Alpha	NGVEGFNCYFPLQSYGFQPTYGVGYQPYRVVVLSFELLHAPATVCGPKKSTNLVKNKCVN
BS001140/B.1.1.7/Alpha	NGVEGFNCYFPLQSYGFQPTYGVGYQPYRVVVLSFELLHAPATVCGPKKSTNLVKNKCVN
MW913362/B.1.1.7/Alpha	NGVEGFNCYFPLQSYGFQPTYGVGYQPYRVVVLSFELLHAPATVCGPKKSTNLVKNKCVN
MZ266365/B.1.1.7/Alpha	NGVEGFNCYFPLQSYGFQPTYGVGYQPYRVVVLSFELLHAPATVCGPKKSTNLVKNKCVN
MZ350105/B.1.1.7/Alpha	NGVEGFNCYFPLQSYGFQPTYGVGYQPYRVVVLSFELLHAPATVCGPKKSTNLVKNKCVN
MZ350109/B.1.1.7/Alpha	NGVEGFNCYFPLQSYGFQPTYGVGYQPYRVVVLSFELLHAPATVCGPKKSTNLVKNKCVN
MZ888516/B.1.1.7/Alpha	NGVEGFNCYFPLQSYGFQPTYGVGYQPYRVVVLSFELLHAPATVCGPKKSTNLVKNKCVN
OU562607/B.1.1.7/Alpha	NGVEGFNCYFPLQSYGFQPTYGVGYQPYRVVVLSFELLHAPATVCGPKKSTNLVKNKCVN
OU514166/B.1.1.7/Alpha	NGVEGFNCYFPLQSYGFQPTYGVGYQPYRVVVLSFELLHAPATVCGPKKSTNLVKNKCVN
OU538851/B.1.1.7/Alpha	NGVEGFNCYFPLQSYGFQPTYGVGYQPYRVVVLSFELLHAPATVCGPKKSTNLVKNKCVN
OU538849/B.1.1.7/Alpha	NGVEGFNCYFPLQSYGFQPTYGVGYQPYRVVVLSFELLHAPATVCGPKKSTNLVKNKCVN
MZ895877/B.1.1.7/Alpha	NGVEGFNCYFPLQSYGFQPTYGVGYQPYRVVVLSFELLHAPATVCGPKKSTNLVKNKCVN
OU514176/B.1.1.7/Alpha	NGVEGFNCYFPLQSYGFQPTYGVGYQPYRVVVLSFELLHAPATVCGPKKSTNLVKNKCVN
OU562608/B.1.1.7/Alpha	NGVEGFNCYFPLQSYGFQPTYGVGYQPYRVVVLSFELLHAPATVCGPKKSTNLVKNKCVN
MW981442/B.1.351/Beta	NGVKGFNCYFPLQSYGFQPTYGVGYQPYRVVVLSFELLHAPATVCGPKKSTNLVKNKCVN
MZ298839/B.1.351/Beta	NGVKGFNCYFPLQSYGFQPTYGVGYQPYRVVVLSFELLHAPATVCGPKKSTNLVKNKCVN
MZ913100/B.1.351/Beta	NGVKGFNCYFPLQSYGFQPTYGVGYQPYRVVVLSFELLHAPATVCGPKKSTNLVKNKCVN
MZ879365/B.1.351/Beta	NGVKGFNCYFPLQSYGFQPTYGVGYQPYRVVVLSFELLHAPATVCGPKKSTNLVKNKCVN
OU525106/B.1.351/Beta	NGVKGFNCYFPLQSYGFQPTYGVGYQPYRVVVLSFELLHAPATVCGPKKSTNLVKNKCVN
OU539352/B.1.351/Beta	NGVKGFNCYFPLQSYGFQPTYGVGYQPYRVVVLSFELLHAPATVCGPKKSTNLVKNKCVN
MZ202314/B.1.351/Beta	NGVKGFNCYFPLQSYGFQPTYGVGYQPYRVVVLSFELLHAPATVCGPKKSTNLVKNKCVN
MZ298840/B.1.351/Beta	NGVKGFNCYFPLQSYGFQPTYGVGYQPYRVVVLSFELLHAPATVCGPKKSTNLVKNKCVN
MZ376663/B.1.351/Beta	NGVKGFNCYFPLQSYGFQPTYGVGYQPYRVVVLSFELLHAPATVCGPKKSTNLVKNKCVN
LC643036/B.1.617.2/Delta	NGVEGFNCYFPLQSYGFQPTNGVGYQPYRVVVLSFELLHAPATVCGPKKSTNLVKNKCVN
LC643044/B.1.617.2/Delta	NGVEGFNCYFPLQSYGFQPTNGVGYQPYRVVVLSFELLHAPATVCGPKKSTNLVKNKCVN
MZ853946/B.1.617.2/Delta	NGVEGFNCYFPLQSYGFQPTNGVGYQPYRVVVLSFELLHAPATVCGPKKSTNLVKNKCVN
MZ854386/B.1.617.2/Delta	NGVEGFNCYFPLQSYGFQPTNGVGYQPYRVVVLSFELLHAPATVCGPKKSTNLVKNKCVN
MZ888532/B.1.617.2/Delta	NGVEGFNCYFPLQSYGFQPTNGVGYQPYRVVVLSFELLHAPATVCGPKKSTNLVKNKCVN
MZ888533/B.1.617.2/Delta	NGVEGFNCYFPLQSYGFQPTNGVGYQPYRVVVLSFELLHAPATVCGPKKSTNLVKNKCVN
MZ896136/B.1.617.2/Delta	NGVEGFNCYFPLQSYGFQPTNGVGYQPYRVVVLSFELLHAPATVCGPKKSTNLVKNKCVN
OU539261/B.1.617.2/Delta	NGVEGFNCYFPLQSYGFQPTNGVGYQPYRVVVLSFELLHAPATVCGPKKSTNLVKNKCVN
MZ854389/B.1.617.2/Delta	NGVEGFNCYFPLQSYGFQPTNGVGYQPYRVVVLSFELLHAPATVCGPKKSTNLVKNKCVN
OU562629/B.1.617.2/Delta	NGVEGFNCYFPLQSYGFQPTNGVGYQPYRVVVLSFELLHAPATVCGPKKSTNLVKNKCVN
OU539690/B.1.617.2/Delta	NGVEGFNCYFPLQSYGFQPTNGVGYQPYRVVVLSFELLHAPATVCGPKKSTNLVKNKCVN
MZ854387/B.1.617.2/Delta	NGVEGFNCYFPLQSYGFQPTNGVGYQPYRVVVLSFELLHAPATVCGPKKSTNLVKNKCVN
OU539784/P.1/Gamma	NGVKGFNCYFPLQSYGFQPTYGVGYQPYRVVVLSFELLHAPATVCGPKKSTNLVKNKCVN
OU539830/P.1/Gamma	NGVKGFNCYFPLQSYGFQPTYGVGYQPYRVVVLSFELLHAPATVCGPKKSTNLVKNKCVN
OU539894/P.1/Gamma	NGVKGFNCYFPLQSYGFQPTYGVGYQPYRVVVLSFELLHAPATVCGPKKSTNLVKNKCVN
MZ477748/P.1/Gamma	NGVKGFNCYFPLQSYGFQPTYGVGYQPYRVVVLSFELLHAPATVCGPKKSTNLVKNKCVN
MZ477746/P.1/Gamma	NGVKGFNCYFPLQSYGFQPTYGVGYQPYRVVVLSFELLHAPATVCGPKKSTNLVKNKCVN
MW938104/P.1/Gamma	NGVKGFNCYFPLQSYGFQPTYGVGYQPYRVVVLSFELLHAPATVCGPKKSTNLVKNKCVN
MZ896179/P.1/Gamma	NGVKGFNCYFPLQSYGFQPTYGVGYQPYRVVVLSFELLHAPATVCGPKKSTNLVKNKCVN
MZ896359/P.1/Gamma	NGVKGFNCYFPLQSYGFQPTYGVGYQPYRVVVLSFELLHAPATVCGPKKSTNLVKNKCVN
MZ896669/P.1/Gamma	NGVKGFNCYFPLQSYGFQPTYGVGYQPYRVVVLSFELLHAPATVCGPKKSTNLVKNKCVN
OU565182/P.1/Gamma	NGVKGFNCYFPLQSYGFQPTYGVGYQPYRVVVLSFELLHAPATVCGPKKSTNLVKNKCVN
OU492255/P.1/Gamma	NGVKGFNCYFPLQSYGFQPTYGVGYQPYRVVVLSFELLHAPATVCGPKKSTNLVKNKCVN
MZ911221/B.1.427/Epsilon	NGVEGFNCYFPLQSYGFQPTNGVGYQPYRVVVLSFELLHAPATVCGPKKSTNLVKNKCVN
MZ375854/B.1.427/Epsilon	NGVEGFNCYFPLQSYGFQPTNGVGYQPYRVVVLSFELLHAPATVCGPKKSTNLVKNKCVN
MZ375858/B.1.427/Epsilon	NGVEGFNCYFPLQSYGFQPTNGVGYQPYRVVVLSFELLHAPATVCGPKKSTNLVKNKCVN
MZ943834/B.1.429/Epsilon	NGVEGFNCYFPLQSYGFQPTNGVGYQPYRVVVLSFELLHAPATVCGPKKSTNLVKNKCVN
MZ277390/B.1.429/Epsilon	NGVEGFNCYFPLQSYGFQPTNGVGYQPYRVVVLSFELLHAPATVCGPKKSTNLVKNKCVN
OU282717/B.1.429/Epsilon	NGVEGFNCYFPLQSYGFQPTNGVGYQPYRVVVLSFELLHAPATVCGPKKSTNLVKNKCVN
OU235436/B.1.429/Epsilon	NGVEGFNCYFPLQSYGFQPTNGVGYQPYRVVVLSFELLHAPATVCGPKKSTNLVKNKCVN
MZ687447/B.1.429/Epsilon	NGVEGFNCYFPLQSYGFQPTNGVGYQPYRVVVLSFELLHAPATVCGPKKSTNLVKNKCVN
MZ538882/B.1.617.1/Kappa	NGVQGFNCYFPLQSYGFQPTNGVGYQPYRVVVLSFELLHAPATVCGPKKSTNLVKNKCVN
MZ724425/B.1.617.1/Kappa	NGVQGFNCYFPLQSYGFQPTNGVGYQPYRVVVLSFELLHAPATVCGPKKSTNLVKNKCVN
MZ363839/B.1.617.1/Kappa	NGVQGFNCYFPLQSYGFQPTNGVGYQPYRVVVLSFELLHAPATVCGPKKSTNLVKNKCVN
MZ332524/B.1.617.1/Kappa	NGVQGFNCYFPLQSYGFQPTNGVGYQPYRVVVLSFELLHAPATVCGPKKSTNLVKNKCVN
MZ332527/B.1.617.1/Kappa	NGVQGFNCYFPLQSYGFQPTNGVGYQPYRVVVLSFELLHAPATVCGPKKSTNLVKNKCVN
LC633761/B.1.617.1/Kappa	NGVQGFNCYFPLQSYGFQPTNGVGYQPYRVVVLSFELLHAPATVCGPKKSTNLVKNKCVN
MZ562746/B.1.617.1/Kappa	NGVQGFNCYFPLQSYGFQPTNGVGYQPYRVVVLSFELLHAPVTVCGPKKSTNLVKNKCVN
MZ571142/B.1.617.1/Kappa	NGVQGFNCYFPLQSYGFQPTNGVGYQPYRVVVLSFELLHAPATVCGPKKSTNLVKNKCVN
OU168413/B.1.617.1/Kappa	NGVQGFNCYFPLQSYGFQPTNGVGYQPYRVVVLSFELLHAPATVCGPKKSTNLVKNKCVN
MZ026886/B.1.617.1/Kappa	NGVQGFNCYFPLQSYGFQPTNGVGYQPYRVVVLSFELLHAPATVCGPKKSTNLVKNKCVN
OU172184/B.1.617.1/Kappa	NGVQGFNCYFPLQSYGFQPTNGVGYQPYRVVVLSFELLHAPATVCGPKKSTNLVKNKCVN
MZ397173/B.1.617.1/Kappa	NGVQGFNCYFPLQSYGFQPTNGVGYQPYRVVVLSFELLHAPATVCGPKKSTNLVKNKCVN
MZ397176/B.1.617.1/Kappa	NGVQGFNCYFPLQSYGFQPTNGVGYQPYRVVVLSFELLHAPATVCGPKKSTNLVKNKCVN
MZ397175/B.1.617.1/Kappa	NGVQGFNCYFPLQSYGFQPTNGVGYQPYRVVVLSFELLHAPATVCGPKKSTNLVKNKCVN
MZ397177/B.1.617.1/Kappa	NGVQGFNCYFPLQSYGFQPTNGVGYQPYRVVVLSFELLHAPATVCGPKKSTNLVKNKCVN
OU290035/B.1.617.1/Kappa	NGVQGFNCYFPLQSYGFQPTNGVGYQPYRVVVLSFELLHAPATVCGPKKSTNLVKNKCVN
OU265178/B.1.617.1/Kappa	NGVQGFNCYFPLQSYGFQPTNGVGYQPYRVVVLSFELLHAPATVCGPKKSTNLVKNKCVN
OU254949/B.1.617.1/Kappa	NGVQGFNCYFPLQSYGFQPTNGVGYQPYRVVVLSFELLHAPATVCGPKKSTNLVKNKCVN
OU247018/B.1.617.1/Kappa	NGVQGFNCYFPLQSYGFQPTNGVGYQPYRVVVLSFELLHAPATVCGPKKSTNLVKNKCVN
OU061269/B.1.617.1/Kappa	NGVQGFNCYFPLQSYGFQPTNGVGYQPYRVVVLSFELLHAPATVCGPKKSTNLVKNKCVN
OU182966/B.1.617.1/Kappa	NGVQGFNCYFPLQSYGFQPTNGVGYQPYRVVVLSFELLHAPATVCGPKKSTNLVKNKCVN
MZ746235/B.1.617.1/Kappa	NGVQGFNCYFPLQSYGFQPTNGVGYQPYRVVVLSFELLHAPATVCGPKKSTNLVKNKCVN
MZ487800/B.1.617.1/Kappa	NGVQGFNCYFPLQSYGFQPTNGVGYQPYRVVVLSFELLHAPATVCGPKKSTNLVKNKCVN
OU322235/B.1.617.1/Kappa	NGVQGFNCYFPLQSYGFQPTNGVGYQPYRVVVLSFELLHAPATVCGPKKSTNLVKNKCVN
MZ953185/C.37/Lambda	NGVEGFNCYSPLQSYGFQPTNGVGYQPYRVVVLSFELLHAPATVCGPKKSTNLVKNKCVN
MZ937666/C.37/Lambda	NGVEGFNCYSPLQSYGFQPTNGVGYQPYRVVVLSFELLHAPATVCGPKKSTNLVKNKCVN
OU356036/C.37/Lambda	NGVEGFNCYSPLQSYGFQPTNGVGYQPYRVVVLSFELLHAPATVCGPKKSTNLVKNKCVN
OU351967/C.37/Lambda	NGVEGFNCYSPLQSYGFQPTNGVGYQPYRVVVLSFELLHAPATVCGPKKSTNLVKNKCVN
OU053018/C.37/Lambda	NGVEGFNCYSPLQSYGFQPTNGVGYQPYRVVVLSFELLHAPATVCGPKKSTNLVKNKCVN
OU268528/C.37/Lambda	NGVEGFNCYSPLQSYGFQPTNGVGYQPYRVVVLSFELLHAPATVCGPKKSTNLVKNKCVN
OU100999/C.37/Lambda	NGVEGFNCYSPLQSYGFQPTNGVGYQPYRVVVLSFELLHAPATVCGPKKSTNLVKNKCVN
MZ275295/C.37/Lambda	NGVEGFNCYSPLQSYGFQPTNGVGYQPYRVVVLSFELLHAPATVCGPKKSTNLVKNKCVN
MZ275301/C.37/Lambda	NGVEGFNCYSPLQSYGFQPTNGVGYQPYRVVVLSFELLHAPATVCGPKKSTNLVKNKCVN
MZ908711/C.37/Lambda	NGVEGFNCYSPLQSYGFQPTNGVGYQPYRVVVLSFELLHAPATVCGPKKSTNLVKNKCVN
MZ496613/C.37/Lambda	NGVEGFNCYSPLQSYGFQPTNGVGYQPYRVVVLSFELLHAPATVCGPKKSTNLVKNKCVN
OU517038/C.37/Lambda	NGVEGFNCYSPLQSYGFQPTNGVGYQPYRVVVLSFELLHAPATVCGPKKSTNLVKNKCVN
MZ953277/C.37/Lambda	NGVEGFNCYSPLQSYGFQPTNGVGYQPYRVVVLSFELLHAPATVCGPKKSTNLVKNKCVN
MZ942542/C.37/Lambda	NGVEGFNCYSPLQSYGFQPTNGVGYQPYRVVVLSFELLHAPATVCGPKKSTNLVKNKCVN
MZ857990/C.37/Lambda	NGVEGFNCYSPLQSYGFQPTNGVGYQPYRVVVLSFELLHAPATVCGPKKSTNLVKNKCVN

	550 560 570 580 590 600
MN908947/Wuhan-Hu-1	FNFNGLTGTGVLTESNKKFLPFQQFGRDIADTTDAVRDPQTLEILDITPCSFGGVSVITP
BS001137/B.1.1.7/Alpha	FNFNGLTGTGVLTESNKKFLPFQQFGRDIDDTTDAVRDPQTLEILDITPCSFGGVSVITP
BS001138/B.1.1.7/Alpha	FNFNGLTGTGVLTESNKKFLPFQQFGRDIDDTTDAVRDPQTLEILDITPCSFGGVSVITP
BS001139/B.1.1.7/Alpha	FNFNGLTGTGVLTESNKKFLPFQQFGRDIDDTTDAVRDPQTLEILDITPCSFGGVSVITP
BS001140/B.1.1.7/Alpha	FNFNGLTGTGVLTESNKKFLPFQQFGRDIDDTTDAVRDPQTLEILDITPCSFGGVSVITP
MW913362/B.1.1.7/Alpha	FNFNGLTGTGVLTESNKKFLPFQQFGRDIDDTTDAVRDPQTLEILDITPCSFGGVSVITP
MZ266365/B.1.1.7/Alpha	FNFNGLTGTGVLTESNKKFLPFQQFGRDIDDTTDAVRDPQTLEILDITPCSFGGVSVITP
MZ350105/B.1.1.7/Alpha	FNFNGLTGTGVLTESNKKFLPFQQFGRDIDDTTDAVRDPQTLEILDITPCSFGGVSVITP
MZ350109/B.1.1.7/Alpha	FNFNGLTGTGVLTESNKKFLPFQQFGRDIDDTTDAVRDPQTLEILDITPCSFGGVSVITP
MZ888516/B.1.1.7/Alpha	FNFNGLTGTGVLTESNKKFLPFQQFGRDIDDTTDAVRDPQTLEILDITPCSFGGVSVITP
OU562607/B.1.1.7/Alpha	FNFNGLTGTGVLTESNKKFLPFQQFGRDIDDTTDAVRDPQTLEILDITPCSFGGVSVITP
OU514166/B.1.1.7/Alpha	FNFNGLTGTGVLTESNKKFLPFQQFGRDIDDTTDAVRDPQTLEILDITPCSFGGVSVITP
OU538851/B.1.1.7/Alpha	FNFNGLTGTGVLTESNKKFLPFQQFGRDIDDTTDAVRDPQTLEILDITPCSFGGVSVITP
OU538849/B.1.1.7/Alpha	FNFNGLTGTGVLTESNKKFLPFQQFGRDIDDTTDAVRDPQTLEILDITPCSFGGVSVITP
MZ895877/B.1.1.7/Alpha	FNFNGLTGTGVLTESNKKFLPFQQFGRDIDDTTDAVRDPQTLEILDITPCSFGGVSVITP
OU514176/B.1.1.7/Alpha	FNFNGLTGTGVLTESNKKFLPFQQFGRDIDDTTDAVRDPQTLEILDITPCSFGGVSVITP
OU562608/B.1.1.7/Alpha	FNFNGLTGTGVLTESNKKFLPFQQFGRDIDDTTDAVRDPQTLEILDITPCSFGGVSVITP
MW981442/B.1.351/Beta	FNFNGLTGTGVLTESNKKFLPFQQFGRDIADTTDAVRDPQTLEILDITPCSFGGVSVITP
MZ298839/B.1.351/Beta	FNFNGLTGTGVLTESNKKFLPFQQFGRDIADTTDAVRDPQTLEILDITPCSFGGVSVITP
MZ913100/B.1.351/Beta	FNFNGLTGTGVLTESNKKFLPFQQFGRDIADTTDAVRDPQTLEILDITPCSFGGVSVITP
MZ879365/B.1.351/Beta	FNFNGLTGTGVLTESNKKFLPFQQFGRDIADTTDAVRDPQTLEILDITPCSFGGVSVITP
OU525106/B.1.351/Beta	FNFNGLTGTGVLTESNKKFLPFQQFGRDIADTTDAVRDPQTLEILDITPCSFGGVSVITP
OU539352/B.1.351/Beta	FNFNGLTGTGVLTESNKKFLPFQQFGRDIADTTDAVRDPQTLEILDITPCSFGGVSVITP
MZ202314/B.1.351/Beta	FNFNGLTGTGVLTESNKKFLPFQQFGRDIADTTDAVRDPQTLEILDITPCSFGGVSVITP
MZ298840/B.1.351/Beta	FNFNGLTGTGVLTESNKKFLPFQQFGRDIADTTDAVRDPQTLEILDITPCSFGGVSVITP
MZ376663/B.1.351/Beta	FNFNGLTGTGVLTESNKKFLPFQQFGRDIADTTDAVRDPQTLEILDITPCSFGGVSVITP
LC643036/B.1.617.2/Delta	FNFNGLTGTGVLTESNKKFLPFQQFGRDIADTTDAVRDPQTLEILDITPCSFGGVSVITP
LC643044/B.1.617.2/Delta	FNFNGLTGTGVLTESNKKFLPFQQFGRDIADTTDAVRDPQTLEILDITPCSFGGVSVITP
MZ853946/B.1.617.2/Delta	FNFNGLTGTGVLTESNKKFLPFQQFGRDIADTTDAVRDPQTLEILDITPCSFGGVSVITP
MZ854386/B.1.617.2/Delta	FNFNGLTGTGVLTESNKKFLPFQQFGRDIADTTDAVRDPQTLEILDITPCSFGGVSVITP
MZ888532/B.1.617.2/Delta	FNFNGLTGTGVLTESNKKFLPFQQFGRDIADTTDAVRDPQTLEILDITPCSFGGVSVITP
MZ888533/B.1.617.2/Delta	FNFNGLTGTGVLTESNKKFLPFQQFGRDIADTTDAVRDPQTLEILDITPCSFGGVSVITP
MZ896136/B.1.617.2/Delta	FNFNGLTGTGVLTESNKKFLPFQQFGRDIADTTDAVRDPQTLEILDITPCSFGGVSVITP
OU539261/B.1.617.2/Delta	FNFNGLTGTGVLTESNKKFLPFQQFGRDIADTTDAVRDPQTLEILDITPCSFGGVSVITP
MZ854389/B.1.617.2/Delta	FNFNGLTGTGVLTESNKKFLPFQQFGRDIADTTDAVRDPQTLEILDITPCSFGGVSVITP
OU562629/B.1.617.2/Delta	FNFNGLTGTGVLTESNKKFLPFQQFGRDIADTTDAVRDPQTLEILDITPCSFGGVSVITP
OU539690/B.1.617.2/Delta	FNFNGLTGTGVLTESNKKFLPFQQFGRDIADTTDAVRDPQTLEILDITPCSFGGVSVITP
MZ854387/B.1.617.2/Delta	FNFNGLTGTGVLTESNKKFLPFQQFGRDIADTTDAVRDPQTLEILDITPCSFGGVSVITP
OU539784/P.1/Gamma	FNFNGLTGTGVLTESNKKFLPFQQFGRDIADTTDAVRDPQTLEILDITPCSFGGVSVITP
OU539830/P.1/Gamma	FNFNGLTGTGVLTESNKKFLPFQQFGRDIADTTDAVRDPQTLEILDITPCSFGGVSVITP
OU539894/P.1/Gamma	FNFNGLTGTGVLTESNKKFLPFQQFGRDIADTTDAVRDPQTLEILDITPCSFGGVSVITP
MZ477748/P.1/Gamma	FNFNGLTGTGVLTESNKKFLPFQQFGRDIADTTDAVRDPQTLEILDITPCSFGGVSVITP
MZ477746/P.1/Gamma	FNFNGLTGTGVLTESNKKFLPFQQFGRDIADTTDAVRDPQTLEILDITPCSFGGVSVITP
MW938104/P.1/Gamma	FNFNGLTGTGVLTESNKKFLPFQQFGRDIADTTDAVRDPQTLEILDITPCSFGGVSVITP
MZ896179/P.1/Gamma	FNFNGLTGTGVLTESNKKFLPFQQFGRDIADTTDAVRDPQTLEILDITPCSFGGVSVITP
MZ896359/P.1/Gamma	FNFNGLTGTGVLTESNKKFLPFQQFGRDIADTTDAVRDPQTLEILDITPCSFGGVSVITP
MZ896669/P.1/Gamma	FNFNGLTGTGVLTESNKKFLPFQQFGRDIADTTDAVRDPQTLEILDITPCSFGGVSVITP
OU565182/P.1/Gamma	FNFNGLTGTGVLTESNKKFLPFQQFGRDIADTTDAVRDPQTLEILDITPCSFGGVSVITP
OU492255/P.1/Gamma	FNFNGLTGTGVLTESNKKFLPFQQFGRDIADTTDAVRDPQTLEILDITPCSFGGVSVITP
MZ911221/B.1.427/Epsilon	FNFNGLTGTGVLTESNKKFLPFQQFGRDIADTTDAVRDPQTLEILDITPCSFGGVSVITP
MZ375854/B.1.427/Epsilon	FNFNGLTGTGVLTESNKKFLPFQQFGRDIADTTDAVRDPQTLEILDITPCSFGGVSVITP
MZ375858/B.1.427/Epsilon	FNFNGLTGTGVLTESNKKFLPFQQFGRDIADTTDAVRDPQTLEILDITPCSFGGVSVITP
MZ943834/B.1.429/Epsilon	FNFNGLTGTGVLTESNKKFLPFQQFGRDIADTTDAVRDPQTLEILDITPCSFGGVSVITP
MZ277390/B.1.429/Epsilon	FNFNGLTGTGVLTESNKKFLPFQQFGRDIADTTDAVRDPQTLEILDITPCSFGGVSVITP
OU282717/B.1.429/Epsilon	FNFNGLTGTGVLTESNKKFLPFQQFGRDIADTTDAVRDPQTLEILDITPCSFGGVSVITP
OU235436/B.1.429/Epsilon	FNFNGLTGTGVLTESNKKFLPFQQFGRDIADTTDAVRDPQTLEILDITPCSFGGVSVITP
MZ687447/B.1.429/Epsilon	FNFNGLTGTGVLTESNKKFLPFQQFGRDIADTTDAVRDPQTLEILDITPCSFGGVSVITP
MZ538882/B.1.617.1/Kappa	FNFNGLTGTGVLTESNKKFLPFQQFGRDIADTTDAVRDPQTLEILDITPCSFGGVSVITP
MZ724425/B.1.617.1/Kappa	FNFNGLTGTGVLTESNKKFLPFQQFGRDIADTTDAVRDPQTLEILDITPCSFGGVSVITP
MZ363839/B.1.617.1/Kappa	FNFNGLTGTGVLTESNKKFLPFQQFGRDIADTTDAVRDPQTLEILDITPCSFGGVSVITP
MZ332524/B.1.617.1/Kappa	FNFNGLTGTGVLTESNKKFLPFQQFGRDIADTTDAVRDPQTLEILDITPCSFGGVSVITP
MZ332527/B.1.617.1/Kappa	FNFNGLTGTGVLTESNKKFLPFQQFGRDIADTTDAVRDPQTLEILDITPCSFGGVSVITP
LC633761/B.1.617.1/Kappa	FNFNGLTGTGVLTESNKKFLPFQQFGRDIADTTDAVRDPQTLEILDITPCSFGGVSVITP
MZ562746/B.1.617.1/Kappa	FNFNGLTGTGVLTESNKKFLPFQQFGRDIADTTDAVRDPQTLEILDITPCSFGGVSVITP
MZ571142/B.1.617.1/Kappa	FNFNGLTGTGVLTESNKKFLPFQQFGRDIADTTDAVRDPQTLEILDITPCSFGGVSVITP
OU168413/B.1.617.1/Kappa	FNFNGLTGTGVLTESNKKFLPFQQFGRDIADTTDAVRDPQTLEILDITPCSFGGVSVITP
MZ026886/B.1.617.1/Kappa	FNFNGLTGTGVLTESNKKFLPFQQFGRDIADTTDAVRDPQTLEILDITPCSFGGVSVITP
OU172184/B.1.617.1/Kappa	FNFNGLTGTGVLTESNKKFLPFQQFGRDIADTTDAVRDPQTLEILDITPCSFGGVSVITP
MZ397173/B.1.617.1/Kappa	FNFNGLTGTGVLTESNKKFLPFQQFGRDIADTTDAVRDPQTLEILDITPCSFGGVSVITP
MZ397176/B.1.617.1/Kappa	FNFNGLTGTGVLTESNKKFLPFQQFGRDIADTTDAVRDPQTLEILDITPCSFGGVSVITP
MZ397175/B.1.617.1/Kappa	FNFNGLTGTGVLTESNKKFLPFQQFGRDIADTTDAVRDPQTLEILDITPCSFGGVSVITP
MZ397177/B.1.617.1/Kappa	FNFNGLTGTGVLTESNKKFLPFQQFGRDIADTTDAVRDPQTLEILDITPCSFGGVSVITP
OU290035/B.1.617.1/Kappa	FNFNGLTGTGVLTESNKKFLPFQQFGRDIADTTDAVRDPQTLEILDITPCSFGGVSVITP
OU265178/B.1.617.1/Kappa	FNFNGLTGTGVLTESNKKFLPFQQFGRDIADTTDAVRDPQTLEILDITPCSFGGVSVITP
OU254949/B.1.617.1/Kappa	FNFNGLTGTGVLTESNKKFLPFQQFGRDIADTTDAVRDPQTLEILDITPCSFGGVSVITP
OU247018/B.1.617.1/Kappa	FNFNGLTGTGVLTESNKKFLPFQQFGRDIADTTDAVRDPQTLEILDITPCSFGGVSVITP
OU061269/B.1.617.1/Kappa	FNFNGLTGTGVLTESNKKFLPFQQFGRDIADTTDAVRDPQTLEILDITPCSFGGVSVITP
OU182966/B.1.617.1/Kappa	FNFNGLTGTGVLTESNKKFLPFQQFGRDIADTTDAVRDPQTLEILDITPCSFGGVSVITP
MZ746235/B.1.617.1/Kappa	FNFNGLTGTGVLTESNKKFLPFQQFGRDIADTTDAVRDPQTLEILDITPCSFGGVSVITP
MZ487800/B.1.617.1/Kappa	FNFNGLTGTGVLTESNKKFLPFQQFGRDIADTTDAVRDPQTLEILDITPCSFGGVSVITP
OU322235/B.1.617.1/Kappa	FNFNGLTGTGVLTESNKKFLPFQQFGRDIADTTDAVRDPQTLEILDITPCSFGGVSVITP
MZ953185/C.37/Lambda	FNFNGLTGTGVLTESNKKFLPFQQFGRDIADTTDAVRDPQTLEILDITPCSFGGVSVITP
MZ937666/C.37/Lambda	FNFNGLTGTGVLTESNKKFLPFQQFGRDIADTTDAVRDPQTLEILDITPCSFGGVSVITP
OU356036/C.37/Lambda	FNFNGLTGTGVLTESNKKFLPFQQFGRDIADTTDAVRDPQTLEILDITPCSFGGVSVITP
OU351967/C.37/Lambda	FNFNGLTGTGVLTESNKKFLPFQQFGRDIADTTDAVRDPQTLEILDITPCSFGGVSVITP
OU053018/C.37/Lambda	FNFNGLTGTGVLTESNKKFLPFQQFGRDIADTTDAVRDPQTLEILDITPCSFGGVSVITP
OU268528/C.37/Lambda	FNFNGLTGTGVLTESNKKFLPFQQFGRDIADTTDAVRDPQTLEILDITPCSFGGVSVITP
OU100999/C.37/Lambda	FNFNGLTGTGVLTESNKKFLPFQQFGRDIADTTDAVRDPQTLEILDITPCSFGGVSVITP
MZ275295/C.37/Lambda	FNFNGLTGTGVLTESNKKFLPFQQFGRDIADTTDAVRDPQTLEILDITPCSFGGVSVITP
MZ275301/C.37/Lambda	FNFNGLTGTGVLTESNKKFLPFQQFGRDIADTTDAVRDPQTLEILDITPCSFGGVSVITP
MZ908711/C.37/Lambda	FNFNGLTGTGVLTESNKKFLPFQQFGRDIADTTDAVRDPQTLEILDITPCSFGGVSVITP
MZ496613/C.37/Lambda	FNFNGLTGTGVLTESNKKFLPFQQFGRDIADTTDAVRDPQTLEILDITPCSFGGVSVITP
OU517038/C.37/Lambda	FNFNGLTGTGVLTESNKKFLPFQQFGRDIADTTDAVRDPQTLEILDITPCSFGGVSVITP
MZ953277/C.37/Lambda	FNFNGLTGTGVLTESNKKFLPFQQFGRDIADTTDAVRDPQTLEILDITPCSFGGVSVITP
MZ942542/C.37/Lambda	FNFNGLTGTGVLTESNKKFLPFQQFGRDIADTTDAVRDPQTLEILDITPCSFGGVSVITP
MZ857990/C.37/Lambda	FNFNGLTGTGVLTESNKKFLPFQQFGRDIADTTDAVRDPQTLEILDITPCSFGGVSVITP

```
                                   610       620       630       640       650       660
MN908947/Wuhan-Hu-1          GTNTSNQVAVLYQDVNCTEVPVAIHADQLTPTWRVYSTGSNVFQTRAGCLIGAEHVNNSY
BS001137/B.1.1.7/Alpha       GTNTSNQVAVLYQGVNCTEVPVAIHADQLTPTWRVYSTGSNVFQTRAGCLIGAEHVNNSY
BS001138/B.1.1.7/Alpha       GTNTSNQVAVLYQGVNCTEVPVAIHADQLTPTWRVYSTGSNVFQTRAGCLIGAEHVNNSY
BS001139/B.1.1.7/Alpha       GTNTSNQVAVLYQGVNCTEVPVAIHADQLTPTWRVYSTGSNVFQTRAGCLIGAEHVNNSY
BS001140/B.1.1.7/Alpha       GTNTSNQVAVLYQGVNCTEVPVAIHADQLTPTWRVYSTGSNVFQTRAGCLIGAEHVNNSY
MW913362/B.1.1.7/Alpha       GTNTSNQVAVLYQGVNCTEVPVAIHADQLTPTWRVYSTGSNVFQTRAGCLIGAEHVNNSY
MZ266365/B.1.1.7/Alpha       GTNTSNQVAVLYQGVNCTEVPVAIHADQLTPTWRVYSTGSNVFQTRAGCLIGAEHVNNSY
MZ350105/B.1.1.7/Alpha       GTNTSNQVAVLYQGVNCTEVPVAIHADQLTPTWRVYSTGSNVFQTRAGCLIGVEHVNNSY
MZ350109/B.1.1.7/Alpha       GTNTSNQVAVLYQGVNCTEVPVAIHADQLTPTWRVYSTGSNVFQTRAGCLIGAEHVNNSY
MZ888516/B.1.1.7/Alpha       GTNTSNQVAVLYQGVNCTEVPVAIHADQLTPTWRVYSTGSNVFQTRAGCLIGAEHVNNSY
OU562607/B.1.1.7/Alpha       GTNTSNQVAVLYQGVNCTEVPVAIHADQLTPTWRVYSTGSNVFQTRAGCLIGAEHVNNSY
OU514166/B.1.1.7/Alpha       GTNTSNQVAVLYQGVNCTEVPVAIHADQLTPTWRVYSTGSNVFQTRAGCLIGAEHVNNSY
OU538851/B.1.1.7/Alpha       GTNTSNQVAVLYQGVNCTEVPVAIHADQLTPTWRVYSTGSNVFQTRAGCLIGAEHVNNSY
OU538849/B.1.1.7/Alpha       GTNTSNQVAVLYQGVNCTEVPVAIHADQLTPTWRVYSTGSNVFQTRAGCLIGAEHVNNSY
MZ895877/B.1.1.7/Alpha       GTNTSNQVAVLYQGVNCTEVPVAIHADQLTPTWRVYSTGSNVFQTRAGCLIGAEHVNNSY
OU514176/B.1.1.7/Alpha       GTNTSNQVAVLYQGVNCTEVPVAIHADQLTPTWRVYSTGSNVFQTRAGCLIGAEHVNNSY
OU562608/B.1.1.7/Alpha       GTNTSNQVAVLYQGVNCTEVPVAIHADQLTPTWRVYSTGSNVFQTRAGCLIGAEHVNNSY
MW981442/B.1.351/Beta        GTNTSNQVAVLYQGVNCTEVPVAIHADQLTPTWRVYSTGSNVFQTRAGCLIGAEHVNNSY
MZ298839/B.1.351/Beta        GTNTSNQVAVLYQGVNCTEVPVAIHADQLTPTWRVYSTGSNVFQTRAGCLIGAEHVNNSY
MZ913100/B.1.351/Beta        GTNTSNQVAVLYQGVNCTEVPVAIHADQLTPTWRVYSTGSNVFQTRAGCLIGAEHVNNSY
MZ879365/B.1.351/Beta        GTNTSNQVAVLYQGVNCTEVPVAIHADQLTPTWRVYSTGSNVFQTRAGCLIGAEHVNNSY
OU525106/B.1.351/Beta        GTNTSNQVAVLYQGVNCTEVPVAIHADQLTPTWRVYSTGSNVFQTRAGCLIGAEHVNNSY
OU539352/B.1.351/Beta        GTNTSNQVAVLYQGVNCTEVPVAIHADQLTPTWRVYSTGSNVFQTRAGCLIGAEHVNNSY
MZ202314/B.1.351/Beta        GTNTSNQVAVLYQGVNCTEVPVAIHADQLTPTWRVYSTGSNVFQTRAGCLIGAEHVNNSY
MZ298840/B.1.351/Beta        GTNTSNQVAVLYQGVNCTEVPVAIHADQLTPTWRVYSTGSNVFQTRAGCLIGAEHVNNSY
MZ376663/B.1.351/Beta        GTNTSNQVAVLYQGVNCTEVPVAIHADQLTPTWRVYSTGSNVFQTRAGCLIGAEHVNNSY
LC643036/B.1.617.2/Delta     GTNTSNQVAVLYQGVNCTEVPVAIHADQLTPTWRVYSTGSNVFQTRAGCLIGAEHVNNSY
LC643044/B.1.617.2/Delta     GTNTSNQVAVLYQGVNCTEVPVAIHADQLTPTWRVYSTGSNVFQTRAGCLIGAEHVNNSY
MZ853946/B.1.617.2/Delta     GTNTSNQVAVLYQGVNCTEVPVAIHADQLTPTWRVYSTGSNVFQTRAGCLIGAEHVNNSY
MZ854386/B.1.617.2/Delta     GTNTSNQVAVLYQGVNCTEVPVAIHADQLTPTWRVYSTGSNVFQTRAGCLIGAEHVNNSY
MZ888532/B.1.617.2/Delta     GTNTSNQVAVLYQGVNCTEVPVAIHADQLTPTWRVYSTGSNVFQTRAGCLIGAEHVNNSY
MZ888533/B.1.617.2/Delta     GTNTSNQVAVLYQGVNCTEVPVAIHADQLTPTWRVYSTGSNVFQTRAGCLIGAEHVNNSY
MZ896136/B.1.617.2/Delta     GTNTSNQVAVLYQGVNCTEVPVAIHADQLTPTWRVYSTGSNVFQTRAGCLIGAEHVNNSY
OU539261/B.1.617.2/Delta     GTNTSNQVAVLYQGVNCTEVPVAIHADQLTPTWRVYSTGSNVFQTRAGCLIGAEHVNNSY
MZ854389/B.1.617.2/Delta     GTNTSNQVAVLYQGVNCTEVPVAIHADQLTPTWRVYSTGSNVFQTRAGCLIGAEHVNNSY
OU562629/B.1.617.2/Delta     GTNTSNQVAVLYQGVNCTEVPVAIHADQLTPTWRVYSTGSNVFQTRAGCLIGAEHVNNSY
OU539690/B.1.617.2/Delta     GTNTSNQVAVLYQGVNCTEVPVAIHADQLTPTWRVYSTGSNVFQTRAGCLIGAEHVNNSY
MZ854387/B.1.617.2/Delta     GTNTSNQVAVLYQGVNCTEVPVAIHADQLTPTWRVYSTGSNVFQTRAGCLIGAEHVNNSY
OU539784/P.1/Gamma           GTNTSNQVAVLYQGVNCTEVPVAIHADQLTPTWRVYSTGSNVFQTRAGCLIGAEYVNNSY
OU539830/P.1/Gamma           GTNTSNQVAVLYQGVNCTEVPVAIHADQLTPTWRVYSTGSNVFQTRAGCLIGAEYVNNSY
OU539894/P.1/Gamma           GTNTSNQVAVLYQGVNCTEVPVAIHADQLTPTWRVYSTGSNVFQTRAGCLIGAEYVNNSY
MZ477748/P.1/Gamma           GTNTSNQVAVLYQGVNCTEVPVAIHADQLTPTWRVYSTGSNVFQTRAGCLIGAEYVNNSY
MZ477746/P.1/Gamma           GTNTSNQVAVLYQGVNCTEVPVAIHADQLTPTWRVYSTGSNVFQTRAGCLIGAEYVNNSY
MW938104/P.1/Gamma           GTNTSNQVAVLYQGVNCTEVPVAIHADQLTPTWRVYSTGSNVFQTRAGCLIGAEYVNNSY
MZ896179/P.1/Gamma           GTNTSNQVAVLYQGVNCTEVPVAIHADQLTPTWRVYSTGSNVFQTRAGCLIGAEYVNNSY
MZ896359/P.1/Gamma           GTNTSNQVAVLYQGVNCTEVPVAIHADQLTPTWRVYSTGSNVFQTRAGCLIGAEYVNNSY
MZ896669/P.1/Gamma           GTNTSNQVAVLYQGVNCTEVPVAIHADQLTPTWRVYSTGSNVFQTRAGCLIGAEYVNNSY
OU565182/P.1/Gamma           GTNTSNQVAVLYQGVNCTEVPVAIHADQLTPTWRVYSTGFNVFQTRAGCLIGAEYVNNSY
OU492255/P.1/Gamma           GTNTSNQVAVLYQGVNCTEVPVAIHADQLTPTWRVYSTGFNVFQTRAGCLIGAEYVNNSY
MZ911221/B.1.427/Epsilon     GTNTSNQVAVLYQGVNCTEVPVAIHADQLTPTWRVYSTGSNVFQTRAGCLIGAEHVNNSY
MZ375854/B.1.427/Epsilon     GTNTSNQVAVLYQGVNCTEVPVAIHADQLTPTWRVYSTGSNVFQTRAGCLIGAEHVNNSY
MZ375858/B.1.427/Epsilon     GTNTSNQVAVLYQGVNCTEVPVAIHADQLTPTWRVYSTGSNVFQTRAGCLIGAEHVNNSY
MZ943834/B.1.429/Epsilon     GTNTSNQVAVLYQGVNCTEVPVAIHADQLTPTWRVYSTGSNVFQTRAGCLIGAEHVNNSY
MZ277390/B.1.429/Epsilon     GTNTSNQVAVLYQGVNCTEVPVAIHADQLTPTWRVYSTGSNVFQTRAGCLIGAEHVNNSY
OU282717/B.1.429/Epsilon     GTNTSNQVAVLYQGVNCTEVPVAIHADQLTPTWRVYSTGSNVFQTRAGCLIGAEHVNNSY
OU235436/B.1.429/Epsilon     GTNTSNQVAVLYQGVNCTEVPVAIHADQLTPTWRVYSTGSNVFQTRAGCLIGAEHVNNSY
MZ687447/B.1.429/Epsilon     GTNTSNQVAVLYQGVNCTEVPVAIHADQLTPTWRVYSTGSNVFQTRAGCLIGAEHVNNSY
MZ538882/B.1.617.1/Kappa     GTNTSNQVAVLYQGVNCTEVPVAIHADQLTPTWRVYSTGSNVFQTRAGCLIGAEHVNNSY
MZ724425/B.1.617.1/Kappa     GTNTSNQVAVLYQGVNCTEVPVAIHADQLTPTWRVYSTGSNVFQTRAGCLIGAEHVNNSY
MZ363839/B.1.617.1/Kappa     GTNTSNQVAVLYQGVNCTEVPVAIHADQLTPTWRVYSTGSNVFQTRAGCLIGAEHVNNSY
MZ332524/B.1.617.1/Kappa     GTNTSNQVAVLYQGVNCTEVPVAIHADQLTPTWRVYSTGSNVFQTRAGCLIGAEHVNNSY
MZ332527/B.1.617.1/Kappa     GTNTSNQVAVLYQGVNCTEVPVAIHADQLTPTWRVYSTGSNVFQTRAGCLIGAEHVNNSY
LC633761/B.1.617.1/Kappa     GTNTSNQVAVLYQGVNCTEVPVAIHADQLTPTWRVYSTGSNVFQTRAGCLIGAEHVNNSY
MZ562746/B.1.617.1/Kappa     GTNTSNQVAVLYQGVNCTEVPVAIHADQLTPTWRVYSTGSNVFQTRAGCLIGAEHVNNSY
MZ571142/B.1.617.1/Kappa     GTNTSNQVAVLYQGVNCTEVPVAIHADQLTPTWRVYSTGSNVFQTRAGCLIGAEHVNNSY
OU168413/B.1.617.1/Kappa     GTNTSNQVAVLYQGVNCTEVPVAIHADQLTPTWRVYSTGSNVFQTRAGCLIGAEHVNNSY
MZ026886/B.1.617.1/Kappa     GTNTSNQVAVLYQGVNCTEVPVAIHADQLTPTWRVYSTGSNVFQTRAGCLIGAEHVNNSY
OU172184/B.1.617.1/Kappa     GTNTSNQVAVLYQGVNCTEVPVAIHADQLTPTWRVYSTGSNVFQTRAGCLIGAEHVNNSY
MZ397173/B.1.617.1/Kappa     GTNTSNQVAVLYQGVNCTEVPVAIHADQLTPTWRVYSTGSNVFQTRAGCLIGAEHVNNSY
MZ397176/B.1.617.1/Kappa     GTNTSNQVAVLYQGVNCTEVPVAIHADQLTPTWRVYSTGSNVFQTRAGCLIGAEHVNNSY
MZ397175/B.1.617.1/Kappa     GTNTSNQVAVLYQGVNCTEVPVAIHADQLTPTWRVYSTGSNVFQTRAGCLIGAEHVNNSY
MZ397177/B.1.617.1/Kappa     GTNTSNQVAVLYQGVNCTEVPVAIHADQLTPTWRVYSTGSNVFQTRAGCLIGAEHVNNSY
OU290035/B.1.617.1/Kappa     GTNTSNQVAVLYQGVNCTEVPVAIHADQLTPTWRVYSTGSNVFQTRAGCLIGAEHVNNSY
OU265178/B.1.617.1/Kappa     GTNTSNQVAVLYQGVNCTEVPVAIHADQLTPTWRVYSTGSNVFQTRAGCLIGAEHVNNSY
OU254949/B.1.617.1/Kappa     GTNTSNQVAVLYQGVNCTEVPVAIHADQLTPTWRVYSTGSNVFQTRAGCLIGAEHVNNSY
OU247018/B.1.617.1/Kappa     GTNTSNQVAVLYQGVNCTEVPVAIHADQLTPTWRVYSTGSNVFQTRAGCLIGAEHVNNSY
OU061269/B.1.617.1/Kappa     GTNTSNQVAVLYQGVNCTEVPVAIHADQLTPTWRVYSTGSNVFQTRAGCLIGAEHVNNSY
OU182966/B.1.617.1/Kappa     GTNTSNQVAVLYQGVNCTEVPVAIHADQLTPTWRVYSTGSNVFQTRAGCLIGAEHVNNSY
MZ746235/B.1.617.1/Kappa     GTNTSNQVAVLYQGVNCTEVPVAIHADQLTPTWRVYSTGSNVFQTRAGCLIGAEHVNNSY
MZ487800/B.1.617.1/Kappa     GTNTSNQVAVLYQGVNCTEVPVAIHADQLTPTWRVYSTGSNVFQTRAGCLIGAEHVNNSY
OU322235/B.1.617.1/Kappa     GTNTSNQVAVLYQGVNCTEVPVAIHADQLTPTWRVYSTGSNVFQTRAGCLIGAEHVNNSY
MZ953185/C.37/Lambda         GTNTSNQVAVLYQGVNCTEVPVAIHADQLTPTWRVYSTGSNVFQTRAGCLIGAEHVNNSY
MZ937666/C.37/Lambda         GTNTSNQVAVLYQGVNCTEVPVAIHADQLTPTWRVYSTGSNVFQTRAGCLIGAEHVNNSY
OU356036/C.37/Lambda         GTNTSNQVAVLYQGVNCTEVPVAIHADQLTPTWRVYSTGSNVFQTRAGCLIGAEHVNNSY
OU351967/C.37/Lambda         GTNTSNQVAVLYQGVNCTEVPVAIHADQLTPTWRVYSTGSNVFQTRAGCLIGAEHVNNSY
OU053018/C.37/Lambda         GTNTSNQVAVLYQGVNCTEVPVAIHADQLTPTWRVYSTGSNVFQTRAGCLIGAEHVNNSY
OU268528/C.37/Lambda         GTNTSNQVAVLYQGVNCTEVPVAIHADQLTPTWRVYSTGFNVFQTRAGCLIGAEHVNNSY
OU100999/C.37/Lambda         GTNTSNQVAVLYQGVNCTEVPVAIHADQLTPTWRVYSTGSNVFQTRAGCLIGAEHVNNSY
MZ275295/C.37/Lambda         GTNTSNQVAVLYQGVNCTEVPVAIHADQLTPTWRVYSTGSNVFQTRAGCLIGAEHVNNSY
MZ275301/C.37/Lambda         GTNTSNQVAVLYQGVNCTEVPVAIHADQLTPTWRVYSTGSNVFQTRAGCLIGAEHVNNSY
MZ908711/C.37/Lambda         GTNTSNQVAVLYQGVNCTEVPVAIHADQLTPTWRVYSTGSNVFQTRAGCLIGAEHVNNSY
MZ496613/C.37/Lambda         GTNTSNQVAVLYQGVNCTEVPVAIHADQLTPTWRVYSTGSNVFQTRAGCLIGAEHVNNSY
OU517038/C.37/Lambda         GTNTSNQVAVLYQGVNCTEVPVAIHADQLTPTWRVYSTGSNVFQTRAGCLIGAEHVNNSY
MZ953277/C.37/Lambda         GTNTSNQVAVLYQGVNCTEVPVAIHADQLTPTWRVYSTGSNVFQTRAGCLIGAEHVNNSY
MZ942542/C.37/Lambda         GTNTSNQVAVLYQGVNCTEVPVAIHADQLTPTWRVYSTGSNVFQTRAGCLIGAEHVNNSY
MZ857990/C.37/Lambda         GTNTSNQVAVLYQGVNCTEVPVAIHADQLTPTWRVYSTGSNVFQTRAGCLIGAEHVNNSY
```

```
                                  670       680       690       700       710       720
                                    .         .         .         .         .         .
MN908947/Wuhan-Hu-1          ECDIPIGAGICASYQTQTNSPRRARSVASQSIIAYTMSLGAENSVAYSNNSIAIPTNFTI
BS001137/B.1.1.7/Alpha       ECDIPIGAGICASYQTQTNSHRRARSVASQSIIAYTMSLGAENSVAYSNNSIAIPINFTI
BS001138/B.1.1.7/Alpha       ECDIPIGAGICASYQTQTNSHRRARSVASQSIIAYTMSLGAENSVAYSNNSIAIPINFTI
BS001139/B.1.1.7/Alpha       ECDIPIGAGICASYQTQTNSHRRARSVASQSIIAYTMSLGAENSVAYSNNSIAIPINFTI
BS001140/B.1.1.7/Alpha       ECDIPIGAGICASYQTQTNSHRRARSVASQSIIAYTMSLGAENSVAYSNNSIAIPINFTI
MW913362/B.1.1.7/Alpha       ECDIPIGAGICASYQTQTNSHRRARSVASQSIIAYTMSLGAENSVAYSNNSIAIPINFTI
MZ266365/B.1.1.7/Alpha       ECDIPIGAGICASYQTQTNSHRRARSVASQSIIAYTMSLGAENSVAYSNNSIAIPINFTI
MZ350105/B.1.1.7/Alpha       ECDIPIGAGICASYQTQTNSHRRARSV..QSIIAYTMSLGAENSVAYSNNSIAIPINFTI
MZ350109/B.1.1.7/Alpha       ECDIPIGAGICASYQTQTNSHRRARSVASQSIIAYTMSLGAENSVAYSNNSIAIPINFTI
MZ888516/B.1.1.7/Alpha       ECDIPIGAGICASYQTQTNSHRRARSVASQSIIAYTMSLGAENSVAYSNNSIAIPINFTI
OU562607/B.1.1.7/Alpha       ECDIPIGAGICASYQTQTNSHRRARSVASQSIIAYTMSLGAENSVAYSNNSIAIPINFTI
OU514166/B.1.1.7/Alpha       ECDIPIGAGICASYQTQTNSHRRARSVASQSIIAYTMSLGAENSVAYSNNSIAIPINFTI
OU538851/B.1.1.7/Alpha       ECDIPIGAGICASYQTQTNSHRRARSVASQSIIAYTMSLGAENSVAYSNNSIAIPINFTI
OU538849/B.1.1.7/Alpha       ECDIPIGAGICASYQTQTNSHRRARSVASQSIIAYTMSLGAENSVAYSNNSIAIPINFTI
MZ895877/B.1.1.7/Alpha       ECDIPIGAGICASYQTQTNSHRRARSVASQSIIAYTMSLGAENSVAYSNNSIAIPINFTI
OU514176/B.1.1.7/Alpha       ECDIPIGAGICASYQTQTNSHRRARSVASQSIIAYTMSLGAENSVAYSNNSIAIPINFTI
OU562608/B.1.1.7/Alpha       ECDIPIGAGICASYQTQTNSHRRARSVASQSIIAYTMSLGAENSVAYSNNSIAIPINFTI
MW981442/B.1.351/Beta        ECDIPIGAGICASYQTHTNSPWRARSVASQSIIAYTMSLGVENSVAYSNNSIAIPTNFTI
MZ298839/B.1.351/Beta        ECDIPIGAGICASYQTQTNSPRRARSVASQSIIAYTMSLGVENSVAYSNNSIAIPTNFTI
MZ913100/B.1.351/Beta        ECDIPIGAGICASYQTQTNSPRRARSVASQSIIAYTMSLGVENSVAYSNNSIAIPTNFTI
MZ879365/B.1.351/Beta        ECDIPIGAGICASYQTQTNSPRRARSVASQSIIAYTMSLGVENSVAYSNNSIAIPTNFTI
OU525106/B.1.351/Beta        ECDIPIGAGICASYQTQTNSPRRARSVASQSIIAYTMSLGVENSVAYSNNSIAIPTNFTI
OU539352/B.1.351/Beta        ECDIPIGAGICASYQTQTNSPRRARSVASQSIIAYTMSLGVENSVAYSNNSIAIPTNFTI
MZ202314/B.1.351/Beta        ECDIPIGAGICASYQTHTNSPWRARSVASQSIIAYTMSLGVENSVAYSNNSIAIPTNFTI
MZ298840/B.1.351/Beta        ECDIPIGAGICASYQTQTNSPRRARSVASQSIIAYTMSLGVENSVAYSNNSIAIPTNFTI
MZ376663/B.1.351/Beta        ECDIPIGAGICASYQTHTNSPWRARSVASQSIIAYTMSLGVENSVAYSNNSIAIPTNFTI
LC643036/B.1.617.2/Delta     ECDIPIGAGICASYQTQTNSRRRARSVASQSIIAYTMSLGAENSVAYSNNSIAIPTNFTI
LC643044/B.1.617.2/Delta     ECDIPIGAGICASYQTQTNSRRRARSVASQSIIAYTMSLGAENSVAYSNNSIAIPTNFTI
MZ853946/B.1.617.2/Delta     ECDIPIGAGICASYQTQTNSRRRARSVASQSIIAYTMSLGAENSVAYSNNSIAIPTNFTI
MZ854386/B.1.617.2/Delta     ECDIPIGAGICASYQTQTNSRRRARSVASQSIIAYTMSLGAENSVAYSNNSIAIPTNFTI
MZ888532/B.1.617.2/Delta     ECDIPIGAGICASYQTQTNSRRRARSVASQSIIAYTMSLGAENSVAYSNNSIAIPTNFTI
MZ888533/B.1.617.2/Delta     ECDIPIGAGICASYQTQTNSRRRARSVASQSIIAYTMSLGAENSVAYSNNSIAIPTNFTI
MZ896136/B.1.617.2/Delta     ECDIPIGAGICASYQTQTNSRRRARSVASQSIIAYTMSLGAENSVAYSNNSIAIPTNFTI
OU539261/B.1.617.2/Delta     ECDIPIGAGICASYQTQTNSRRRARSVASQSIIAYTMSLGAENSVAYSNNSIAIPTNFTI
MZ854389/B.1.617.2/Delta     ECDIPIGAGICASYQTQTNSRRRARSVASQSIIAYTMSLGAENSVAYSNNSIAIPTNFTI
OU562629/B.1.617.2/Delta     ECDIPIGAGICASYQTQTNSRRRARSVASQSIIAYTMSLGAENSVAYSNNSIAIPTNFTI
OU539690/B.1.617.2/Delta     ECDIPIGAGICASYQTQTNSRRRARSVASQSIIAYTMSLGAENSVAYSNNSIAIPTNFTI
MZ854387/B.1.617.2/Delta     ECDIPIGAGICASYQTQTNSRRRARSVASQSIIAYTMSLGAENSVAYSNNSIAIPTNFTI
OU539784/P.1/Gamma           ECDIPIGAGICASYQTQTNSPRRARSVASQSIIAYTMSLGAENSVAYSNNSIAIPTNFTI
OU539830/P.1/Gamma           ECDIPIGAGICASYQTQTNSPRRARSVASQSIIAYTMSLGAENSVAYSNNSIAIPTNFTI
OU539894/P.1/Gamma           ECDIPIGAGICASYQTQTNSPRRARSVASQSIIAYTMSLGAENSVAYSNNSIAIPTNFTI
MZ477748/P.1/Gamma           ECDIPIGAGICASYQTQTNSPRRARSVASQSIIAYTMSLGAENSVAYSNNSIAIPTNFTI
MZ477746/P.1/Gamma           ECDIPIGAGICASYQTQTNSPRRARSVASQSIIAYTMSLGAENSVAYSNNSIAIPTNFTI
MW938104/P.1/Gamma           ECDIPIGAGICASYQTQTNSPRRARSVASQSIIAYTMSLGAENSVAYSNNSIAIPTNFTI
MZ896179/P.1/Gamma           ECDIPIGAGICASYQTQTNSPRRARSVASQSIIAYTMSLGAENSVAYSNNSIAIPTNFTI
MZ896359/P.1/Gamma           ECDIPIGAGICASYQTQTNSPRRARSVASQSIIAYTMSLGAENSVAYSNNSIAIPTNFTI
MZ896669/P.1/Gamma           ECDIPIGAGICASYQTQTNSPRRARSVASQSIIAYTMSLGAENSVAYSNNSIAIPTNFTI
OU565182/P.1/Gamma           ECDIPIGAGICASYQTQTNSPRRARSVASQSIIAYTMSLGAENSVAYSNNSIAIPTNFTI
OU492255/P.1/Gamma           ECDIPIGAGICASYQTQTNSPRRARSVASQSIIAYTMSLGAENSVAYSNNSIAIPTNFTI
MZ911221/B.1.427/Epsilon     ECDIPIGAGICASYQTQTNSPRRARSVASQSIIAYTMSLGAENSVAYSNNSIAIPTNFTI
MZ375854/B.1.427/Epsilon     ECDIPIGAGICASYQTQTNSPRRARSVASQSIIAYTMSLGAENSVAYSNNSIAIPTNFTI
MZ375858/B.1.427/Epsilon     ECDIPIGAGICASYQTQTNSHRRARSVASQSIIAYTMSLGAENSVAYSNNSIAIPTNFTI
MZ943834/B.1.429/Epsilon     ECDIPIGAGICASYQTQTNSPRRARSVASQSIIAYTMSLGAENSVAYSNNSIAIPTNFTI
MZ277390/B.1.429/Epsilon     ECDIPIGAGICASYQTQTNSPRRARSVASQSIIAYTMSLGAENSVAYSNNSIAIPTNFTI
OU282717/B.1.429/Epsilon     ECDIPIGAGICASYQTQTNSPRRARSVASQSIIAYTMSLGAENSVAYSNNSIAIPTNFTI
OU235436/B.1.429/Epsilon     ECDIPIGAGICASYQTQTNSPRRARSVASQSIIAYTMSLGAENSVAYSNNSIAIPTNFTI
MZ687447/B.1.429/Epsilon     ECDIPIGAGICASYQTQTNSPRRARSVASQSIIAYTMSLGAENSVAYSNNSIAIPTNFTI
MZ538882/B.1.617.1/Kappa     ECDIPIGAGICASYQTQTNSRRRARSVASQSIIAYTMSLGAENSVAYSNNSIAIPTNFTI
MZ724425/B.1.617.1/Kappa     ECDIPIGAGICASYQTQTNSRRRARSVASQSIIAYTMSLGAENSVAYSNNSIAIPTNFTI
MZ363839/B.1.617.1/Kappa     ECDIPIGAGICASYQTQTNSRRRARSVASQSIIAYTMSLGAENSVAYSNNSIAIPTNFTI
MZ332524/B.1.617.1/Kappa     ECDIPIGAGICASYQTQTNSRRRARSVASQSIIAYTMSLGAENSVAYSNNSIAIPTNFTI
MZ332527/B.1.617.1/Kappa     ECDIPIGAGICASYQTQTNSRRRARSVASQSIIAYTMSLGAENSVAYSNNSIAIPTNFTI
LC633761/B.1.617.1/Kappa     ECDIPIGAGICASYQTQTNSRRRARSVASQSIIAYTMSLGAENSVAYSNNSIAIPTNFTI
MZ562746/B.1.617.1/Kappa     ECDIPIGAGICASYQTQTNSRRRARSVASQSIIAYTMSLGAENSVAYSNNSIAIPTNFTI
MZ571142/B.1.617.1/Kappa     ECDIPIGAGICASYQTQTNSRRRARSVASQSIIAYTMSLGAENSVAYSNNSIAIPTNFTI
OU168413/B.1.617.1/Kappa     ECDIPIGAGICASYQTQTNSRRRARSVASQSIIAYTMSLGAENSVAYSNNSIAIPTNFTI
MZ026886/B.1.617.1/Kappa     ECDIPIGAGICASYQTQTNSRRRARSVASQSIIAYTMSLGAENSVAYSNNSIAIPTNFTI
OU172184/B.1.617.1/Kappa     ECDIPIGAGICASYQTQTNSRRRARSVASQSIIAYTMSLGAENSVAYSNNSIAIPTNFTI
MZ397173/B.1.617.1/Kappa     ECDIPIGAGICASYQTQTNSRRRARSVASQSIIAYTMSLGAENSVAYSNNSIAIPTNFTI
MZ397176/B.1.617.1/Kappa     ECDIPIGAGICASYQTQTNSRRRARSVASQSIIAYTMSLGAENSVAYSNNSIAIPTNFTI
MZ397175/B.1.617.1/Kappa     ECDIPIGAGICASYQTQTNSRRRARSVASQSIIAYTMSLGAENSVAYSNNSIAIPTNFTI
MZ397177/B.1.617.1/Kappa     ECDIPIGAGICASYQTQTNSRRRARSVASQSIIAYTMSLGAENSVAYSNNSIAIPTNFTI
OU290035/B.1.617.1/Kappa     ECDIPIGAGICASYQTQTNSRRRARSVASQSIIAYTMSLGAENSVAYSNNSIAIPTNFTI
OU265178/B.1.617.1/Kappa     ECDIPIGAGICASYQTQTNSRRRARSVASQSIIAYTMSLGAENSVAYSNNSIAIPTNFTI
OU254949/B.1.617.1/Kappa     ECDIPIGAGICASYQTQTNSRRRARSVASQSIIAYTMSLGAENSVAYSNNSIAIPTNFTI
OU247018/B.1.617.1/Kappa     ECDIPIGAGICASYQTQTNSRRRARSVASQSIIAYTMSLGAENSVAYSNNSIAIPTNFTI
OU061269/B.1.617.1/Kappa     ECDIPIGAGICASYQTQTNSRRRARSVASQSIIAYTMSLGAENSVAYSNNSIAIPTNFTI
OU182966/B.1.617.1/Kappa     ECDIPIGAGICASYQTQTNSRRRARSVASQSIIAYTMSLGAENSVAYSNNSIAIPTNFTI
MZ746235/B.1.617.1/Kappa     ECDIPIGAGICASYQTQTNSRRRARSVASQSIIAYTMSLGAENSVAYSNNSIAIPTNFTI
MZ487800/B.1.617.1/Kappa     ECDIPIGAGICASYQTQTNSRRRARSVASQSIIAYTMSLGAENSVAYSNNSIAIPTNFTI
OU322235/B.1.617.1/Kappa     ECDIPIGAGICASYQTQTNSRRRARSVASQSIIAYTMSLGAENSVAYSNNSIAIPTNFTI
MZ953185/C.37/Lambda         ECDIPIGAGICASYQTQTNSPRRARSVASQSIIAYTMSLGAENSVAYSNNSIAIPTNFTI
MZ937666/C.37/Lambda         ECDIPIGAGICASYQTQTNSPRRARSVASQSIIAYTMSLGAENSVAYSNNSIAIPTNFTI
OU356036/C.37/Lambda         ECDIPIGAGICASYQTQTNSPRRARSVASQSIIAYTMSLGAENSVAYSNNSIAIPTNFTI
OU351967/C.37/Lambda         ECDIPIGAGICASYQTQTNSPRRARSVASQSIIAYTMSLGAENSVAYSNNSIAIPTNFTI
OU053018/C.37/Lambda         ECDIPIGAGICASYQTQTNSPRRARSVASQSIIAYTMSLGAENSVAYSNNSIAIPTNFTI
OU268528/C.37/Lambda         ECDIPIGAGICASYQTQTNSPRRARSVASQSIIAYTMSLGAENSVAYSNNSIAIPTNFTI
OU100999/C.37/Lambda         ECDIPIGAGICASYQTQTNSPRRARSVASQSIIAYTMSLGAENSVAYSNNSIAVPTNFTI
MZ275295/C.37/Lambda         ECDIPIGAGICASYQTQTNSPRRARSVASQSIIAYTMSLGAENSVAYSNNSIAIPTNFTI
MZ275301/C.37/Lambda         ECDIPIGAGICASYQTQTNSPRRARSVASQSIIAYTMSLGAENSVAYSNNSIAIPTNFTI
MZ908711/C.37/Lambda         ECDIPIGAGICASYQTQTNSPRRARSVASQSIIAYTMSLGAENSVAYSNNSIAIPTNFTI
MZ496613/C.37/Lambda         ECDIPIGAGICASYQTQTNSPRRARSVASQSIIAYTMSLGAENSVAYSNNSIAIPTNFTI
OU517038/C.37/Lambda         ECDIPIGAGICASYQTQTNSPRRARSVASQSIIAYTMSLGAENSVAYSNNSIAIPTNFTI
MZ953277/C.37/Lambda         ECDIPIGAGICASYQTQTNSPRRARSVASQSIIAYTMSLGAENSVAYSNNSIAIPTNFTI
MZ942542/C.37/Lambda         ECDIPIGAGICASYQTQTNSPRRARSVASQSIIAYTMSLGAENSVAYSNNSIAIPTNFTI
MZ857990/C.37/Lambda         ECDIPIGAGICASYQTQTNSPRRARSVASQSIIAYTMSLGAENSVAYSNNSIAIPTNFTI
```

```
                                              730       740       750       760       770       780
MN908947/Wuhan-Hu-1          SVTTEILPVSMTKTSVDCTMYICGDSTECSNLLLQYGSFCTQLNRALTGIAVEQDKNTQE
BS001137/B.1.1.7/Alpha       SVTTEILPVSMTKTSVDCTMYICGDSTECSNLLLQYGSFCTQLNRALTGIAVEQDKNTQE
BS001138/B.1.1.7/Alpha       SVTTEILPVSMTKTSVDCTMYICGDSTECSNLLLQYGSFCTQLNRALTGIAVEQDKNTQE
BS001139/B.1.1.7/Alpha       SVTTEILPVSMTKTSVDCTMYICGDSTECSNLLLQYGSFCTQLNRALTGIAVEQDKNTQE
BS001140/B.1.1.7/Alpha       SVTTEILPVSMTKTSVDCTMYICGDSTECSNLLLQYGSFCTQLNRALTGIAVEQDKNTQE
MW913362/B.1.1.7/Alpha       SVTTEILPVSMTKTSVDCTMYICGDSTECSNLLLQYGSFCTQLNRALTGIAVEQDKNTQE
MZ266365/B.1.1.7/Alpha       SVTTEILPVSMTKTSVDCTMYICGDSTECSNLLLQYGSFCTQLNRALTGIAVEQDKNTQE
MZ350105/B.1.1.7/Alpha       SVTTEILPVSMTKTSVDCTMYICGDSTECSNLLLQYGSFCTQLNRALTGIAVEQDKNTQE
MZ350109/B.1.1.7/Alpha       SVTTEILPVSMTKTSVDCTMYICGDSTECSNLLLQYGSFCTQLNRALTGIAVEQDKNTQE
MZ888516/B.1.1.7/Alpha       SVTTEILPVSMTKTSVDCTMYICGDSTECSNLLLQYGSFCTQLNRALTGIAVEQDKNTQE
OU562607/B.1.1.7/Alpha       SVTTEILPVSMTKTSVDCTMYICGDSTECSNLLLQYGSFCTQLNRALTGIAVEQDKNTQE
OU514166/B.1.1.7/Alpha       SVTTEILPVSMTKTSVDCTMYICGDSTECSNLLLQYGSFCTQLNRALTGIAVEQDKNTQE
OU538851/B.1.1.7/Alpha       SVTTEILPVSMTKTSVDCTMYICGDSTECSNLLLQYGSFCTQLNRALTGIAVEQDKNTQE
OU538849/B.1.1.7/Alpha       SVTTEILPVSMTKTSVDCTMYICGDSTECSNLLLQYGSFCTQLNRALTGIAVEQDKNTQE
MZ895877/B.1.1.7/Alpha       SVTTEILPVSMTKTSVDCTMYICGDSTECSNLLLQYGSFCTQLNRALTGIAVEQDKNTQE
OU514176/B.1.1.7/Alpha       SVTTEILPVSMTKTSVDCTMYICGDSTECSNLLLQYGSFCTQLNRALTGIAVEQDKNTQE
OU562608/B.1.1.7/Alpha       SVTTEILPVSMTKTSVDCTMYICGDSTECSNLLLQYGSFCTQLNRALTGIAVEQDKNTQE
MW981442/B.1.351/Beta        SVTTEILPVSMTKTSVDCTMYICGDSTECSNLLLQYGSFCTQLNRALTGIAVEQDKNTQE
MZ298839/B.1.351/Beta        SVTTEILPVSMTKTSVDCTMYICGDSTECSNLLLQYGSFCTQLNRALTGIAVEQDKNTQE
MZ913100/B.1.351/Beta        SVTTEILPVSMTKTSVDCTMYICGDSTECSNLLLQYGSFCTQLNRALTGIAVEQDKNTQE
MZ879365/B.1.351/Beta        SVTTEILPVSMTKTSVDCTMYICGDSTECSNLLLQYGSFCTQLNRALTGIAVEQDKNTQE
OU525106/B.1.351/Beta        SVTTEILPVSMTKTSVDCTMYICGDSTECSNLLLQYGSFCTQLNRALTVIAVEQDKNTQE
OU539352/B.1.351/Beta        SVTTEILPVSMTKTSVDCTMYICGDSTECSNLLLQYGSFCTQLNRALTGIAVEQDKNTQE
MZ202314/B.1.351/Beta        SVTTEILPVSMTKTSVDCTMYICGDSTECSNLLLQYGSFCTQLNRALTGIAVEQDKNTQE
MZ298840/B.1.351/Beta        SVTTEILPVSMTKTSVDCTMYICGDSTECSNLLLQYGSFCTQLNRALTGIAVEQDKNTQE
MZ376663/B.1.351/Beta        SVTTEILPVSMTKTSVDCTMYICGDSTECSNLLLQYGSFCTQLNRALTGIAVEQDKNTQE
LC643036/B.1.617.2/Delta     SVTTEILPVSMTKTSVDCTMYICGDSTECSNLLLQYGSFCTQLNRALTGIAVEQDKNTQE
LC643044/B.1.617.2/Delta     SVTTEILPVSMTKTSVDCTMYICGDSTECSNLLLQYGSFCTQLNRALTGIAVEQDKNTQE
MZ853946/B.1.617.2/Delta     SVTTEILPVSMTKTSVDCTMYICGDSTECSNLLLQYGSFCTQLNRALTGIAVEQDKNTQE
MZ854386/B.1.617.2/Delta     SVTTEILPVSMTKTSVDCTMYICGDSTECSNLLLQYGSFCTQLNRALTGIAVEQDKNTQE
MZ888532/B.1.617.2/Delta     SVTTEILPVSMTKTSVDCTMYICGDSTECSNLLLQYGSFCTQLNRALTGIAVEQDKNTQE
MZ888533/B.1.617.2/Delta     SVTTEILPVSMTKTSVDCTMYICGDSTECSNLLLQYGSFCTQLNRALTGIAVEQDKNTQE
MZ896136/B.1.617.2/Delta     SVTTEILPVSMTKTSVDCTMYICGDSTECSNLLLQYGSFCTQLNRALTGIAVEQDKNTQE
OU539261/B.1.617.2/Delta     SVTTEILPVSMTKTSVDCTMYICGDSTECSNLLLQYGSFCTQLNRALTGIAVEQDKNTQE
MZ854389/B.1.617.2/Delta     SVTTEILPVSMTKTSVDCTMYICGDSTECSNLLLQYGSFCTQLNRALTGIAVEQDKNTQE
OU562629/B.1.617.2/Delta     SVTTEILPVSMTKTSVDCTMYICGDSTECSNLLLQYGSFCTQLNRALTGIAVEQDKNTQE
OU539690/B.1.617.2/Delta     SVTTEILPVSMTKTSVDCTMYICGDSTECSNLLLQYGSFCTQLNRALTGIAVEQDKNTQE
MZ854387/B.1.617.2/Delta     SVTTEILPVSMTKTSVDCTMYICGDSTECSNLLLQYGSFCTQLNRALTGIAVEQDKNTQE
OU539784/P.1/Gamma           SVTTEILPVSMTKTSVDCTMYICGDSTECSNLLLQYGSFCTQLNRALTGIAVEQDKNTQE
OU539830/P.1/Gamma           SVTTEILPVSMTKTSVDCTMYICGDSTECSNLLLQYGSFCTQLNRALTGIAVEQDKNTQE
OU539894/P.1/Gamma           SVTTEILPVSMTKTSVDCTMYICGDSTECSNLLLQYGSFCTQLNRALTGIAVEQDKNTQE
MZ477748/P.1/Gamma           SVTTEILPVSMTKTSVDCTMYICGDSTECSNLLLQYGSFCTQLNRALTGIAVEQDKNTQE
MZ477746/P.1/Gamma           SVTTEILPVSMTKTSVDCTMYICGDSTECSNLLLQYGSFCTQLNRALTGIAVEQDKNTQE
MW938104/P.1/Gamma           SVTTEILPVSMTKTSVDCTMYICGDSTECSNLLLQYGSFCTQLNRALTGIAVEQDKNTQE
MZ896179/P.1/Gamma           SVTTEILPVSMTKTSVDCTMYICGDSTECSNLLLQYGSFCTQLNRALTGIAVEQDKNTQE
MZ896359/P.1/Gamma           SVTTEILPVSMTKTSVDCTMYICGDSTECSNLLLQYGSFCTQLNRALTGIAVEQDKNTQE
MZ896669/P.1/Gamma           SVTTEILPVSMTKTSVDCTMYICGDSTECSNLLLQYGSFCTQLNCALTGIAVEQDKNTQE
OU565182/P.1/Gamma           SVTTEILPVSMTKTSVDCTMYICGDSTECSNLLLQYGSFCTQLNRALTGIAVEQDKNTQE
OU492255/P.1/Gamma           SVTTEILPVSMTKTSVDCTMYICGDSTECSNLLLQYGSFCTQLNRALTGIAVEQDKNTQE
MZ911221/B.1.427/Epsilon     SVTTEILPVSMTKTSVDCTMYICGDSTECSNLLLQYGSFCTQLNRALTGIAVEQDKNTQE
MZ375854/B.1.427/Epsilon     SVTTEILPVSMTKTSVDCTMYICGDSTECSNLLLQYGSFCTQLNRALTGIAVEQDKNTQE
MZ375858/B.1.427/Epsilon     SVTTEILPVSMTKTSVDCTMYICGDSTECSNLLLQYGSFCTQLNRALTGIAVEQDKNTQE
MZ943834/B.1.429/Epsilon     SVTTEILPVSMTKTSVDCTMYICGDSTECSNLLLQYGSFCTQLNRALTGIAVEQDKNTQE
MZ277390/B.1.429/Epsilon     SVTTEILPVSMTKTSVDCTMYICGDSTECSNLLLQYGSFCTQLNRALTGIAVEQDKNTQE
OU282717/B.1.429/Epsilon     SVTTEILPVSMTKTSVDCTMYICGDSTECSNLLLQYGSFCTQLNRALTGIAVEQDKNTQE
OU235436/B.1.429/Epsilon     SVTTEILPVSMTKTSVDCTMYICGDSTECSNLLLQYGSFCTQLNRALTGIAVEQDKNTQE
MZ687447/B.1.429/Epsilon     SVTTEILPVSMTKTSVDCTMYICGDSTECSNLLLQYGSFCTQLNRALTGIAVEQDKNTQE
MZ538882/B.1.617.1/Kappa     SVTTEILPVSMTKTSVDCTMYICGDSTECSNLLLQYGSFCTQLNRALTGIAVEQDKNTQE
MZ724425/B.1.617.1/Kappa     SVTTEILPVSMTKTSVDCTMYICGDSTECSNLLLQYGSFCTQLNRALTGIAVEQDKNTQE
MZ363839/B.1.617.1/Kappa     SVTTEILPVSMTKTSVDCTMYICGDSTECSNLLLQYGSFCTQLNRALTGIAVEQDKNTQE
MZ332524/B.1.617.1/Kappa     SVTTEILPVSMTKTSVDCTMYICGDSTECSNLLLQYGSFCTQLNRALTGIAVEQDKNTQE
MZ332527/B.1.617.1/Kappa     SVTTEILPVSMTKTSVDCTMYICGDSTECSNLLLQYGSFCTQLNRALTGIAVEQDKNTQE
LC633761/B.1.617.1/Kappa     SVTTEILPVSMTKTSVDCTMYICGDSTECSNLLLQYGSFCTQLNRALTGIAVEQDKNTQE
MZ562746/B.1.617.1/Kappa     SVTTEILPVSMTKTSVDCTMYICGDSTECSNLLLQYGSFCTQLNRALTGIAVEQDKNTQE
MZ571142/B.1.617.1/Kappa     SVTTEILPVSMTKTSVDCTMYICGDSTECSNLLLQYGSFCTQLNRALTGIAVEQDKNTQE
OU168413/B.1.617.1/Kappa     SVTTEILPVSMTKTSVDCTMYICGDSTECSNLLLQYGSFCTQLNRALTGIAVEQDKNTQE
MZ026886/B.1.617.1/Kappa     SVTTEILPVSMTKTSVDCTMYICGDSTECSNLLLQYGSFCTQLNRALTGIAVEQDKNTQE
OU172184/B.1.617.1/Kappa     SVTTEILPVSMTKTSVDCTMYICGDSTECSNLLLQYGSFCTQLNRALTGIAVEQDKNTQE
MZ397173/B.1.617.1/Kappa     SVTTEILPVSMTKTSVDCTMYICGDSTECSNLLLQYGSFCTQLNRALTGIAVEQDKNTQE
MZ397176/B.1.617.1/Kappa     SVTTEILPVSMTKTSVDCTMYICGDSTECSNLLLQYGSFCTQLNRALTGIAVEQDKNTQE
MZ397175/B.1.617.1/Kappa     SVTTEILPVSMTKTSVDCTMYICGDSTECSNLLLQYGSFCTQLNRALTGIAVEQDKNTQE
MZ397177/B.1.617.1/Kappa     SVTTEILPVSMTKTSVDCTMYICGDSTECSNLLLQYGSFCTQLNRALTGIAVEQDKNTQE
OU290035/B.1.617.1/Kappa     SVTTEILPVSMTKTSVDCTMYICGDSTECSNLLLQYGSFCTQLNRALTGIAVEQDKNTQE
OU265178/B.1.617.1/Kappa     SVTTEILPVSMTKTSVDCTMYICGDSTECSNLLLQYGSFCTQLNRALTGIAVEQDKNTQE
OU254949/B.1.617.1/Kappa     SVTTEILPVSMTKTSVDCTMYICGDSTECSNLLLQYGSFCTQLNRALTGIAVEQDKNTQE
OU247018/B.1.617.1/Kappa     SVTTEILPVSMTKTSVDCTMYICGDSTECSNLLLQYGSFCTQLNRALTGIAVEQDKNTQE
OU061269/B.1.617.1/Kappa     SVTTEILPVSMTKTSVDCTMYICGDSTECSNLLLQYGSFCTQLNRALTGIAVEQDKNTQE
OU182966/B.1.617.1/Kappa     SVTTEILPVSMTKTSVDCTMYICGDSTECSNLLLQYGSFCTQLNRALTGIAVEQDKNTQE
MZ746235/B.1.617.1/Kappa     SVTTEILPVSMTKTSVDCTMYICGDSTECSNLLLQYGSFCTQLNRALTGIAVEQDKNTQE
MZ487800/B.1.617.1/Kappa     SVTTEILPVSMTKTSVDCTMYICGDSTECSNLLLQYGSFCTQLNRALTGIAVEQDKNTQE
OU322235/B.1.617.1/Kappa     SVTTEILPVSMTKTSVDCTMYICGDSTECSNLLLQYGSFCTQLNRALTGIAVEQDKNTQE
MZ953185/C.37/Lambda         SVTTEILPVSMTKTSVDCTMYICGDSTECSNLLLQYGSFCTQLNRALTGIAVEQDKNTQE
MZ937666/C.37/Lambda         SVTTEILPVSMTKTSVDCTMYICGDSTECSNLLLQYGSFCTQLNRALTGIAVEQDKNTQE
OU356036/C.37/Lambda         SVTTEILPVSMTKTSVDCTMYICGDSTECSNLLLQYGSFCTQLNRALTGIAVEQDKNTQE
OU351967/C.37/Lambda         SVTTEILPVSMTKTSVDCTMYICGDSTECSNLLLQYGSFCTQLNRALTGIAVEQDKNTQE
OU053018/C.37/Lambda         SVTTEILPVSMTKTSVDCTMYICGDSTECSNLLLQYGSFCTQLNRALTGIAVEQDKNTQE
OU268528/C.37/Lambda         SVTTEILPVSMTKTSVDCTMYICGDSTECSNLLLQYGSFCTQLNRALTGIAVEQDKNTQE
OU100999/C.37/Lambda         SVTTEILPVSMTKTSVDCTMYICGDSTECSNLLLQYGSFCTQLNRALTGIAVEQDKNTQE
MZ275295/C.37/Lambda         SVTTEILPVSMTKTSVDCTMYICGDSTECSNLLLQYGSFCTQLNRALTGIAVEQDKNTQE
MZ275301/C.37/Lambda         SVTTEILPVSMTKTSVDCTMYICGDSTECSNLLLQYGSFCTQLNRALTGIAVEQDKNTQE
MZ908711/C.37/Lambda         SVTTEILPVSMTKTSVDCTMYICGDSTECSNLLLQYGSFCTQLNRALTGIAVEQDKNTQE
MZ496613/C.37/Lambda         SVTTEILPVSMTKTSVDCTMYICGDSTECSNLLLQYGSFCTQLNRALTGIAVEQDKNTQE
OU517038/C.37/Lambda         SVTTEILPVSMTKTSVDCTMYICGDSTECSNLLLQYGSFCTQLNRALTGIAVEQDKNTQE
MZ953277/C.37/Lambda         SVTTEILPVSMTKTSVDCTMYICGDSTECSNLLLQYGSFCTQLNRALTGIAVEQDKNTQE
MZ942542/C.37/Lambda         SVTTEILPVSMTKTSVDCTMYICGDSTECSNLLLQYGSFCTQLNRALTGIAVEQDKNTQE
MZ857990/C.37/Lambda         SVTTEILPVSMTKTSVDCTMYICGDSTECSNLLLQYGSFCTQLNRALTGIAVEQDKNTQE
```

```
                                790       800       810       820       830       840
MN908947/Wuhan-Hu-1       VFAQVKQIYKTPPIKDFGGFNFSQILPDPSKPSKRSFIEDLLFNKVTLADAGFIKQYGDC
BS001137/B.1.1.7/Alpha    VFAQVKQIYKTPPIKDFGGFNFSQILPDPSKPSKRSFIEDLLFNKVTLADAGFIKQYGDC
BS001138/B.1.1.7/Alpha    VFAQVKQIYKTPPIKDFGGFNFSQILPDPSKPSKRSFIEDLLFNKVTLADAGFIKQYGDC
BS001139/B.1.1.7/Alpha    VFAQVKQIYKTPPIKDFGGFNFSQILPDPSKPSKRSFIEDLLFNKVTLADAGFIKQYGDC
BS001140/B.1.1.7/Alpha    VFAQVKQIYKTPPIKDFGGFNFSQILPDPSKPSKRSFIEDLLFNKVTLADAGFIKQYGDC
MW913362/B.1.1.7/Alpha    VFAQVKQIYKTPPIKDFGGFNFSQILPDPSKPSKRSFIEDLLFNKVTLADAGFIKQYGDC
MZ266365/B.1.1.7/Alpha    VFAQVKQIYKTPPIKDFGGFNFSQILPDPSKPSKRSFIEDLLFNKVTLADAGFIKQYGDC
MZ350105/B.1.1.7/Alpha    VFAQVKQIYKTPPIKDFGGFNFSQILPDPSKPSKRSFIEDLLFNKVTLADAGFIKQYGDC
MZ350109/B.1.1.7/Alpha    VFAQVKQIYKTPPIKDFGGFNFSQILPDPSKPSKRSFIEDLLFNKVTLADAGFIKQYGDC
MZ888516/B.1.1.7/Alpha    VFAQVKQIYKTPPIKDFGGFNFSQILPDPSKPSKRSFIEDLLFNKVTLADAGFIKQYGDC
OU562607/B.1.1.7/Alpha    VFAQVKQIYKTPPIKDFGGFNFSQILPDPSKPSKRSFIEDLLFNKVTLADAGFIKQYGDC
OU514166/B.1.1.7/Alpha    VFAQVKQIYKTPPIKDFGGFNFSQILPDPSKPSKRSFIEDLLFNKVTLADAGFIKQYGDC
OU538851/B.1.1.7/Alpha    VFAQVKQIYKTPPIKDFGGFNFSQILPDPSKPSKRSFIEDLLFNKVTLADAGFIKQYGDC
OU538849/B.1.1.7/Alpha    VFAQVKQIYKTPPIKDFGGFNFSQILPDPSKPSKRSFIEDLLFNKVTLADAGFIKQYGDC
MZ895877/B.1.1.7/Alpha    VFAQVKQIYKTPPIKDFGGFNFSQILPDPSKPSKRSFIEDLLFNKVTLADAGFIKQYGDC
OU514176/B.1.1.7/Alpha    VFAQVKQIYKTPPIKDFGGFNFSQILPDPSKPSKRSFIEDLLFNKVTLADAGFIKQYGDC
OU562608/B.1.1.7/Alpha    VFAQVKQIYKTPPIKDFGGFNFSQILPDSSKPSKRSFIEDLLFNKVTLADAGFIKQYGDC
MW981442/B.1.351/Beta     VFAQVKQIYKTPPIKDFGGFNFSQILPDPSKPSKRSFIEDLLFNKVTLADAGFIKQYGDC
MZ298839/B.1.351/Beta     VFAQVKQIYKTPPIKDFGGFNFSQILPDPSKPSKRSFIEDLLFNKVTLADAGFIKQYGDC
MZ913100/B.1.351/Beta     VFAQVKQIYKTPPIKDFGGFNFSQILPDPSKPSKRSFIEDLLFNKVTLADAGFIKQYGDC
MZ879365/B.1.351/Beta     VFAQVKQIYKTPPIKDFGGFNFSQILPDPSKPSKRSFIEDLLFNKVTLADAGFIKQYGDC
OU525106/B.1.351/Beta     VFAQVKQIYKTPPIKDFGGFNFSQILPDPSKPSKRSFIEDLLFNKVTLADAGFIKQYGDC
OU539352/B.1.351/Beta     VFAQVKQIYKTPPIKDFGGFNFSQILPDPSKPSKRSFIEDLLFNKVTLADAGFIKQYGDC
MZ202314/B.1.351/Beta     VFAQVKQIYKTPPIKDFGGFNFSQILPDPSKPSKRSFIEDLLFNKVTLADAGFIKQYGDC
MZ298840/B.1.351/Beta     VFAQVKQIYKTPPIKDFGGFNFSQILPDPSKPSKRSFIEDLLFNKVTLADAGFIKQYGDC
MZ376663/B.1.351/Beta     VFAQVKQIYKTPPIKDFGGFNFSQILPDPSKPSKRSFIEDLLFNKVTLADAGFIKQYGDC
LC643036/B.1.617.2/Delta  VFAQVKQIYKTPPIKDFGGFNFSQILPDPSKPSKRSFIEDLLFNKVTLADAGFIKQYGDC
LC643044/B.1.617.2/Delta  VFAQVKQIYKTPPIKDFGGFNFSQILPDPSKPSKRSFIEDLLFNKVTLADAGFIKQYGDC
MZ853946/B.1.617.2/Delta  VFAQVKQIYKTPPIKDFGGFNFSQILPDPSKPSKRSFIEDLLFNKVTLADAGFIKQYGDC
MZ854386/B.1.617.2/Delta  VFAQVKQIYKTPPIKDFGGFNFSQILPDPSKPSKRSFIEDLLFNKVTLADAGFIKQYGDC
MZ888532/B.1.617.2/Delta  VFAQVKQIYKTPPIKDFGGFNFSQILPDPSKPSKRSFIEDLLFNKVTLADAGFIKQYGDC
MZ888533/B.1.617.2/Delta  VFAQVKQIYKTPPIKDFGGFNFSQILPDPSKPSKRSFIEDLLFNKVTLADAGFIKQYGDC
MZ896136/B.1.617.2/Delta  VFAQVKQIYKTPPIKDFGGFNFSQILPDPSKPSKRSFIEDLLFNKVTLADAGFIKQYGDC
OU539261/B.1.617.2/Delta  VFAQVKQIYKTPPIKDFGGFNFSQILPDPSKPSKRSFIEDLLFNKVTLADAGFIKQYGDC
MZ854389/B.1.617.2/Delta  VFAQVKQIYKTPPIKDFGGFNFSQILPDPSKPSKRSFIEDLLFNKVTLADAGFIKQYGDC
OU562629/B.1.617.2/Delta  VFAQVKQIYKTPPIKDFGGFNFSQILPDPSKPSKRSFIEDLLFNKVTLADAGFIKQYGDC
OU539690/B.1.617.2/Delta  VFAQVKQIYKTPPIKDFGGFNFSQILPDPSKPSKRSFIEDLLFNKVTLADAGFIKQYGDC
MZ854387/B.1.617.2/Delta  VFAQVKQIYKTPPIKDFGGFNFSQILPDPSKPSKRSFIEDLLFNKVTLADAGFIKQYGDC
OU539784/P.1/Gamma        VFAQVKQIYKTPPIKDFGGFNFSQILPDPSKPSKRSFIEDLLFNKVTLADAGFIKQYGDC
OU539830/P.1/Gamma        VFAQVKQIYKTPPIKDFGGFNFSQILPDPSKPSKRSFIEDLLFNKVTLADAGFIKQYGDC
OU539894/P.1/Gamma        VFAQVKQIYKTPPIKDFGGFNFSQILPDPSKPSKRSFIEDLLFNKVTLADAGFIKQYGDC
MZ477748/P.1/Gamma        VFAQVKQIYKTPPIKDFGGFNFSQILPDPSKPSKRSFIEDLLFNKVTLADAGFIKQYGDC
MZ477746/P.1/Gamma        VFAQVKQIYKTPPIKDFGGFNFSQILPDPSKPSKRSFIEDLLFNKVTLADAGFIKQYGDC
MW938104/P.1/Gamma        VFAQVKQIYKTPPIKDFGGFNFSQILPDPSKPSKRSFIEDLLFNKVTLADAGFIKQYGDC
MZ896179/P.1/Gamma        VFAQVKQIYKTPPIKDFGGFNFSQILPDPSKPSKRSFIEDLLFNKVTLADAGFIKQYGDC
MZ896359/P.1/Gamma        VFAQVKQIYKTPPIKDFGGFNFSQILPDPSKPSKRSFIEDLLFNKVTLADAGFIKQYGDC
MZ896669/P.1/Gamma        VFAQVKQIYKTPPIKDFGGFNFSQILPDPSKPSKRSFIEDLLFNKVTLADAGFIKQYGDC
OU565182/P.1/Gamma        VFAQVKQIYKTPPIKDFGGFNFSQILPDPSKPSKRSFIEDLLFNKVTLADAGFIKQYGDC
OU492255/P.1/Gamma        VFAQVKQIYKTPPIKDFGGFNFSQILPDPSKPSKRSFIEDLLFNKVTLADAGFIKQYGDC
MZ911221/B.1.427/Epsilon  VFAQVKQIYKTPPIKDFGGFNFSQILPDPSKPSKRSFIEDLLFNKVTLADAGFIKQYGDC
MZ375854/B.1.427/Epsilon  VFAQVKQIYKTPPIKDFGGFNFSQILPDPSKPSKRSFIEDLLFNKVTLADAGFIKQYGDC
MZ375858/B.1.427/Epsilon  VFAQVKQIYKTPPIKDFGGFNFSQILPDPSKPSKRSFIEDLLFNKVTLADAGFIKQYGDC
MZ943834/B.1.429/Epsilon  VFAQVKQIYKTPPIKDFGGFNFSQILPDPSKPSKRSFIEDLLFNKVTLADAGFIKQYGDC
MZ277390/B.1.429/Epsilon  VFAQVKQIYKTPPIKDFGGFNFSQILPDPSKPSKRSFIEDLLFNKVTLADAGFIKQYGDC
OU282717/B.1.429/Epsilon  VFAQVKQIYKTPPIKNFGGFNFSQILPDPSKPSKRSFIEDLLFNKVTLADAGFIKQYGDC
OU235436/B.1.429/Epsilon  VFAQVKQIYKTPPIKDFGGFNFSQILPDPSKPSKRSFIEDLLFNKVTLADAGFIKQYGDC
MZ687447/B.1.429/Epsilon  VFAQVKQIYKTPPIKDFGGFNFSQILPDPSKPSKRSFIEDLLFNKVTLADAGFIKQYGDC
MZ538882/B.1.617.1/Kappa  VFAQVKQIYKTPPIKDFGGFNFSQILPDPSKPSKRSFIEDLLFNKVTLADAGFIKQYGDC
MZ724425/B.1.617.1/Kappa  VFAQVKQIYKTPPIKDFGGFNFSQILPDPSKPSKRSFIEDLLFNKVTLADAGFIKQYGDC
MZ363839/B.1.617.1/Kappa  VFAQVKQIYKTPPIKDFGGFNFSQILPDPSKPSKRSFIEDLLFNKVTLADAGFIKQYGDC
MZ332524/B.1.617.1/Kappa  VFAQVKQIYKTPPIKDFGGFNFSQILPDPSKPSKRSFIEDLLFNKVTLADAGFIKQYGDC
MZ332527/B.1.617.1/Kappa  VFAQVKQIYKTPPIKDFGGFNFSQILPDPSKPSKRSFIEDLLFNKVTLADAGFIKQYGDC
LC633761/B.1.617.1/Kappa  VFAQVKQIYKTPPIKDFGGFNFSQILPDPSKPSKRSFIEDLLFNKVTLADAGFIKQYGDC
MZ562746/B.1.617.1/Kappa  VFAQVKQIYKTPPIKDFGGFNFSQILPDPSKPSKRSFIEDLLFNKVTLADAGFIKQYGDC
MZ571142/B.1.617.1/Kappa  VFAQVKQIYKTPPIKDFGGFNFSQILPDPSKPSKRSFIEDLLFNKVTLADAGFIKQYGDC
OU168413/B.1.617.1/Kappa  VFAQVKQIYKTPPIKDFGGFNFSQILPDPSKPSKRSFIEDLLFNKVTLADAGFIKQYGDC
MZ026886/B.1.617.1/Kappa  VFAQVKQIYKTPPIKDFGGFNFSQILPDPSKPSKRSFIEDLLFNKVTLADAGFIKQYGDC
OU172184/B.1.617.1/Kappa  VFAQVKQIYKTPPIKDFGGFNFSQILPDPSKPSKRSFIEDLLFNKVTLADAGFIKQYGDC
MZ397173/B.1.617.1/Kappa  VFAQVKQIYKTPPIKDFGGFNFSQILPDPSKPSKRSFIEDLLFNKVTLADAGFIKQYGDC
MZ397176/B.1.617.1/Kappa  VFAQVKQIYKTPPIKDFGGFNFSQILPDPSKPSKRSFIEDLLFNKVTLADAGFIKQYGDC
MZ397175/B.1.617.1/Kappa  VFAQVKQIYKTPPIKDFGGFNFSQILPDPSKPSKRSFIEDLLFNKVTLADAGFIKQYGDC
MZ397177/B.1.617.1/Kappa  VFAQVKQIYKTPPIKDFGGFNFSQILPDPSKPSKRSFIEDLLFNKVTLADAGFIKQYGDC
OU290035/B.1.617.1/Kappa  VFAQVKQIYKTPPIKDFGGFNFSQILPDPSKPSKRSFIEDLLFNKVTLADAGFIKQYGDC
OU265178/B.1.617.1/Kappa  VFAQVKQIYKTPPIKDFGGFNFSQILPDPSKPSKRSFIEDLLFNKVTLADAGFIKQYGDC
OU254949/B.1.617.1/Kappa  VFAQVKQIYKTPPIKDFGGFNFSQILPDPSKPSKRSFIEDLLFNKVTLADAGFIKQYGDC
OU247018/B.1.617.1/Kappa  VFAQVKQIYKTPPIKDFGGFNFSQILPDPSKPSKRSFIEDLLFNKVTLADAGFIKQYGDC
OU061269/B.1.617.1/Kappa  VFAQVKQIYKTPPIKDFGGFNFSQILPDPSKPSKRSFIEDLLFNKVTLADAGFIKQYGDC
OU182966/B.1.617.1/Kappa  VFAQVKQIYKTPPIKDFGGFNFSQILPDPSKPSKRSFIEDLLFNKVTLADAGFIKQYGDC
MZ746235/B.1.617.1/Kappa  VFAQVKQIYKTPPIKDFGGFNFSQILPDPSKPSKRSFIEDLLFNKVTLADAGFIKQYGDC
MZ487800/B.1.617.1/Kappa  VFAQVKQIYKTPPIKDFGGFNFSQILPDPSKPSKRSFIEDLLFNKVTLADAGFIKQYGDC
OU322235/B.1.617.1/Kappa  VFAQVKQIYKTPPIKDFGGFNFSQILPDPSKPSKRSFIEDLLFNKVTLADAGFIKQYGDC
MZ953185/C.37/Lambda      VFAQVKQIYKTPPIKDFGGFNFSQILPDPSKPSKRSFIEDLLFNKVTLADAGFIKQYGDC
MZ937666/C.37/Lambda      VFAQVKQIYKTPPIKDFGGFNFSQILPDPSKPSKRSFIEDLLFNKVTLADAGFIKQYGDC
OU356036/C.37/Lambda      VFAQVKQIYKTPPIKDFGGFNFSQILPDPSKPSKRSFIEDLLFNKVTLADAGFIKQYGDC
OU351967/C.37/Lambda      VFAQVKQIYKTPPIKDFGGFNFSQILPDPSKPSKRSFIEDLLFNKVTLADAGFIKQYGDC
OU053018/C.37/Lambda      VFAQVKQIYKTPPIKDFGGFNFSQILPDPSKPSKRSFIEDLLFNKVTLADAGFIKQYGDC
OU268528/C.37/Lambda      VFAQVKQIYKTPPIKDFGGFNFSQILPDPSKPSKRSFIEDLLFNKVTLADAGFIKQYGDC
OU100999/C.37/Lambda      VFAQVKQIYKTPPIKDFGGFNFSQILPDPSKPSKRSFIEDLLFNKVTLADAGFIKQYGDC
MZ275295/C.37/Lambda      VFAQVKQIYKTPPIKDFGGFNFSQILPDPSKPSKRSFIEDLLFNKVTLADAGFIKQYGDC
MZ275301/C.37/Lambda      VFAQVKQIYKTPPIKDFGGFNFSQILPDPSKPSKRSFIEDLLFNKVTLADAGFIKQYGDC
MZ908711/C.37/Lambda      VFAQVKQIYKTPPIKDFGGFNFSQILPDPSKPSKRSFIEDLLFNKVTLADAGFIKQYGDC
MZ496613/C.37/Lambda      VFAQVKQIYKTPPIKDFGGFNFSQILPDPSKPSKRSFIEDLLFNKVTLADAGFIKQYGDC
OU517038/C.37/Lambda      VFAQVKQIYKTPPIKDFGGFNFSQILPDPSKPSKRSFIEDLLFNKVTLADAGFIKQYGDC
MZ953277/C.37/Lambda      VFAQVKQIYKTPPIKDFGGFNFSQILPDPSKPSKRSFIEDLLFNKVTLADAGFIKQYGDC
MZ942542/C.37/Lambda      VFAQVKQIYKTPPIKDFGGFNFSQILPDPSKPSKRSFIEDLLFNKVTLADAGFIKQYGDC
MZ857990/C.37/Lambda      VFAQVKQIYKTPPIKDFGGFNFSQILPDPSKPSKRSFIEDLLFNKVTLADAGFIKQYGDC
```

```
                                       850       860       870       880       890       900
                                         .         .         .         .         .         .
MN908947/Wuhan-Hu-1            LGDIAARDLICAQKFNGLTVLPPLLTDEMIAQYTSALLAGTITSGWTFGAGAALQIPFAM
BS001137/B.1.1.7/Alpha         LGDIAARDLICAQKFNGLTVLPPLLTDEMIAQYTSALLAGTITSGWTFGAGAALQIPFAM
BS001138/B.1.1.7/Alpha         LGDIAARDLICAQKFNGLTVLPPLLTDEMIAQYTSALLAGTITSGWTFGAGAALQIPFAM
BS001139/B.1.1.7/Alpha         LGDIAARDLICAQKFNGLTVLPPLLTDEMIAQYTSALLAGTITSGWTFGAGAALQIPFAM
BS001140/B.1.1.7/Alpha         LGDIAARDLICAQKFNGLTVLPPLLTDEMIAQYTSALLAGTITSGWTFGAGAALQIPFAM
MW913362/B.1.1.7/Alpha         LGDIAARDLICAQKFNGLTVLPPLLTDEMIAQYTSALLAGTITSGWTFGAGAALQIPFAM
MZ266365/B.1.1.7/Alpha         LGDIAARDLICAQKFNGLTVLPPLLTDEMIAQYTSALLAGTITSGWTFGAGAALQIPFAM
MZ350105/B.1.1.7/Alpha         LGDIAARDLICAQKFNGLTVLPPLLTDEMIAQYTSALLAGTITSGWTFGAGAALQIPFAM
MZ350109/B.1.1.7/Alpha         LGDIAARDLICAQKFNGLTVLPPLLTDEMIAQYTSALLAGTITSGWTFGAGAALQIPFAM
MZ888516/B.1.1.7/Alpha         LGDIAARDLICAQKFNGLTVLPPLLTDEMIAQYTSALLAGTITSGWTFGAGAALQIPFAM
OU562607/B.1.1.7/Alpha         LGDIAARDLICAQKFNGLTVLPPLLTDEMIAQYTSALLAGTITSGWTFGAGAALQIPFAM
OU514166/B.1.1.7/Alpha         LGDIAARDLICAQKFNGLTVLPPLLTDEMIAQYTSALLAGTITSGWTFGAGAALQIPFAM
OU538851/B.1.1.7/Alpha         LGDIAARDLICAQKFNGLTVLPPLLTDEMIAQYTSALLAGTITSGWTFGAGAALQIPFAM
OU538849/B.1.1.7/Alpha         LGDIAARDLICAQKFNGLTVLPPLLTDEMIAQYTSALLAGTITSGWTFGAGAALQIPFAM
MZ895877/B.1.1.7/Alpha         LGDIAARDLICAQKFNGLTVLPPLLTDEMIAQYTSALLAGTITSGWTFGAGAALQIPFAM
OU514176/B.1.1.7/Alpha         LGDIAARDLICAQKFNGLTVLPPLLTDEMIAQYTSALLAGTITSGWTFGAGAALQIPFAM
OU562608/B.1.1.7/Alpha         LGDIAARDLICAQKFNGLTVLPPLLTDEMIAQYTSALLAGTITSGWTFGAGAALQIPFAM
MW981442/B.1.351/Beta          LGDIAARDLICAQKFNGLTVLPPLLTDEMIAQYTSALLAGTITSGWTFGAGAALQIPFAM
MZ298839/B.1.351/Beta          LGDIAARDLICAQKFNGLTVLPPLLTDEMIAQYTSALLAGTITSGWTFGAGAALQIPFAM
MZ913100/B.1.351/Beta          LGDIAARDLICAQKFNGLTVLPPLLTDEMIAQYTSALLAGTITSGWTFGAGAALQIPFAM
MZ879365/B.1.351/Beta          LGDIAARDLICAQKFNGLTVLPPLLTDEMIAQYTSALLAGTITSGWTFGAGAALQIPFAM
OU525106/B.1.351/Beta          LGDIAARDLICAQKFNGLTVLPPLLTDEMIAQYTSALLAGTITSGWTFGAGAALQIPFAM
OU539352/B.1.351/Beta          LGDIAARDLICAQKFNGLTVLPPLLTDEMIAQYTSALLAGTITSGWTFGAGAALQIPFAM
MZ202314/B.1.351/Beta          LGDIAARDLICAQKFNGLTVLPPLLTDEMIAQYTSALLAGTITSGWTFGAGAALQIPFAM
MZ298840/B.1.351/Beta          LGDIAARDLICAQKFNGLTVLPPLLTDEMIAQYTSALLAGTITSGWTFGAGAALQIPFAM
MZ376663/B.1.351/Beta          LGDIAARDLICAQKFNGLTVLPPLLTDEMIAQYTSALLAGTITSGWTFGAGAALQIPFAM
LC643036/B.1.617.2/Delta       LGDIAARDLICAQKFNGLTVLPPLLTDEMIAQYTSALLAGTITSGWTFGAGAALQIPFAM
LC643044/B.1.617.2/Delta       LGDIAARDLICAQKFNGLTVLPPLLTDEMIAQYTSALLAGTITSGWTFGAGAALQIPFAM
MZ853946/B.1.617.2/Delta       LGDIAARDLICAQKFNGLTVLPPLLTDEMIAQYTSALLAGTITSGWTFGAGAALQIPFAM
MZ854386/B.1.617.2/Delta       LGDIAARDLICAQKFNGLTVLPPLLTDEMIAQYTSALLAGTITSGWTFGAGAALQIPFAM
MZ888532/B.1.617.2/Delta       LGDIAARDLICAQKFNGLTVLPPLLTDEMIAQYTSALLAGTITSGWTFGAGAALQIPFAM
MZ888533/B.1.617.2/Delta       LGDIAARDLICAQKFNGLTVLPPLLTDEMIAQYTSALLAGTITSGWTFGAGAALQIPFAM
MZ896136/B.1.617.2/Delta       LGDIAARDLICAQKFNGLTVLPPLLTDEMIAQYTSALLAGTITSGWTFGAGAALQIPFAM
OU539261/B.1.617.2/Delta       LGDIAARDLICAQKFNGLTVLPPLLTDEMIAQYTSALLAGTITSGWTFGAGAALQIPFAM
MZ854389/B.1.617.2/Delta       LGDIAARDLICAQKFNGLTVLPPLLTDEMIAQYTSALLAGTITSGWTFGAGAALQIPFAM
OU562629/B.1.617.2/Delta       LGDIAARDLICAQKFNGLTVLPPLLTDEMIAQYTSALLAGTITSGWTFGAGAALQIPFAM
OU539690/B.1.617.2/Delta       LGDIAARDLICAQKFNGLTVLPPLLTDEMIAQYTSALLAGTITSGWTFGAGAALQIPFAM
MZ854387/B.1.617.2/Delta       LGDIAARDLICAQKFNGLTVLPPLLTDEMIAQYTSALLAGTITSGWTFGAGAALQIPFAM
OU539784/P.1/Gamma             LGDIAARDLICAQKFNGLTVLPPLLTDEMIAQYTSALLAGTITSGWTFGAGAALQIPFAM
OU539830/P.1/Gamma             LGDIAARDLICAQKFNGLTVLPPLLTDEMIAQYTSALLAGTITSGWTFGAGAALQIPFAM
OU539894/P.1/Gamma             LGDIAARDLICAQKFNGLTVLPPLLTDEMIAQYTSALLAGTITSGWTFGAGAALQIPFAM
MZ477748/P.1/Gamma             LGDIAARDLICAQKFNGLTVLPPLLTDEMIAQYTSALLAGTITSGWTFGAGAALQIPFAM
MZ477746/P.1/Gamma             LGDIAARDLICAQKFNGLTVLPPLLTDEMIAQYTSALLAGTITSGWTFGAGAALQIPFAM
MW938104/P.1/Gamma             LGDIAARDLICAQKFNGLTVLPPLLTDEMIAQYTSALLAGTITSGWTFGAGAALQIPFAM
MZ896179/P.1/Gamma             LGDIAARDLICAQKFNGLTVLPPLLTDEMIAQYTSALLAGTITSGWTFGAGAALQIPFAM
MZ896359/P.1/Gamma             LGDIAARDLICAQKFNGLTVLPPLLTDEMIAQYTSALLAGTITSGWTFGAGAALQIPFAM
MZ896669/P.1/Gamma             LGDIAARDLICAQKFNGLTVLPPLLTDEMIAQYTSALLAGTITSGWTFGAGAALQIPFAM
OU565182/P.1/Gamma             LGDIAARDLICAQKFNGLTVLPPLLTDEMIAQYTSALLAGTITSGWTFGAGAALQIPFAM
OU492255/P.1/Gamma             LGDIAARDLICAQKFNGLTVLPPLLTDEMIAQYTSALLAGTITSGWTFGAGAALQIPFAM
MZ911221/B.1.427/Epsilon       LGDIAARDLICAQKFNGLTVLPPLLTDEMIAQYTSALLAGTITSGWTFGAGAALQIPFAM
MZ375854/B.1.427/Epsilon       LGDIAARDLICAQKFNGLTVLPPLLTDEMIAQYTSALLAGTITSGWTFGAGAALQIPFAM
MZ375858/B.1.427/Epsilon       LGDIAARDLICAQKFNGLTVLPPLLTDEMIAQYTSALLAGTITSGWTFGAGAALQIPFAM
MZ943834/B.1.429/Epsilon       LGDIAARDLICAQKFNGLTVLPPLLTDEMIAQYTSALLAGTITSGWTFGAGAALQIPFAM
MZ277390/B.1.429/Epsilon       LGDIAARDLICAQKFNGLTVLPPLLTDEMIAQYTSALLAGTITSGWTFGAGAALQIPFAM
OU282717/B.1.429/Epsilon       LGDIAARDLICAQKFNGLTVLPPLLTDEMIAQYTSALLAGTITSGWTFGAGAALQIPFAM
OU235436/B.1.429/Epsilon       LGDIAARDLICAQKFNGLTVLPPLLTDEMIAQYTSALLAGTITSGWTFGAGAALQIPFAM
MZ687447/B.1.429/Epsilon       LGDIAARDLICAQKFNGLTVLPPLLTDEMIAQYTSALLAGTITSGWTFGAGAALQIPFAM
MZ538882/B.1.617.1/Kappa       LGDIAARDLICAQKFNGLTVLPPLLTDEMIAQYTSALLAGTITSGWTFGAGAALQIPFAM
MZ724425/B.1.617.1/Kappa       LGDIAARDLICAQKFNGLTVLPPLLTDEMIAQYTSALLAGTITSGWTFGAGAALQIPFAM
MZ363839/B.1.617.1/Kappa       LGDIAARDLICAQKFNGLTVLPPLLTDEMIAQYTSALLAGTITSGWTFGAGAALQIPFAM
MZ332524/B.1.617.1/Kappa       LGDIAARDLICAQKFNGLTVLPPLLTDEMIAQYTSALLAGTITSGWTFGAGAALQIPFAM
MZ332527/B.1.617.1/Kappa       LGDIAARDLICAQKFNGLTVLPPLLTDEMIAQYTSALLAGTITSGWTFGAGAALQIPFAM
LC633761/B.1.617.1/Kappa       LGDIAARDLICAQKFNGLTVLPPLLTDEMIAQYTSALLAGTITSGWTFGAGAALQIPFAM
MZ562746/B.1.617.1/Kappa       LGDIAARDLICAQKFNGLTVLPPLLTDEMIAQYTSALLAGTITSGWTFGAGAALQIPFAM
MZ571142/B.1.617.1/Kappa       LGDIAARDLICAQKFNGLTVLPPLLTDEMIAQYTSALLAGTITSGWTFGAGAALQIPFAM
OU168413/B.1.617.1/Kappa       LGDIAARDLICAQKFNGLTVLPPLLTDEMIAQYTSALLAGTITSGWTFGAGAALQIPFAM
MZ026886/B.1.617.1/Kappa       LGDIAARDLICAQKFNGLTVLPPLLTDEMIAQYTSALLAGTITSGWTFGAGAALQIPFAM
OU172184/B.1.617.1/Kappa       LGDIAARDLICAQKFNGLTVLPPLLTDEMIAQYTSALLAGTITSGWTFGAGAALQIPFAM
MZ397173/B.1.617.1/Kappa       LGDIAARDLICAQKFNGLTVLPPLLTDEMIAQYTSALLAGTITSGWTFGAGAALQIPFAM
MZ397176/B.1.617.1/Kappa       LGDIAARDLICAQKFNGLTVLPPLLTDEMIAQYTSALLAGTITSGWTFGAGAALQIPFAM
MZ397175/B.1.617.1/Kappa       LGDIAARDLICAQKFNGLTVLPPLLTDEMIAQYTSALLAGTITSGWTFGAGAALQIPFAM
MZ397177/B.1.617.1/Kappa       LGDIAARDLICAQKFNGLTVLPPLLTDEMIAQYTSALLAGTITSGWTFGAGAALQIPFAM
OU290035/B.1.617.1/Kappa       LGDIAARDLICAQKFNGLTVLPPLLTDEMIAQYTSALLAGTITSGWTFGAGAALQIPFAM
OU265178/B.1.617.1/Kappa       LGDIAARDLICAQKFNGLTVLPPLLTDEMIAQYTSALLAGTITSGWTFGAGAALQIPFAM
OU254949/B.1.617.1/Kappa       LGDIAARDLICAQKFNGLTVLPPLLTDEMIAQYTSALLAGTITSGWTFGAGAALQIPFAM
OU247018/B.1.617.1/Kappa       LGDIAARDLICAQKFNGLTVLPPLLTDEMIAQYTSALLAGTITSGWTFGAGAALQIPFAM
OU061269/B.1.617.1/Kappa       LGDIAARDLICAQKFNGLTVLPPLLTDEMIAQYTSALLAGTITSGWTFGAGAALQIPFAM
OU182966/B.1.617.1/Kappa       LGDIAARDLICAQKFNGLTVLPPLLTDEMIAQYTSALLAGTITSGWTFGAGAALQIPFAM
MZ746235/B.1.617.1/Kappa       LGDIAARDLICAQKFNGLTVLPPLLTDEMIAQYTSALLAGTITSGWTFGAGAALQIPFAM
MZ487800/B.1.617.1/Kappa       LGDIAARDLICAQKFNGLTVLPPLLTDEMIAQYTSALLAGTITSGWTFGAGAALQIPFAM
OU322235/B.1.617.1/Kappa       LGDIAARDLICAQKFNGLTVLPPLLTDEMIAQYTSALLAGTITSGWTFGAGAALQIPFAM
MZ953185/C.37/Lambda           LGDIAARDLICAQKFNGLNVLPPLLTDEMIAQYTSALLAGTITSGWTFGAGAALQIPFAM
MZ937666/C.37/Lambda           LGDIAARDLICAQKFNGLNVLPPLLTDEMIAQYTSALLAGTITSGWTFGAGAALQIPFAM
OU356036/C.37/Lambda           LGDIAARDLICAQKFNGLNVLPPLLTDEMIAQYTSALLAGTITSGWTFGAGAALQIPFAM
OU351967/C.37/Lambda           LGDIAARDLICAQKFNGLNVLPPLLTDEMIAQYTSALLSGTITSGWTFGAGAALQIPFAM
OU053018/C.37/Lambda           LGDIAARDLICAQKFNGLNVLPPLLTDEMIAQYTSALLAGTITSGWTFGAGAALQIPFAM
OU268528/C.37/Lambda           LGDIAARDLICAQKFNGLNVLPPLLTDEMIAQYTSALLAGTITSGWTFGAGAALQIPFAM
OU100999/C.37/Lambda           LGDIAARDLICAQKFNGLNVLPPLLTDEMIAQYTSALLAGTITSGWTFGAGAALQIPFAM
MZ275295/C.37/Lambda           LADIAARDLICAQKFNGLNVLPPLLTDEMIAQYTSALLAGTITSGWTFGAGAALQIPFAM
MZ275301/C.37/Lambda           LGDIAARDLICAQKFNGLNVLPPLLTDEMIAQYTSALLAGTITSGWTFGAGAALQIPFAM
MZ908711/C.37/Lambda           LGDIAARDLICAQKFNGLNVLPPLLTDEMIAQYTSALLAGTITSGWTFGAGAALQIPFAM
MZ496613/C.37/Lambda           LGDIAARDLICAQKFNGLNVLPPLLTDEMIAQYTSALLAGTITSGWTFGAGAALQIPFAM
OU517038/C.37/Lambda           LGDIAARDLICAQKFNGLNVLPPLLTDEMIAQYTSALLAGTITSGWTFGAGAALQIPFAM
MZ953277/C.37/Lambda           LGDIAARDLICAQKFNGLNVLPPLLTDEMIAQYTSALLAGTITSGWTFGAGAALQIPFAM
MZ942542/C.37/Lambda           LGDIAARDLICAQKFNGLNVLPPLLTDEMIAQYTSALLAGTITSGWTFGAGAALQIPFAM
MZ857990/C.37/Lambda           LGDIAARDLICAQKFNGLNVLPPLLTDEMIAQYTSALLAGTITSGWTFGAGAALQIPFAM
```

```
                                     910       920       930       940       950       960
                                       .         .         .         .         .         .
MN908947/Wuhan-Hu-1           QMAYRFNGIGVTQNVLYENQKLIANQFNSAIGKIQDSLSSTASALGKLQDVVNQNAQALN
BS001137/B.1.1.7/Alpha        QMAYRFNGIGVTQNVLYENQKLIANQFNSAIGKIQDSLSSTASALGKLQDVVNQNAQALN
BS001138/B.1.1.7/Alpha        QMAYRFNGIGVTQNVLYENQKLIANQFNSAIGKIQDSLSSTASALGKLQDVVNQNAQALN
BS001139/B.1.1.7/Alpha        QMAYRFNGIGVTQNVLYENQKLIANQFNSAIGKIQDSLSSTASALGKLQDVVNQNAQALN
BS001140/B.1.1.7/Alpha        QMAYRFNGIGVTQNVLYENQKLIANQFNSAIGKIQDSLSSTASALGKLQDVVNQNAQALN
MW913362/B.1.1.7/Alpha        QMAYRFNGIGVTQNVLYENQKLIANQFNSAIGKIQDSLSSTASALGKLQDVVNQNAQALN
MZ266365/B.1.1.7/Alpha        QMAYRFNGIGVTQNVLYENQKLIANQFNSAIGKIQDSLSSTASALGKLQDVVNQNAQALN
MZ350105/B.1.1.7/Alpha        QMAYRFNGIGVTQNVLYENQKLIANQFNSAIGKIQDSLSSTASALGKLQDVVNQNAQALN
MZ350109/B.1.1.7/Alpha        QMAYRFNGIGVTQNVLYENQKLIANQFNSAIGKIQDSLSSTASALGKLQDVVNQNAQALN
MZ888516/B.1.1.7/Alpha        QMAYRFNGIGVTQNVLYENQKLIANQFNSAIGKIQDSLSSTASALGKLQDVVNQNAQALN
OU562607/B.1.1.7/Alpha        QMAYRFNGIGVTQNVLYENQKLIANQFNSAIGKIQDSLSSTASALGKLQDVVNQNAQALN
OU514166/B.1.1.7/Alpha        QMAYRFNGIGVTQNVLYENQKLIANQFNSAIGKIQDSLSSTASALGKLQDVVNQNAQALN
OU538851/B.1.1.7/Alpha        QMAYRFNGIGVTQNVLYENQKLIANQFNSAIGKIQDSLSSTASALGKLQDVVNQNAQALN
OU538849/B.1.1.7/Alpha        QMAYRFNGIGVTQNVLYENQKLIANQFNSAIGKIQDSLSSTASALGKLQDVVNQNAQALN
MZ895877/B.1.1.7/Alpha        QMAYRFNGIGVTQNVLYENQKLIANQFNSAIGKIQDSLSSTASALGKLQDVVNQNAQALN
OU514176/B.1.1.7/Alpha        QMAYRFNGIGVTQNVLYENQKLIANQFNSAIGKIQDSLSSTASALGKLQDVVNQNAQALN
OU562608/B.1.1.7/Alpha        QMAYRFNGIGVTQNVLYENQKLIANQFNSAIGKIQDSLSSTASALGKLQDVVNQNAQALN
MW981442/B.1.351/Beta         QMAYRFNGIGVTQNVLYENQKLIANQFNSAIGKIQDSLSSTASALGKLQDVVNQNAQALN
MZ298839/B.1.351/Beta         QMAYRFNGIGVTQNVLYENQKLIANQFNSAIGKIQDSLSSTASALGKLQDVVNQNAQALN
MZ913100/B.1.351/Beta         QMAYRFNGIGVTQNVLYENQKLIANQFNSAIGKIQDSLSSTASALGKLQDVVNQNAQALN
MZ879365/B.1.351/Beta         QMAYRFNGIGVTQNVLYENQKLIANQFNSAIGKIQDSLSSTASALGKLQDVVNQNAQALN
OU525106/B.1.351/Beta         QMAYRFNGIGVTQNVLYENQKLIANQFNSAIGKIQDSLSSTASALGKLQDVVNQNAQALN
OU539352/B.1.351/Beta         QMAYRFNGIGVTQNVLYENQKLIANQFNSAIGKIQDSLSSTASALGKLQDVVNQNAQALN
MZ202314/B.1.351/Beta         QMAYRFNGIGVTQNVLYENQKLIANQFNSAIGKIQDSLSSTASALGKLQDVVNQNAQALN
MZ298840/B.1.351/Beta         QMAYRFNGIGVTQNVLYENQKLIANQFNSAIGKIQDSLSSTASALGKLQDVVNQNAQALN
MZ376663/B.1.351/Beta         QMAYRFNGIGVTQNVLYENQKLIANQFNSAIGKIQDSLSSTASALGKLQDVVNQNAQALN
LC643036/B.1.617.2/Delta      QMAYRFNGIGVTQNVLYENQKLIANQFNSAIGKIQDSLSSTASALGKLQNVVNQNAQALN
LC643044/B.1.617.2/Delta      QMAYRFNGIGVTQNVLYENQKLIANQFNSAIGKIQDSLSSTASALGKLQNVVNQNAQALN
MZ853946/B.1.617.2/Delta      QMAYRFNGIGVTQNVLYENQKLIANQFNSAIGKIQDSLSSTASALGKLQNVVNQNAQALN
MZ854386/B.1.617.2/Delta      QMAYRFNGIGVTQNVLYENQKLIANQFNSAIGKIQDSLSSTASALGKLQNVVNQNAQALN
MZ888532/B.1.617.2/Delta      QMAYRFNGIGVTQNVLYENQKLIANQFNSAIGKIQDSLSSTASALGKLQNVVNQNAQALN
MZ888533/B.1.617.2/Delta      QMAYRFNGIGVTQNVLYENQKLIANQFNSAIGKIQDSLSSTASALGKLQNVVNQNAQALN
MZ896136/B.1.617.2/Delta      QMAYRFNGIGVTQNVLYENQKLIANQFNSAIGKIQDSLSSTASALGKLQNVVNQNAQALN
OU539261/B.1.617.2/Delta      QMAYRFNGIGVTQNVLYENQKLIANQFNSAIGKIQDSLSSTASALGKLQNVVNQNAQALN
MZ854389/B.1.617.2/Delta      QMAYRFNGIGVTQNVLYENQKLIANQFNSAIGKIQDSLSSTASALGKLQNVVNQNAQALN
OU562629/B.1.617.2/Delta      QMAYRFNGIGVTQNVLYENQKLIANQFNSAIGKIQDSLSSTASALGKLQNVVNQNAQALN
OU539690/B.1.617.2/Delta      QMAYRFNGIGVTQNVLYENQKLIANQFNSAIGKIQDSLSSTASALGKLQNVVNQNAQALN
MZ854387/B.1.617.2/Delta      QMAYRFNGIGVTQNVLYENQKLIANQFNSAIGKIQDSLSSTASALGKLQNVVNQNAQALN
OU539784/P.1/Gamma            QMAYRFNGIGVTQNVLYENQKLIANQFNSAIGKIQDSLSSTASALGKLQDVVNQNAQALN
OU539830/P.1/Gamma            QMAYRFNGIGVTQNVLYENQKLIANQFNSAIGKIQDSLSSTASALGKLQDVVNQNAQALN
OU539894/P.1/Gamma            QMAYRFNGIGVTQNVLYENQKLIANQFNSAIGKIQDSLSSTASALGKLQDVVNQNAQALN
MZ477748/P.1/Gamma            QMAYRFNGIGVTQNVLYENQKLIANQFNSAIGKIQDSLSSTASALGKLQDVVNQNAQALN
MZ477746/P.1/Gamma            QMAYRFNGIGVTQNVLYENQKLIANQFNSAIGKIQDSLSSTASALGKLQDVVNQNAQALN
MW938104/P.1/Gamma            QMAYRFNGIGVTQNVLYENQKLIANQFNSAIGKIQDSLSSTASALGKLQDVVNQNAQALN
MZ896179/P.1/Gamma            QMAYRFNGIGVTQNVLYENQKLIANQFNSAIGKIQDSLSSTASALRKLQDVVNQNAQALN
MZ896359/P.1/Gamma            QMAYRFNGIGVTQNVLYENQKLIANQFNSAIGKIQDSLSSTASALGKLQDVVNQNAQALN
MZ896669/P.1/Gamma            QMAYRFNGIGVTQNVLYENQKLIANQFNSAIGKIQDSLSSTASALGKLQDVVNQNAQALN
OU565182/P.1/Gamma            QMAYRFNGIGVTQNVLYENQKLIANQFNSAIGKIQDSLSSTASALGKLQDVVNQNAQALN
OU492255/P.1/Gamma            QMAYRFNGIGVTQNVLYENQKLIANQFNSAIGKIQDSLSSTASALGKLQDVVNQNAQALN
MZ911221/B.1.427/Epsilon      QMAYRFNGIGVTQNVLYENQKLIANQFNSAIGKIQDSLSSTASALGKLQDVVNQNAQALN
MZ375854/B.1.427/Epsilon      QMAYRFNGIGVTQNVLYENQKLIANQFNSAIGKIQDSLSSTASALGKLQDVVNQNAQALN
MZ375858/B.1.427/Epsilon      QMAYRFNGIGVTQNVLYENQKLIANQFNSAIGKIQDSLSSTASALGKLQDVVNQNAQALN
MZ943834/B.1.429/Epsilon      QMAYRFNGIGVTQNVLYENQKLIANQFNSAIGKIQDSLSSTASALGKLQDVVNQNAQALN
MZ277390/B.1.429/Epsilon      QMAYRFNGIGVTQNVLYENQKLIANQFNSAIGKIQDSLSSTASALGKLQDVVNQNAQALN
OU282717/B.1.429/Epsilon      QMAYRFNGIGVTQNVLYENQKLIANQFNSAIGKIQDSLSSTASALGKLQDVVNQNAQALN
OU235436/B.1.429/Epsilon      QMAYRFNGIGVTQNVLYENQKLIANQFNSAIGKIQDSLSSTASALGKLQDVVNQNAQALN
MZ687447/B.1.429/Epsilon      QMAYRFNGIGVTQNVLYENQKLIANQFNSAIGKIQDSLSSTASALGKLQDVVNQNAQALN
MZ538882/B.1.617.1/Kappa      QMAYRFNGIGVTQNVLYENQKLIANQFNSAIGKIQDSLSSTASALGKLQDVVNQNAQALN
MZ724425/B.1.617.1/Kappa      QMAYRFNGIGVTQNVLYENQKLIANQFNSAIGKIQDSLSSTASALGKLQDVVNQNAQALN
MZ363839/B.1.617.1/Kappa      QMAYRFNGIGVTQNVLYENQKLIANQFNSAIGKIQDSLSSTASALGKLQDVVNQNAQALN
MZ332524/B.1.617.1/Kappa      QMAYRFNGIGVTQNVLYENQKLIANQFNSAIGKIQDSLSSTASALGKLQDVVNQNAQALN
MZ332527/B.1.617.1/Kappa      QMAYRFNGIGVTQNVLYENQKLIANQFNSAIGKIQDSLSSTASALGKLQDVVNQNAQALN
LC633761/B.1.617.1/Kappa      QMAYRFNGIGVTQNVLYENQKLIANQFNSAIGKIQDSLSSTASALGKLQDVVNQNAQALN
MZ562746/B.1.617.1/Kappa      QMAYRFNGIGVTQNVLYENQKLIANQFNSAIGKIQDSLSSTASALGKLQDVVNQNAQALN
MZ571142/B.1.617.1/Kappa      QMAYRFNGIGVTQNVLYENQKLIANQFNSAIGKIQDSLSSTASALGKLQDVVNQNAQALN
OU168413/B.1.617.1/Kappa      QMAYRFNGIGVTQNVLYENQKLIANQFNSAIGKIQDSLSSTASALGKLQDVVNQNAQALN
MZ026886/B.1.617.1/Kappa      QMAYRFNGIGVTQNVLYENQKLIANQFNSAIGKIQDSLSSTASALGKLQDVVNQNAQALN
OU172184/B.1.617.1/Kappa      QMAYRFNGIGVTQNVLYENQKLIANQFNSAIGKIQDSLSSTASALGKLQDVVNQNAQALN
MZ397173/B.1.617.1/Kappa      QMAYRFNGIGVTQNVLYENQKLIANQFNSAIGKIQDSLSSTASALGKLQDVVNQNAQALN
MZ397176/B.1.617.1/Kappa      QMAYRFNGIGVTQNVLYENQKLIANQFNSAIGKIQDSLSSTASALGKLQDVVNQNAQALN
MZ397175/B.1.617.1/Kappa      QMAYRFNGIGVTQNVLYENQKLIANQFNSAIGKIQDSLSSTASALGKLQDVVNQNAQALN
MZ397177/B.1.617.1/Kappa      QMAYRFNGIGVTQNVLYENQKLIANQFNSAIGKIQDSLSSTASALGKLQDVVNQNAQALN
OU290035/B.1.617.1/Kappa      QMAYRFNGIGVTQNVLYENQKLIANQFNSAIGKIQDSLSSTASALGKLQDVVNQNAQALN
OU265178/B.1.617.1/Kappa      QMAYRFNGIGVTQNVLYENQKLIANQFNSAIGKIQDSLSSTASALGKLQDVVNQNAQALN
OU254949/B.1.617.1/Kappa      QMAYRFNGIGVTQNVLYENQKLIANQFNSAIGKIQDSLSSTASALGKLQDVVNQNAQALN
OU247018/B.1.617.1/Kappa      QMAYRFNGIGVTQNVLYENQKLIANQFNSAIGKIQDSLSSTASALGKLQDVVNQNAQALN
OU061269/B.1.617.1/Kappa      QMAYRFNGIGVTQNVLYENQKLIANQFNSAIGKIQDSLSSTASALGKLQDVVNQNAQALN
OU182966/B.1.617.1/Kappa      QMAYRFNGIGVTQNVLYENQKLIANQFNSAIGKIQDSLSSTASALGKLQDVVNQNAQALN
MZ746235/B.1.617.1/Kappa      QMAYRFNGIGVTQNVLYENQKLIANQFNSAIGKIQDSLSSTASALGKLQDVVNQNAQALN
MZ487800/B.1.617.1/Kappa      QMAYRFNGIGVTQNVLYENQKLIANQFNSAIGKIQDSLSSTASALGKLQDVVNQNAQALN
OU322235/B.1.617.1/Kappa      QMAYRFNGIGVTQNVLYENQKLIANQFNSAIGKIQDSLSSTASALGKLQDVVNQNAQALN
MZ953185/C.37/Lambda          QMAYRFNGIGVTQNVLYENQKLIANQFNSAIGKIQDSLSSTASALGKLQDVVNQNAQALN
MZ937666/C.37/Lambda          QMAYRFNGIGVTQNVLYENQKLIANQFNSAIGKIQDSLSSTASALGKLQDVVNQNAQALN
OU356036/C.37/Lambda          QMAYRFNGIGVTQNVLYENQKLIANQFNSAIGKIQDSLSSTASALGKLQDVVNQNAQALN
OU351967/C.37/Lambda          QMAYRFNGIGVTQNVLYENQKLIANQFNSAIGKIQDSLSSTASALGKLQDVVNQNAQALN
OU053018/C.37/Lambda          QMAYRFNGIGVTQNVLYENQKLIANQFNSAIGKIQDSLSSTASALGKLQDVVNQNAQALN
OU268528/C.37/Lambda          QMAYRFNGIGVTQNVLYENQKLIANQFNSAIGKIQDSLSSTASALGKLQDVVNQNAQALN
OU100999/C.37/Lambda          QMAYRFNGIGVTQNVLYENQKLIANQFNSAIGKIQDSLSSTASALGKLQDVVNQNAQALN
MZ275295/C.37/Lambda          QMAYRFNGIGVTQNVLYENQKLIANQFNSAIGKIQDSLSSTASALGKLQDVVNQNAQALN
MZ275301/C.37/Lambda          QMAYRFNGIGVTQNVLYENQKLIANQFNSAIGKIQDSLSSTASALGKLQDVVNQNAQALN
MZ908711/C.37/Lambda          QMAYRFNGIGVTQNVLYENQKLIANQFNSAIGKIQDSLSSTASALGKLQDVVNQNAQALN
MZ496613/C.37/Lambda          QMAYRFNGIGVTQNVLYENQKLIANQFNSAIGKIQDSLSSTASALGKLQDVVNQNAQALN
OU517038/C.37/Lambda          QMAYRFNGIGVTQNVLYENQKLIANQFNSAIGKIQDSLSSTASALGKLQDVVNQNAQALN
MZ953277/C.37/Lambda          QMAYRFNGIGVTQNVLYENQKLIANQFNSAIGKIQDSLSSTASALGKLQDVVNQNAQALN
MZ942542/C.37/Lambda          QMAYRFNGIGVTQNVLYENQKLIANQFNSAIGKIQDSLSSTASALGKLQDVVNQNAQALN
MZ857990/C.37/Lambda          QMAYRFNGIGVTQNVLYENQKLIANQFNSAIGKIQDSLSSTASALGKLQDVVNQNAQALN
```

```
                                    970       980       990      1000      1010      1020
MN908947/Wuhan-Hu-1            TLVKQLSSNFGAISSVLNDILSRLDKVEAEVQIDRLITGRLQSLQTYVTQQLIRAAEIRA
BS001137/B.1.1.7/Alpha         TLVKQLSSNFGAISSVLNDILARLDKVEAEVQIDRLITGRLQSLQTYVTQQLIRAAEIRA
BS001138/B.1.1.7/Alpha         TLVKQLSSNFGAISSVLNDILARLDKVEAEVQIDRLITGRLQSLQTYVTQQLIRAAEIRA
BS001139/B.1.1.7/Alpha         TLVKQLSSNFGAISSVLNDILARLDKVEAEVQIDRLITGRLQSLQTYVTQQLIRAAEIRA
BS001140/B.1.1.7/Alpha         TLVKQLSSNFGAISSVLNDILARLDKVEAEVQIDRLITGRLQSLQTYVTQQLIRAAEIRA
MW913362/B.1.1.7/Alpha         TLVKQLSSNFGAISSVLNDILARLDKVEAEVQIDRLITGRLQSLQTYVTQQLIRAAEIRA
MZ266365/B.1.1.7/Alpha         TLVKQLSSNFGAISSVLNDILARLDKVEAEVQIDRLITGRLQSLQTYVTQQLIRAAEIRA
MZ350105/B.1.1.7/Alpha         TLVKQLSSNFGAISSVLNDILARLDKVEAEVQIDRLITGRLQSLQTYVTQQLIRAAEIRA
MZ350109/B.1.1.7/Alpha         TLVKQLSSNFGAISSVLNDILARLDKVEAEVQIDRLITGRLQSLQTYVTQQLIRAAEIRA
MZ888516/B.1.1.7/Alpha         TLVKQLSSNFGAISSVLNDILARLDKVEAEVQIDRLITGRLQSLQTYVTQQLIRAAEIRA
OU562607/B.1.1.7/Alpha         TLVKQLSSNFGAISSVLNDILARLDKVEAEVQIDRLITGRLQSLQTYVTQQLIRAAEIRA
OU514166/B.1.1.7/Alpha         TLVKQLSSNFGAISSVLNDILARLDKVEAEVQIDRLITGRLQSLQTYVTQQLIRAAEIRS
OU538851/B.1.1.7/Alpha         TLVKQLSSNFGAISSVLNDILARLDKVEAEVQIDRLITGRLQSLQTYVTQQLIRAAEIRA
OU538849/B.1.1.7/Alpha         TLVKQLSSNFGAISSVLNDILARLDKVEAEVQIDRLITGRLQSLQTYVTQQLIRAAEIRA
MZ895877/B.1.1.7/Alpha         TLVKQLSSNFGAISSVLNDILARLDKVEAEVQIDRLITGRLQSLQTYVTQQLIRAAEIRA
OU514176/B.1.1.7/Alpha         TLVKQLSSNFGAISSVLNDILARLDKVEAEVQIDRLITGRLQSLQTYVTQQLIRAAEIRA
OU562608/B.1.1.7/Alpha         TLVKQLSSNFGAISSVLNDILARLDKVEAEVQIDRLITGRLQSLQTYVTQQLIRAAEIRA
MW981442/B.1.351/Beta          TLVKQLSSNFGAISSVLNDILSRLDKVEAEVQIDRLITGRLQSLQTYVTQQLIRAAEIRA
MZ298839/B.1.351/Beta          TLVKQLSSNFGAISSVLNDILSRLDKVEAEVQIDRLITGRLQSLQTYVTQQLIRAAEIRA
MZ913100/B.1.351/Beta          TLVKQLSSNFGAISSVLNDILSRLDKVEAEVQIDRLITGRLQSLQTYVTQQLIRAAEIRA
MZ879365/B.1.351/Beta          TLVKQLSSNFGAISSVLNDILSRLDKVEAEVQIDRLITGRLQSLQTYVTQQLIRAAEIRA
OU525106/B.1.351/Beta          TLVKQLSSNFGAISSGLNDILSRLDKVEAEVQIDRLITGRLQSLQTYVTQQLIRAAEIRA
OU539352/B.1.351/Beta          TLVKQLSSNFGAISSVLNDILSRLDKVEAEVQIDRLITGRLQSLQTYVTQQLIRAAEIRA
MZ202314/B.1.351/Beta          TLVKQLSSNFGAISSVLNDILSRLDKVEAEVQIDRLITGRLQSLQTYVTQQLIRAAEIRA
MZ298840/B.1.351/Beta          TLVKQLSSNFGAISSVLNDILSRLDKVEAEVQIDRLITGRLQSLQTYVTQQLIRAAEIRA
MZ376663/B.1.351/Beta          TLVKQLSSNFGAISSVLNDILSRLDKVEAEVQIDRLITGRLQSLQTYVTQQLIRAAEIRA
LC643036/B.1.617.2/Delta       TLVKQLSSNFGAISSVLNDILSRLDKVEAEVQIDRLITGRLQSLQTYVTQQLIRAAEIRA
LC643044/B.1.617.2/Delta       TLVKQLSSNFGAISSVLNDILSRLDKVEAEVQIDRLITGRLQSLQTYVTQQLIRAAEIRA
MZ853946/B.1.617.2/Delta       TLVKQLSSNFGAISSVLNDILSRLDKVEAEVQIDRLITGRLQSLQTYVTQQLIRAAEIRA
MZ854386/B.1.617.2/Delta       TLVKQLSSNFGAISSVLNDILSRLDKVEAEVQIDRLITGRLQSLQTYVTQQLIRAAEIRA
MZ888532/B.1.617.2/Delta       TLVKQLSSNFGAISSVLNDILSRLDKVEAEVQIDRLITGRLQSLQTYVTQQLIRAAEIRA
MZ888533/B.1.617.2/Delta       TLVKQLSSNFGAISSVLNDILSRLDKVEAEVQIDRLITGRLQSLQTYVTQQLIRAAEIRA
MZ896136/B.1.617.2/Delta       TLVKQLSSNFGAISSVLNDILSRLDKVEAEVQIDRLITGRLQSLQTYVTQQLIRAAEIRA
OU539261/B.1.617.2/Delta       TLVKQLSSNFGAISSVLNDILSRLDKVEAEVQIDRLITGRLQSLQTYVTQQLIRAAEIRA
MZ854389/B.1.617.2/Delta       TLVKQLSSNFGAISSVLNDILSRLDKVEAEVQIDRLITGRLQSLQTYVTQQLIRAAEIRA
OU562629/B.1.617.2/Delta       TLVKQLSSNFGAISSVLNDILSRLDKVEAEVQIDRLITGRLQSLQTYVTQQLIRAAEIRA
OU539690/B.1.617.2/Delta       TLVKQLSSNFGAISSVLNDILSRLDKVEAEVQIDRLITGRLQSLQTYVTQQLIRAAEIRA
MZ854387/B.1.617.2/Delta       TLVKQLSSNFGAISSVLNDILSRLDKVEAEVQIDRLITGRLQSLQTYVTQQLIRAAEIRA
OU539784/P.1/Gamma             TLVKQLSSNFGAISSVLNDILSRLDKVEAEVQIDRLITGRLQSLQTYVTQQLIRAAEIRA
OU539830/P.1/Gamma             TLVKQLSSNFGAISSVLNDILSRLDKVEAEVQIDRLITGRLQSLQTYVTQQLIRAAEIRA
OU539894/P.1/Gamma             TLVKQLSSNFGAISSVLNDILSRLDKVEAEVQIDRLITGRLQSLQTYVTQQLIRAAEIRA
MZ477748/P.1/Gamma             TLVKQLSSNFGAISSVLNDILSRLDKVEAEVQIDRLITGRLQSLQTYVTQQLIRAAEIRA
MZ477746/P.1/Gamma             TLVKQLSSNFGAISSVLNDILSRLDKVEAEVQIDRLITGRLQSLQTYVTQQLIRAAEIRA
MW938104/P.1/Gamma             TLVKQLSSNFGAISSVLNDILSRLDKVEAEVQIDRLITGRLQSLQTYVTQQLIRAAEIRA
MZ896179/P.1/Gamma             TLVKQLSSNFGAISSVLNDILSRLDKVEAEVQIDRLITGRLQSLQTYVTQQLIRAAEIRA
MZ896359/P.1/Gamma             TLVKQLSSNFGAISSVLNDILSRLDKVEAEVQIDRLITGRLQSLQTYVTQQLIRAAEIRA
MZ896669/P.1/Gamma             TLVKQLSSNFGAISSVLNDILSRLDKVEAEVQIDRLITGRLQSLQTYVTQQLIRAAEIRA
OU565182/P.1/Gamma             TLVKQLSSNFGAISSVLNDILSRLDKVEAEVQIDRLITGRLQSLQTYVTQQLIRAAEIRA
OU492255/P.1/Gamma             TLVKQLSSNFGAISSVLNDILSRLDKVEAEVQIDRLITGRLQSLQTYVTQQLIRAAEIRA
MZ911221/B.1.427/Epsilon       TLVKQLSSNFGAISSVLNDILSRLDKVEAEVQIDRLITGRLQSLQTYVTQQLIRAAEIRA
MZ375854/B.1.427/Epsilon       TLVKQLSSNFGAISSVLNDILSRLDKVEAEVQIDRLITGRLQSLQTYVTQQLIRAAEIRA
MZ375858/B.1.427/Epsilon       TLVKQLSSNFGAISSVLNDILSRLDKVEAEVQIDRLITGRLQSLQTYVTQQLIRAAEIRA
MZ943834/B.1.429/Epsilon       TLVKQLSSNFGAISSVLNDILSRLDKVEAEVQIDRLITGRLQSLQTYVTQQLIRAAEIRA
MZ277390/B.1.429/Epsilon       TLVKQLSSNFGAISSVLNDILSRLDKVEAEVQIDRLITGRLQSLQTYVTQQLIRAAEIRA
OU282717/B.1.429/Epsilon       TLVKQLSSNFGAISSVLNDILSRLDKVEAEVQIDRLITGRLQSLQTYVTQQLIRAAEIRA
OU235436/B.1.429/Epsilon       TLVKQLSSNFGAISSVLNDILSRLDKVEAEVQIDRLITGRLQSLQTYVTQQLIRAAEIRA
MZ687447/B.1.429/Epsilon       TLVKQLSSNFGAISSVLNDILSRLDKVEAEVQIDRLITGRLQSLQTYVTQQLIRAAEIRA
MZ538882/B.1.617.1/Kappa       TLVKQLSSNFGAISSVLNDILSRLDKVEAEVQIDRLITGRLQSLQTYVTQQLIRAAEIRA
MZ724425/B.1.617.1/Kappa       TLVKQLSSNFGAISSVLNDILSRLDKVEAEVQIDRLITGRLQSLQTYVTQQLIRAAEIRA
MZ363839/B.1.617.1/Kappa       TLVKQLSSNFGAISSVLNDILSRLDKVEAEVQIDRLITGRLQSLQTYVTQQLIRAAEIRA
MZ332524/B.1.617.1/Kappa       TLVKQLSSNFGAISSVLNDILSRLDKVEAEVQIDRLITGRLQSLQTYVTQQLIRAAEIRA
MZ332527/B.1.617.1/Kappa       TLVKQLSSNFGAISSVLNDILSRLDKVEAEVQIDRLITGRLQSLQTYVTQQLIRAAEIRA
LC633761/B.1.617.1/Kappa       TLVKQLSSNFGAISSVLNDILSRLDKVEAEVQIDRLITGRLQSLQTYVTQQLIRAAEIRA
MZ562746/B.1.617.1/Kappa       TLVKQLSSNFGAISSVLNDILSRLDKVEAEVQIDRLITGRLQSLQTYVTQQLIRAAEIRA
MZ571142/B.1.617.1/Kappa       TLVKQLSSNFGAISSVLNDILSRLDKVEAEVQIDRLITGRLQSLQTYVTQQLIRAAEIRA
OU168413/B.1.617.1/Kappa       TLVKQLSSNFGAISSVLNDILSRLDKVEAEVQIDRLITGRLQSLQTYVTQQLIRAAEIRA
MZ026886/B.1.617.1/Kappa       TLVKQLSSNFGAISSVLNDILSRLDKVEAEVQIDRLITGRLQSLQTYVTQQLIRAAEIRA
OU172184/B.1.617.1/Kappa       TLVKQLSSNFGAISSVLNDILSRLDKVEAEVQIDRLITGRLQSLQTYVTQQLIRAAEIRA
MZ397173/B.1.617.1/Kappa       TLVKQLSSNFGAISSVLNDILSRLDKVEAEVQIDRLITGRLQSLQTYVTQQLIRAAEIRA
MZ397176/B.1.617.1/Kappa       TLVKQLSSNFGAISSVLNDILSRLDKVEAEVQIDRLITGRLQSLQTYVTQQLIRAAEIRA
MZ397175/B.1.617.1/Kappa       TLVKQLSSNFGAISSVLNDILSRLDKVEAEVQIDRLITGRLQSLQTYVTQQLIRAAEIRA
MZ397177/B.1.617.1/Kappa       TLVKQLSSNFGAISSVLNDILSRLDKVEAEVQIDRLITGRLQSLQTYVTQQLIRAAEIRA
OU290035/B.1.617.1/Kappa       TLVKQLSSNFGAISSVLNDILSRLDKVEAEVQIDRLITGRLQSLQTYVTQQLIRAAEIRA
OU265178/B.1.617.1/Kappa       TLVKQLSSNFGAISSVLNDILSRLDKVEAEVQIDRLITGRLQSLQTYVTQQLIRAAEIRA
OU254949/B.1.617.1/Kappa       TLVKQLSSNFGAISSVLNDILSRLDKVEAEVQIDRLITGRLQSLQTYVTQQLIRAAEIRA
OU247018/B.1.617.1/Kappa       TLVKQLSSNFGAISSVLNDILSRLDKVEAEVQIDRLITGRLQSLQTYVTQQLIRAAEIRA
OU061269/B.1.617.1/Kappa       TLVKQLSSNFGAISSVLNDILSRLDKVEAEVQIDRLITGRLQSLQTYVTQQLIRAAEIRA
OU182966/B.1.617.1/Kappa       TLVKQLSSNFGAISSVLNDILSRLDKVEAEVQIDRLITGRLQSLQTYVTQQLIRAAEIRA
MZ746235/B.1.617.1/Kappa       TLVKQLSSNFGAISSVLNDILSRLDKVEAEVQIDRLITGRLQSLQTYVTQQLIRAAEIRA
MZ487800/B.1.617.1/Kappa       TLVKQLSSNFGAISSVLNDILSRLDKVEAEVQIDRLITGRLQSLQTYVTQQLIRAAEIRA
OU322235/B.1.617.1/Kappa       TLVKQLSSNFGAISSVLNDILSRLDKVEAEVQIDRLITGRLQSLQTYVTQQLIRAAEIRA
MZ953185/C.37/Lambda           TLVKQLSSNFGAISSVLNDILSRLDKVEAEVQIDRLITGRLQSLQTYVTQQLIRAAEIRA
MZ937666/C.37/Lambda           TLVKQLSSNFGAISSVLNDILSRLDKVEAEVQIDRLITGRLQSLQTYVTQQLIRAAEIRA
OU356036/C.37/Lambda           TLVKQLSSNFGAISSVLNDILSRLDKVEAEVQIDRLITGRLQSLQTYVTQQLIRAAEIRA
OU351967/C.37/Lambda           TLVKQLSSNFGAISSVLNDILSRLDKVEAEVQIDRLITGRLQSLQTYVTQQLIRAAEIRA
OU053018/C.37/Lambda           TLVKQLSSNFGAISSVLNDILSRLDKVEAEVQIDRLITGRLQSLQTYVTQQLIRAAEIRA
OU268528/C.37/Lambda           TLVKQLSSNFGAISSVLNDILSRLDKVEAEVQIDRLITGRLQSLQTYVTQQLIRAAEIRA
OU100999/C.37/Lambda           TLVKQLSSNFGAISSVLNDILSRLDKVEAEVQIDRLITGRLQSLQTYVTQQLIRAAEIRA
MZ275295/C.37/Lambda           TLVKQLSSNFGAISSVLNDILSRLDKVEAEVQIDRLITGRLQSLQTYVTQQLIRAAEIRA
MZ275301/C.37/Lambda           TLVKQLSSNFGAISSVLNDILSRLDKVEAEVQIDRLITGRLQSLQTYVTQQLIRAAEIRA
MZ908711/C.37/Lambda           TLVKQLSSNFGAISSVLNDILSRLDKVEAEVQIDRLITGRLQSLQTYVTQQLIRAAEIRA
MZ496613/C.37/Lambda           TLVKQLSSNFGAISSVLNDILSRLDKVEAEVQIDRLITGRLQSLQTYVTQQLIRAAEIRA
OU517038/C.37/Lambda           TLVKQLSSNFGAISSVLNDILSRLDKVEAEVQIDRLITGRLQSLQTYVTQQLIRAAEIRA
MZ953277/C.37/Lambda           TLVKQLSSNFGAISSVLNDILSRLDKVEAEVQIDRLITGRLQSLQTYVTQQLIRAAEIRA
MZ942542/C.37/Lambda           TLVKQLSSNFGAISSVLNDILSRLDKVEAEVQIDRLITGRLQSLQTYVTQQLIRAAEIRA
MZ857990/C.37/Lambda           TLVKQLSSNFGAISSVLNDILSRLDKVEAEVQIDRLITGRLQSLQTYVTQQLIRAAEIRA
```

```
                                   1030      1040      1050      1060      1070      1080
MN908947/Wuhan-Hu-1           SANLAATKMSECVLGQSKRVDFCGKGYHLMSFPQSAPHGVVFLHVTYVPAQEKNFTTAPA
BS001137/B.1.1.7/Alpha        SANLAATKMSECVLGQSKRVDFCGKGYHLMSFPQSAPHGVVFLHVTYVPAQEKNFTTAPA
BS001138/B.1.1.7/Alpha        SANLAATKMSECVLGQSKRVDFCGKGYHLMSFPQSAPHGVVFLHVTYVPAQEKNFTTAPA
BS001139/B.1.1.7/Alpha        SANLAATKMSECVLGQSKRVDFCGKGYHLMSFPQSAPHGVVFLHVTYVPAQEKNFTTAPA
BS001140/B.1.1.7/Alpha        SANLAATKMSECVLGQSKRVDFCGKGYHLMSFPQSAPHGVVFLHVTYVPAQEKNFTTAPA
MW913362/B.1.1.7/Alpha        SANLAATKMSECVLGQSKRVDFCGKGYHLMSFPQSAPHGVVFLHVTYVPAQEKNFTTAPA
MZ266365/B.1.1.7/Alpha        SANLAATKMSECVLGQSKRVDFCGKGYHLMSFPQSAPHGVVFLHVTYVPAQEKNFTTAPA
MZ350105/B.1.1.7/Alpha        SANLAATKMSECVLGQSKRVDFCGKGYHLMSFPQSAPHGVVFLHVTYVPAQEKNFTTAPA
MZ350109/B.1.1.7/Alpha        SANLAATKMSECVLGQSKRVDFCGKGYHLMSFPQSAPHGVVFLHVTYVPAQEKNFTTAPA
MZ888516/B.1.1.7/Alpha        SANLAATKMSECVLGQSKRVDFCGKGYHLMSFPQSAPHGVVFLHVTYVPAQEKNFTTAPA
OU562607/B.1.1.7/Alpha        SANLAATKMSECVLGQSKRVDFCGKGYHLMSFPQSAPHGVVFLHVTYVPAQEKNFTTAPA
OU514166/B.1.1.7/Alpha        SANLAATKMSECVLGQSKRVDFCGKGYHLMSFPQSAPHGVVFLHVTYVPAQEKNFTTAPA
OU538851/B.1.1.7/Alpha        SANLAATKMSECVLGQSKRVDFCGKGYHLMSFPQSAPHGVVFLHVTYVPAQEKNFTTAPA
OU538849/B.1.1.7/Alpha        SANLAATKMSECVLGQSKRVDFCGKGYHLMSFPQSAPHGVVFLHVTYVPAQEKNFTTAPA
MZ895877/B.1.1.7/Alpha        SANLAATKMSECVLGQSKRVDFCGKGYHLMSFPQSAPHGVVFLHVTYVPAQEKNFTTAPA
OU514176/B.1.1.7/Alpha        SANLAATKMSECVLGQSKRVDFCGKGYHLMSFPQSAPHGVVFLHVTYVPAQEKNFTTAPA
OU562608/B.1.1.7/Alpha        SANLAATKMSECVLGQSKRVDFCGKGYHLMSFPQSAPHGVVFLHVTYVPAQEKNFTTAPA
MW981442/B.1.351/Beta         SANLAATKMSECVLGQSKRVDFCGKGYHLMSFPQSAPHGVVFLHVTYVPAQEKNFTTAPA
MZ298839/B.1.351/Beta         SANLAATKMSECVLGQSKRVDFCGKGYHLMSFPQSAPHGVVFLHVTYVPAQEKNFTTAPA
MZ913100/B.1.351/Beta         SANLAATKMSECVLGQSKRVDFCGKGYHLMSFPQSAPHGVVFLHVTYVPAQEKNFTTAPA
MZ879365/B.1.351/Beta         SANLAATKMSECVLGQSKRVDFCGKGYHLMSFPQSAPHGVVFLHVTYVPAQEKNFTTAPA
OU525106/B.1.351/Beta         SANLAATKMSECVLGQSKRVDFCGKGYHLMSFPQSAPHGVVFLHVTYVPAQEKNFTTAPA
OU539352/B.1.351/Beta         SANLAATKMSECVLGQSKRVDFCGKGYHLMSFPQSAPHGVVFLHVTYVPAQEKNFTTAPA
MZ202314/B.1.351/Beta         SANLAATKMSECVLGQSKRVDFCGKGYHLMSFPQSAPHGVVFLHVTYVPAQEKNFTTAPA
MZ298840/B.1.351/Beta         SANLAATKMSECVLGQSKRVDFCGKGYHLMSFPQSAPHGVVFLHVTYVPAQEKNFTTAPA
MZ376663/B.1.351/Beta         SANLAATKMSECVLGQSKRVDFCGKGYHLMSFPQSAPHGVVFLHVTYVPAQEKNFTTAPA
LC643036/B.1.617.2/Delta      SANLAATKMSECVLGQSKRVDFCGKGYHLMSFPQSAPHGVVFLHVTYVPAQEKNFTTAPA
LC643044/B.1.617.2/Delta      SANLAATKMSECVLGQSKRVDFCGKGYHLMSFPQSAPHGVVFLHVTYVPAQEKNFTTAPA
MZ853946/B.1.617.2/Delta      SANLAATKMSECVLGQSKRVDFCGKGYHLMSFPQSAPHGVVFLHVTYVPAQEKNFTTAPA
MZ854386/B.1.617.2/Delta      SANLAATKMSECVLGQSKRVDFCGKGYHLMSFPQSAPHGVVFLHVTYVPAQEKNFTTAPA
MZ888532/B.1.617.2/Delta      SANLAATKMSECVLGQSKRVDFCGKGYHLMSFPQSAPHGVVFLHVTYVPAQEKNFTTAPA
MZ888533/B.1.617.2/Delta      SANLAATKMSECVLGQSKRVDFCGKGYHLMSFPQSAPHGVVFLHVTYVPAQEKNFTTAPA
MZ896136/B.1.617.2/Delta      SANLAATKMSECVLGQSKRVDFCGKGYHLMSFPQSAPHGVVFLHVTYVPAQEKNFTTAPA
OU539261/B.1.617.2/Delta      SANLAATKMSECVLGQSKRVDFCGKGYHLMSFPQSAPHGVVFLHVTYVPAQEKNFTTAPA
MZ854389/B.1.617.2/Delta      SANLAATKMSECVLGQSKRVDFCGKGYHLMSFPQSAPHGVVFLHVTYVPAQEKNFTTAPA
OU562629/B.1.617.2/Delta      SANLAATKMSECVLGQSKRVDFCGKGYHLMSFPQSAPHGVVFLHVTYVPAQEKNFTTAPA
OU539690/B.1.617.2/Delta      SANLAATKMSECVLGQSKRVDFCGKGYHLMSFPQSAPHGVVFLHVTYVPAQEKNFTTAPA
MZ854387/B.1.617.2/Delta      SANLAATKMSECVLGQSKRVDFCGKGYHLMSFPQSAPHGVVFLHVTYVPAQEKNFTTAPA
OU539784/P.1/Gamma            SANLAAIKMSECVLGQSKRVDFCGKGYHLMSFPQSAPHGVVFLHVTYVPAQEKNFTTAPA
OU539830/P.1/Gamma            SANLAAIKMSECVLGQSKRVDFCGKGYHLMSFPQSAPHGVVFLHVTYVPAQEKNFTTAPA
OU539894/P.1/Gamma            SANLAAIKMSECVLGQSKRVDFCGKGYHLMSFPQSAPHGVVFLHVTYVPAQEKNFTTAPA
MZ477748/P.1/Gamma            SANLAAIKMSECVLGQSKRVDFCGKGYHLMSFPQSAPHGVVFLHVTYVPAQEKNFTTAPA
MZ477746/P.1/Gamma            SANLAAIKMSECVLGQSKRVDFCGKGYHLMSFPQSAPHGVVFLHVTYVPAQEKNFTTAPA
MW938104/P.1/Gamma            SANLAAIKMSECVLGQSKRVDFCGKGYHLMSFPQSAPHGVVFLHVTYVPAQEKNFTTAPA
MZ896179/P.1/Gamma            SANLAAIKMSECVLGQSKRVDFCGKGYHLMSFPQSAPHGVVFLHVTYVPAQEKNFTTAPA
MZ896359/P.1/Gamma            SANLAAIKMSECVLGQSKRVDFCGKGYHLMSFPQSAPHGVVFLHVTYVPAQEKNFTTAPA
MZ896669/P.1/Gamma            SANLAAIKMSECVLGQSKRVDFCGKGYHLMSFPQSAPHGVVFLHVTYVPAQEKNFTTAPA
OU565182/P.1/Gamma            SANLAAIKMSECVLGQSKRVDFCGKGYHLMSFPQSAPHGVVFLHVTYVPAQEKNFTTAPA
OU492255/P.1/Gamma            SANLAAIKMSECVLGQSKRVDFCGKGYHLMSFPQSAPHGVVFLHVTYVPAQEKNFTTAPA
MZ911221/B.1.427/Epsilon      SANLAATKMSECVLGQSKRVDFCGKGYHLMSFPQSAPHGVVFLHVTYVPAQEKNFTTAPA
MZ375854/B.1.427/Epsilon      SANLAATKMSECVLGQSKRVDFCGKGYHLMSFPQSAPHGVVFLHVTYVPAQEKNFTTAPA
MZ375858/B.1.427/Epsilon      SANLAATKMSECVLGQSKRVDFCGKGYHLMSFPQSAPHGVVFLHVTYVPAQEKNFTTAPA
MZ943834/B.1.429/Epsilon      SANLAATKMSECVLGQSKRVDFCGKGYHLMSFPQSAPHGVVFLHVTYVPAQEKNFTTAPA
MZ277390/B.1.429/Epsilon      SANLAATKMSECVLGQSKRVDFCGKGYHLMSFPQSAPHGVVFLHVTYVPAQEKNFTTAPA
OU282717/B.1.429/Epsilon      SANLAATKMSECVLGQSKRVDFCGKGYHLMSFPQSAPHGVVFLHVTYVPAQEKNFTTAPA
OU235436/B.1.429/Epsilon      SANLAATKMSECVLGQSKRVDFCGKGYHLMSFPQSAPHGVVFLHVTYVPAQEKNFTTAPA
MZ687447/B.1.429/Epsilon      SANLAATKMSECVLGQSKRVDFCGKGYHLMSFPQSAPHGVVFLHVTYVPAQEKNFTTAPA
MZ538882/B.1.617.1/Kappa      SANLAATKMSECVLGQSKRVDFCGKGYHLMSFPQSAPHGVVFLHVTYVPAHEKNFTTAPA
MZ724425/B.1.617.1/Kappa      SANLAATKMSECVLGQSKRVDFCGKGYHLMSFPQSAPHGVVFLHVTYVPAHEKNFTTAPA
MZ363839/B.1.617.1/Kappa      SANLAATKMSECVLGQSKRVDFCGKGYHLMSFPQSAPHGVVFLHVTYVPAHEKNFTTAPA
MZ332524/B.1.617.1/Kappa      SANLAATKMSECVLGQSKRVDFCGKGYHLMSFPQSAPHGVVFLHVTYVPAHEKNFTTAPA
MZ332527/B.1.617.1/Kappa      SANLAATKMSECVLGQSKRVDFCGKGYHLMSFPQSAPHGVVFLHVTYVPAHEKNFTTAPA
LC633761/B.1.617.1/Kappa      SANLAATKMSECVLGQSKRVDFCGKGYHLMSFPQSAPHGVVFLHVTYVPAHEKNFTTAPA
MZ562746/B.1.617.1/Kappa      SANLAATKMSECVLGQSKRVDFCGKGYHLMSFPQSAPHGVVFLHVTYVPAHEKNFTTAPA
MZ571142/B.1.617.1/Kappa      SANLAATKMSECVLGQSKRVDFCGKGYHLMSFPQSAPHGVVFLHVTYVPAHEKNFTTAPA
OU168413/B.1.617.1/Kappa      SANLAATKMSECVLGQSKRVDFCGKGYHLMSFPQSAPHGVVFLHVTYVPAHEKNFTTAPA
MZ026886/B.1.617.1/Kappa      SANLAATKMSECVLGQSKRVDFCGKGYHLMSFPQSAPHGVVFLHVTYVPAHEKNFTTAPA
OU172184/B.1.617.1/Kappa      SANLAATKMSECVLGQSKRVDFCGKGYHLMSFPQSAPHGVVFLHVTYVSAHEKNFTTAPA
MZ397173/B.1.617.1/Kappa      SANLAATKMSECVLGQSKRVDFCGKGYHLMSFPQSAPHGVVFLHVTYVPAHEKNFTTAPA
MZ397176/B.1.617.1/Kappa      SANLAATKMSECVLGQSKRVDFCGKGYHLMSFPQSAPHGVVFLHVTYVPAHEKNFTTAPA
MZ397175/B.1.617.1/Kappa      SANLAATKMSECVLGQSKRVDFCGKGYHLMSFPQSAPHGVVFLHVTYVPAHEKNFTTAPA
MZ397177/B.1.617.1/Kappa      SANLAATKMSECVLGQSKRVDFCGKGYHLMSFPQSAPHGVVFLHVTYVPAHEKNFTTAPA
OU290035/B.1.617.1/Kappa      SANLAATKMSECVLGQSKRVDFCGKGYHLMSFPQSAPHGVVFLHVTYVPAHEKNFTTAPA
OU265178/B.1.617.1/Kappa      SANLAATKMSECVLGQSKRVDFCGKGYHLMSFPQSAPHGVVFLHVTYVPAHEKNFTTAPA
OU254949/B.1.617.1/Kappa      SANLAATKMSECVLGQSKRVDFCGKGYHLMSFPQSAPHGVVFLHVTYVPAHEKNFTTAPA
OU247018/B.1.617.1/Kappa      SANLAATKMSECVLGQSKRVDFCGKGYHLMSFPQSAPHGVVFLHVTYVPAHEKNFTTAPA
OU061269/B.1.617.1/Kappa      SANLAATKMSECVLGQSKRVDFCGKGYHLMSFPQSAPHGVVFLHVTYVPAHEKNFTTAPA
OU182966/B.1.617.1/Kappa      SANLAATKMSECVLGQSKRVDFCGKGYHLMSFPQSAPHGVVFLHVTYVPAHEKNFTTAPA
MZ746235/B.1.617.1/Kappa      SANLAATKMSECVLGQSKRVDFCGKGYHLMSFPQSAPHGVVFLHVTYVPAHEKNFTTAPA
MZ487800/B.1.617.1/Kappa      SANLAATKMSECVLGQSKRVDFCGKGYHLMSFPQSAPHGVVFLHVTYVPAHEKNFTTAPA
OU322235/B.1.617.1/Kappa      SANLAATKMSECVLGQSKRVDFCGKGYHLMSFPQSAPHGVVFLHVTYVPAHEKNFTTAPA
MZ953185/C.37/Lambda          SANLAATKMSECVLGQSKRVDFCGKGYHLMSFPQSAPHGVVFLHVTYVPAQEKNFTTAPA
MZ937666/C.37/Lambda          SANLAATKMSECVLGQSKRVDFCGKGYHLMSFPQSAPHGVVFLHVTYVPAQEKNFTTAPA
OU356036/C.37/Lambda          SANLAATKMSECVLGQSKRVDFCGKGYHLMSFPQSAPHGVVFLHVTYVPAQEKNFTTAPA
OU351967/C.37/Lambda          SANLAATKMSECVLGQSKRVDFCGKGYHLMSFPQSAPHGVVFLHVTYVPAQEKNFTTAPA
OU053018/C.37/Lambda          SANLAATKMSECVLGQSKRVDFCGKGYHLMSFPQSAPHGVVFLHVTYVPAQEKNFTTAPA
OU268528/C.37/Lambda          SANLAATKMSECVLGQSKRVDFCGKGYHLMSFPQSAPHGVVFLHVTYVPAQEKNFTTAPA
OU100999/C.37/Lambda          SANLAATKMSECVLGQSKRVDFCGKGYHLMSFPQSAPHGVVFLHVTYVPAQEKNFTTAPA
MZ275295/C.37/Lambda          SANLAATKMSECVLGQSKRVDFCGKGYHLMSFPQSAPHGVVFLHVTYVPAQEKNFTTAPA
MZ275301/C.37/Lambda          SANLAATKMSECVLGQSKRVDFCGKGYHLMSFPQSAPHGVVFLHVTYVPAQEKNFTTAPA
MZ908711/C.37/Lambda          SANLAATKMSECVLGQSKRVDFCGKGYHLMSFPQSAPHGVVFLHVTYVPAQEKNFTTAPA
MZ496613/C.37/Lambda          SANLAATKMSECVLGQSKRVDFCGKGYHLMSFPQSAPHGVVFLHVTYVPAQEKNFTTAPA
OU517038/C.37/Lambda          SANLAATKMSECVLGQSKRVDFCGKGYHLMSFPQSAPHGVVFLHVTYVPAQEKNFTTAPA
MZ953277/C.37/Lambda          SANLAATKMSECVLGQSKRVDFCGKGYHLMSFPQSAPHGVVFLHVTYVPAQEKNFTTAPA
MZ942542/C.37/Lambda          SANLAATKMSECVLGQSKRVDFCGKGYHLMSFPQSAPHGVVFLHVTYVPAQEKNFTTAPA
MZ857990/C.37/Lambda          SANLAATKMSECVLGQSKRVDFCGKGYHLMSFPQSAPHGVVFLHVTYVPAQEKNFTTAPA
```

	1090 1100 1110 1120 1130 1140
MN908947/Wuhan-Hu-1	ICHDGKAHFPREGVFVSNGTHWFVTQRNFYEPQIITTDNTFVSGNCDVVIGIVNNTVYDP
BS001137/B.1.1.7/Alpha	ICHDGKAHFPREGVFVSNGTHWFVTQRNFYEPQIITTHNTFVSGNCDVVIGIVNNTVYDP
BS001138/B.1.1.7/Alpha	ICHDGKAHFPREGVFVSNGTHWFVTQRNFYEPQIITTHNTFVSGNCDVVIGIVNNTVYDP
BS001139/B.1.1.7/Alpha	ICHDGKAHFPREGVFVSNGTHWFVTQRNFYEPQIITTHNTFVSGNCDVVIGIVNNTVYDP
BS001140/B.1.1.7/Alpha	ICHDGKAHFPREGVFVSNGTHWFVTQRNFYEPQIITTHNTFVSGNCDVVIGIVNNTVYDP
MW913362/B.1.1.7/Alpha	ICHDGKAHFPREGVFVSNGTHWFVTQRNFYEPQIITTHNTFVSGNCDVVIGIVNNTVYDP
MZ266365/B.1.1.7/Alpha	ICHDGKAHFPREGVFVSNGTHWFVTQRNFYEPQIITTHNTFVSGNCDVVIGIVNNTVYDP
MZ350105/B.1.1.7/Alpha	ICHDGKAHFPREGVFVSNGTHWFVTQRNFYEPQIITTHNTFVSGNCDVVIGIVNNTVYDP
MZ350109/B.1.1.7/Alpha	ICHDGKAHFPREGVFVSNGTHWFVTQRNFYEPQIITTHNTFVSGNCDVVIGIVNNTVYDP
MZ888516/B.1.1.7/Alpha	ICHDGKAHFPREGVFVSNGTHWFVTQRNFYEPQIITTHNTFVSGNCDVVIGIVNNTVYDP
OU562607/B.1.1.7/Alpha	ICHDGKAHFPREGVFVSNGTHWFVTQRNFYEPQIITTHNTFVSGNCDVVIGIVNNTVYDP
OU514166/B.1.1.7/Alpha	ICHDGKAHFPREGVFVSNGTHWFVTQRNFYEPQIITTHNTFVSGNCDVVIGIVNNTVYDP
OU538851/B.1.1.7/Alpha	ICHDGKAHFPREGVFVSNGTHWFVTQRNFYEPQIITTHNTFVSGNCDVVIGIVNNTVYDP
OU538849/B.1.1.7/Alpha	ICHDGKAHFPREGVFVSNGTHWFVTQRNFYEPQIITTHNTFVSGNCDVVIGIVNNTVYDP
MZ895877/B.1.1.7/Alpha	ICHDGKAHFPREGVFVSNGTHWFVTQRNFYEPQIITTHNTFVSGNCDVVIGIVNNTVYDP
OU514176/B.1.1.7/Alpha	ICHDGKAHFPREGVFVSNGTHWFVTQRNFYEPQIITTHNTFVSGNCDVVIGIVNNTVYDP
OU562608/B.1.1.7/Alpha	ICHDGKAHFPREGVFVSNGTHWFVTQRNFYEPQIITTHNTFVSGNCDVVIGIVNNTVYDP
MW981442/B.1.351/Beta	ICHDGKAHFPREGVFVSNGTHWFVTQRNFYEPQIITTDNTFVSGNCDVVIGIVNNTVYDP
MZ298839/B.1.351/Beta	ICHDGKAHFPREGVFVSNGTHWFVTQRNFYEPQIITTDNTFVSCNCDVVIGIVNNTVYDP
MZ913100/B.1.351/Beta	ICHDGKAHFPREGVFVSNGTHWFVTQRNFYEPQIITTDNTFVSGNCDVVIGIVNNTVYDP
MZ879365/B.1.351/Beta	ICHDGKAHFPREGVFVSNGTHWFVTQRNFYEPQIITTDNTFVSGNCDVVIGIVNNTVYDP
OU525106/B.1.351/Beta	ICHDGKAHFPREGVFVSNGTHWFVTQRNFYEPQIITTDNTFVSGNCDVVIGIVNNTVYDP
OU539352/B.1.351/Beta	ICHDGKAHFPREGVFVSNGTHWFVTQRNFYEPQIITTDNTFVSGNCDVVIGIVNNTVYDP
MZ202314/B.1.351/Beta	ICHDGKAHFPREGVFVSNGTHWFVTQRNFYEPQIITTDNTFVSGNCDVVIGIVNNTVYDP
MZ298840/B.1.351/Beta	ICHDGKAHFPREGVFVSNGTHWFVTQRNFYEPQIITTDNTFVSCNCDVVIGIVNNTVYDP
MZ376663/B.1.351/Beta	ICHDGKAHFPREGVFVSNGTHWFVTQRNFYEPQIITTDNTFVSGNCDVVIGIVNNTVYDP
LC643036/B.1.617.2/Delta	ICHDGKAHFPREGVFVSNGTHWFLTQRNFYEPQIITTDNTFVSGNCDVVIGIVNNTVYDP
LC643044/B.1.617.2/Delta	ICHDGKAHFPREGVFVSNGTHWFLTQRNFYEPQIITTDNTFVSGNCDVVIGIVNNTVYDP
MZ853946/B.1.617.2/Delta	ICHDGKAHFPREGVFVSNGTHWFVTQRNFYEPQIITTDNTFVSGNCDVVIGIVNNTVYDP
MZ854386/B.1.617.2/Delta	ICHDGKAHFPREGVFVSNGTHWFVTQRNFYEPQIITTDNTFVSGNCDVVIGIVNNTVYDP
MZ888532/B.1.617.2/Delta	ICHDGKAHFPREGVFVSNGTHWFVTQRNFYEPQIITTDNTFVSGNCDVVIGIVNNTVYDP
MZ888533/B.1.617.2/Delta	ICHDGKAHFPREGVFVSNGTHWFVTQRNFYEPQIITTDNTFVSGNCDVVIGIVNNTVYDP
MZ896136/B.1.617.2/Delta	ICHDGKAHFPREGVFVSNGTHWFVTQRNFYEPQIITTDNTFVSGNCDVVIGIVNNTVYDP
OU539261/B.1.617.2/Delta	ICHDGKAHFPREGVFVSNGTHWFVTQRNFYEPQIITTDNTFVSGNCDVVIGIVNNTVYDP
MZ854389/B.1.617.2/Delta	ICHDGKAHFPREGVFVSNGTHWFVTQRNFYEPQIITTDNTFVSGNCDVVIGIVNNTVYDP
OU562629/B.1.617.2/Delta	ICHDGKAHFPREGVFVSNGTHWFVTQRNFYEPQIITTDNTFVSGNCDVVIGIVNNTVYDP
OU539690/B.1.617.2/Delta	ICHDGKAHFPREGVFVSNGTHWFVTQRNFYEPQIITTDNTFVSGNCDVVIGIVNNTVYDP
MZ854387/B.1.617.2/Delta	ICHDGKAHFPREGVFVSNGTHWFVTQRNFYEPQIITTDNTFVSGNCDVVIGIVNNTVYDP
OU539784/P.1/Gamma	ICHDGKAHFPREGVFVSNGTHWFVTQRNFYEPQIITTDNTFVSGNCDVVIGIVNNTVYDP
OU539830/P.1/Gamma	ICHDGKAHFPREGVFVSNGTHWFVTQRNFYEPQIITTDNTFVSGNCDVVIGIVNNTVYDP
OU539894/P.1/Gamma	ICHDGKAHFPREGVFVSNGTHWFVTQRNFYEPQIITTDNTFVSGNCDVVIGIVNNTVYDP
MZ477748/P.1/Gamma	ICHDGKAHFPREGVFVSNGTHWFVTQRNFYEPQIITTDNTFVSGNCDVVIGIVNNTVYDP
MZ477746/P.1/Gamma	ICHDGKAHFPREGVFVSNGTHWFVTQRNFYEPQIITTDNTFVSGNCDVVIGIVNNTVYDP
MW938104/P.1/Gamma	ICHDGKAHFPREGVFVSNGTHWFVTQRNFYEPQIITTDNTFVSGNCDVVIGIVNNTVYDP
MZ896179/P.1/Gamma	ICHDGKAHFPREGVFVSNGTHWFVTQRNFYEPQIITTDNTFVSGNCDVVIGIVNNTVYDP
MZ896359/P.1/Gamma	ICHDGKAHFPREGVFVSNGTHWFVTQRNFYEPQIITTDNTFVSGNCDVVIGIVNNTVYDP
MZ896669/P.1/Gamma	ICHDGKAHFPREGVFVSNGTHWFVTQRNFYEPQIITTDNTFVSGNCDVVIGIVNNTVYDP
OU565182/P.1/Gamma	ICHDGKAHFPREGVFVSNGTHWFVTQRNFYEPQIITTDNTFVSGNCDVVIGIVNNTVYDP
OU492255/P.1/Gamma	ICHDGKAHFPREGVFVSNGTHWFVTQRNFYEPQIITTDNTFVSGNCDVVIGIVNNTVYDP
MZ911221/B.1.427/Epsilon	ICHDGKAHFPREGVFVSNGTHWFVTQRNFYEPQIITTDNTFVSGNCDVVIGIVNNTVYDP
MZ375854/B.1.427/Epsilon	ICHDGKAHFPREGVFVSNGTHWFVTQRNFYEPQIITTDNTFVSGNCDVVIGIVNNTVYDP
MZ375858/B.1.427/Epsilon	ICHDGKAHFPREGVFVSNGTHWFVTQRNFYEPQIITTDNTFVSGNCDVVIGIVNNTVYDP
MZ943834/B.1.429/Epsilon	ICHDGKAHFPREGVFVSNGTHWFVTQRNFYEPQIITTDNTFVSGNCDVVIGIVNNTVYDP
MZ277390/B.1.429/Epsilon	ICHDGKAHFPREGVFVSNGTHWFVTQRNFYEPQIITTDNTFVSGNCDVVIGIVNNTVYDP
OU282717/B.1.429/Epsilon	ICHDGKAHFPREGVFVSNGTHWFVTQRNFYEPQIITTDNTFVSGNCDVVIGIVNNTVYDP
OU235436/B.1.429/Epsilon	ICHDGKAHFPREGVFVSNGTHWFVTQRNFYEPQIITTDNTFVSGNCDVVIGIVNNTVYDP
MZ687447/B.1.429/Epsilon	ICHDGKAHFPREGVFVSNGTHWFVTQRNFYEPQIITTDNTFVSGNCDVVIGIVNNTVYDP
MZ538882/B.1.617.1/Kappa	ICHDGKAHFPREGVFVSNGTHWFVTQRNFYEPQIITTDNTFVSGNCDVVIGIVNNTVYDP
MZ724425/B.1.617.1/Kappa	ICHDGKAHFPREGVFVSNGTHWFVTQRNFYEPQIITTDNTFVSGNCDVVIGIVNNTVYDP
MZ363839/B.1.617.1/Kappa	ICHDGKAHFPREGVFVSNGTHWFVTQRNFYEPQIITTDNTFVSGNCDVVIGIVNNTVYDP
MZ332524/B.1.617.1/Kappa	ICHDGKAHFPREGVFVSNGTHWFVTQRNFYEPQIITTDNTFVSGNCDVVIGIVNNTVYDP
MZ332527/B.1.617.1/Kappa	ICHDGKAHFPREGVFVSNGTHWFVTQRNFYEPQIITTDNTFVSGNCDVVIGIVNNTVYDP
LC633761/B.1.617.1/Kappa	ICHDGKAHFPREGVFVSNGTHWFVTQRNFYEPQIITTDNTFVSGNCDVVIGIVNNTVYDP
MZ562746/B.1.617.1/Kappa	ICHDGKAHFPREGVFVSNGTHWFVTQRNFYEPQIITTDNTFVSGNCDVVIGIVNNTVYDP
MZ571142/B.1.617.1/Kappa	ICHDGKAHFPREGVFVSNGTHWFVTQRNFYEPQIITTDNTFVSGNCDVVIGIVNNTVYDP
OU168413/B.1.617.1/Kappa	ICHDGKAHFPREGVFVSNGTHWFVTQRNFYEPQIITTDNTFVSGNCDVVIGIVNNTVYDP
MZ026886/B.1.617.1/Kappa	ICHDGKAHFPREGVFVSNGTHWFVTQRNFYEPQIITTDNTFVSGNCDVVIGIVNNTVYDP
OU172184/B.1.617.1/Kappa	ICHDGKAHFPREGVFVSNGTHWFVTQRNFYEPQIITTDNTFVSGNCDVVIGIVNNTVYDP
MZ397173/B.1.617.1/Kappa	ICHDGKAHFPREGVFVSNGTHWFVTQRNFYEPQIITTDNTFVSGNCDVVIGIVNNTVYDP
MZ397176/B.1.617.1/Kappa	ICHDGKAHFPREGVFVSNGTHWFVTQRNFYEPQIITTDNTFVSGNCDVVIGIVNNTVYDP
MZ397175/B.1.617.1/Kappa	ICHDGKAHFPREGVFVSNGTHWFVTQRNFYEPQIITTDNTFVSGNCDVVIGIVNNTVYDP
MZ397177/B.1.617.1/Kappa	ICHDGKAHFPREGVFVSNGTHWFVTQRNFYEPQIITTDNTFVSGNCDVVIGIVNNTVYDP
OU290035/B.1.617.1/Kappa	ICHDGKAHFPREGVFVSNGTDWFVTQRNFYEPQIITTDNTFVSGNCDVVIGIVNNTVYDP
OU265178/B.1.617.1/Kappa	ICHDGKAHFPREGVFVSNGTDWFVTQRNFYEPQIITTDNTFVSGNCDVVIGIVNNTVYDP
OU254949/B.1.617.1/Kappa	ICHDGKAHFPREGVFVSNGTDWFVTQRNFYEPQIITTDNTFVSGNCDVVIGIVNNTVYDP
OU247018/B.1.617.1/Kappa	ICHDGKAHFPREGVFVSNGTDWFVTQRNFYEPQIITTDNTFVSGNCDVVIGIVNNTVYDP
OU061269/B.1.617.1/Kappa	ICHDGKAHFPREGVFVSNGTDWFVTQRNFYEPQIITTDNTFVSGNCDVVIGIVNNTVYDP
OU182966/B.1.617.1/Kappa	ICHDGKAHFPREGVFVSNGTDWFVTQRNFYEPQIITTDNTFVSGNCDVVIGIVNNTVYDP
MZ746235/B.1.617.1/Kappa	ICHDGKAHFPREGVFVSNGTHWFVTQRNFYEPQIITTDNTFVSGNCDVVIGIVNNTVYDP
MZ487800/B.1.617.1/Kappa	ICHDGKAHFPREGVFVSNGTDWFVTQRNFYEPQIITTDNTFVSGNCDVVIGIVNNTVYDP
OU322235/B.1.617.1/Kappa	ICHDGKAHFPREGVFVSNGTDWFVTQRNFYEPQIITTDNTFVSGNCDVVIGIVNNTVYDP
MZ953185/C.37/Lambda	ICHDGKAHFPREGVFVSNGTHWFVTQRNFYEPQIITTDNTFVSGNCDVVIGIVNNTVYDP
MZ937666/C.37/Lambda	ICHDGKAHFPREGVFVSNGTHWFVTQRNFYEPQIITTDNTFVSGNCDVVIGIVNNTVYDP
OU356036/C.37/Lambda	ICHDGKAHFPREGVFVSNGTHWFVTQRNFYEPQIITTDNTFVSGNCDVVIGIVNNTVYDP
OU351967/C.37/Lambda	ICHDGKAHFPREGVFVSNGTHWFVTQRNFYEPQIITTDNTFVSGNCDVVIGIVNNTVYDP
OU053018/C.37/Lambda	ICHDGKAHFPREGVFVSNGTHWFVTQRNFYEPQIITTDNTFVSGNCDVVIGIVNNTVYDP
OU268528/C.37/Lambda	ICHDGKAHFPREGVFVSNGTHWFVTQRNFYEPQIITTDNTFVSGNCDVVIGIVNNTVYDP
OU100999/C.37/Lambda	ICHDGKAHFPREGVFVSNGTHWFVTQRNFYEPQIITTDNTFVSGNCDVVIGIVNNTVYDP
MZ275295/C.37/Lambda	ICHDGKAHFPREGVFVSNGTHWFVTQRNFYEPQIITTDNTFVSGNCDVVIGIVNNTVYDP
MZ275301/C.37/Lambda	ICHDGKAHFPREGVFVSNGTHWFVTQRNFYEPQIITTDNTFVSGNCDVVIGIVNNTVYDP
MZ908711/C.37/Lambda	ICHDGKAHFPREGVFVSNGTHWFVTQRNFYEPQIITTDNTFVSGNCDVVIGIVNNTVYDP
MZ496613/C.37/Lambda	ICHDGKAHFPREGVFVSNGTHWFVTQRNFYEPQIITTDNTFVSGNCDVVIGIVNNTVYDP
OU517038/C.37/Lambda	ICHDGKAHFPREGVFVSNGTHWFVTQRNFYEPQIITTDNTFVSGNCDVVIGIVNNTVYDP
MZ953277/C.37/Lambda	ICHDGKAHFPREGVFVSNGTHWFVTQRNFYEPQIITTDNTFVSGNCDVVIGIVNNTVYDP
MZ942542/C.37/Lambda	ICHDGKAHFPREGVFVSNGTHWFVTQRNFYEPQIITTDNTFVSGNCDVVIGIVNNTVYDP
MZ857990/C.37/Lambda	ICHDGKAHFPREGVFVSNGTHWFVTQRNFYEPQIITTDNTFVSGNCDVVIGIVNNTVYDP

```
                                   1150      1160      1170      1180      1190      1200
                                      .         .         .         .         .         .
MN908947/Wuhan-Hu-1          LQPELDSFKEELDKYFKNHTSPDVDLGDISGINASVVNIQKEIDRLNEVAKNLNESLIDL
BS001137/B.1.1.7/Alpha       LQPELDSFKEELDKYFKNHTSPDVDLGDISGINASVVNIQKEIDRLNEVAKNLNESLIDL
BS001138/B.1.1.7/Alpha       LQPELDSFKEELDKYFKNHTSPDVDLGDISGINASVVNIQKEIDRLNEVAKNLNESLIDL
BS001139/B.1.1.7/Alpha       LQPELDSFKEELDKYFKNHTSPDVDLGDISGINASVVNIQKEIDRLNEVAKNLNESLIDL
BS001140/B.1.1.7/Alpha       LQPELDSFKEELDKYFKNHTSPDVDLGDISGINASVVNIQKEIDRLNEVAKNLNESLIDL
MW913362/B.1.1.7/Alpha       LQPELDSFKEELDKYFKNHTSPDVDLGDISGINASVVNIQKEIDRLNEVAKNLNESLIDL
MZ266365/B.1.1.7/Alpha       LQPELDSFKEELDKYFKNHTSPDVDLGDISGINASVVNIQKEIDRLNEVAKNLNESLIDL
MZ350105/B.1.1.7/Alpha       LQPELDSFKEELDKYFKNHTSPDVDLGDISGINASVVNIQKEIDRLNEVAKNLNESLIDL
MZ350109/B.1.1.7/Alpha       LQPELDSFKEELDKYFKNHTSPDVDLGDISGINASVVNIQKEIDRLNEVAKNLNESLIDL
MZ888516/B.1.1.7/Alpha       LQPELDSFKEELDKYFKNHTSPDVDLGDISGINASVVNIQKEIDRLNEVAKNLNESLIDL
OU562607/B.1.1.7/Alpha       LQPELDSFKEELDKYFKNHTSPDVDLGDISGINASVVNIQKEIDRLNEVAKNLNESLIDL
OU514166/B.1.1.7/Alpha       LQPELDSFKEELDKYFKNHTSPDVDLGDISGINASVVNIQKEIDRLNEVAKNLNESLIDL
OU538851/B.1.1.7/Alpha       LQPELDSFKEELDKYFKNHTSPDVDLGDISGINASVVNIQKEIDRLNEVAKNLNESLIDL
OU538849/B.1.1.7/Alpha       LQPELDSFKEELDKYFKNHTSPDVDLGDISGINASVVNIQKEIDRLNEVAKNLNESLIDL
MZ895877/B.1.1.7/Alpha       LQPELDSFKEELDKYFKNHTSPDVDLGDISGINASVVNIQKEIDRLNEVAKNLNESLIDL
OU514176/B.1.1.7/Alpha       LQPELDSFKEELDKYFKNHTSPDVDLGDISGINASVVNIQKEIDRLNEVAKNLNESLIDL
OU562608/B.1.1.7/Alpha       LQPELDSFKEELDKYFKNHTSPDVDLGDISGINASVVNIQKEIDRLNEVAKNLNESLIDL
MW981442/B.1.351/Beta        LQPELDSFKEELDKYFKNHTSPDVDLGDISGINASVVNIQKEIDRLNEVAKNLNESLIDL
MZ298839/B.1.351/Beta        LQPELDSFKEELDKYFKNHTSPDVDLGDISGINASVVNIQKEIDRLNEVAKNLNESLIDL
MZ913100/B.1.351/Beta        LQPELDSFKEELDKYFKNHTSPDVDLGDISGINASVVNIQKEIDRLNEVAKNLNESLIDL
MZ879365/B.1.351/Beta        LQPELDSFKEELDKYFKNHTSPDVDLGDISGINASVVNIQKEIDRLNEVAKNLNESLIDL
OU525106/B.1.351/Beta        LQPELDSFKEELDKYFKNHTSPDVDLGDISGINASVVNIQKEIDRLNEVAKNLNESLIDL
OU539352/B.1.351/Beta        LQPELDSFKEELDKYFKNHTSPDVDLGDISGINASVVNIQKEIDRLNEVAKNLNESLIDL
MZ202314/B.1.351/Beta        LQPELDSFKEELDKYFKNHTSPDVDLGDISGINASVVNIQKEIDRLNEVAKNLNESLIDL
MZ298840/B.1.351/Beta        LQPELDSFKEELDKYFKNHTSPDVDLGDISGINASVVNIQKEIDRLNEVAKNLNESLIDL
MZ376663/B.1.351/Beta        LQPELDSFKEELDKYFKNHTSPDVDLGDISGINASVVNIQKEIDRLNEVAKNLNESLIDL
LC643036/B.1.617.2/Delta     LQPELDSFKEELDKYFKNHTSPDVDLGDISGINASVVNIQKEIDRLNEVAKNLNESLIDL
LC643044/B.1.617.2/Delta     LQPELDSFKEELDKYFKNHTSPDVDLGDISGINASVVNIQKEIDRLNEVAKNLNESLIDL
MZ853946/B.1.617.2/Delta     WQPELDSFKEELDKYFKNHTSPDVDLGDISGINASVVNIQKEIDRLNEVAKNLNESLIDL
MZ854386/B.1.617.2/Delta     LQPELDSFKEELDKYFKNHTSPDVDLGDISGINASVVNIQKEIDRLNEVAKNLNESLIDL
MZ888532/B.1.617.2/Delta     LQPELDSFKEELDKYFKNHTSPDVDLGDISGINASVVNIQKEIDRLNEVAKNLNESLIDL
MZ888533/B.1.617.2/Delta     LQPELDSFKEELDKYFKNHTSPDVDLGDISGINASVVNIQKEIDRLNEVAKNLNESLIDL
MZ896136/B.1.617.2/Delta     LQPELDSFKEELDKYFKNHTSPDVDLGDISGINASVVNIQKEIDRLNEVAKNLNESLIDL
OU539261/B.1.617.2/Delta     LQPELDSFKEELDKYFKNHTSPDVDLGDISGINASVVNIQKEIDRLNEVAKNLNESLIDL
MZ854389/B.1.617.2/Delta     LQPELDSFKEELDKYFKNHTSPDVDLGDISGINASVVNIQKEIDRLNEVAKNLNESLIDL
OU562629/B.1.617.2/Delta     LQPELDSFKEELDKYFKNHTSPDVDLGDISGINASVVNIQKEIDRLNEVAKNLNESLIDL
OU539690/B.1.617.2/Delta     LQPELDSFKEELDKYFKNHTSPDVDLGDISGINASVVNIQKEIDRLNEVAKNLNESLIDL
MZ854387/B.1.617.2/Delta     LQPELDSFKEELDKYFKNHTSPDVDLGDISGINASVVNIQKEIDRLNEVAKNLNESLIDL
OU539784/P.1/Gamma           LQPELDSFKEELDKYFKNHTSPDVDLGDISGINASFVNIQKEIDRLNEVAKNLNESLIDL
OU539830/P.1/Gamma           LQPELDSFKEELDKYFKNHTSPDVDLGDISGINASFVNIQKEIDRLNEVAKNLNESLIDL
OU539894/P.1/Gamma           LQPELDSFKEELDKYFKNHTSPDVDLGDISGINASFVNIQKEIDRLNEVAKNLNESLIDL
MZ477748/P.1/Gamma           LQPELDSFKEELDKYFKNHTSPDVDLGDISGINASFVNIQKEIDRLNEVAKNLNESLIDL
MZ477746/P.1/Gamma           LQPELDSFKEELDKYFKNHTSPDVDLGDISGINASFVNIQKEIDRLNEVAKNLNESLIDL
MW938104/P.1/Gamma           LQPELDSFKEELDKYFKNHTSPDVDLGDISGINASFVNIQKEIDRLNEVAKNLNESLIDL
MZ896179/P.1/Gamma           LQPELDSFKEELDKYFKNHTSPDVDLGDISGINASFVNIQKEIDRLNEVAKNLNESLIDL
MZ896359/P.1/Gamma           LQPELDSFKEELDKYFKNHTSPDVDLGDISGINASFVNIQKEIDRLNEVAKNLNESLIDL
MZ896669/P.1/Gamma           LQPELDSFKEELDKYFKNHTSPDVDLGDISGINASFVNIQKEIDRLNEVAKNLNESLIDL
OU565182/P.1/Gamma           LQPELDSFKEELDKYFKNHTSPDVDLGDISGINASFVNIQKEIDRLNEVAKNLNESLIDL
OU492255/P.1/Gamma           LQPELDSFKEELDKYFKNHTSPDVDLGDISGINASFVNIQKEIDRLNEVAKNLNESLIDL
MZ911221/B.1.427/Epsilon     LQPELDSFKEELDKYFKNHTSPDVDLGDISGINASVVNIQKEIDRLNEVAKNLNESLIDL
MZ375854/B.1.427/Epsilon     LQPELDSFKEELDKYFKNHTSPDVDLGDISGINASVVNIQKEIDRLNEVAKNLNESLIDL
MZ375858/B.1.427/Epsilon     LQPELDSFKEELDKYFKNHTSPDVDLGDISGINASVVNIQKEIDRLNEVAKNLNESLIDL
MZ943834/B.1.429/Epsilon     LQPELDSFKEELDKYFKNHTSPDVDLGDISGINASVVNIQKEIDRLNEVAKNLNESLIDL
MZ277390/B.1.429/Epsilon     LQPELDSFKEELDKYFKNHTSPDVDLGDISGINASVVNIQKEIDRLNEVAKNLNESLIDL
OU282717/B.1.429/Epsilon     LQPELDSFKEELDKYFKNHTSPDVDLGDISGINASVVNIQKEIDRLNEVAKNLNESLIDL
OU235436/B.1.429/Epsilon     LQPELDSFKEELDKYFKNHTSPDVDLGDISGINASVVNIQKEIDRLNEVAKNLNESLIDL
MZ687447/B.1.429/Epsilon     LQPELDSFKEELDKYFKNHTSPDVDLGDISGINASVVNIQKEIDRLNEVAKNLNESLIDL
MZ538882/B.1.617.1/Kappa     LQPELDSFKEELDKYFKNHTSPDVDLGDISGINASVVNIQKEIDRLNEVAKNLNESLIDL
MZ724425/B.1.617.1/Kappa     LQPELDSFKEELDKYFKNHTSPDVDLGDISGINASVVNIQKEIDRLNEVAKNLNESLIDL
MZ363839/B.1.617.1/Kappa     LQPELDSFKEELDKYFKNHTSPDVDLGDISGINASVVNIQKEIDRLNEVAKNLNESLIDL
MZ332524/B.1.617.1/Kappa     LQPELDSFKEELDKYFKNHTSPDVDLGDISGINASVVNIQKEIDRLNEVAKNLNESLIDL
MZ332527/B.1.617.1/Kappa     LQPELDSFKEELDKYFKNHTSPDVDLGDISGINASVVNIQKEIDRLNEVAKNLNESLIDL
LC633761/B.1.617.1/Kappa     LQPELDSFKEELDKYFKNHTSPDVDLGDISGINASVVNIQKEIDRLNEVAKNLNESLIDL
MZ562746/B.1.617.1/Kappa     LQPELDSFKEELDKYFKNHTSPDVDLGDISGINASVVNIQKEIDRLNEVAKNLNESLIDL
MZ571142/B.1.617.1/Kappa     LQPELDSFKEELDKYFKNHTSPDVDLGDISGINASVVNIQKEIDRLNEVAKNLNESLIDL
OU168413/B.1.617.1/Kappa     LQPELDSFKEELDKYFKNHTSPDVDLGDISGINASVVNIQKEIDRLNEVAKNLNESLIDL
MZ026886/B.1.617.1/Kappa     LQPELDSFKEELDKYFKNHTSPDVDLGDISGINASVVNIQKEIDRLNEVAKNLNESLIDL
OU172184/B.1.617.1/Kappa     LQPELDSFKEELDKYFKNHTSPDVDLGDISGINASVVNIQKEIDRLNEVAKNLNESLIDL
MZ397173/B.1.617.1/Kappa     LQPELDSFKEELDKYFKNHTSPDVDLGDISGINASVVNIQKEIDRLNEVAKNLNESLIDL
MZ397176/B.1.617.1/Kappa     LQPELDSFKEELDKYFKNHTSPDVDLGDISGINASVVNIQKEIDRLNEVAKNLNESLIDL
MZ397175/B.1.617.1/Kappa     LQPELDSFKEELDKYFKNHTSPDVDLGDISGINASVVNIQKEIDRLNEVAKNLNESLIDL
MZ397177/B.1.617.1/Kappa     LQPELDSFKEELDKYFKNHTSPDVDLGDISGINASVVNIQKEIDRLNEVAKNLNESLIDL
OU290035/B.1.617.1/Kappa     LQPELDSFKEELDKYFKNHTSPDVDLGDISGINASVVNIQKEIDRLNEVAKNLNESLIDL
OU265178/B.1.617.1/Kappa     LQPELDSFKEELDKYFKNHTSPDVDLGDISGINASVVNIQKEIDRLNEVAKNLNESLIDL
OU254949/B.1.617.1/Kappa     LQPELDSFKEELDKYFKNHTSPDVDLGDISGINASVVNIQKEIDRLNEVAKNLNESLIDL
OU247018/B.1.617.1/Kappa     LQPELDSFKEELDKYFKNHTSPDVDLGDISGINASVVNIQKEIDRLNEVAKNLNESLIDL
OU061269/B.1.617.1/Kappa     LQPELDSFKEELDKYFKNHTSPDVDLGDISGINASVVNIQKEIDRLNEVAKNLNESLIDL
OU182966/B.1.617.1/Kappa     LQPELDSFKEELDKYFKNHTSPDVDLGDISGINASVVNIQKEIDRLNEVAKNLNESLIDL
MZ746235/B.1.617.1/Kappa     LQPELDSFKEELDKYFKNHTSPDVDLGDISGINASVVNIQKEIDRLNEVAKNLNESLIDL
MZ487800/B.1.617.1/Kappa     LQPELDSFKEELDKYFKNHTSPDVDLGDISGINASVVNIQKEIDRLNEVAKNLNESLIDL
OU322235/B.1.617.1/Kappa     LQPELDSFKEELDKYFKNHTSPDVDLGDISGINASVVNIQKEIDRLNEVAKNLNESLIDL
MZ953185/C.37/Lambda         LQPELDSFKEELDKYFKNHTSPDVDLGDISGINASVVNIQKEIDRLNEVAKNLNESLIDL
MZ937666/C.37/Lambda         LQPELDSFKEELDKYFKNHTSPDVDLGDISGINASVVNIQKEIDRLNEVAKNLNESLIDL
OU356036/C.37/Lambda         LQPELDSFKEELDKYFKNHTSPDVDLGDISGINASVVNIQKEIDRLNEVAKNLNESLIDL
OU351967/C.37/Lambda         LQPELDSFKEELDKYFKNHTSPDVDLGDISGINASVVNIQKEIDRLNEVAKNLNESLIDL
OU053018/C.37/Lambda         LQPELDSFKEELDKYFKNHTSPDVDLGDISGINASVVNIQKEIDRLNEVAKNLNESLIDL
OU268528/C.37/Lambda         LQPELDSFKEELDKYFKNHTSPDVDLGDISGINASVVNIQKEIDRLNEVAKNLNESLIDL
OU100999/C.37/Lambda         LQPELDSFKEELDKYFKNHTSPDVDLGDISGINASVVNIQKEIDRLNEVAKNLNESLIDL
MZ275295/C.37/Lambda         LQPELDSFKEELDKYFKNHTSPDVDLGDISGINASVVNIQKEIDRLNEVAKNLNESLIDL
MZ275301/C.37/Lambda         LQPELDSFKEELDKYFKNHTSPDVDLGDISGINASVVNIQKEIDRLNEVAKNLNESLIDL
MZ908711/C.37/Lambda         LQPELDSFKEELDKYFKNHTSPDVDLGDISGINASVVNIQKEIDRLNEVAKNLNESLIDL
MZ496613/C.37/Lambda         LQPELDSFKEELDKYFKNHTSPDVDLGDISGINASVVNIQKEIDRLNEVAKNLNESLIDL
OU517038/C.37/Lambda         LQPELDSFKEELDKYFKNHTSPDVDLGDISGINASVVNIQKEIDRLNEVAKNLNESLIDL
MZ953277/C.37/Lambda         LQPELDSFKEELDKYFKNHTSPDVDLGDFSGINASVVNIQKEIDRLNEVAKNLNESLIDL
MZ942542/C.37/Lambda         LQPELDSFKEELDKYFKNHTSPDVDLGDISGINASVVNIQKEIDRLNEVAKNLNESLIDL
MZ857990/C.37/Lambda         LQPELDSFKEELDKYFKNHTSPDVDLGDISGINASVVNIQKEIDRLNEVAKNLNESLIDL
```

```
                                   1210      1220      1230      1240      1250      1260
MN908947/Wuhan-Hu-1          QELGKYEQYIKWPWYIWLGFIAGLIAIVMVTIMLCCMTSCCSCLKGCCSCGSCCKFDEDD
BS001137/B.1.1.7/Alpha       QELGKYEQYIKWPWYIWLGFIAGLIAIVMVTIMLCCMTSCCSCLKGCCSCGSCCKFDEDD
BS001138/B.1.1.7/Alpha       QELGKYEQYIKWPWYIWLGFIAGLIAIVMVTIMLCCMTSCCSCLKGCCSCGSCCKFDEDD
BS001139/B.1.1.7/Alpha       QELGKYEQYIKWPWYIWLGFIAGLIAIVMVTIMLCCMTSCCSCLKGCCSCGSCCKFDEDD
BS001140/B.1.1.7/Alpha       QELGKYEQYIKWPWYIWLGFIAGLIAIVMVTIMLCCMTSCCSCLKGCCSCGSCCKFDEDD
MW913362/B.1.1.7/Alpha       QELGKYEQYIKWPWYIWLGFIAGLIAIVMVTIMLCCMTSCCSCLKGCCSCGSCCKFDEDD
MZ266365/B.1.1.7/Alpha       QELGKYEQYIKWPWYIWLGFIAGLIAIVMVTIMLCCMTSCCSCLKGCCSCGSCCKFDEDD
MZ350105/B.1.1.7/Alpha       QELGKYEQYIKWPWYIWLGFIAGLIAIVIVTIMLCCMTSCCSCLKGCCSCGSCCKFDEDD
MZ350109/B.1.1.7/Alpha       QELGKYEQYIKWPWYIWLGFIAGLIAIVMVTIMLCCMTSCCSCLKGCCSCGSCCKFDEDD
MZ888516/B.1.1.7/Alpha       QELGKYEQYIKWPWYIWLGFIAGLIAIVMVTIMLCCMTSCCSCLKGCCSCGSCCKFDEDD
OU562607/B.1.1.7/Alpha       QELGKYEQYIKWPWYIWLGFIAGLIAIVMVTIMLCCMTSCCSCLKGCCSCGSCCKFDEDD
OU514166/B.1.1.7/Alpha       QELGKYEQYIKWPWYIWLGFIAGLIAIVMVTIMLCCMTSCCSCLKGCCSCGSCCKFDEDD
OU538851/B.1.1.7/Alpha       QELGKYEQYIKWPWYIWLGFIAGLIAIVMVTIMLCCMTSCCSCLKGCCSCGSCCKFDEDD
OU538849/B.1.1.7/Alpha       QELGKYEQYIKWPWYIWLGFIAGLIAIVMVTIMLCCMTSCCSCLKGCCSCGSCCKFDEDD
MZ895877/B.1.1.7/Alpha       QELGKYEQYIKWPWYIWLGFIAGLIAIVMVTIMLCCMTSCCSCLKGCCSCGSCCKFDEDD
OU514176/B.1.1.7/Alpha       QELGKYEQYIKWPWYIWLGFIAGLIAIVMVTIMLCCMTSCCSCLKGCCSCGSCCKFDEDD
OU562608/B.1.1.7/Alpha       QELGKYEQYIKWPWYIWLGFIAGLIAIVMVTIMLCCMTSCCSCLKGCCSCGSCCKFDEDD
MW981442/B.1.351/Beta        QELGKYEQYIKWPWYIWLGFIAGLIAIVMVTIMLCCMTSCCSCLKGCCSCGSCCKFDEDD
MZ298839/B.1.351/Beta        QELGKYEQYIKWPWYIWLGFIAGLIAIVMVTIMLCCMTSCCSCLKGCCSCGSCCKFDEDD
MZ913100/B.1.351/Beta        QELGKYEQYIKWPWYIWLGFIAGLIAIVMVTIMLCCMTSCCSCLKGCCSCGSCCKFDEDD
MZ879365/B.1.351/Beta        QELGKYEQYIKWPWYIWLGFIAGLIAIVMVTIMLCCMTSCCSCLKGCCSCGSCCKFDEDD
OU525106/B.1.351/Beta        QELGKYEQYIKWPWYIWLGFIAGLIAIVMVTIMLCCMTSCCSCLKGCCSCGSCCKFDEDD
OU539352/B.1.351/Beta        QELGKYEQYIKWPWYIWLGFIAGLIAIVIVTIMLCCMTSCCSCLKGCCSCGSCCKFDEDD
MZ202314/B.1.351/Beta        QELGKYEQYIKWPWYIWLGFIAGLIAIVMVTIMLCCMTSCCSCLKGCCSCGSCCKFDEDD
MZ298840/B.1.351/Beta        QELGKYEQYIKWPWYIWLGFIAGLIAIVMVTIMLCCMTSCCSCLKGCCSCGSCCKFDEDD
MZ376663/B.1.351/Beta        QELGKYEQYIKWPWYIWLGFIAGLIAIVMVTIMLCCMTSCCSCLKGCCSCGSCCKFDEDD
LC643036/B.1.617.2/Delta     QELGKYEQYIKWPWYIWLGFIAGLIAIVMVTIMLCCMTSCCSCLKGCCSCGSCCKFDEDD
LC643044/B.1.617.2/Delta     QELGKYEQYIKWPWYIWLGFIAGLIAIVMVTIMLCCMTSCCSCLKGCCSCGSCCKFDEDD
MZ853946/B.1.617.2/Delta     QELGKYEQYIKWPWYIWLGFIAGLIAIVMVTIMLCCMTSCCSCLKGCCSCGSCCKFDEDD
MZ854386/B.1.617.2/Delta     QELGKYEQYIKWPWYIWLGFIAGLIAIVMVTIMLCCMTSCCSCLKGCCSCGSCCKFDEDD
MZ888532/B.1.617.2/Delta     QELGKYEQYIKWPWYIWLGFIAGLIAIVMVTIMLCCMTSCCSCLKGCCSCGSCCKFDEDD
MZ888533/B.1.617.2/Delta     QELGKYEQYIKWPWYIWLGFIAGLIAIVMVTIMLCCMTSCCSCLKGCCSCGSCCKFDEDD
MZ896136/B.1.617.2/Delta     QELGKYEQYIKWPWYIWLGFIAGLIAIVMVTIMLCCMTSCCSCLKGCCSCGSCCKFDEDD
OU539261/B.1.617.2/Delta     QELGKYEQYIKWPWYIWLGFIAGLIAIVMVTIMLCCMTSCCSCLKGCCSCGSCCKFDEDD
MZ854389/B.1.617.2/Delta     QELGKYEQYIKWPWYIWLGFIAGLIAIVMVTIMLCCMTSCCSCLKGCCSCGSCCKFDEDD
OU562629/B.1.617.2/Delta     QELGKYEQYIKWPWYIWLGFIAGLIAIVMVTIMLCCMTSCCSCLKGCCSCGSCCKFDEDD
OU539690/B.1.617.2/Delta     QELGKYEQYIKWPWYIWLGFIAGLIAIVMVTIMLCCMTSCCSCLKGCCSCGSCCKFDEDD
MZ854387/B.1.617.2/Delta     QELGKYEQYIKWPWYIWLGFIAGLIAIVMVTIMLCCMTSCCSCLKGCCSCGSCCKFDEDD
OU539784/P.1/Gamma           QELGKYEQYIKWPWYIWLGFIAGLIAIVMVTIMLCCMTSCCSCLKGCCSCGSCCKFDEDD
OU539830/P.1/Gamma           QELGKYEQYIKWPWYIWLGFIAGLIAIVMVTIMLCCMTSCCSCLKGCCSCGSCCKFDEDD
OU539894/P.1/Gamma           QELGKYEQYIKWPWYIWLGFIAGLIAIVMVTIMLCCMTSCCSCLKGCCSCGSCCKFDEDD
MZ477748/P.1/Gamma           QELGKYEQYIKWPWYIWLGFIAGLIAIVMVTIMLCCMTSCCSCLKGCCSCGSCCKFDEDD
MZ477746/P.1/Gamma           QELGKYEQYIKWPWYIWLGFIAGLIAIVMVTIMLCCMTSCCSCLKGCCSCGSCCKFDEDD
MW938104/P.1/Gamma           QELGKYEQYIKWPWYIWLGFIAGLIAIVMVTIMLCCMTSCCSCLKGCCSCGSCCKFDEDD
MZ896179/P.1/Gamma           QELGKYEQYIKWPWYIWLGFIAGLIAIVMVTIMLCCMTSCCSCLKGCCSCGSCCKFDEDD
MZ896359/P.1/Gamma           QELGKYEQYIKWPWYIWLGFIAGLIAIVMVTIMLCCMTSCCSCLKGCCSCGSCCKFDEDD
MZ896669/P.1/Gamma           QELGKYEQYIKWPWYIWLGFIAGLIAIVMVTIMLCCMTSCCSCLKGCCSCGSCCKFDEDD
OU565182/P.1/Gamma           QELGKYEQYIKWPWYIWLGFIAGLIAIVMVTIMLCCMTSCCSCLKGCCSCGSCCKFDEDD
OU492255/P.1/Gamma           QELGKYEQYIKWPWYIWLGFIAGLIAIVMVTIMLCCMTSCCSCLKGCCSCGSCCKFDEDD
MZ911221/B.1.427/Epsilon     QELGKYEQYIKWPWYIWLGFIAGLIAIVMVTIMLCCMTSCCSCLKGCCSCGSCCKFDEDD
MZ375854/B.1.427/Epsilon     QELGKYEQYIKWPWYIWLGFIAGLIAIVMVTIMLCCMTSCCSCLKGCCSCGSCCKFDEDD
MZ375858/B.1.427/Epsilon     QELGKYEQYIKWPWYIWLGFIAGLIAIVMVTIMLCCMTSCCSCLKGCCSCGSCCKFDEDD
MZ943834/B.1.429/Epsilon     QELGKYEQYIKWPWYIWLGFIAGLIAIVMVTIMLCCMTSCCSCLKGCCSCGSCCKFDEDD
MZ277390/B.1.429/Epsilon     QELGKYEQYIKWPWYIWLGFIAGLIAIVMVTIMLCCMTSCCSCLKGCCSCGSCCKFDEDD
OU282717/B.1.429/Epsilon     QELGKYEQYIKWPWYIWLGFIAGLIAIVMVTIMLCCMTSCCSCLKGCCSCGSCCKFDEDD
OU235436/B.1.429/Epsilon     QELGKYEQYIKWPWYIWLGFIAGLIAIVMVTIMLCCMTSCCSCLKGCCSCGSCCKFDEDD
MZ687447/B.1.429/Epsilon     QELGKYEQYIKWPWYIWLGFIAGLIAIVMVTIMLCCMTSCCSCLKGCCSCGSCCKFDEDD
MZ538882/B.1.617.1/Kappa     QELGKYEQYIKWPWYIWLGFIAGLIAIVMVTIMLCCMTSCCSCLKGCCSCGSCCKFDEDD
MZ724425/B.1.617.1/Kappa     QELGKYEQYIKWPWYIWLGFIAGLIAIVMVTIMLCCMTSCCSCLKGCCSCGSCCKFDEDD
MZ363839/B.1.617.1/Kappa     QELGKYEQYIKWPWYIWLGFIAGLIAIVMVTIMLCCMTSCCSCLKGCCSCGSCCKFDEDD
MZ332524/B.1.617.1/Kappa     QELGKYEQYIKWPWYIWLGFIAGLIAIVMVTIMLCCMTSCCSCLKGCCSCGSCCKFDEDD
MZ332527/B.1.617.1/Kappa     QELGKYEQYIKWPWYIWLGFIAGLIAIVMVTIMLCCMTSCCSCLKGCCSCGSCCKFDEDD
LC633761/B.1.617.1/Kappa     QELGKYEQYIKWPWYIWLGFIAGLIAIVMVTIMLCCMTSCCSCLKGCCSCGSCCKFDEDD
MZ562746/B.1.617.1/Kappa     QELGKYEQYIKWPWYIWLGFIAGLIAIVMVTIMLCCMTSCCSCLKGCCSCGSCCKFDEDD
MZ571142/B.1.617.1/Kappa     QELGKYEQYIKWPWYIWLGFIAGLIAIVMVTIMLCCMTSCCSCLKGCCSCGSCCKFDEDD
OU168413/B.1.617.1/Kappa     QELGKYEQYIKWPWYIWLGFIAGLIAIVMVTIMLCCMTSCCSCLKGCCSCGSCCKFDEDD
MZ026886/B.1.617.1/Kappa     QELGKYEQYIKWPWYIWLGFIAGLIAIVMVTIMLCCMTSCCSCLKGCCSCGSCCKFDEDD
OU172184/B.1.617.1/Kappa     QELGKYEQYIKWPWYIWLGFIAGLIAIVMVTIMLCCMTSCCSCLKGCCSCGSCCKFDEDD
MZ397173/B.1.617.1/Kappa     QELGKYEQYIKWPWYIWLGFIAGLIAIVMVTIMLCCMTSCCSCLKGCCSCGSCCKFDEDD
MZ397176/B.1.617.1/Kappa     QELGKYEQYIKWPWYIWLGFIAGLIAIVMVTIMLCCMTSCCSCLKGCCSCGSCCKFDEDD
MZ397175/B.1.617.1/Kappa     QELGKYEQYIKWPWYIWLGFIAGLIAIVMVTIMLCCMTSCCSCLKGCCSCGSCCKFDEDD
MZ397177/B.1.617.1/Kappa     QELGKYEQYIKWPWYIWLGFIAGLIAIVMVTIMLCCMTSCCSCLKGCCSCGSCCKFDEDD
OU290035/B.1.617.1/Kappa     QELGKYEQYIKWPWYIWLGFIAGLIAIVMVTIMLCCMTSCCSCLKGCCSCGSCCKFDEDD
OU265178/B.1.617.1/Kappa     QELGKYEQYIKWPWYIWLGFIAGLIAIVMVTIMLCCMTSCCSCLKGCCSCGSCCKFDEDD
OU254949/B.1.617.1/Kappa     QELGKYEQYIKWPWYIWLGFIAGLIAIVMVTIMLCCMTSCCSCLKGCCSCGSCCKFDEDD
OU247018/B.1.617.1/Kappa     QELGKYEQYIKWPWYIWLGFIAGLIAIVMVTIMLCCMTSCCSCLKGCCSCGSCCKFDEDD
OU061269/B.1.617.1/Kappa     QELGKYEQYIKWPWYIWLGFIAGLIAIVMVTIMLCCMTSCCSCLKGCCSCGSCCKFDEDD
OU182966/B.1.617.1/Kappa     QELGKYEQYIKWPWYIWLGFIAGLIAIVMVTIMLCCMTSCCSCLKGCCSCGSCCKFDEDD
MZ746235/B.1.617.1/Kappa     QELGKYEQYIKWPWYIWLGFIAGLIAIVMVTIMLCCMTSCCSCLKGCCSCGSCCKFDEDD
MZ487800/B.1.617.1/Kappa     QELGKYEQYIKWPWYIWLGFIAGLIAIVMVTIMLCCMTSCCSCLKGCCSCGSCCKFDEDD
OU322235/B.1.617.1/Kappa     QELGKYEQYIKWPWYIWLGFIAGLIAIVMVTIMLCCMTSCCSCLKGCCSCGSCCKFDEDD
MZ953185/C.37/Lambda         QELGKYEQYIKWPWYIWLGFIAGLIAIVMVTIMLCCMTSCCSCLKGCCSCGSCCKFDEDD
MZ937666/C.37/Lambda         QELGKYEQYIKWPWYIWLGFIAGLIAIVMVTIMLCCMTSCCSCLKGCCSCGSCCKFDEDD
OU356036/C.37/Lambda         QELGKYEQYIKWPWYIWLGFIAGLIAIVMVTIMLCCMTSCCSCLKGCCSCGSCCKFDEDD
OU351967/C.37/Lambda         QELGKYEQYIKWPWYIWLGFIAGLIAIVMVTIMLCCMTSCCSCLKGCCSCGSCCKFDEDD
OU053018/C.37/Lambda         QELGKYEQYIKWPWYIWLGFIAGLIAIVMVTIMLCCMTSCCSCLKGCCSCGSCCKFDEDD
OU268528/C.37/Lambda         QELGKYEQYIKWPWYIWLGFIAGLIAIVMVTIMLCCMTSCCSCLKGCCSCGSCCKFDEDD
OU100999/C.37/Lambda         QELGKYEQYIKWPWYIWLGFIAGLIAIVMVTIMLCCMTSCCSCLKGCCSCGSCCKFDEDD
MZ275295/C.37/Lambda         QELGKYEQYIKWPWYIWLGFIAGLIAIVMVTIMLCCMTSCCSCLKGCCSCGSCCKFDEDD
MZ275301/C.37/Lambda         QELGKYEQYIKWPWYIWLGFIAGLIAIVMVTIMLCCMTSCCSCLKGCCSCGSCCKFDEDD
MZ908711/C.37/Lambda         QELGKYEQYIKWPWYIWLGFIAGLIAIVMVTIMLCCMTSCCSCLKGCCSCGSCCKFDEDD
MZ496613/C.37/Lambda         QELGKYEQYIKWPWYIWLGFIAGLIAIVMVTIMLCCMTSCCSCLKGCCSCGSCCKFDEDD
OU517038/C.37/Lambda         QELGKYEQYIKWPWYIWLGFIAGLIAIVMVTIMLCCMTSCCSCLKGCCSCGSCCKFDEDD
MZ953277/C.37/Lambda         QELGKYEQYIKWPWYIWLGFIAGLIAIVMVTIMLCCMTSCCSCLKGCCSCGSCCKFDEDD
MZ942542/C.37/Lambda         QELGKYEQYIKWPWYIWLGFIAGLIAIVMVTIMLCCMTSCCSCLKGCCSCGSCCKFDEDD
MZ857990/C.37/Lambda         QELGKYEQYIKWPWYIWLGFIAGLIAIVMVTIMLCCMTSCCSCLKGCCSCGSCCKFDEDD
```

	1270
MN908947/Wuhan-Hu-1	SEPVLKGVKLHYT
BS001137/B.1.1.7/Alpha	SEPVLKGVKLHYT
BS001138/B.1.1.7/Alpha	SEPVLKGVKLHYT
BS001139/B.1.1.7/Alpha	SEPVLKGVKLHYT
BS001140/B.1.1.7/Alpha	SEPVLKGVKLHYT
MW913362/B.1.1.7/Alpha	SEPVLKGVKLHYT
MZ266365/B.1.1.7/Alpha	SEPVLKGVKLHYT
MZ350105/B.1.1.7/Alpha	SEPVLKGVKLHYT
MZ350109/B.1.1.7/Alpha	SEPVLKGVKLHYT
MZ888516/B.1.1.7/Alpha	SEPVLKGVKLHYT
OU562607/B.1.1.7/Alpha	SEPVLKGVKLHYT
OU514166/B.1.1.7/Alpha	SEPVLKGVKLHYT
OU538851/B.1.1.7/Alpha	SEPVLKGVKLHYT
OU538849/B.1.1.7/Alpha	SEPVLKGVKLHYT
MZ895877/B.1.1.7/Alpha	SEPVLKGVKLHYT
OU514176/B.1.1.7/Alpha	SEPVLKGVKLHYT
OU562608/B.1.1.7/Alpha	SEPVLKGVKLHYT
MW981442/B.1.351/Beta	SEPVLKGVKLHYT
MZ298839/B.1.351/Beta	SEPVLKGVKLHYT
MZ913100/B.1.351/Beta	SEPVLKGVKLHYT
MZ879365/B.1.351/Beta	SEPVLKGVKLHYT
OU525106/B.1.351/Beta	SEPVLKGVKLHYT
OU539352/B.1.351/Beta	SEPVLKGVKLHYT
MZ202314/B.1.351/Beta	SEPVLKGVKLHYT
MZ298840/B.1.351/Beta	SEPVLKGVKLHYT
MZ376663/B.1.351/Beta	SEPVLKGVKLHYT
LC643036/B.1.617.2/Delta	SEPVLKGVKLHYT
LC643044/B.1.617.2/Delta	SEPVLKGVKLHYT
MZ853946/B.1.617.2/Delta	SEPVLKGVKLHYT
MZ854386/B.1.617.2/Delta	SEPVLKGVKLHYT
MZ888532/B.1.617.2/Delta	SEPVLKGVKLHYT
MZ888533/B.1.617.2/Delta	SEPVLKGVKLHYT
MZ896136/B.1.617.2/Delta	SEPVLKGVKLHYT
OU539261/B.1.617.2/Delta	SEPVLKGVKLHYT
MZ854389/B.1.617.2/Delta	SEPVLKGVKLHYT
OU562629/B.1.617.2/Delta	SEPVLKGVKLHYT
OU539690/B.1.617.2/Delta	SEPVLKGVKLHYT
MZ854387/B.1.617.2/Delta	SEPVLKGVKLHYT
OU539784/P.1/Gamma	SEPVLKGVKLHYT
OU539830/P.1/Gamma	SEPVLKGVKLHYT
OU539894/P.1/Gamma	SEPVLKGVKLHYT
MZ477748/P.1/Gamma	SEPVLKGVKLHYT
MZ477746/P.1/Gamma	SEPVLKGVKLHYT
MW938104/P.1/Gamma	SEPVLKGVKLHYT
MZ896179/P.1/Gamma	SEPVLKGVKLHYT
MZ896359/P.1/Gamma	SEPVLKGVKLHYT
MZ896669/P.1/Gamma	SEPVLKGVKLHYT
OU565182/P.1/Gamma	SEPVLKGVKLHYT
OU492255/P.1/Gamma	SEPVLKGVKLHYT
MZ911221/B.1.427/Epsilon	SEPVLKGVKLHYT
MZ375854/B.1.427/Epsilon	SEPVLKGVKLHYT
MZ375858/B.1.427/Epsilon	SEPVLKGVKLHYT
MZ943834/B.1.429/Epsilon	SEPVLKGVKLHYT
MZ277390/B.1.429/Epsilon	SEPVLKGVKLHYT
OU282717/B.1.429/Epsilon	SEPVLKGVKLHYT
OU235436/B.1.429/Epsilon	SEPVLKGVKLHYT
MZ687447/B.1.429/Epsilon	SEPVLKGVKLHYT
MZ538882/B.1.617.1/Kappa	SEPVLKGVKLHYT
MZ724425/B.1.617.1/Kappa	SEPVLKGVKLHYT
MZ363839/B.1.617.1/Kappa	SEPVLKGVKLHYT
MZ332524/B.1.617.1/Kappa	SEPVLKGVKLHYT
MZ332527/B.1.617.1/Kappa	SEPVLKGVKLHYT
LC633761/B.1.617.1/Kappa	SEPVLKGVKLHYT
MZ562746/B.1.617.1/Kappa	SEPVLKGVKLHYT
MZ571142/B.1.617.1/Kappa	SEPVLKGVKLHYT
OU168413/B.1.617.1/Kappa	SEPVLKGVKLHYT
MZ026886/B.1.617.1/Kappa	SEPVLKGVKLHYT
OU172184/B.1.617.1/Kappa	SEPVLKGVKLHYT
MZ397173/B.1.617.1/Kappa	SEPVLKGVKLHYT
MZ397176/B.1.617.1/Kappa	SEPVLKGVKLHYT
MZ397175/B.1.617.1/Kappa	SEPVLKGVKLHYT
MZ397177/B.1.617.1/Kappa	SEPVLKGVKLHYT
OU290035/B.1.617.1/Kappa	SEPVLKGVKLHYT
OU265178/B.1.617.1/Kappa	SEPVLKGVKLHYT
OU254949/B.1.617.1/Kappa	SEPVLKGVKLHYT
OU247018/B.1.617.1/Kappa	SEPVLKGVKLHYT
OU061269/B.1.617.1/Kappa	SEPVLKGVKLHYT
OU182966/B.1.617.1/Kappa	SEPVLKGVKLHYT
MZ746235/B.1.617.1/Kappa	SEPVLKGVKLHYT
MZ487800/B.1.617.1/Kappa	SEPVLKGVKLHYT
OU322235/B.1.617.1/Kappa	SEPVLKGVKLHYT
MZ953185/C.37/Lambda	SEPVLKGVKLHYT
MZ937666/C.37/Lambda	SEPVLKGVKLHYT
OU356036/C.37/Lambda	SEPVLKGVKLHYT
OU351967/C.37/Lambda	SEPVLKGVKLHYT
OU053018/C.37/Lambda	SEPVLKGVKLHYT
OU268528/C.37/Lambda	SEPVLKGVKLHYT
OU100999/C.37/Lambda	SEPVLKGVKLHYT
MZ275295/C.37/Lambda	SEPVLKGVKLHYT
MZ275301/C.37/Lambda	SEPVLKGVKLHYT
MZ908711/C.37/Lambda	SEPVLKGVKLHYT
MZ496613/C.37/Lambda	SEPVLKGVKLHYT
OU517038/C.37/Lambda	SEPVLKGVKLHYT
MZ953277/C.37/Lambda	SEPVLKGVKLHYT
MZ942542/C.37/Lambda	SEPVLKGVKLHYT
MZ857990/C.37/Lambda	SEPVLKGVKLHYT

Supplementary Figure S1. Multiple alignment of amino acid sequences of spike protein from different variants of concern or variants of interest from different regions, and compared with wild-type SARS-CoV-2 strain isolated from Wuhan, China (accession number MN908947). SARS-CoV-2 genomes used for alignment were download from NCBI GenBank database and its detailed information was listed in Supplementary Table S1. Multiple alignment was performed and visualized using MAFFT version 7 and ESPript 3.0 respectively. The accession number, pangolin lineage and WHO classification are shown on the left side of the sequences. The red boxes represent identical residues while similar residues are indicated by red letters.

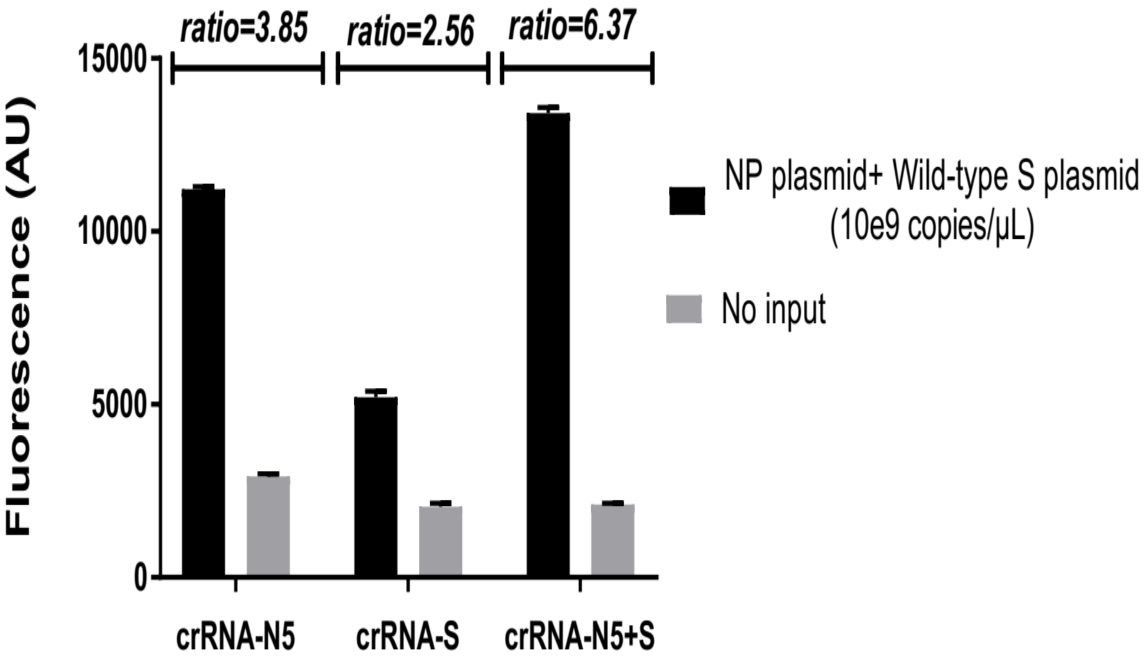

Supplementary Figure S2. Enhanced detection sensitivity for the combination of different guide RNAs in CRISPR-Cas12a-based detection. 10^9 copies/μL of plasmid DNA for SARS-CoV-2 nucleoprotein (NP) and wild-type spike (S) gene were detected by CRISPR-Cas12a-based assay using crRNA-N5, crRNA-S and the mixture of crRNA-N5 and crRNA-S, respectively. The ration of fluorescence signal for SARS-CoV-2 plasmid DNA over no input control was presented at the top of the figure.

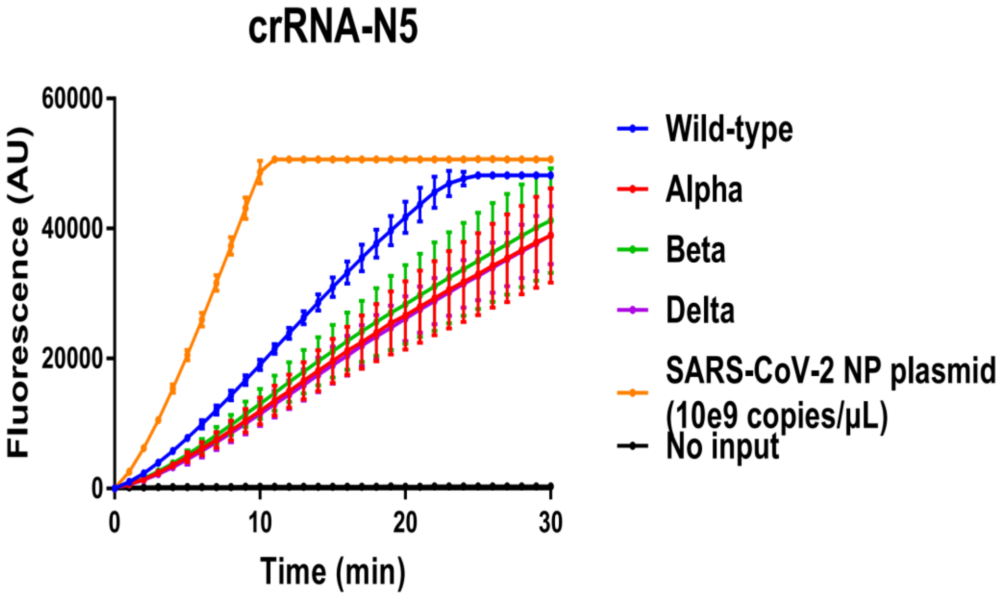

Supplementary Figure S3. CRISPR-Cas12a-based detection of SARS-CoV-2 NP gene. Viral RNA templates were extracted from SARS-CoV-2 wild-type strain as well as Alpha, Beta and Delta SARS-CoV-2 variants of concern and amplified by RT-PCR followed by CRISPR-Cas12a-mediated detection by using crRNA-N5. The SARS-CovV-2 NP plasmid DNA (10^9 copies/μL) was used as positive control. The fluorescence was measured at different time points and presented as the mean ± standard deviation (SD) from 3 replicates. No input, negative control with no plasmid DNA.
